# Supplementary material for: Assessing Geographic Overlap between Zero-Dose Diphtheria–Tetanus–Pertussis Vaccination Prevalence and Other Health Indicators
Source: Vaccines (Basel). 2023 Apr 5;11(4):802. doi: 10.3390/vaccines11040802 (PMC10144604; doi:10.3390/vaccines11040802)

Supplemental Materials

Table of Contents

Supplemental Methods....4

1.1 GATHER compliance....4

1.2 Geospatial modeling....4

1.3 Additional limitations....5

1.4 Supplemental References.... **Error! Bookmark not defined.**

Supplemental Tables....6

Supplemental Table S1: Ten countries with highest no-DTP counts in 2019 and indicator data availability details....6

Supplemental Table S2: Surveys included in DTP modeling....7

Supplemental Table S3: Surveys excluded from DTP modeling....39

Supplemental Table S4: GATHER Compliance checklist....44

Supplemental Table S5: Geospatial covariates used in modeling....46

Supplemental Figures....49

Supplemental Figures S1–S23: Country-specific AUC step plots....49

Supplemental Figure S1: Nigeria, U5M....50

Supplemental Figure S2: Nigeria, missed ITNs....51

Supplemental Figure S3: Nigeria, missed ORT....52

Supplemental Figure S4: Nigeria, LF....53

Supplemental Figure S5: Angola, stunting....54

Supplemental Figure S6: Angola, U5M....55

Supplemental Figure S7: Angola, missed ITNs....56

Supplemental Figure S8: Angola, missed ORT....57

Supplemental Figure S9: Angola, LF....58

Supplemental Figure S10: DRC, stunting....59

Supplemental Figure S11: DRC, U5M....60

Supplemental Figure S12: DRC, missed ITNs....61

Supplemental Figure S13: DRC, missed ORT....62

Supplemental Figure S14: DRC, LF....63

Supplemental Figure S15: Ethiopia, stunting....64

Supplemental Figure S16: Ethiopia, U5M....65

Supplemental Figure S17: Ethiopia, missed ITNs....66

Supplemental Figure S18: Ethiopia, missed ORT ... 67
Supplemental Figure S19: Ethiopia, LF ... 68
Supplemental Figure S20: Indonesia, stunting ... 69
Supplemental Figure S21: Indonesia, U5M ... 70
Supplemental Figure S22: Indonesia, missed ORT ... 71
Supplemental Figure S23: Indonesia, LF ... 72
Supplemental Figures S24–S46: Country-specific, all quartile overlap. ... 73
Supplemental Figure S24 Nigeria, U5M ... 74
Supplemental Figure S25: Nigeria, missed ITNs ... 75
Supplemental Figure S26: Nigeria, missed ORT ... 76
Supplemental Figure S27: Nigeria, LF ... 77
Supplemental Figure S28: Angola, stunting ... 78
Supplemental Figure S29: Angola, U5M ... 79
Supplemental Figure S30: Angola, missed ORT ... 80
Supplemental Figure S31: Angola, missed ITNs ... 81
Supplemental Figure S32: Angola, LF ... 82
Supplemental Figure S33: DRC, stunting ... 83
Supplemental Figure S34: DRC, U5M ... 84
Supplemental Figure S35: DRC, missed ITNs ... 85
Supplemental Figure S36: DRC, missed ORT ... 86
Supplemental Figure S37: DRC, LF ... 87
Supplemental Figure S38: Ethiopia, stunting ... 88
Supplemental Figure S39: Ethiopia, U5M ... 89
Supplemental Figure S40: Ethiopia, missed ITNs ... 90
Supplemental Figure S41: Ethiopia, missed ORT ... 91
Supplemental Figure S42: Ethiopia, LF ... 92
Supplemental Figure S43: Indonesia, stunting ... 93
Supplemental Figure S44: Indonesia, U5M ... 94
Supplemental Figure S45: Indonesia, missed ORT ... 95
Supplemental Figure S46: Indonesia, LF ... 96
Supplemental Figures S47–S69: Country-specific, highest quartile overlap. ... 97
Supplemental Figure S47: Nigeria, U5M ... 97
Supplemental Figure S48: Nigeria, missed ITNs ... 98
Supplemental Figure S49: Nigeria, missed ORT ... 99
Supplemental Figure S50: Nigeria, LF ... 100

Supplemental Figure S51: Angola, stunting 101
Supplemental Figure S52: Angola, U5M 102
Supplemental Figure S53: Angola, missed ITNs 103
Supplemental Figure S54: Angola, missed ORT 104
Supplemental Figure S55: Angola, LF 105
Supplemental Figure S56: DRC, stunting 106
Supplemental Figure S57: DRC, U5M 107
Supplemental Figure S58: DRC, missed ITNs 108
Supplemental Figure S59: DRC, missed ORT 109
Supplemental Figure S60: DRC, LF 110
Supplemental Figure S61: Ethiopia, stunting 111
Supplemental Figure S62: Ethiopia, U5M 112
Supplemental Figure S63: Ethiopia, missed ITNs 113
Supplemental Figure S64: Ethiopia, missed ORT 114
Supplemental Figure S65: Ethiopia, LF 115
Supplemental Figure S66: Indonesia, stunting 116
Supplemental Figure S67: Indonesia, U5M 117
Supplemental Figure S68: Indonesia, missed ORT 118
Supplemental Figure S69: Indonesia, LF 119
Supplemental Figures S70–S73: Multi-indicator overlap of country-specific highest quartiles. 120
Supplemental Figure S70: Angola 120
Supplemental Figure S71: DRC 121
Supplemental Figure S72: Ethiopia 122
Supplemental Figure S73: Indonesia 123
Supplemental Figures S74–S77: Multinational all quartile overlap by prevalence. 124
Supplemental Figure S74: U5M 124
Supplemental Figure S75: Missed ITNs 125
Supplemental Figure S76: Missed ORT 126
Supplemental Figure S77: LF 127
Supplemental Figures S78–S81: Multinational highest quartile overlap by prevalence. 128
Supplemental Figure S78: U5M 128
Supplemental Figure S79: Missed ITNs 129
Supplemental Figure S80: Missed ORT 130
Supplemental Figure S81: LF 131
Supplemental Figures S82–S85: Multinational all quartile overlap by counts. 132

Supplemental Figure S82: U5M 132
Supplemental Figure S83: Missed ITNs 133
Supplemental Figure S84: Missed ORT 134
Supplemental Figure S85: LF 135
Supplemental Figures S86–S89: Multinational highest quartile overlap by counts. 136
Supplemental Figure S86: U5M 136
Supplemental Figure S87: Missed ITNs 137
Supplemental Figure S88: Missed ORT 138
Supplemental Figure S89: LF 139

Supplemental Methods

1.1 GATHER compliance

Refer to Supplemental Table S4 for information on how this study meets GATHER compliance criteria [49].

1.2 Geospatial modeling

Portions of this section of the Supplemental Methods have been adapted from the methods sections and supplementary materials in Mosser *et al.* [17] and Sbarra *et al.* [18]. The geospatial model used here to produce estimates of the proportion of children aged 12-23 months who have not received any doses of diphtheria-tetanus-pertussis-containing vaccine (no-DTP) expands upon methods previously used to map DTP coverage in Africa [17]. Here, we have included additional surveys made available since this prior publication for each country included in the analysis (as well as surrounding countries), while also extending the annual time series through 2019. In brief, this model uses a Bayesian continuation ratio ordinal regression model-based geostatistical framework to produce spatiotemporal estimates of dose-specific DTP coverage under the assumption that coverage is similar in location-years that have similar covariate patterns and that are close together in space and time. To estimate coverage for the first dose of DTP (DTP1), we used a continuation ratio ordinal regression approach, whereby we first modelled coverage for the third dose of DTP (DTP3), as well as coverage of DTP1 or DTP2 conditional on having not received DTP3 (DTP1,2_{cond}). DTP1 coverage was then calculated as:

$$\text{DTP1} = \text{DTP1,}2_{cond} * (1 - \text{DTP3}) + \text{DTP3}$$

This method leverages all available DTP data and allows for internal consistency between DTP1 and DTP3 estimates at the draw level.

Along with the survey data, twenty-six geospatial covariates were selected for inclusion via a Variance Inflation Factor (VIF) algorithm to ensure a lack of collinearity [50] (Supplemental Table S5). Covariate selection was optimized per region. As previously, four national level covariates also included in the model: lag-distributed income, prevalence of the completion of the fourth antenatal care visit among pregnant women, mortality due to war and terror, and bias-adjusted national-level administrative coverage.

To allow for complex and non-linear interactions, we used a stacked ensemble modeling approach. Three child models – generalized additive models, lasso regression, and boosted regression trees – were first used to model covariate relationships and predict possible surfaces of dose-specific coverage probabilities. Then, we modelled the probability of dose-specific vaccination using a hierarchical logistic Bayesian geostatistical model. This model accounted for variation in coverage due to spatial and temporal correlation, as well as covariate patterns derived from the stacking child models. This second-step Bayesian geostatistical model used a generalized linear regression in binomial space with a logit link. A correlated spatiotemporal error term was modelled via a three-dimensional spatiotemporal Gaussian process with mean zero and covariance equal to the Kronecker product of temporal and spatial covariance kernels. Temporal covariance was modelled via an autoregressive order 1 function and spatial covariance was assumed to be an isotropic, stationary Matérn function. Also included were country-specific random effects, along with additional spatiotemporal residuals to account for any remaining variation beyond the predictive capability of modelled covariates, random effects, and observation-specific irreducible error. Models were fit in R version 4.2.1 and with the R-INLA package [51,52].

This modelling framework produced 500 Bayesian posterior samples of dose-specific coverage estimates for each 5x5-kilometer (km) area for years 2000 through 2019. Results were calibrated to updated national-level estimates from the Global Burden of Diseases, Injuries, and Risk Factors Study 2020 [3]. These pixel-level estimates were then aggregated based on fractional population weights to first- and second-level administrative units using population estimates from the WorldPop project and a modified version of the Database of Global Administrative Areas (GADM) shapefile [35,37]. Similar models have been validated previously using spatially stratified five-fold out-of-sample cross-validation and have yielded high predictive validity [17,18].

1.3 Additional limitations

This study is subject to several important additional limitations. First, these results are the product of a secondary analysis of modeled estimates of vaccination coverage and the spatial distribution of other health indicators. These modeled estimates allow for comparisons across space and time, including in years without available survey data, and at finer geographic resolutions than are typically available using traditional analytic methods. In cases where two geospatial models use similar predictive covariates, however, our approach may over-estimate the degree of correlation between indicators. Second, spatial smoothing inherent in geospatial modeling may underestimate true short-scale spatial variation in each indicator, especially if high-spatial-resolution data are not available. These approaches may be particularly limited in analyzing geographic overlap in dense urban settings as a result. Third, comparisons in these analyses relied on mean estimates of the different indicators and did not account for the uncertainty intervals associated with each of them.

Supplemental Tables

Supplemental Table S1: Ten countries with highest no-DTP counts in 2019 and indicator data availability details

Rank	Country	Children with no-DTP in 2019*	Stunting available?	U5M available?	Missed ITNs available?	Missed ORT available?	LF available?
1	Nigeria**	2,437,252	X	X	X	X	X
2	India	1,992,430	X			X	
3	DRC**	1,066,064	X	X	X	X	X
4	Ethiopia**	907,989	X	X	X	X	X
5	Indonesia**	804,887	X	X		X	X
6	Mexico	569,160	X			X	
7	Pakistan	549,225	X	X		X	
8	Angola**	318,400	X	X	X	X	X
9	Somalia	216,876	X	X	X	X	X
10	Chad	178,750	X	X	X	X	X

*Counts estimated according to geospatial modeling methods described for estimating coverage of DTP1, combined with under 1 population counts according to Worldpop [35].

**The five countries with highest estimated numbers of zero-dose children and available spatial estimates for at least 4 out of 5 of the comparator indicators were selected for inclusion in the overlap analyses.

Supplemental Table S2: Surveys included in DTP modeling

Country	GPS-located clusters	Areally-located clusters	Number of children included	Series	Years	Citation	Link
Angola	0	18	5617	Angola Multiple Indicator Cluster Survey 2001	2001	National Institute of Statistics (Angola), United Nations Children's Fund (UNICEF). Angola Multiple Indicator Cluster Survey 2001. New York, United States of America: United Nations Children's Fund (UNICEF).	GHDx
Angola	0	18	5710	Angola Integrated Inquiry into People's Well-Being 2008-2009	2008-2009	National Institute of Statistics (Angola), Oxford Policy Management, United Nations Children's Fund (UNICEF). Angola Integrated Inquiry into People's Well-Being 2008-2009.	GHDx
Angola	625	0	8470	Angola Demographic and Health Survey 2015-2016	2015-2016	ICF International, Ministry of Health (Angola), National Institute of Statistics (Angola), United Nations Children's Fund (UNICEF). Angola Demographic and Health Survey 2015-2016. Fairfax, United States of America: ICF International, 2017.	GHDx
Burundi	0	534	6308	Burundi Multiple Indicator Cluster Survey 2005	2005	United Nations Children's Fund (UNICEF), Burundi Institute of Statistics and Economic Studies, United Nations Population Fund (UNFPA). Burundi Multiple Indicator Cluster Survey 2005. New York, United States: United Nations Children's Fund (UNICEF).	GHDx
Burundi	376	0	7224	Burundi Demographic and Health Survey 2010-2011	2010-2011	Burundi Institute of Statistics and Economic Studies, ICF International, Ministry of Public Health and the Fight Against AIDS (Burundi). Burundi Demographic and Health Survey 2010-2011. Fairfax, United States of America: ICF International, 2012.	GHDx
Burundi	552	0	7591	Burundi Demographic and Health Survey 2016-2017	2016-2017	Burundi Institute of Statistics and Economic Studies, ICF International, Ministry of Public Health and the Fight Against AIDS (Burundi). Burundi Demographic and Health Survey 2016-2017. Fairfax, United States of America: ICF International, 2018.	GHDx
Benin	247	0	4703	Benin Demographic and Health Survey 2001	2001	National Institute of Statistics and Economic Analysis (INSAE) (Benin), ORC Macro. Benin Demographic and Health Survey 2001. Fairfax, United States of America: ICF International.	GHDx
Benin	0	12	13784	Benin Demographic	2006	Macro International, Inc, National Institute of Statistics and Economic Analysis (INSAE) (Benin), National Program	GHDx

Country	GPS-located clusters	Areally-located clusters	Number of children included	Series	Years	Citation	Link
				and Health Survey 2006		Against AIDS (PNLS) (Benin). Benin Demographic and Health Survey 2006. Fairfax, United States of America: ICF International.	
Benin	0	12	6310	Benin Multiple Indicator Cluster Survey 2014	2014	National Institute of Statistics and Economic Analysis (INSAE) (Benin), United Nations Children's Fund (UNICEF). Benin Multiple Indicator Cluster Survey 2014. New York, United States of America: United Nations Children's Fund (UNICEF), 2017.	GHDx
Benin	540	0	7586	Benin Demographic and Health Survey 2017-2018	2017-2018	Hubert Koutoukou Maga National University Hospital Center (CNHU-HKM)(Benin), ICF International, National Institute of Statistics and Economic Analysis (INSAE) (Benin), National Malaria Control Program, Ministry of Health (Benin), Permanent Secretariat of the Food Council and Nutrition (SP-CAN)(Benin). Benin Demographic and Health Survey 2017-2018. Fairfax, United States of America: ICF International, 2018.	GHDx
Burkina Faso	195	0	5096	Burkina Faso Multiple Indicator Cluster Survey 2006	2006	National Institute of Statistics and Demography (Burkina Faso), United Nations Children's Fund (UNICEF). Burkina Faso Multiple Indicator Cluster Survey 2006. New York, United States of America: United Nations Children's Fund (UNICEF).	GHDx
Burkina Faso	397	0	9229	Burkina Faso Demographic and Health Survey 2003	2003	Macro International, Inc, National Institute of Statistics and Demography (Burkina Faso). Burkina Faso Demographic and Health Survey 2003. Fairfax, United States of America: ICF International.	GHDx
Burkina Faso	541	0	12898	Burkina Faso Demographic and Health Survey 2010-2011	2010-2011	ICF Macro, Ministry of Health (Burkina Faso), National Institute of Statistics and Demography (Burkina Faso). Burkina Faso Demographic and Health Survey 2010-2011. Fairfax, United States of America: ICF International.	GHDx
Burkina Faso	0	13	5990	Burkina Faso Global Fund Household Health Coverage Survey 2008	2008	Global Fund to Fight Aids Tuberculosis and Malaria (GFATM). Burkina Faso Global Fund Household Health Coverage Survey 2008.	GHDx
Burkina Faso	0	13	6677	Burkina Faso Continuous Multisectoral Survey 2014	2014	National Institute of Statistics and Demography (Burkina Faso), World Bank. Burkina Faso Continuous Multisectoral Survey 2014. Washington DC, United States of America: World Bank.	GHDx

Country	GPS-located clusters	Areally-located clusters	Number of children included	Series	Years	Citation	Link
Central African Republic	0	17	12778	Central African Republic Multiple Indicator Cluster Survey 2000	2000	Division of Statistics and Economic and Social Studies (Central African Republic), Ministry of Economy, Planning and International Cooperation (Central African Republic), United Nations Children's Fund (UNICEF). Central African Republic Multiple Indicator Cluster Survey 2000. New York, United States of America: United Nations Children's Fund (UNICEF).	GHDx
Central African Republic	0	17	9311	Central African Republic Multiple Indicator Cluster Survey 2010-2011	2010-2011	Central African Institute of Statistics, Economic and Social Studies (ICASEES) (Central African Republic), ICF International. Central African Republic Multiple Indicator Cluster Survey 2010-2011. Fairfax, United States of America: ICF International, 2013.	GHDx
Côte d'Ivoire	341	0	6822	Côte d'Ivoire Demographic and Health Survey 2011-2012	2011-2012	ICF International, Ministry of the Fight Against AIDS (CÃ´te d'Ivoire), National Institute of Statistics (CÃ´te d'Ivoire). CÃ´te d'Ivoire Demographic and Health Survey 2011-2012. Fairfax, United States of America: ICF International.	GHDx
Côte d'Ivoire	0	11	3136	Côte d'Ivoire Multiple Indicator Cluster Survey 2006	2006	United Nations Children's Fund (UNICEF), National Institute of Statistics (CÃ´te d'Ivoire). CÃ´te d'Ivoire Multiple Indicator Cluster Survey 2006. New York, United States: United Nations Children's Fund (UNICEF).	GHDx
Côte d'Ivoire	0	11	7943	Côte d'Ivoire Multiple Indicator Cluster Survey 2000	2000	National School for Statistics and Economics Applied (ENSEA), United Nations Children's Fund (UNICEF), United Nations Educational, Scientific and Cultural Organization (UNESCO). CÃ´te d'Ivoire Multiple Indicator Cluster Survey 2000. New York, United States of America: United Nations Children's Fund (UNICEF).	GHDx
Cameroon	0	10	3403	Cameroon Multiple Indicator Cluster Survey 2000	2000	Directorate of Statistics and National Accounts, Ministry of Economics and Finance (Cameroon), United Nations Children's Fund (UNICEF). Cameroon Multiple Indicator Cluster Survey 2000. New York, United States of America: United Nations Children's Fund (UNICEF).	GHDx
Cameroon	0	218	5717	Cameroon Multiple Indicator Cluster Survey 2006	2006	United Nations Children's Fund (UNICEF), National Institute of Statistics (Cameroon). Cameroon Multiple Indicator Cluster Survey 2006. New York, United States: United Nations Children's Fund (UNICEF).	GHDx

Country	GPS-located clusters	Areally-located clusters	Number of children included	Series	Years	Citation	Link
Cameroon	461	0	7159	Cameroon Demographic and Health Survey 2004	2004	Macro International, Inc, National Institute of Statistics (Cameroon). Cameroon Demographic and Health Survey 2004. Fairfax, United States of America: ICF International.	GHDx
Cameroon	577	0	10629	Cameroon Demographic and Health Survey 2011	2011	ICF International, Ministry of Economy, Planning and Regional Development (Cameroon), Ministry of Public Health (Cameroon), National Institute of Statistics (Cameroon), Pasteur Center of Cameroon. Cameroon Demographic and Health Survey 2011. Fairfax, United States of America: ICF International.	GHDx
Cameroon	0	12	3345	Cameroon Multiple Indicator Cluster Survey 2014	2014	Ministry of Public Health (Cameroon), National Institute of Statistics (Cameroon), United Nations Children's Fund (UNICEF). Cameroon Multiple Indicator Cluster Survey 2014. New York, United States of America: United Nations Children's Fund (UNICEF), 2017.	GHDx
Cameroon	429	0	5487	Cameroon Demographic and Health Survey 2018-2019	2018-2019	ICF International, Ministry of Public Health (Cameroon), National Institute of Statistics (Cameroon). Cameroon Demographic and Health Survey 2018-2019. Fairfax, United States of America: ICF International.	GHDx
Democratic Republic of the Congo	0	11	2023	Democratic Republic of the Congo Multiple Indicator Cluster Survey 2001	2001	Ministry of Planning and Reconstruction (Congo, DR), United Nations Children's Fund (UNICEF). Congo, DR Multiple Indicator Cluster Survey 2001. New York, United States: United Nations Children's Fund (UNICEF).	GHDx
Democratic Republic of the Congo	293	0	7622	Democratic Republic of the Congo Demographic and Health Survey 2007	2007	Macro International, Inc, Ministry of Planning (Congo, DR). Democratic Republic of the Congo Demographic and Health Survey 2007. Fairfax, United States of America: ICF International.	GHDx
Democratic Republic of the Congo	357	0	10109	Democratic Republic of the Congo Multiple Indicator Cluster Survey 2010	2010	National Statistical Institute (Congo, DR), Ministry of Planning (Congo, DR), United Nations Children's Fund (UNICEF). Congo, DR Multiple Indicator Cluster Survey 2010. New York, United States: United Nations Children's Fund (UNICEF).	GHDx

Country	GPS-located clusters	Areally-located clusters	Number of children included	Series	Years	Citation	Link
Democratic Republic of the Congo	492	0	15808	Democratic Republic of the Congo Demographic and Health Survey 2013-2014	2013-2014	ICF International, Ministry of Planning and Monitoring Implementation of the Revolution of Modernity (Congo, DR), Ministry of Public Health (Congo, DR), National Institute of Statistics (Congo, DR). Democratic Republic of the Congo Demographic and Health Survey 2013-2014. Fairfax, United States of America: ICF International, 2014.	GHDx
Democratic Republic of the Congo	0	26	6899	Democratic Republic of the Congo Multiple Indicator Cluster Survey 2017-2018	2017-2018	National Statistical Institute (Congo, DR), Ministry of Planning (Congo, DR), United Nations Children's Fund (UNICEF). Congo, DR Multiple Indicator Cluster Survey 2010. New York, United States: United Nations Children's Fund (UNICEF).	GHDx
Democratic Republic of the Congo	0	497	43461	Democratic Republic of the Congo Vaccination Coverage Survey among Children from 6 to 23 Months 2021-2022	2021-2022	School of Public Health, University of Kinshasa. Democratic Republic of the Congo Vaccination Coverage Survey among Children from 6 to 23 Months 2021-2022.	GHDx
Congo	0	12	1487	Congo Demographic and Health Survey 2005	2005	Macro International, Inc, National Center for Statistics and Economic Studies (Congo, Rep.). Congo Demographic and Health Survey 2005. Fairfax, United States of America: ICF International.	GHDx
Congo	0	12	7703	Congo Demographic and Health Survey 2011-2012	2011-2012	ICF International, Ministry of Health (Congo, Rep.), National Center for Statistics and Economic Studies (Congo, Rep.). Congo Demographic and Health Survey 2011-2012. Fairfax, United States of America: ICF International.	GHDx
Congo	0	11	4547	Congo Multiple Indicator Cluster Survey 2014-2015	2014-2015	National Institute of Statistics (INS) (Congo, Rep.), United Nations Children's Fund (UNICEF). Congo Multiple Indicator Cluster Survey 2014-2015. New York, United States of America: United Nations Children's Fund (UNICEF), 2018.	GHDx
Comoros	0	3	4431	Comoros Multiple Indicator	2000	United Nations Development Programme (UNDP), United Nations Children's Fund (UNICEF). Comoros Multiple	GHDx

Country	GPS-located clusters	Areally-located clusters	Number of children included	Series	Years	Citation	Link
				Cluster Survey 2000		Indicator Cluster Survey 2000. New York, United States: United Nations Children's Fund (UNICEF).	
Comoros	242	0	2818	Comoros Demographic and Health Survey 2012-2013	2012-2013	General Directorate of Statistics and Forecasting (Comoros), ICF International. Comoros Demographic and Health Survey 2012-2013. Fairfax, United States of America: ICF International.	GHDx
Cabo Verde	0	9	381	Cape Verde Demographic and Health Survey 2005	2005	Macro International, Inc, Ministry of Health (Cape Verde), National Institute of Statistics (Cape Verde). Cape Verde Demographic and Health Survey 2005.	GHDx
Djibouti	0	1	549	Djibouti Family Health Survey 2002	2002	Department of Statistics and Demographic Studies (Djibouti), League of Arab States, Ministry of Health (Djibouti), Pan Arab Project for Family Health (PAPFAM). Djibouti Family Health Survey 2002.	GHDx
Djibouti	35	1	1871	Djibouti Multiple Indicator Cluster Survey 2006	2006	Ministry of Economy, Finance, and Planning in charge of Privatization (Djibouti), Ministry of Health (Djibouti), United Nations Children's Fund (UNICEF). Djibouti Multiple Indicator Cluster Survey 2006. New York, United States of America: United Nations Children's Fund (UNICEF).	GHDx
Djibouti	0	6	783	Djibouti Family Health Survey 2012	2012	Department of Statistics and Demographic Studies (Djibouti), League of Arab States, Ministry of Health (Djibouti), Pan Arab Project for Family Health (PAPFAM). Djibouti Family Health Survey 2012.	GHDx
Eritrea	0	6	5155	Eritrea Demographic and Health Survey 2002	2002	Macro International, Inc, National Statistics and Evaluation Office (Eritrea). Eritrea Demographic and Health Survey 2002. Fairfax, United States of America: ICF International.	GHDx
Eritrea	0	6	1157	Eritrea Population and Health Survey 2010	2010	Kenya Medical Research Institute (KEMRI), National Statistics Office (Eritrea), The Fafo Research Foundation. Eritrea Population and Health Survey 2010.	GHDx
Ethiopia	528	0	8740	Ethiopia Demographic and Health Survey 2005	2005	Macro International, Inc, Population and Housing Census Commissions Office (PHCCO). Ethiopia Demographic and Health Survey 2005. Fairfax, United States of America: ICF International.	GHDx
Ethiopia	533	0	9387	Ethiopia Demographic and Health Survey 2000	2000	Central Statistics Agency (Ethiopia), ORC Macro. Ethiopia Demographic and Health Survey 2000. Calverton, United States of America: ORC Macro, 2001.	GHDx

Country	GPS-located clusters	Areally-located clusters	Number of children included	Series	Years	Citation	Link
Ethiopia	571	0	10321	Ethiopia Demographic and Health Survey 2010-2011	2010-2011	Central Statistics Agency (Ethiopia), ICF Macro, Ministry of Health (Ethiopia). Ethiopia Demographic and Health Survey 2010-2011. Fairfax, United States of America: ICF International.	GHDx
Ethiopia	620	0	5711	Ethiopia Demographic and Health Survey 2016	2016	Central Statistics Agency (Ethiopia), ICF International. Ethiopia Demographic and Health Survey 2016. Fairfax, United States of America: ICF International, 2017.	GHDx
Ethiopia	0	11	2661	Ethiopia Interim Demographic and Health Survey 2019	2019	Central Statistics Agency (Ethiopia), Ethiopian Public Health Institute (EPHI), ICF International, Ministry of Health (Ethiopia). Ethiopia Interim Demographic and Health Survey 2019.	GHDx
Gabon	0	40	4051	Gabon Demographic and Health Survey 2000-2001	2000-2001	General Directorate of Statistics and Economic Studies (Gabon), Macro International, Inc. Gabon Demographic and Health Survey 2000-2001. Fairfax, United States of America: ICF International.	GHDx
Gabon	330	0	5606	Gabon Demographic and Health Survey 2012	2012	General Directorate of Statistics (Gabon), ICF International, Ministry of Economy, Employment and Sustainable Development (Gabon), Ministry of Health (Gabon). Gabon Demographic and Health Survey 2012. Fairfax, United States of America: ICF International, 2013.	GHDx
Ghana	0	10	618	Ghana Multiple Indicator Cluster Survey 2006	2006	Ministry of Health (MOH) (Ghana), Ghana Statistical Service and United Nations Children's Fund (UNICEF). Ghana Multiple Indicator Cluster Survey 2006. New York, United States: United Nations Children's Fund (UNICEF).	GHDx
Ghana	408	0	3479	Ghana Demographic and Health Survey 2003	2003	Ghana Statistical Service, Macro International, Inc. Ghana Demographic and Health Survey 2003. Fairfax, United States of America: ICF International.	GHDx
Ghana	401	0	2727	Ghana Demographic and Health Survey 2008	2008	Ghana Statistical Service, Macro International, Inc, Ministry of Health (Ghana). Ghana Demographic and Health Survey 2008. Fairfax, United States of America: ICF International.	GHDx
Ghana	805	0	7451	Ghana Multiple Indicator	2011	Centers for Disease Control and Prevention (CDC), Ghana Statistical Service, Government of Japan, ICF Macro, Ministry of Health (Ghana), Navrongo Health Research Centre, United	GHDx

Country	GPS-located clusters	Areally-located clusters	Number of children included	Series	Years	Citation	Link
				Cluster Survey 2011		Nations Children's Fund (UNICEF), United Nations Population Fund (UNFPA), United States Agency for International Development (USAID). Ghana Multiple Indicator Cluster Survey 2011. New York, United States of America: United Nations Children's Fund (UNICEF), 2013.	
Ghana	422	0	5499	Ghana Demographic and Health Survey 2014	2014	Ghana Health Service, Ghana Statistical Service, ICF International. Ghana Demographic and Health Survey 2014. Fairfax, United States of America: ICF International, 2016.	GHDx
Ghana	0	10	3274	Ghana Multiple Indicator Cluster Survey 2017-2018	2017-2018	Centers for Disease Control and Prevention (CDC), Ghana Statistical Service, Government of Japan, ICF Macro, Ministry of Health (Ghana), Navrongo Health Research Centre, United Nations Children's Fund (UNICEF), United Nations Population Fund (UNFPA), United States Agency for International Development (USAID). Ghana Multiple Indicator Cluster Survey 2017-2018. New York, United States of America: United Nations Children's Fund (UNICEF), 2013.	GHDx
Guinea	291	0	5465	Guinea Demographic and Health Survey 2005	2005	Macro International, Inc, National Statistics Directorate (Guinea). Guinea Demographic and Health Survey 2005. Fairfax, United States of America: ICF International.	GHDx
Guinea	300	0	6414	Guinea Demographic and Health Survey 2012	2012	ICF Macro, Ministry of Health and Public Hygiene (Guinea), National Institute of Statistics (Guinea). Guinea Demographic and Health Survey 2012. Fairfax, United States of America: ICF International.	GHDx
Guinea	0	8	3867	Guinea Multiple Indicator Cluster Survey 2016	2016	National Institute of Public Health (NPHI) (Guinea), National Institute of Statistics (Guinea), National Malaria Control Program (Guinea), United Nations Children's Fund (UNICEF). Guinea Multiple Indicator Cluster Survey 2016. New York, United States of America: United Nations Children's Fund (UNICEF), 2018.	GHDx
Guinea	400	0	4211	Guinea Demographic and Health Survey 2018	2018	Ministry of Health (Guinea), Ministry of Planning and Economic Development (Guinea), National Institute of Statistics (Guinea). Guinea Demographic and Health Survey 2018. Fairfax, United States of America: ICF International, 2019.	GHDx
Gambia	0	7	3153	Gambia Multiple Indicator	2000	Central Statistics Department (Gambia), United Nations Children's Fund (UNICEF). Gambia Multiple Indicator Cluster Survey 2000. New York, United States: United Nations Children's Fund (UNICEF).	GHDx

Country	GPS-located clusters	Areally-located clusters	Number of children included	Series	Years	Citation	Link
				Cluster Survey 2000			
Gambia	0	35	6252	Gambia Multiple Indicator Cluster Survey 2005-2006	2005-2006	Gambia Bureau of Statistics (GBOS), United Nations Children's Fund (UNICEF). Gambia Multiple Indicator Cluster Survey 2005-2006. New York, United States of America: United Nations Children's Fund (UNICEF).	GHDx
Gambia	0	36	6394	Gambia Demographic and Health Survey 2013	2013	Gambia Bureau of Statistics (GBOS), ICF International, Ministry of Health and Social Welfare (The Gambia). Gambia Demographic and Health Survey 2013. Fairfax, United States of America: ICF International, 2015.	GHDx
Gambia	0	5	7578	Gambia Multiple Indicator Cluster Survey 2010	2010	Gambia Bureau of Statistics (GBOS), United Nations Children's Fund (UNICEF). Gambia Multiple Indicator Cluster Survey 2010. New York, United States of America: United Nations Children's Fund (UNICEF), 2018.	GHDx
Gambia	0	7	4907	Gambia Multiple Indicator Cluster Survey 2018	2018	Gambia Bureau of Statistics (GBOS), United Nations Children's Fund (UNICEF). Gambia Multiple Indicator Cluster Survey 2018. New York, United States of America: United Nations Children's Fund (UNICEF), 2019.	GHDx
Gambia	0	7	4395	Gambia Demographic and Health Survey 2019-2020	2019-2020	Gambia Bureau of Statistics (GBOS), ICF International, Ministry of Health and Social Welfare (The Gambia). Gambia Demographic and Health Survey 2019-2020. Fairfax, United States of America: ICF International, 2021.	GHDx
Guinea-Bissau	0	9	4575	Guinea-Bissau Multiple Indicator Cluster Survey 2000	2000	Secretary State of Planning, National Institute of Statistics and Census (INEC), United Nations Children's Fund (UNICEF). Guinea-Bissau Multiple Indicator Cluster Survey 2000. New York, United States: United Nations Children's Fund (UNICEF).	GHDx
Guinea-Bissau	0	9	5111	Guinea-Bissau Multiple Indicator Cluster Survey 2006	2006	United Nations Children's Fund (UNICEF), Government of Guinea-Bissau. Guinea-Bissau Multiple Indicator Cluster Survey 2006. New York, United States: United Nations Children's Fund (UNICEF).	GHDx
Guinea-Bissau	0	9	4020	Guinea-Bissau Multiple Indicator	2014	National Statistics Institute (Guinea-Bissau), United Nations Children's Fund (UNICEF). Guinea-Bissau Multiple Indicator Cluster Survey 2014. New York, United States of America: United Nations Children's Fund (UNICEF), 2016.	GHDx

Country	GPS-located clusters	Areally-located clusters	Number of children included	Series	Years	Citation	Link
				Cluster Survey 2014			
Guinea-Bissau	0	8	3764	Guinea-Bissau Multiple Indicator Cluster Survey 2018-2019	2018-2019	National Statistics Institute (Guinea-Bissau), United Nations Children's Fund (UNICEF). Guinea-Bissau Multiple Indicator Cluster Survey 2018-2019. New York, United States of America: United Nations Children's Fund (UNICEF), 2020.	GHDx
Equatorial Guinea	0	7	2929	Equatorial Guinea Multiple Indicator Cluster Survey 2000	2000	Ministry of Planning, Economic Development and Public Investment (Equatorial Guinea), United Nations Children's Fund (UNICEF). Equatorial Guinea Multiple Indicator Cluster Survey 2000. New York, United States of America: United Nations Children's Fund (UNICEF).	GHDx
Equatorial Guinea	0	2	462	Equatorial Guinea Demographic and Health Survey 2011	2011	ICF International, Ministry of Health and Social Welfare (Equatorial Guinea), Ministry of Planning, Economic Development and Public Investment (Equatorial Guinea). Equatorial Guinea Demographic and Health Survey 2011. Fairfax, United States of America: ICF International, 2012.	GHDx
Indonesia	1315	0	14622	Indonesia Demographic and Health Survey 2002-2003	2002-2003	Macro International, Inc, Ministry of Health (Indonesia), National Family Planning Coordinating Board (Indonesia), Statistics Indonesia. Indonesia Demographic and Health Survey 2002-2003. Fairfax, United States of America: ICF International.	GHDx
Indonesia	0	33	14769	Indonesia Demographic and Health Survey 2007	2007	Macro International, Inc, Ministry of Health (Indonesia), National Family Planning Coordinating Board (Indonesia), Statistics Indonesia. Indonesia Demographic and Health Survey 2007. Fairfax, United States of America: ICF International.	GHDx
Indonesia	0	33	16645	Indonesia Demographic and Health Survey 2012	2012	ICF International, Ministry of Health (Indonesia), National Population and Family Planning Board (Indonesia), Statistics Indonesia. Indonesia Demographic and Health Survey 2012. Fairfax, United States of America: ICF International.	GHDx
Indonesia	0	3	1248	Indonesia - West Papua Multiple Indicator Cluster Survey 2011	2011	Ministry of Home Affairs (Indonesia), National Development Planning Agency (BAPPENAS) (Indonesia), Statistics Indonesia, United Nations Children's Fund (UNICEF). Indonesia - West Papua Multiple Indicator Cluster Survey 2011. New York, United States of America: United Nations Children's Fund (UNICEF), 2013.	GHDx
Indonesia	0	3	1258	Indonesia - Papua Multiple	2011	Ministry of Home Affairs (Indonesia), National Development Planning Agency (BAPPENAS) (Indonesia), Statistics	GHDx

Country	GPS-located clusters	Areally-located clusters	Number of children included	Series	Years	Citation	Link
				Indicator Cluster Survey 2011		Indonesia, United Nations Children's Fund (UNICEF). Indonesia - Papua Multiple Indicator Cluster Survey 2011. New York, United States of America: United Nations Children's Fund (UNICEF), 2013.	
Indonesia	0	1058	4436	Indonesia Family Life Survey 2014-2015	2014-2015	RAND Corporation, SurveyMETER. Indonesia Family Life Survey 2014-2015. Santa Monica, United States of America: RAND Corporation, 2016.	GHDx
Indonesia	0	33	9367	Indonesia Demographic and Health Survey 2017	2017	Ministry of Health (Indonesia), National Population and Family Planning Board (Indonesia), Statistics Indonesia. Indonesia Demographic and Health Survey 2017. Fairfax, United States of America: ICF International, 2019.	GHDx
Indonesia	0	34	53816	Indonesia National Socioeconomic Survey 2017	2017	Central Bureau of Statistics (Indonesia). Indonesia National Socioeconomic Survey 2017. Jakarta, Indonesia: Central Bureau of Statistics (Indonesia), 2018.	GHDx
Indonesia	0	3	20	Indonesia Special Demographic and Health Survey 2017	2017	ICF International, Ministry of Health (Indonesia), National Population and Family Planning Board (Indonesia), Statistics Indonesia. Indonesia Special Demographic and Health Survey 2017. Fairfax, United States of America: ICF International, 2019.	GHDx
Kenya	1310	0	7587	Kenya Integrated Household Budget Survey 2005-2006	2005-2006	Central Bureau of Statistics (Kenya), UK Department for International Development (DFID), United States Agency for International Development (USAID), European Union (EU), Danish International Development Agency (DANIDA), World Bank (WB), United Nations Development Programme (UNDP). Kenya Integrated Household Budget Survey 2005-2006. Nairobi, Kenya: Central Bureau of Statistics (Kenya).	GHDx
Kenya	803	0	7266	Kenya Multiple Indicator Cluster Survey 2000	2000	Central Bureau of Statistics (Kenya), United Nations Children's Fund (UNICEF). Kenya Multiple Indicator Cluster Survey 2000. New York, United States: United Nations Children's Fund (UNICEF).	GHDx
Kenya	590	0	13936	Kenya - Eastern Province Multiple Indicator Cluster Survey 2008	2008	Kenya National Bureau of Statistics, United Nations Children's Fund (UNICEF). Kenya - Eastern Province Multiple Indicator Cluster Survey 2008. Nairobi, Kenya: Kenya National Bureau of Statistics.	GHDx

Country	GPS-located clusters	Areally-located clusters	Number of children included	Series	Years	Citation	Link
Kenya	397	0	5677	Kenya Demographic and Health Survey 2008-2009	2008-2009	ICF Macro, Kenya Medical Research Institute (KEMRI), Kenya National Bureau of Statistics, Ministry of Public Health and Sanitation (Kenya), National AIDS and STI Control Programme (NASCOP) (Kenya), National Association of County and City Health Officials (NACCHO) (United States), National Coordinating Agency for Population and Development (Kenya). Kenya Demographic and Health Survey 2008-2009. Fairfax, United States of America: ICF International.	GHDx
Kenya	0	1	446	Kenya - Coast Multiple Indicator Cluster Survey 2009	2009	Kenya National Bureau of Statistics, United Nations Children's Fund (UNICEF). Kenya - Coast Multiple Indicator Cluster Survey 2009. New York, United States of America: United Nations Children's Fund (UNICEF), 2014.	GHDx
Kenya	289	0	4803	Kenya - Nyanza Province Multiple Indicator Cluster Survey 2011	2011	Kenya National Bureau of Statistics, United Nations Children's Fund (UNICEF). Kenya - Nyanza Province Multiple Indicator Cluster Survey 2011. Nairobi, Kenya: Kenya National Bureau of Statistics.	GHDx
Kenya	1584	0	19942	Kenya Demographic and Health Survey 2014	2014	ICF International, Kenya Medical Research Institute (KEMRI), Kenya National Bureau of Statistics, Ministry of Health (Kenya), National AIDS Control Council (Kenya), National Council for Population and Development (Kenya). Kenya Demographic and Health Survey 2014. Fairfax, United States of America: ICF International.	GHDx
Kenya	40	0	831	Kenya - Bungoma County Multiple Indicator Survey 2013-2014	2013-2014	Kenya National Bureau of Statistics, Population Studies and Research Institute, University of Nairobi (Kenya), United Nations Children's Fund (UNICEF). Kenya - Bungoma County Multiple Indicator Survey 2013-2014. New York, United States of America: United Nations Children's Fund (UNICEF), 2015.	GHDx
Kenya	48	0	762	Kenya - Kakamega County Multiple Indicator	2013-2014	Kenya National Bureau of Statistics, Population Studies and Research Institute, University of Nairobi (Kenya), United Nations Children's Fund (UNICEF). Kenya - Kakamega County Multiple Indicator Survey 2013-2014. New York, United States of America: United Nations Children's Fund (UNICEF), 2015.	GHDx

Country	GPS-located clusters	Areally-located clusters	Number of children included	Series	Years	Citation	Link
				Survey 2013-2014			
Kenya	50	0	1028	Kenya - Turkana County Multiple Indicator Survey 2013-2014	2013-2014	Kenya National Bureau of Statistics, Population Studies and Research Institute, University of Nairobi (Kenya), United Nations Children's Fund (UNICEF). Kenya - Turkana County Multiple Indicator Survey 2013-2014. New York, United States of America: United Nations Children's Fund (UNICEF), 2015.	GHDx
Cambodia	470	0	7772	Cambodia Demographic and Health Survey 2000	2000	Macro International, Inc, Ministry of Health (Cambodia), National Institute of Statistics (Cambodia). Cambodia Demographic and Health Survey 2000. Fairfax, United States of America: ICF International.	GHDx
Cambodia	548	0	7490	Cambodia Demographic and Health Survey 2005-2006	2005-2006	Macro International, Inc, National Institute of Public Health (Cambodia), National Institute of Statistics (Cambodia). Cambodia Demographic and Health Survey 2005-2006. Fairfax, United States of America: ICF International.	GHDx
Cambodia	607	0	7742	Cambodia Demographic and Health Survey 2010-2011	2010-2011	ICF Macro, Ministry of Health (Cambodia), National Institute of Statistics (Cambodia). Cambodia Demographic and Health Survey 2010-2011. Fairfax, United States of America: ICF International.	GHDx
Cambodia	258	17	1264	Cambodia Socio-Economic Survey 2003-2005	2003-2005	National Institute of Statistics (Cambodia), Statistics Sweden. Cambodia Socio-Economic Survey 2003-2005. Phnom Penh, Cambodia: National Institute of Statistics (Cambodia).	GHDx
Cambodia	242	0	587	Cambodia Socio-Economic Survey 2006-2007	2006-2007	National Institute of Statistics (Cambodia), Statistics Sweden. Cambodia Socio-Economic Survey 2006-2007. Phnom Penh, Cambodia: National Institute of Statistics (Cambodia).	GHDx
Cambodia	609	0	6962	Cambodia Demographic and Health Survey 2014	2014	ICF International, Ministry of Health (Cambodia), National Institute of Statistics (Cambodia). Cambodia Demographic and Health Survey 2014. Fairfax, United States of America: ICF International, 2017.	GHDx
Lao People's Democratic Republic	0	3	848	Laos Multiple Indicator	2000	Ministry of Health (Laos), National Institute of Public Health (NIOPH), National Statistical Center (Laos), United Nations Children's Fund (UNICEF). Laos Multiple Indicator Cluster	GHDx

Country	GPS-located clusters	Areally-located clusters	Number of children included	Series	Years	Citation	Link
				Cluster Survey 2000		Survey 2000. New York, United States of America: United Nations Children's Fund (UNICEF).	
Lao People's Democratic Republic	300	0	4093	Laos Multiple Indicator Cluster Survey 2006	2006	United Nations Children's Fund (UNICEF), Department of Statistics (Laos), Ministry of Health (Laos). Laos Multiple Indicator Cluster Survey 2006. New York, United States: United Nations Children's Fund (UNICEF).	GHDx
Lao People's Democratic Republic	0	17	1873	Laos Multiple Indicator Cluster Survey 2011-2012	2011-2012	Ministry of Education and Sports (Laos), Ministry of Health (Laos), Ministry of Planning and Investment (Laos). Laos Multiple Indicator Cluster Survey 2011-2012. New York, United States of America: United Nations Children's Fund (UNICEF), 2013.	GHDx
Lao People's Democratic Republic	0	18	6431	Laos Multiple Indicator Cluster Survey 2017	2017	Lao Statistics Bureau, Ministry of Education and Sports (Laos), Ministry of Health (Laos), United Nations Children's Fund (UNICEF). Laos Multiple Indicator Cluster Survey 2017. New York, United States of America: United Nations Children's Fund (UNICEF), 2018.	GHDx
Liberia	290	0	5131	Liberia Demographic and Health Survey 2006-2007	2006-2007	Liberia Institute for Statistics and Geo-information Services (LISGIS), Macro International, Inc. Liberia Demographic and Health Survey 2006-2007. Fairfax, United States of America: ICF International.	GHDx
Liberia	322	0	7015	Liberia Demographic and Health Survey 2013	2013	ICF International, Liberia Institute for Statistics and Geo-information Services (LISGIS), National AIDS and STI Control Program (NACP), Ministry of Health and Social Welfare (Liberia). Liberia Demographic and Health Survey 2013. Fairfax, United States of America: ICF International.	GHDx
Liberia	150	0	1725	Liberia Malaria Indicator Survey 2016	2016	ICF International, Liberia Institute for Statistics and Geo-information Services (LISGIS), National Malaria Control Program (Liberia). Liberia Malaria Indicator Survey 2016. Fairfax, United States of America: ICF International, 2017.	GHDx
Liberia	321	0	3125	Liberia Demographic and Health Survey 2019-2020	2019-2020	ICF International, Liberia Institute for Statistics and Geo-information Services (LISGIS), Ministry of Health and Social Welfare (Liberia). Liberia Demographic and Health Survey 2019-2020. 2021.	GHDx
Madagascar	0	6	2848	Madagascar Demographic and Health Survey 2003-2004	2003-2004	Macro International, Inc, National Institute of Statistics (Madagascar). Madagascar Demographic and Health Survey 2003-2004. Fairfax, United States of America: ICF International.	GHDx

Country	GPS-located clusters	Areally-located clusters	Number of children included	Series	Years	Citation	Link
Madagascar	585	0	11510	Madagascar Demographic and Health Survey 2008-2009	2008-2009	ICF Macro, National Institute of Statistics (Madagascar). Madagascar Demographic and Health Survey 2008-2009. Fairfax, United States of America: ICF International.	GHDx
Madagascar	127	0	2933	Madagascar - South Multiple Indicator Cluster Survey 2012	2012	National Institute of Statistics (Madagascar), United Nations Children's Fund (UNICEF). Madagascar - South Multiple Indicator Cluster Survey 2012. New York, United States of America: United Nations Children's Fund (UNICEF), 2015.	GHDx
Madagascar	772	0	7650	Madagascar Multiple Indicator Cluster Survey 2018	2018	National Institute of Statistics (Madagascar), United Nations Children's Fund (UNICEF). Madagascar Multiple Indicator Cluster Survey 2018. 2019.	GHDx
Madagascar	649	0	7200	Madagascar Demographic and Health Survey 2021	2021	ICF Macro, National Institute of Statistics (Madagascar). Madagascar Demographic and Health Survey 2021. Fairfax, United States of America: ICF International, 2022.	GHDx
Madagascar	0	22	3279	Madagascar Vaccine Coverage Survey 2021	2021	Ministry of Public Health (Madagascar), National Institute of Statistics (Madagascar). Madagascar Vaccine Coverage Survey 2021.	GHDx
Mali	405	0	12206	Mali Demographic and Health Survey 2006	2006	Macro International, Inc, Ministry of Health (Mali), National Directorate of Statistics and Informatics (DNSI) (Mali). Mali Demographic and Health Survey 2006. Fairfax, United States of America: ICF International.	GHDx
Mali	399	0	10824	Mali Demographic and Health Survey 2001	2001	Macro International, Inc, National Directorate of Statistics and Informatics (DNSI) (Mali), Planning and Statistics Unit, Ministry of Health (Mali). Mali Demographic and Health Survey 2001. Fairfax, United States of America: ICF International.	GHDx
Mali	413	0	9582	Mali Demographic and Health Survey 2012-2013	2012-2013	ICF International, INFO-STAT (Mali), Ministry of Health (Mali), National Institute of Statistics (INSTAT) (Mali), Planning and Statistics Unit, Ministry of Health (Mali). Mali Demographic and Health Survey 2012-2013. Fairfax, United States of America: ICF International, 2014.	GHDx
Mali	0	8	7791	Mali Multiple Indicator	2015	Ministry of Health (Mali), Ministry of Planning (Mali), National Institute of Statistics (INSTAT) (Mali), United Nations Children's Fund (UNICEF). Mali Multiple Indicator	GHDx

Country	GPS-located clusters	Areally-located clusters	Number of children included	Series	Years	Citation	Link
				Cluster Survey 2015		Cluster Survey 2015. New York, United States of America: United Nations Children's Fund (UNICEF), 2017.	
Mali	0	44	3387	Mali Agricultural Integrated Economic Survey 2014-2015	2014-2015	Ministry of Rural Development (Mali), National Institute of Statistics (INSTAT) (Mali), World Bank. Mali Agricultural Integrated Economic Survey 2014-2015. Washington DC, United States of America: World Bank.	GHDx
Mali	328	0	5398	Mali Demographic and Health Survey 2018	2018	ICF International, National Institute of Statistics (INSTAT) (Mali), Sector Planning and Statistics Unit Health-Social Development and Family Promotion (CPS / SS-DS-PF) (Mali). Mali Demographic and Health Survey 2018. Fairfax, United States of America: ICF International, 2019.	GHDx
Myanmar	0	16	14431	Myanmar Multiple Indicator Cluster Survey 2000	2000	Ministry of Health (Myanmar), United Nations Children's Fund (UNICEF). Myanmar Multiple Indicator Cluster Survey 2000. New York, United States of America: United Nations Children's Fund (UNICEF).	GHDx
Myanmar	0	17	14764	Myanmar Multiple Indicator Cluster Survey 2009-2010	2009-2010	Ministry of Health (Myanmar), Ministry of National Planning and Economic Development (Myanmar), United Nations Children's Fund (UNICEF). Myanmar Multiple Indicator Cluster Survey 2009-2010.	GHDx
Myanmar	0	17	10357	Myanmar Multiple Indicator Cluster Survey 2003	2003	Ministry of Health (Myanmar), United Nations Children's Fund (UNICEF). Myanmar Multiple Indicator Cluster Survey 2003.	GHDx
Myanmar	440	0	4593	Myanmar Demographic and Health Survey 2015-2016	2015-2016	ICF International, Ministry of Health and Sports (Myanmar). Myanmar Demographic and Health Survey 2015-2016. Fairfax, United States of America: ICF International, 2017.	GHDx
Mozambique	0	11	6769	Mozambique Demographic and Health Survey 2003-2004	2003-2004	Macro International, Inc, National Institute of Statistics (INE) (Mozambique). Mozambique Demographic and Health Survey 2003-2004. Fairfax, United States of America: ICF International.	GHDx
Mozambique	67	593	10566	Mozambique Multiple	2008-2009	United Nations Children's Fund (UNICEF), National Statistics Institute (Mozambique). Mozambique Multiple Indicator	GHDx

Country	GPS-located clusters	Areally-located clusters	Number of children included	Series	Years	Citation	Link
				Indicator Cluster Survey 2008-2009		Cluster Survey 2008-2009. New York, United States: United Nations Children's Fund (UNICEF).	
Mozambique	609	0	10255	Mozambique Demographic and Health Survey 2011	2011	ICF Macro, Manhica Health Research Center (CISM), Ministry of Health (Mozambique), National Institute of Statistics (INE) (Mozambique). Mozambique Demographic and Health Survey 2011. Fairfax, United States of America: ICF International.	GHDx
Mozambique	306	0	4695	Mozambique AIDS Indicator Survey 2015	2015	Centers for Disease Control and Prevention (CDC), ICF International, Ministry of Health (Mozambique), National Institute of Health (Mozambique), National Institute of Statistics (INE) (Mozambique). Mozambique AIDS Indicator Survey 2015. Fairfax, United States of America: ICF International, 2018.	GHDx
Mauritania	0	195	8195	Mauritania Multiple Indicator Cluster Survey 2007	2007	National Office of Statistics (Mauritania), United Nations Children's Fund (UNICEF). Mauritania Multiple Indicator Cluster Survey 2007. New York, United States of America: United Nations Children's Fund (UNICEF).	GHDx
Mauritania	0	13	3336	Mauritania Demographic and Health Survey 2000-2001	2000-2001	Macro International, Inc, National Office of Statistics (Mauritania). Mauritania Demographic and Health Survey 2000-2001. Fairfax, United States of America: ICF International.	GHDx
Mauritania	0	194	8880	Mauritania Multiple Indicator Cluster Survey 2011	2011	National Office of Statistics (Mauritania), United Nations Children's Fund (UNICEF). Mauritania Multiple Indicator Cluster Survey 2011. New York, United States of America: United Nations Children's Fund (UNICEF), 2015.	GHDx
Mauritania	0	13	5107	Mauritania Multiple Indicator Cluster Survey 2015	2015	National Office of Statistics (Mauritania), United Nations Children's Fund (UNICEF). Mauritania Multiple Indicator Cluster Survey 2015. New York, United States of America: United Nations Children's Fund (UNICEF), 2018.	GHDx
Mauritania	0	12	5315	Mauritania Demographic and Health Survey 2019-2020	2019-2021	National Office of Statistics (Mauritania). Mauritania Demographic and Health Survey 2019-2020. Fairfax, United States of America: ICF International, 2022.	GHDx

Country	GPS-located clusters	Areally-located clusters	Number of children included	Series	Years	Citation	Link
Malawi	0	26	20037	Malawi Multiple Indicator Cluster Survey 2006	2006	United Nations Children's Fund (UNICEF), National Statistics Office (Malawi). Malawi Multiple Indicator Cluster Survey 2006. New York, United States: United Nations Children's Fund (UNICEF).	GHDx
Malawi	559	0	10359	Malawi Demographic and Health Survey 2000	2000	Macro International, Inc, National Statistical Office of Malawi. Malawi Demographic and Health Survey 2000. Fairfax, United States of America: ICF International.	GHDx
Malawi	520	0	9834	Malawi Demographic and Health Survey 2004-2005	2004-2005	Macro International, Inc, National Statistical Office of Malawi. Malawi Demographic and Health Survey 2004-2005. Fairfax, United States of America: ICF International.	GHDx
Malawi	827	0	17903	Malawi Demographic and Health Survey 2010	2010	ICF Macro, National Statistical Office of Malawi. Malawi Demographic and Health Survey 2010. Fairfax, United States of America: ICF International.	GHDx
Malawi	0	10	1442	Malawi Global Fund Household Health Coverage Survey 2007-2008	2007-2008	Ministry of Economic Planning and Development (Malawi), National Statistical Office of Malawi. Malawi Global Fund Household Health Coverage Survey 2007-2008.	GHDx
Malawi	0	31	9487	Malawi Multiple Indicator Cluster Survey 2013-2014	2013-2014	National Statistical Office of Malawi, United Nations Children's Fund (UNICEF). Malawi Multiple Indicator Cluster Survey 2013-2014. New York, United States of America: United Nations Children's Fund (UNICEF), 2015.	GHDx
Malawi	850	0	9839	Malawi Demographic and Health Survey 2015-2016	2015-2016	Emory University and Centers for Disease Control & Prevention Collaboration, ICF International, Ministry of Health (Malawi), National Statistical Office of Malawi. Malawi Demographic and Health Survey 2015-2016. Fairfax, United States of America: ICF International, 2017.	GHDx
Malawi	1110	0	9459	Malawi Multiple Indicator Cluster Survey 2019-2020	2019-2020	National Statistical Office of Malawi, United Nations Children's Fund (UNICEF). Malawi Multiple Indicator Cluster Survey 2019-2020.	GHDx

Country	GPS-located clusters	Areally-located clusters	Number of children included	Series	Years	Citation	Link
Malaysia	0	16	9510	Malaysia National Health and Morbidity Survey 2016	2016	Institute for Public Health, Ministry of Health (Malaysia), National Institutes of Health (NIH). Malaysia National Health and Morbidity Survey 2016.	GHDx
Niger	0	8	4649	Niger Multiple Indicator Cluster Survey 2000	2000	Government of Niger, Macro International, Inc, United Nations Children's Fund (UNICEF). Niger Multiple Indicator Cluster Survey 2000. New York, United States of America: United Nations Children's Fund (UNICEF).	GHDx
Niger	0	8	6994	Niger Demographic and Health Survey 2006	2006	Department of Statistics and National Accounts (Niger), Macro International, Inc. Niger Demographic and Health Survey 2006. Fairfax, United States of America: ICF International.	GHDx
Niger	476	0	11470	Niger Demographic and Health Survey 2012	2012	ICF International, Ministry of Public Health (Niger), National Institute of Statistics (Niger). Niger Demographic and Health Survey 2012. Fairfax, United States of America: ICF International, 2019.	GHDx
Nigeria	0	37	15833	Nigeria Multiple Indicator Cluster Survey 2007	2007	United Nations Children's Fund (UNICEF), National Bureau of Statistics (Nigeria). Nigeria Multiple Indicator Cluster Survey 2007. New York, United States: United Nations Children's Fund (UNICEF).	GHDx
Nigeria	359	0	5081	Nigeria Demographic and Health Survey 2003	2003	Department for International Development (DFiD) (United Kingdom), National Population Commission of Nigeria, ORC Macro, United Nations Children's Fund (UNICEF), United Nations Population Fund (UNFPA). Nigeria Demographic and Health Survey 2003. Fairfax, United States of America: ICF International.	GHDx
Nigeria	886	0	25203	Nigeria Demographic and Health Survey 2008	2008	Macro International, Inc, National Population Commission of Nigeria. Nigeria Demographic and Health Survey 2008. Fairfax, United States of America: ICF International, 2009.	GHDx
Nigeria	0	37	8235	Nigeria Living Standards Survey 2003-2004	2003-2004	Federal Office of Statistics (Nigeria). Nigeria Living Standards Survey 2003-2004.	GHDx
Nigeria	0	37	102	Nigeria Reproductive Health, Child Health, and	2005	MEASURE Evaluation Project, Carolina Population Center, University of North Carolina, Center for Research, Evaluation, and Resource Development (CRERD), Center for Communication Programs, Bloomberg School of Public	GHDx

Country	GPS-located clusters	Areally-located clusters	Number of children included	Series	Years	Citation	Link
				Education Household, School, and Health Facility Baseline Surveys 2005		Health, Johns Hopkins, Creative Associates International, Constella Futures, Adolescent Health and Information Project (Nigeria), Federation of Muslim Womenâ€™s Associations of Nigeria (FOMWAN), Nigerian Medical Association, Management Sciences for Health (MSH), Civil Society Action Coalition on Education For All. Nigeria Reproductive Health, Child Health, and Education Household, School, and Health Facility Baseline Surveys 2005. Chapel Hill, United States: MEASURE Evaluation Project, Carolina Population Center, University of North Carolina.	
Nigeria	0	37	15906	Nigeria Multiple Indicator Cluster Survey 2011	2011	National Bureau of Statistics (Nigeria), United Nations Children's Fund (UNICEF). Nigeria Multiple Indicator Cluster Survey 2011. New York, United States of America: United Nations Children's Fund (UNICEF), 2013.	GHDx
Nigeria	889	0	28207	Nigeria Demographic and Health Survey 2013	2013	ICF International, National Population Commission of Nigeria. Nigeria Demographic and Health Survey 2013. Fairfax, United States of America: ICF International.	GHDx
Nigeria	0	769	17382	Nigeria Living Standards Survey 2008-2010	2008-2010	National Bureau of Statistics (Nigeria). Nigeria Living Standards Survey 2008-2010. Abuja, Nigeria: National Bureau of Statistics (Nigeria).	GHDx
Nigeria	1381	0	18165	Nigeria Demographic and Health Survey 2018	2018	Federal Ministry of Health (Nigeria), ICF International, National Population Commission (NPC). Nigeria Demographic and Health Survey 2018. Fairfax, United States of America: ICF International, 2020.	GHDx
Nigeria	2198	0	16304	Nigeria Multiple Indicator Cluster Survey with National Immunization Coverage Survey Supplement 2016-2017 - National Bureau of Statistics	2016-2017	National Agency for the Control of AIDS (Nigeria), National Bureau of Statistics (Nigeria), National Primary Health Care Development Agency (NPHCDA) (Nigeria), United Nations Children's Fund (UNICEF). Nigeria Multiple Indicator Cluster Survey with National Immunization Coverage Survey Supplement 2016-2017 - National Bureau of Statistics. New York, United States of America: United Nations Children's Fund (UNICEF), 2018.	GHDx

Country	GPS-located clusters	Areally-located clusters	Number of children included	Series	Years	Citation	Link
Nigeria	0	37	6630	Nigeria National Nutrition and Health Survey 2018	2018	Federal Ministry of Health (Nigeria), National Bureau of Statistics (Nigeria), National Population Commission of Nigeria. Nigeria National Nutrition and Health Survey 2018.	GHDx
Nigeria	0	37	4942	Nigeria Multiple Indicator Cluster Survey and National Immunization Coverage Survey 2021 - National Bureau of Statistics	2021	National Bureau of Statistics (Nigeria), United Nations Children's Fund (UNICEF). Nigeria Multiple Indicator Cluster Survey and National Immunization Coverage Survey 2021 - National Bureau of Statistics. New York, United States of America: United Nations Children's Fund (UNICEF).	GHDx
Philippines	805	0	6864	Philippines Demographic and Health Survey 2003	2003	Macro International, Inc, National Statistics Office (Philippines). Philippines Demographic and Health Survey 2003. Fairfax, United States of America: ICF International.	GHDx
Philippines	768	0	6336	Philippines Demographic and Health Survey 2008	2008	Macro International, Inc, National Statistics Office (Philippines). Philippines Demographic and Health Survey 2008. Fairfax, United States of America: ICF International, 2010.	GHDx
Philippines	0	17	7004	Philippines Demographic and Health Survey 2013	2013	ICF International, Philippines Statistics Authority. Philippines Demographic and Health Survey 2013. Fairfax, United States of America: ICF International, 2014.	GHDx
Papua New Guinea	0	4	1097	Papua New Guinea Demographic and Health Survey 2006-2007	2006-2007	National Statistical Office (Papua New Guinea), National Statistics Office (Philippines). Papua New Guinea Demographic and Health Survey 2006-2007.	GHDx
Papua New Guinea	722	0	5306	Papua New Guinea Demographic and Health Survey 2016-2018	2016-2018	ICF International, National Statistical Office (Papua New Guinea). Papua New Guinea Demographic and Health Survey 2016-2018. Fairfax, United States of America: ICF International, 2019.	GHDx

Country	GPS-located clusters	Areally-located clusters	Number of children included	Series	Years	Citation	Link
Rwanda	0	12	6710	Rwanda Demographic and Health Survey 2000	2000	Macro International, Inc, National Office of Population (Rwanda). Rwanda Demographic and Health Survey 2000. Fairfax, United States of America: ICF International.	GHDx
Rwanda	456	0	7620	Rwanda Demographic and Health Survey 2005	2005	Macro International, Inc, National Institute of Statistics of Rwanda. Rwanda Demographic and Health Survey 2005. Fairfax, United States of America: ICF International.	GHDx
Rwanda	246	0	4996	Rwanda Interim Demographic and Health Survey 2007-2008	2007-2008	Macro International, Inc, Ministry of Health (Rwanda), National Institute of Statistics of Rwanda. Rwanda Interim Demographic and Health Survey 2007-2008. Fairfax, United States of America: ICF International.	GHDx
Rwanda	0	12	3154	Rwanda Multiple Indicator Cluster Survey 2000	2000	Department of Statistics (Rwanda), United Nations Children's Fund (UNICEF). Rwanda Multiple Indicator Cluster Survey 2000. New York, United States: United Nations Children's Fund (UNICEF).	GHDx
Rwanda	492	0	8480	Rwanda Demographic and Health Survey 2010-2011	2010-2011	ICF Macro, Ministry of Health (Rwanda), National Institute of Statistics of Rwanda. Rwanda Demographic and Health Survey 2010-2011. Fairfax, United States of America: ICF International.	GHDx
Rwanda	0	5	8909	Rwanda Integrated Household Living Conditions Survey 2010-2011	2010-2011	National Institute of Statistics of Rwanda. Rwanda Integrated Household Living Conditions Survey 2010-2011. Kigali, Rwanda: National Institute of Statistics of Rwanda.	GHDx
Rwanda	492	0	7553	Rwanda Demographic and Health Survey 2014-2015	2014-2015	ICF International, Ministry of Health (Rwanda), National Institute of Statistics of Rwanda. Rwanda Demographic and Health Survey 2014-2015. Fairfax, United States of America: ICF International, 2016.	GHDx
Rwanda	500	0	4729	Rwanda Demographic and Health	2019-2020	Ministry of Health (Rwanda), National Institute of Statistics (Rwanda), Rwanda Biomedical Center (RBC). Rwanda Demographic and Health Survey 2019-2020. Fairfax, United States of America: ICF International, 2021.	GHDx

Country	GPS-located clusters	Areally-located clusters	Number of children included	Series	Years	Citation	Link
				Survey 2019-2020			
Senegal	366	0	9744	Senegal Demographic and Health Survey 2005	2005	Ministry of Health and Prevention (Senegal), Research Center for Human Development (Senegal). Senegal Demographic and Health Survey 2005. Fairfax, United States of America: ICF International.	GHDx
Senegal	385	0	11385	Senegal Demographic and Health Survey 2010-2011	2010-2011	Center for Research in Human Development (CRDH), Cheikh Anta Diop University, Hospital Aristide Le Dantec, ICF Macro, National Agency of Statistics and Demography (Senegal). Senegal Demographic and Health Survey 2010-2011. Fairfax, United States of America: ICF International.	GHDx
Senegal	200	0	6537	Senegal Continuous Demographic and Health Survey 2012-2013	2012-2013	ICF International, Ministry of Health and Social Action (Senegal), National Agency of Statistics and Demography (Senegal). Senegal Continuous Demographic and Health Survey 2012-2013. Fairfax, United States of America: ICF International.	GHDx
Senegal	196	0	6412	Senegal Continuous Demographic and Health Survey 2014	2014	Cheikh Anta Diop University, ICF International, National Agency of Statistics and Demography (Senegal). Senegal Continuous Demographic and Health Survey 2014. Fairfax, United States of America: ICF International.	GHDx
Senegal	214	0	6601	Senegal Continuous Demographic and Health Survey 2015	2015	Cheikh Anta Diop University, ICF International, National Agency of Statistics and Demography (Senegal). Senegal Continuous Demographic and Health Survey 2015. Fairfax, United States of America: ICF International, 2016.	GHDx
Senegal	214	0	6413	Senegal Continuous Demographic and Health Survey 2016	2016	ICF International, Ministry of Health and Social Action (Senegal), National Agency of Statistics and Demography (Senegal). Senegal Continuous Demographic and Health Survey 2016. Fairfax, United States of America: ICF International, 2017.	GHDx
Senegal	0	4	2472	Senegal - Dakar Urban Multiple Indicator Cluster Survey 2015-2016	2015-2016	National Agency of Statistics and Demography (Senegal), United Nations Children's Fund (UNICEF). Senegal - Dakar Urban Multiple Indicator Cluster Survey 2015-2016. New York, United States of America: United Nations Children's Fund (UNICEF), 2018.	GHDx
Senegal	400	0	7004	Senegal Continuous	2017	ICF International, Ministry of Health and Social Action (Senegal), National Agency of Statistics and Demography	GHDx

Country	GPS-located clusters	Areally-located clusters	Number of children included	Series	Years	Citation	Link
				Demographic and Health Survey 2017		(Senegal), Unit for the Fight Against Malnutrition (Senegal). Senegal Continuous Demographic and Health Survey 2017. Fairfax, United States of America: ICF International, 2018.	
Senegal	0	14	3618	Senegal Continuous Demographic and Health Survey 2018	2018	Directorate of Forecasting and Statistics, Ministry of the Economy, Finance and Planning (Senegal), ICF International, Ministry of Health and Social Action (Senegal), United States Agency for International Development (USAID). Senegal Continuous Demographic and Health Survey 2018. Fairfax, United States of America: ICF International, 2020.	GHDx
Senegal	213	0	2503	Senegal Continuous Demographic and Health Survey 2019	2019	ICF International, Ministry of Health and Social Action (Senegal), National Agency of Statistics and Demography (Senegal), United States Agency for International Development (USAID). Senegal Continuous Demographic and Health Survey 2019. Fairfax, United States of America: ICF International, 2020.	GHDx
Sierra Leone	0	4	2680	Sierra Leone Multiple Indicator Cluster Survey 2000	2000	Central Statistics Office (Sierra Leone), United Nations Children's Fund (UNICEF). Sierra Leone Multiple Indicator Cluster Survey 2000. New York, United States of America: United Nations Children's Fund (UNICEF).	GHDx
Sierra Leone	0	14	5038	Sierra Leone Multiple Indicator Cluster Survey 2005	2005	United Nations Children's Fund (UNICEF), Statistics Sierra Leone. Sierra Leone Multiple Indicator Cluster Survey 2005. New York, United States: United Nations Children's Fund (UNICEF).	GHDx
Sierra Leone	349	0	4892	Sierra Leone Demographic and Health Survey 2008	2008	Macro International, Inc, Statistics Sierra Leone. Sierra Leone Demographic and Health Survey 2008. Fairfax, United States of America: ICF International.	GHDx
Sierra Leone	0	14	6783	Sierra Leone Multiple Indicator Cluster Survey 2010	2010	Statistics Sierra Leone, United Nations Children's Fund (UNICEF). Sierra Leone Multiple Indicator Cluster Survey 2010. New York, United States of America: United Nations Children's Fund (UNICEF).	GHDx
Sierra Leone	435	0	10369	Sierra Leone Demographic and Health Survey 2013	2013	ICF International, Ministry of Health and Sanitation (Sierra Leone), Statistics Sierra Leone. Sierra Leone Demographic and Health Survey 2013. Fairfax, United States of America: ICF International, 2014.	GHDx
Sierra Leone	0	14	9665	Sierra Leone Multiple	2017	Statistics Sierra Leone, United Nations Children's Fund (UNICEF). Sierra Leone Multiple Indicator Cluster Survey	GHDx

Country	GPS-located clusters	Areally-located clusters	Number of children included	Series	Years	Citation	Link
				Indicator Cluster Survey 2017		2017. New York, United States of America: United Nations Children's Fund (UNICEF), 2018.	
Somalia	0	18	6103	Somalia Multiple Indicator Cluster Survey 2006	2006	Pan Arab Project for Family Health (PAPFAM), United Nations Children's Fund (UNICEF). Somalia Multiple Indicator Cluster Survey 2006. New York, United States of America: United Nations Children's Fund (UNICEF).	GHDx
Somalia	245	0	4263	Somalia - Somaliland Multiple Indicator Cluster Survey 2011	2011	Ministry of National Planning and Development (Somaliland), United Nations Children's Fund (UNICEF). Somalia - Somaliland Multiple Indicator Cluster Survey 2011. New York, United States of America: United Nations Children's Fund (UNICEF), 2015.	GHDx
Somalia	276	0	4425	Somalia - Northeast Zone Multiple Indicator Cluster Survey 2011	2011	Puntland Ministry of Planning and International Cooperation (Somalia), United Nations Children's Fund (UNICEF). Somalia - Northeast Zone Multiple Indicator Cluster Survey 2011. New York, United States of America: United Nations Children's Fund (UNICEF), 2015.	GHDx
South Sudan	0	7	1225	South Sudan Expanded Program on Immunization Coverage Survey 2017-2018	2017-2018	Ministry of Health (South Sudan), South Sudan National Bureau of Statistics. South Sudan Expanded Program on Immunization Coverage Survey 2017-2018.	GHDx
South Sudan	0	10	1403	Sudan - South Multiple Indicator Cluster Survey 2010	2010	Central Bureau of Statistics (Sudan), Federal Ministry of Health (Sudan), Government of Sudan, Ministry of Health (South Sudan), Southern Sudan Centre for Census, Statistics and Evaluation. Sudan - South Multiple Indicator Cluster Survey 2010. New York, United States of America: United Nations Children's Fund (UNICEF), 2015.	GHDx
Sao Tome and Principe	0	7	1684	Sao Tome and Principe Demographic and Health Survey 2008-2009	2008-2009	ICF Macro, Ministry of Health (Sao Tome and Principe), National Institute of Statistics (Sao Tome and Principe). Sao Tome and Principe Demographic and Health Survey 2008-2009. Fairfax, United States of America: ICF International.	GHDx

Country	GPS-located clusters	Areally-located clusters	Number of children included	Series	Years	Citation	Link
Sao Tome and Principe	0	4	912	Sao Tome and Principe Multiple Indicator Cluster Survey 2000	2000	National Institute of Statistics (Sao Tome and Principe), United Nations Children's Fund (UNICEF). Sao Tome and Principe Multiple Indicator Cluster Survey 2000. New York, United States of America: United Nations Children's Fund (UNICEF).	GHDx
Sao Tome and Principe	0	7	1034	Sao Tome and Principe Multiple Indicator Cluster Survey 2014	2014	Global Fund to Fight Aids Tuberculosis and Malaria (GFATM), ICF International, National Center for Endemic Diseases (CNE) (Sao Tome and Principe), National Institute of Statistics (Sao Tome and Principe), United Nations Children's Fund (UNICEF), United Nations Development Programme (UNDP). Sao Tome and Principe Multiple Indicator Cluster Survey 2014. New York, United States of America: United Nations Children's Fund (UNICEF), 2016.	GHDx
Sao Tome and Principe	0	5	861	Sao Tome and Principe Multiple Indicator Cluster Survey 2019	2019	National Institute of Statistics (Sao Tome and Principe), United Nations Children's Fund (UNICEF). Sao Tome and Principe Multiple Indicator Cluster Survey 2019. New York, United States of America: United Nations Children's Fund (UNICEF), 2021.	GHDx
Chad	0	15	4128	Chad Multiple Indicator Cluster Survey 2000	2000	United Nations Children's Fund (UNICEF), Census Bureau (Chad), National Institute of Statistical, Economic and Demographic Studies (Chad). Chad Multiple Indicator Cluster Survey 2000. New York, United States: United Nations Children's Fund (UNICEF).	GHDx
Chad	0	9	3652	Chad Demographic and Health Survey 2004	2004	Macro International, Inc, National Institute for Statistics, Economic and Demographic Studies (INSEED) (Chad). Chad Demographic and Health Survey 2004. Fairfax, United States of America: ICF International.	GHDx
Chad	0	60	12026	Chad Multiple Indicator Cluster Survey 2010	2010	Ministry of Planning, Economy, and International Cooperation (Chad), National Institute for Statistics, Economic and Demographic Studies (INSEED) (Chad), United Nations Children's Fund (UNICEF). Chad Multiple Indicator Cluster Survey 2010. New York, United States of America: United Nations Children's Fund (UNICEF), 2014.	GHDx
Chad	624	0	16545	Chad Demographic and Health Survey 2014-2015	2014-2015	ICF International, National Institute for Statistics, Economic and Demographic Studies (INSEED) (Chad). Chad Demographic and Health Survey 2014-2015. Fairfax, United States of America: ICF International, 2016.	GHDx

Country	GPS-located clusters	Areally-located clusters	Number of children included	Series	Years	Citation	Link
Togo	0	29	2996	Togo Multiple Indicator Cluster Survey 2000	2000	United Nations Children's Fund (UNICEF). Togo Multiple Indicator Cluster Survey 2000. New York, United States of America: United Nations Children's Fund (UNICEF).	GHDx
Togo	0	6	3410	Togo Multiple Indicator Cluster Survey 2006	2006	Directorate General of Statistics and National Accounting (Togo), United Nations Children's Fund (UNICEF). Togo Multiple Indicator Cluster Survey 2006. New York, United States: United Nations Children's Fund (UNICEF).	GHDx
Togo	0	6	3975	Togo Multiple Indicator Cluster Survey 2010	2010	Directorate General of Statistics and National Accounting (Togo), United Nations Children's Fund (UNICEF). Togo Multiple Indicator Cluster Survey 2010. New York, United States: United Nations Children's Fund (UNICEF).	GHDx
Togo	330	0	6521	Togo Demographic and Health Survey 2013-2014	2013-2014	Directorate General of Statistics and National Accounts (Togo), ICF International, Ministry of Health (Togo), Ministry of Planning, Development and Zoning (Togo). Togo Demographic and Health Survey 2013-2014. Fairfax, United States of America: ICF International, 2015.	GHDx
Togo	0	5	920	Incomplete immunization among children aged 12-23 months in Togo: a multilevel analysis of individual and contextual factors	2017	Ekouevi DK, Gbeasor-Komlanvi FA, Yaya I, Zida-Compaore WI, Boko A, Sewu E, Lacle A, Ndibu N, Toke Y, Landoh DE. Incomplete immunization among children aged 12-23Â months in Togo: a multilevel analysis of individual and contextual factors. BMC Public Health. 2018; 18(1): 952.	GHDx
Togo	409	0	2926	Togo Multiple Indicator Cluster Survey 2017	2017	Directorate General of Statistics and National Accounting (Togo), United Nations Children's Fund (UNICEF). Togo Multiple Indicator Cluster Survey 2010. New York, United States: United Nations Children's Fund (UNICEF).	GHDx
Thailand	0	4	5037	Thailand Multiple Indicator Cluster Survey 2005-2006	2005-2006	National Statistical Office (Thailand), United Nations Children's Fund (UNICEF). Thailand Multiple Indicator Cluster Survey 2005-2006. New York, United States of America: United Nations Children's Fund (UNICEF).	GHDx
Thailand	0	5	4096	Thailand Multiple Indicator	2012	College of Population Studies, Chulalongkorn University (Thailand), Institute for Population and Social Research, Mahidol University (Thailand), International Health Policy	GHDx

Country	GPS-located clusters	Areally-located clusters	Number of children included	Series	Years	Citation	Link
				Cluster Survey 2012		Program (Thailand), Ministry of Education (Thailand), Ministry of Public Health (Thailand), Ministry of Social Development and Human Security (MSDHS) (Thailand), National Health Security Office (Thailand), National Statistical Office (Thailand), Thai Health Promotion Foundation, United Nations Children's Fund (UNICEF). Thailand Multiple Indicator Cluster Survey 2012. New York, United States of America: United Nations Children's Fund (UNICEF), 2016.	
Thailand	0	5	3023	Thailand Multiple Indicator Cluster Survey 2015-2016	2015-2016	National Health Security Office (Thailand), National Statistical Office (Thailand), United Nations Children's Fund (UNICEF). Thailand Multiple Indicator Cluster Survey 2015-2016. New York, United States of America: United Nations Children's Fund (UNICEF), 2018.	GHDx
Thailand	0	1	503	Thailand - Bangkok Small Community Multiple Indicator Cluster Survey 2016	2016	National Health Security Office (Thailand), National Statistical Office (Thailand), United Nations Children's Fund (UNICEF). Thailand - Bangkok Small Community Multiple Indicator Cluster Survey 2016. New York, United States of America: United Nations Children's Fund (UNICEF), 2018.	GHDx
Thailand	0	77	8602	Thailand Multiple Indicator Cluster Survey 2019	2019	National Health Security Office (Thailand), National Statistical Office (Thailand), United Nations Children's Fund (UNICEF). Thailand Multiple Indicator Cluster Survey 2019. New York, United States of America: United Nations Children's Fund (UNICEF), 2020.	GHDx
Timor-Leste	0	163	4508	Timor-Leste Demographic and Health Survey 2003	2003	ACIL Australia Pty Ltd., Australian National University, Ministry of Health (Timor-Leste), National Statistics Directorate (Timor-Leste), University of Newcastle (Australia). Timor-Leste Demographic and Health Survey 2003. Newcastle, Australia: University of Newcastle (Australia).	GHDx
Timor-Leste	0	13	8933	Timor-Leste Demographic and Health Survey 2009-2010	2009-2010	ICF Macro, Ministry of Finance (Timor-Leste), National Statistics Directorate (Timor-Leste). Timor-Leste Demographic and Health Survey 2009-2010. Fairfax, United States of America: ICF International.	GHDx

Country	GPS-located clusters	Areally-located clusters	Number of children included	Series	Years	Citation	Link
Timor-Leste	455	0	4228	Timor-Leste Demographic and Health Survey 2016	2016	ICF International, National Statistics Directorate (Timor-Leste). Timor-Leste Demographic and Health Survey 2016. Fairfax, United States of America: ICF International, 2018.	GHDx
United Republic of Tanzania	0	26	7594	Tanzania Demographic and Health Survey 2004-2005	2004-2005	Macro International, Inc, National Bureau of Statistics (Tanzania). Tanzania Demographic and Health Survey 2004-2005. Fairfax, United States of America: ICF International.	GHDx
United Republic of Tanzania	458	0	7268	Tanzania Demographic and Health Survey 2009-2010	2009-2010	ICF Macro, National Bureau of Statistics (Tanzania). Tanzania Demographic and Health Survey 2009-2010. Fairfax, United States of America: ICF International.	GHDx
United Republic of Tanzania	0	124	2277	Tanzania National Panel Survey 2008-2009	2008-2009	National Bureau of Statistics (Tanzania). Tanzania Living Standards Measurement Study - Integrated Survey on Agriculture 2008-2009. Dar es Salaam, Tanzania: National Bureau of Statistics (Tanzania).	GHDx
United Republic of Tanzania	608	0	6090	Tanzania Demographic and Health Survey 2015-2016	2015-2016	ICF International, Ministry of Health (Zanzibar), Ministry of Health, Community Development, Gender, Elderly and Children (MoHCDEC) (Tanzania), National Bureau of Statistics (Tanzania), Office of the Chief Government Statistician (OCGS) (Zanzibar). Tanzania Demographic and Health Survey 2015-2016. Fairfax, United States of America: ICF International, 2016.	GHDx
Uganda	269	0	5605	Uganda Demographic and Health Survey 2000-2001	2000-2001	Macro International, Inc, Uganda Bureau of Statistics. Uganda Demographic and Health Survey 2000-2001. Fairfax, United States of America: ICF International.	GHDx
Uganda	336	0	6931	Uganda Demographic and Health Survey 2006	2006	Macro International, Inc, Uganda Bureau of Statistics. Uganda Demographic and Health Survey 2006. Fairfax, United States of America: ICF International.	GHDx
Uganda	400	0	7253	Uganda Demographic and Health Survey 2011	2011	ICF Macro, Uganda Bureau of Statistics. Uganda Demographic and Health Survey 2011. Fairfax, United States of America: ICF International.	GHDx
Uganda	0	80	6479	Uganda National	2008	Ministry of Public Service (Uganda), Uganda Bureau of Statistics. Uganda National Service Delivery Survey 2008.	GHDx

Country	GPS-located clusters	Areally-located clusters	Number of children included	Series	Years	Citation	Link
				Service Delivery Survey 2008			
Uganda	685	0	8769	Uganda Demographic and Health Survey 2016	2016	ICF International, Uganda Bureau of Statistics. Uganda Demographic and Health Survey 2016. Fairfax, United States of America: ICF International, 2018.	GHDx
Viet Nam	0	8	2974	Vietnam Multiple Indicator Cluster Survey 2000	2000	General Statistics Office (Vietnam), United Nations Children's Fund (UNICEF). Vietnam Multiple Indicator Cluster Survey 2000. New York, United States of America: United Nations Children's Fund (UNICEF).	GHDx
Viet Nam	0	8	2578	Vietnam Multiple Indicator Cluster Survey 2006	2006	General Statistics Office (Vietnam), United Nations Children's Fund (UNICEF). Vietnam Multiple Indicator Cluster Survey 2006. New York, United States of America: United Nations Children's Fund (UNICEF).	GHDx
Viet Nam	0	41	1235	Vietnam Demographic and Health Survey 2002	2002	General Statistics Office (Vietnam), Macro International, Inc. Vietnam Demographic and Health Survey 2002. Fairfax, United States of America: ICF International.	GHDx
Viet Nam	0	587	3603	Vietnam Multiple Indicator Cluster Survey 2010-2011	2010-2011	General Statistics Office (Vietnam), United Nations Children's Fund (UNICEF). Vietnam Multiple Indicator Cluster Survey 2010-2011. New York, United States of America: United Nations Children's Fund (UNICEF).	GHDx
Viet Nam	0	500	2072	Vietnam Multiple Indicator Cluster Survey 2013-2014	2013-2014	General Statistics Office (Vietnam), United Nations Children's Fund (UNICEF). Vietnam Multiple Indicator Cluster Survey 2013-2014. New York, United States of America: United Nations Children's Fund (UNICEF), 2015.	GHDx
Yemen	0	443	6152	Yemen Family Health Survey 2003	2003	Central Statistical Organization (Yemen), League of Arab States, Ministry of Public Health and Population (Yemen), Pan Arab Project for Family Health (PAPFAM). Yemen Family Health Survey 2003.	GHDx
Yemen	0	21	3360	Yemen Multiple Indicator Cluster Survey 2006	2006	Ministry of Health (Yemen) and United Nations Children's Fund (UNICEF). Yemen Multiple Indicator Cluster Survey 2006. New York, United States: United Nations Children's Fund (UNICEF).	GHDx

Country	GPS-located clusters	Areally-located clusters	Number of children included	Series	Years	Citation	Link
Yemen	0	489	10932	Yemen Household Budget Survey 2005-2006	2005-2006	Central Statistical Organization (Yemen). Yemen Household Budget Survey 2005-2006. Sana'a, Yemen: Central Statistical Organization (Yemen).	GHDx
Yemen	0	528	14906	Yemen Demographic and Health Survey 2013	2013	Central Statistical Organization (Yemen), ICF International, Ministry of Public Health and Population (Yemen). Yemen Demographic and Health Survey 2013. Fairfax, United States of America: ICF International.	GHDx
Zambia	0	72	5726	Zambia Living Conditions Monitoring Survey 2002-2003	2002-2003	Central Statistical Office (Zambia). Zambia Living Conditions Monitoring Survey 2002-2003. Lusaka, Zambia: Central Statistical Office (Zambia).	GHDx
Zambia	0	132	8478	Zambia Living Conditions Monitoring Survey 2004-2005	2004-2005	Central Statistical Office (Zambia). Zambia Living Conditions Monitoring Survey 2004-2005. Lusaka, Zambia: Central Statistical Office (Zambia).	GHDx
Zambia	0	72	5982	Zambia Demographic and Health Survey 2001-2002	2001-2002	Central Board of Health (Zambia), Central Statistical Office (Zambia), Macro International, Inc. Zambia Demographic and Health Survey 2001-2002. Fairfax, United States of America: ICF International.	GHDx
Zambia	319	0	5844	Zambia Demographic and Health Survey 2007	2007	Central Statistical Office (Zambia), Macro International, Inc. Zambia Demographic and Health Survey 2007. Fairfax, United States of America: ICF International.	GHDx
Zambia	0	22	1155	Zambia Global Fund Household Health Coverage Survey 2008	2008	Central Statistical Office (Zambia). Zambia Global Fund Household Health Coverage Survey 2008. Lusaka, Zambia: Central Statistical Office (Zambia).	GHDx
Zambia	0	146	2829	Zambia Living Conditions Monitoring Survey 2010	2010	Central Statistical Office (Zambia). Zambia Living Conditions Monitoring Survey 2010.	GHDx
Zambia	719	0	12654	Zambia Demographic and Health	2013-2014	Central Statistical Office (Zambia), ICF International, Ministry of Health (Zambia), Tropical Diseases Research Centre, University Teaching Hospital (Zambia), University of Zambia.	GHDx

Country	GPS-located clusters	Areally-located clusters	Number of children included	Series	Years	Citation	Link
				Survey 2013-2014		Zambia Demographic and Health Survey 2013-2014. Fairfax, United States of America: ICF International.	
Zambia	0	72	4259	Zambia Living Conditions Monitoring Survey 2015	2015	Central Statistical Office (Zambia), World Bank. Zambia Living Conditions Monitoring Survey 2015.	GHDx
Zambia	535	0	5665	Zambia Demographic and Health Survey 2018-2019	2018-2019	Central Statistical Office (Zambia), ICF International, Ministry of Health (Zambia), University Teaching Hospital (Zambia), University of Zambia. Zambia Demographic and Health Survey 2018-2019. Fairfax, United States of America: ICF International, 2020.	GHDx

Supplemental Table S3: Surveys excluded from DTP modeling.

Country	Series	Year(s)	Citation	Rationale for exclusion
Angola	Angola Core Welfare Indicators Questionnaire Survey 2011	2011	Ministry of Planning and Territorial Development (Angola), National Institute of Statistics (Angola). Angola Core Welfare Indicators Questionnaire Survey 2011. Luanda, Angola: National Institute of Statistics (Angola).	Estimates considered implausible. Survey estimates are systematically low compared to admin estimates and other sources.
Benin	Benin Demographic and Health Survey 2011-2012	2011-2012	ICF International, National Institute of Statistics and Economic Analysis (INSAE) (Benin), National Program Against AIDS (PNLS) (Benin). Benin Demographic and Health Survey 2011-2012. Fairfax, United States: ICF International, 2014.	Estimates considered implausible. Survey estimates are systematically low compared to admin estimates and estimates from other established survey series (2009-2012 Benin Health Statistical Yearbook, 2008 External Review of the Extended Programme on Immunisation)
Djibouti	Djibouti Family Health Survey 2002	2002	Department of Statistics and Demographic Studies (Djibouti), League of Arab States, Ministry of Health (Djibouti), Pan Arab Project for Family Health (PAPFAM). Djibouti Family Health Survey 2002.	Estimates considered implausible. Survey estimates are systematically high compared to admin estimates and other source for all cohorts except 12-23 and 24-35 months cohort
Ethiopia	Ethiopia Welfare Monitoring Survey 2004	2004	Central Statistical Agency (Ethiopia). Ethiopia Welfare Monitoring Survey 2004.	Estimates considered implausible. Survey estimates are systematically high compared to admin estimates and estimates from other established surveys series (2001-2003 DHS, 2001 National EPI Coverage Survey)
Ghana	Ghana Living Standards Measurement Survey 2012-2013	2012-2013	Ghana Statistical Service, World Bank. Ghana Living Standards Measurement Survey 2012-2013. Accra, Ghana: Ghana Statistical Service.	Estimates considered implausible. Survey estimates are systematically low compared to admin estimates and other sources.
Indonesia	Indonesia National Socioeconomic Survey 2006	2004-2013	Statistics Indonesia. Indonesia National Socioeconomic Survey 2006.	Estimates considered implausible. Survey estimates are inconsistent with admin estimates and estimates from other established survey series (2007-2008 Indonesia Family Life Survey, 2003-2005 DHS)
Indonesia	Indonesia Family Life Survey East 2012	2012	National Team for the Acceleration of Poverty Reduction (TNP2K) (Indonesia), SurveyMETER, University of Southern California, World Bank. Indonesia Family Life Survey East 2012.	Estimates considered implausible. Survey estimates are inconsistent with admin estimates and estimates from other established survey series (2008-2011 DHS)
Burkina Faso	Burkina Faso Core Welfare Indicators Questionnaire Survey 2007	2007	National Institute of Statistics and Demography (INSD). Burkina Faso Core Welfare Indicators Questionnaire Survey 2007. Ouagadougou, Burkina Faso: National Institute of Statistics and Demography (INSD), 2008.	Estimates considered implausible. Survey estimates are inconsistent with admin estimates and estimates from other established survey series (Burkina Faso Multiple Indicator Cluster Survey 2006, Burkina Faso Demographic and Health Survey 2003)
Burkina Faso	Burkina Faso Core Welfare Indicators Questionnaire Survey 2005	2005	National Institute of Statistics and Demography (Burkina Faso), World Bank. Burkina Faso Core Welfare Indicators Questionnaire Survey 2005. Ouagadougou, Burkina Faso: National Institute of Statistics and Demography (Burkina Faso).	Estimates considered implausible. Survey estimates are inconsistent with admin estimates and estimates from other established survey series (Burkina Faso Multiple Indicator Cluster Survey 2006, Burkina Faso Demographic and Health Survey 2003)
Cote d'Ivoire	Cote d'Ivoire Multiple Indicator Cluster Survey 2016	2016	National Institute of Statistics (Côte d'Ivoire), United Nations Children's Fund (UNICEF). Cote d'Ivoire Multiple Indicator Cluster Survey 2016. New York, United States of America: United Nations Children's Fund (UNICEF), 2018.	Estimates outliered due to implausibly high coverage values in some data points (100%)
Cameroon	Cameroon Multiple	2014	Ministry of Public Health (Cameroon), National Institute of Statistics (Cameroon), United Nations Children's Fund	Coverage is implausibly high for the 48-59 months cohort compared to estimates for other cohorts of this MICS

Country	Series	Year(s)	Citation	Rationale for exclusion
	Indicator Cluster Survey 2014		(UNICEF). Cameroon Multiple Indicator Cluster Survey 2014. New York, United States of America: United Nations Children's Fund (UNICEF), 2017.	
Congo	Congo Demographic and Health Survey 2005	2005	Macro International, Inc, National Center for Statistics and Economic Studies (Congo, Rep.). Congo Demographic and Health Survey 2005. Fairfax, United States of America: ICF International.	Coverage patterns suggest strong influence of catch-up vaccination rather than on-schedule routine immunization
Ghana	Ghana Socioeconomic Panel Survey 2009-2010	2009-2010	Economic Growth Center, Yale University, Institute of Statistical, Social and Economic Research, University of Ghana. Ghana Socioeconomic Panel Survey 2009-2010. Washington DC, United States: World Bank.	Estimates outliered due to implausible coverage, including values of both 100% and 0% coverage in 2000
Guinea-Bissau	Guinea-Bissau Multiple Indicator Cluster Survey 2000	2000	Secretary State of Planning, National Institute of Statistics and Census (INEC), United Nations Children's Fund (UNICEF). Guinea-Bissau Multiple Indicator Cluster Survey 2000. New York, United States: United Nations Children's Fund (UNICEF).	Estimates outliered due to a large observed cohort effect (24-25 months cohort).
Indonesia	Indonesia National Socioeconomic Survey 2005	2005	Statistics Indonesia. Indonesia National Socioeconomic Survey 2005.	Estimates considered implausible. Survey estimates are inconsistent with admin estimates and estimates from other established survey series (2007-2008 Indonesia Family Life Survey, 2003-2005 DHS)
Indonesia	Indonesia National Socioeconomic Survey 2004	2004	Statistics Indonesia. Indonesia National Socioeconomic Survey 2004.	Estimates considered implausible. Survey estimates are inconsistent with admin estimates and estimates from other established survey series (2007-2008 Indonesia Family Life Survey, 2003-2005 DHS)
Indonesia	Indonesia National Socioeconomic Survey 2007	2007	Statistics Indonesia. Indonesia National Socioeconomic Survey 2007.	Estimates considered implausible. Survey estimates are inconsistent with admin estimates and estimates from other established survey series (2007-2008 Indonesia Family Life Survey, 2003-2005 DHS)
Indonesia	Indonesia National Socioeconomic Survey 2010	2010	Statistics Indonesia. Indonesia National Socioeconomic Survey 2010.	Estimates considered implausible. Survey estimates are inconsistent with admin estimates and estimates from other established survey series (2007-2008 Indonesia Family Life Survey, 2003-2005 DHS)
Indonesia	Indonesia National Socioeconomic Survey 2008	2008	Statistics Indonesia. Indonesia National Socioeconomic Survey 2008.	Estimates considered implausible. Survey estimates are inconsistent with admin estimates and estimates from other established survey series (2007-2008 Indonesia Family Life Survey, 2003-2005 DHS)
Indonesia	Indonesia National Socioeconomic Survey 2009	2009	Statistics Indonesia. Indonesia National Socioeconomic Survey 2009.	Estimates considered implausible. Survey estimates are inconsistent with admin estimates and estimates from other established survey series (2007-2008 Indonesia Family Life Survey, 2003-2005 DHS)
Indonesia	Indonesia National Socioeconomic Survey 2011	2011	Statistics Indonesia. Indonesia National Socioeconomic Survey 2011.	Estimates considered implausible. Survey estimates are inconsistent with admin estimates and estimates from other established survey series (2007-2008 Indonesia Family Life Survey, 2003-2005 DHS)
Indonesia	Indonesia National Socioeconomic Survey 2012	2012	Statistics Indonesia. Indonesia National Socioeconomic Survey 2012.	Estimates considered implausible. Survey estimates are inconsistent with admin estimates and estimates from other established survey series (2007-2008 Indonesia Family Life Survey, 2003-2005 DHS)

Country	Series	Year(s)	Citation	Rationale for exclusion
Indonesia	Indonesia National Socioeconomic Survey 2013	2013	Statistics Indonesia. Indonesia National Socioeconomic Survey 2013.	Estimates considered implausible. Survey estimates are inconsistent with admin estimates and estimates from other established survey series (2007-2008 Indonesia Family Life Survey, 2003-2005 DHS)
Indonesia	Indonesia Basic Health Research 2018	2018	Agency of Health Research and Development (Indonesia). Indonesia Basic Health Research 2018.	Estimates considered implausible. Survey estimates are inconsistent with admin estimates and estimates from other established surveys (2017 DHS)
Kenya	Kenya Demographic and Health Survey 2003	2003	Centers for Disease Control and Prevention (CDC), Central Bureau of Statistics (Kenya), Macro International, Inc, Ministry of Health (Kenya), National Council for Population and Development (Kenya). Kenya Demographic and Health Survey 2003. Fairfax, United States of America: ICF International.	Estimates considered implausible. Survey estimates are systematically low compared to admin estimates and estimates from other established survey series (2005-2006 Kenya Integrated Household Budget Survey, 2004-2006 DHS)
Kenya	Kenya - North Eastern Province Multiple Indicator Cluster Survey 2007	2007	Kenya National Bureau of Statistics, United Nations Children's Fund (UNICEF). Kenya - North Eastern Province Multiple Indicator Cluster Survey 2007. Nairobi, Kenya: Kenya National Bureau of Statistics.	Estimates considered implausible. Survey estimates are systematically low compared to admin estimates and estimates from other established survey series (2005-2006 Kenya Integrated Household Budget Survey, 2004-2006 DHS)
Cambodia	Cambodia Socio-Economic Survey 2009	2009	National Institute of Statistics (Cambodia), Statistics Sweden. Cambodia Socio-Economic Survey 2009. Phnom Penh, Cambodia: National Institute of Statistics (Cambodia).	Estimates considered implausible. Survey estimates are inconsistent with admin estimates and estimates from other established survey series (Cambodia Demographic and Health Survey 2005-2006, Cambodia Demographic and Health Survey 2010-2011)
Madagascar	Madagascar Multiple Indicator Cluster Survey 2000	2000	National Institute of Statistics (Madagascar), United Nations Children's Fund (UNICEF). Madagascar Multiple Indicator Cluster Survey 2000. New York, United States of America: United Nations Children's Fund (UNICEF).	Estimates outliered due to implausibly low coverage
Mali	Mali Multiple Indicator Cluster Survey 2009-2010	2009-2010	Ministry of Health (Mali), National Institute of Statistics (INSTAT) (Mali), United Nations Children's Fund (UNICEF). Mali Multiple Indicator Cluster Survey 2009-2010. New York, United States of America: United Nations Children's Fund (UNICEF), 2017.	Estimates considered implausible. Survey estimates are inconsistent with both admin estimates and estimates from other established survey series (2008-2011 DHS, 2010-2013 World Bank Living Standards Measurement Survey)
Niger	Niger National Survey on Household Living Conditions and Agriculture 2014-2015	2014-2015	National Institute of Statistics (Niger), World Bank. Niger National Survey on Household Living Conditions and Agriculture 2014-2015. Washington DC, United States of America: World Bank.	Estimates outliered due to reliability concerns, survey design, and implausibly low coverage
Nigeria	Nigeria General Household Survey 2007	2007	Central Bank of Nigeria, National Bureau of Statistics (Nigeria), Nigerian Communications Commission (NCC). Nigeria General Household Survey 2007. Abuja, Nigeria: National Bureau of Statistics (Nigeria).	Estimates considered implausible. Survey estimates are systematically low compared to admin estimates and estimates from other established survey series (1999-2007 DHS, 20072010 MICS)
Nigeria	Nigeria Reproductive Health, Child Health, and Education Household,	2007	MEASURE Evaluation Project, Carolina Population Center, University of North Carolina, Center for Research, Evaluation, and Resource Development (CRERD), Center for Communication Programs, Bloomberg School of Public Health, Johns Hopkins, Creative Associates International, Constella Futures, Adolescent Health and Information Project (Nigeria), Federation of Muslim	Estimates considered implausible. Survey estimates are inconsistent with both admin estimates and estimates from other established survey series (1999-2007 DHS, 2007-2010 MICS)

Country	Series	Year(s)	Citation	Rationale for exclusion
	School, and Health Facility Midline Surveys 2007		WomenÃ¢Â€Â™s Associations of Nigeria (FOMWAN), Nigerian Medical Association, Management Sciences for Health (MSH), Civil Society Action Coalition on Education For All. Nigeria Reproductive Health, Child Health, and Education Household, School, and Health Facility Midline Surveys 2007. Chapel Hill, United States: MEASURE Evaluation Project, Carolina Population Center, University of North Carolina.	
Nigeria	Nigeria General Household Survey, Panel 2012-2013, Wave 2	2012-2013	National Bureau of Statistics (Nigeria). Nigeria General Household Survey, Panel 2012-2013, Wave 2. Washington DC, United States of America: World Bank.	Estimates considered implausible. Survey estimates are systematically high compared to admin estimates and estimates from other established survey series (2009 DHS, 2009 MICS, 2009 Nigeria Reproductive Health, Child Health, and Education Household, School, and Health Facility Survey, 2009-2012 Nigeria Annual Abstract of Statistics)
Nigeria	Nigeria General Household Survey 2010-2011	2010-2011	National Bureau of Statistics (Nigeria). Nigeria General Household Survey 2010-2011. Abuja, Nigeria: National Bureau of Statistics (Nigeria).	Estimates considered implausible. Survey estimates are systematically high compared to admin estimates and estimates from other established survey series (2009 DHS, 2009 MICS, 2009 Nigeria Reproductive Health, Child Health, and Education Household, School, and Health Facility Survey, 2009-2012 Nigeria Annual Abstract of Statistics)
Philippines	Philippines Demographic and Health Survey 2017	2017	ICF International, Philippines Statistics Authority, United States Agency for International Development (USAID). Philippines Demographic and Health Survey 2017. Fairfax, United States of America: ICF International, 2018.	Estimates considered implausible. Some survey estimates are systematically low compared those in other locations in the same survey.
Senegal	Senegal Continuous Demographic and Health Survey 2019	2019	ICF International, Ministry of Health and Social Action (Senegal), National Agency of Statistics and Demography (Senegal), United States Agency for International Development (USAID). Senegal Continuous Demographic and Health Survey 2019. Fairfax, United States of America: ICF International, 2020.	Coverage patterns in data suggest strong influence of catch-up vaccination after 2018 health workers strike rather than on-schedule routine immunization
Sao Tome and Principe	Sao Tome and Principe Multiple Indicator Cluster Survey 2000	2000	National Institute of Statistics (Sao Tome and Principe), United Nations Children's Fund (UNICEF). Sao Tome and Principe Multiple Indicator Cluster Survey 2000. New York, United States of America: United Nations Children's Fund (UNICEF).	Coverage patterns in data suggest strong influence of catch-up vaccination rather than routine immunization
Uganda	Uganda National Service Delivery Survey 2004	2004	Ministry of Public Service (Uganda), Uganda Bureau of Statistics. Uganda National Service Delivery Survey 2004. OpenMicroData.	Estimates considered implausible. Survey estimates are systematically high compared to estimates from other established surveys over the same period (Uganda Demographic and Health Survey 2000-2001, Uganda Demographic and Health Survey 2011). Also, some households were not covered due to insecurity in the districts of Gulu, Lira, Katakwi, Kitgum, and Pader; while in Karamoja region (Kotido, Nakapiripirit and Moroto) due to mobility of the pastoralist communities.
Uganda	Uganda Gavi FCE Household Survey 2015	2015	Institute for Health Metrics and Evaluation (IHME), Infectious Diseases Research Collaboration (IDRC). Uganda Gavi FCE Household Survey 2015. Seattle, United States: Institute for Health Metrics and Evaluation (IHME), 2017.	Estimates considered implausible. Survey estimates are inconsistent with both admin estimates and estimates from other established survey series (2016 DHS)
Yemen	Yemen - Aden Nutritional Status and	2015	Ministry of Public Health and Population (Yemen), Yemen UNICEF, Field Medical Foundation. Yemen - Aden Nutrition and Mortality Survey 2015.	Estimates considered implausible. Survey estimates include implausibly high coverage rates of >100%

Country	Series	Year(s)	Citation	Rationale for exclusion
	Mortality Survey 2015			
Yemen	Yemen - Ibb Nutritional Status and Mortality Survey 2012	2012	Ministry of Public Health and Population (Yemen), Yemen UNICEF. Yemen - Ibb Nutrition and Mortality Survey 2012.	Estimates considered implausible. Survey estimates include implausibly high coverage rates of >100%
Yemen	Yemen - Mahweet Nutritional Status and Mortality Survey 2013	2013	Ministry of Public Health and Population (Yemen), Yemen UNICEF. Yemen - Mahweet Nutrition and Mortality Survey 2013.	Estimates considered implausible. Survey estimates include implausibly high coverage rates of >100%
Zambia	Zambia Global Fund Household Health Coverage Survey 2008	2008	Central Statistical Office (Zambia). Zambia Global Fund Household Health Coverage Survey 2008. Lusaka, Zambia: Central Statistical Office (Zambia).	Estimates considered implausible for 24-35 and 36-47 cohort. Survey estimates are systematically low compared to admin estimates and estimates from other established survey series (DHS 2007, LCMS 2010)

Supplemental Table S4: GATHER Compliance checklist

Item number	Checklist item	Reported on page number(s):
**Objectives and funding**		
1	Define the indicator(s), populations (including age, sex, and geographic entities), and time period(s) for which estimates were made.	Main text, Table 1
2	List the funding sources for the work.	Main text, Conflict of Interest section, paragraph 1; Funding section, paragraph 1
**Data inputs**		
For all data inputs from multiple sources that are synthesised as part of the study:		
3	Describe how the data were identified and how the data were accessed.	Main text, Geospatial estimation of vaccine coverage section, paragraphs 3-4; Spatial estimates of other health indicators section, paragraphs 1-2; Main text, Table 1
4	Specify the inclusion and exclusion criteria. Identify all ad hoc exclusions.	Main text, Materials and Methods section, paragraph 2-3, 5; Supplemental tables S2 and S3
5	Provide information on all included data sources and their main characteristics. For each data source used, report reference information or contact name/institution, population represented, data collection method, year(s) of data collection, sex and age range, diagnostic criteria or measurement method, and sample size, as relevant.	Supplemental Table S2
6	Identify and describe any categories of input data that have potentially important biases (e.g., based on characteristics listed in item 5).	Main text, Discussion section, paragraph 4
For data inputs that contribute to the analysis but were not synthesised as part of the study:		
7	Describe and give sources for any other data inputs.	N/A
For all data inputs:		
8	Provide all data inputs in a file format from which data can be efficiently extracted (e.g., a spreadsheet rather than a PDF), including all relevant meta-data listed in item 5. For any data inputs that cannot be shared because of ethical or	Available through: https://ghdx.healthdata.org/record/ihme-data/dtp-vaccines-zerodose-overlap

	legal reasons, such as third-party ownership, provide a contact name or the name of the institution that retains the right to the data.	
**Data analysis**		
9	Provide a conceptual overview of the data analysis method. A diagram might be helpful.	Main text, Materials and methods section
10	Provide a detailed description of all steps of the analysis, including mathematical formulae. This description should cover, as relevant, data cleaning, data pre-processing, data adjustments and weighting of data sources, and mathematical or statistical model(s).	Main text, Analyses of spatial overlap section
11	Describe how candidate models were evaluated and how the final model(s) were selected.	N/A
12	Provide the results of an evaluation of model performance, if done, as well as the results of any relevant sensitivity analysis.	N/A
13	Describe methods for calculating uncertainty of the estimates. State which sources of uncertainty were, and were not, accounted for in the uncertainty analysis.	Main text, Materials and methods section, paragraph 7; Discussion section, paragraph 4
14	State how analytic or statistical source used to generate estimates can be accessed.	Available through: https://www.github.com/ihmeuw/vaccines-zerodose-overlap
**Results and discussion**		
15	Provide published estimates in a file format from which data can be efficiently extracted.	Available through: https://ghdx.healthdata.org/record/ihme-data/dtp-vaccines-zerodose-overlap
16	Report a quantitative measure of uncertainty of the estimates (e.g., uncertainty intervals).	Main text, Materials and methods section, paragraph 7; Discussion section, paragraph 4
17	Interpret results in light of existing evidence. If updating a previous set of estimates, describe the reasons for changes in estimates.	Main text, Discussion section, paragraphs 3-5
18	Discuss limitations that affect interpretation of the estimates.	Main text, Discussion section, paragraph 4

Supplemental Table S5: Geospatial covariates used in modeling.

Covariate	Temporal resolution	Source	Reference
**Travel time to nearest settlement**	Static	Oxford	Weiss, D. J., et al. A global map of travel time to cities to assess inequalities in accessibility in 2015. Nature 533, 333-336 (2018).
**Distance from rivers or lakes**	Static	Natural Earth Data (derived)	Available at: http://www.naturalearthdata.com/downloads/10m-physical-vectors/10m-rivers-lake-centerlines/ AND http://www.worldwildlife.org/pages/global-lakes-and-wetlands-database
**Elevation**	Static	NOAA GLOBE	Available at: https://www.ngdc.noaa.gov/mgg/topo/gltiles.html
**Population**	Annual	WorldPop	Lloyd, C. T., et al. Global spatio-temporally harmonised datasets for producing high-resolution gridded population distribution datasets. Big Earth Data. 2096(4471): 2574-5417 (2019).<br><br>World Pop. Get data. Available at: http://www.worldpop.org.uk/data/get_data/. (Accessed: 22th January 2019)
**Urban or rural**	Annual	European Commission / GHS	Pesaresi, M. et al. Operating procedure for the production of the Global Human Settlement Layer from Landsat data of the epochs 1975, 1990, 2000, and 2014. (Publications Office of the European Union, 2016). Available at: http://ghsl.jrc.ec.europa.eu/data.php
**Irrigation**	Static	University of Frankfurt and FAO	Siebert, S., Doll, P., Hoogeveen, J., Faures, J.-M., Frenken, K., & Feick, S. Development and validation of the global map of irrigation areas. Hydrology and Earth System Sciences 9, 535-547 (2005).<br><br>Goethe-Universität. Generation of a digital global map of irrigation areas. Available at: https://www.unifrankfurt.de/45218039/Global_Irrigation_Map. (Accessed: 25th July 2017). Also from: http://www.fao.org/nr/water/aquastat/irrigationmap/index10.stm
**Cloud cover percentage**	Annual	Climatic Research Unit	Harris, I., Osborn, T.J., Jones, P. et al. Version 4 of the CRU TS monthly high-resolution gridded multivariate climate dataset. Sci Data 7, 109 (2020). https://doi.org/10.1038/s41597-020-0453-3<br><br>High-resolution gridded datasets (and derived products). Available at: https://crudata.uea.ac.uk/cru/data/hrg/.
**Mean diurnal temperature range**	Annual	Climatic Research Unit	Harris, I., Osborn, T.J., Jones, P. et al. Version 4 of the CRU TS monthly high-resolution gridded multivariate climate dataset. Sci Data 7, 109 (2020). https://doi.org/10.1038/s41597-020-0453-3<br><br>High-resolution gridded datasets (and derived products). Available at: https://crudata.uea.ac.uk/cru/data/hrg/.
**Frost day frequency**	Annual	Climatic Research Unit	Harris, I., Osborn, T.J., Jones, P. et al. Version 4 of the CRU TS monthly high-resolution gridded multivariate climate dataset. Sci Data 7, 109 (2020). https://doi.org/10.1038/s41597-020-0453-3<br><br>High-resolution gridded datasets (and derived products). Available at: https://crudata.uea.ac.uk/cru/data/hrg/.
**Mean potential evapotranspiration per day per month**	Annual	Climatic Research Unit	Harris, I., Osborn, T.J., Jones, P. et al. Version 4 of the CRU TS monthly high-resolution gridded multivariate climate dataset. Sci Data 7, 109 (2020). https://doi.org/10.1038/s41597-020-0453-3

Covariate	Temporal resolution	Source	Reference
			High-resolution gridded datasets (and derived products). Available at: https://crudata.uea.ac.uk/cru/data/hrg/.
**Average daily minimum temperature**	Annual	Climatic Research Unit	Harris, I., Osborn, T.J., Jones, P. et al. Version 4 of the CRU TS monthly high-resolution gridded multivariate climate dataset. Sci Data 7, 109 (2020). https://doi.org/10.1038/s41597-020-0453-3<br><br>High-resolution gridded datasets (and derived products). Available at: https://crudata.uea.ac.uk/cru/data/hrg/.
**Average daily mean temperature**	Annual	Climatic Research Unit	Harris, I., Osborn, T.J., Jones, P. et al. Version 4 of the CRU TS monthly high-resolution gridded multivariate climate dataset. Sci Data 7, 109 (2020). https://doi.org/10.1038/s41597-020-0453-3<br><br>High-resolution gridded datasets (and derived products). Available at: https://crudata.uea.ac.uk/cru/data/hrg/.
**Average daily maximum temperature**	Annual	Climatic Research Unit	Harris, I., Osborn, T.J., Jones, P. et al. Version 4 of the CRU TS monthly high-resolution gridded multivariate climate dataset. Sci Data 7, 109 (2020). https://doi.org/10.1038/s41597-020-0453-3<br><br>High-resolution gridded datasets (and derived products). Available at: https://crudata.uea.ac.uk/cru/data/hrg/.
**Mean vapour pressure**	Annual	Climatic Research Unit	Harris, I., Osborn, T.J., Jones, P. et al. Version 4 of the CRU TS monthly high-resolution gridded multivariate climate dataset. Sci Data 7, 109 (2020). https://doi.org/10.1038/s41597-020-0453-3<br><br>High-resolution gridded datasets (and derived products). Available at: https://crudata.uea.ac.uk/cru/data/hrg/.
**Monthly wet day frequency**	Annual	Climatic Research Unit	Harris, I., Osborn, T.J., Jones, P. et al. Version 4 of the CRU TS monthly high-resolution gridded multivariate climate dataset. Sci Data 7, 109 (2020). https://doi.org/10.1038/s41597-020-0453-3<br><br>High-resolution gridded datasets (and derived products). Available at: https://crudata.uea.ac.uk/cru/data/hrg/.
**Enhanced vegetation index**	Annual	Moderate Resolution Imaging Spectroradiometer (MODIS)	Weiss, D. J. et al. An effective approach for gapfilling continental scale remotely sensed timeseries. Isprs J. Photogramm. Remote Sens. 98, 106–118 (2014).<br><br>USGS & NASA. Vegetation indices 16-Day L3 global 500m MOD13A1 dataset. Available at: https://lpdaac.usgs.gov/dataset_discovery/modis/m odis_products_table/mcd43b4. (Accessed: 25th July 2017) Huete, A., Justice, C. & van Leeuwen, W. MODIS vegetation index (MOD 13) algorithm theoretical basis document. (1999).
**Growing season length**	Static	FAO Global Agro-Ecological Zone (GAEZ) project	Available at: http://www.fao.org/nr/gaez/about-data-portal/en/
**Normalised difference vegetation index**	Annual	MODIS	NASA & NOAA. Advanced Very High Resolution Radiometer (AVHRR) Normalized Difference Vegetation Index (NDVI) dataset. Available at: https://nex.nasa.gov/nex/projects/1349/. (Accessed: 25th July 2017)

Covariate	Temporal resolution	Source	Reference
**Multi-source Weighted-Ensemble Precipitation**	Annual	Princeton University	Beck, H.E., A.I.J.M. van Dijk, V. Levizzani, J. Schellekens, D.G. Miralles, B. Martens, A. de Roo: MSWEP: 3-hourly 0.25 global gridded precipitation (1979-2015) by merging gauge, satellite, and reanalysis data, Hydrology and Earth System Sciences, 21(1), 589-615, 2017.<br><br>Available at: https://data.princetonclimate.com/opendap.
**Tasseled cap brightness**	Annual	MODIS	Lobser, S.E., Cohen, W.B. MODIS tasseled cap: land cover characteristics expressed through transformed MODIS data. International Journal of Remote Sensing. 28(22): 5079-5101 (2007).<br><br>Available at: https://modis.gsfc.nasa.gov/data/dataprod/mod43.php
**Tasseled cap wetness**	Annual	MODIS	Lobser, S.E., Cohen, W.B. MODIS tasseled cap: land cover characteristics expressed through transformed MODIS data. International Journal of Remote Sensing. 28(22): 5079-5101 (2007).<br><br>Available at: https://modis.gsfc.nasa.gov/data/dataprod/mod43.php.
**Average land surface temperature**	Annual	MODIS	Weiss, D. J. et al. An effective approach for gapfilling continental scale remotely sensed timeseries. Isprs J. Photogramm. Remote Sens. 98, 106–118 (2014).<br><br>Available at https://modis.gsfc.nasa.gov/data/dataprod/mod11.php
**Daytime land surface temperature**	Annual	MODIS	Weiss, D. J. et al. An effective approach for gapfilling continental scale remotely sensed timeseries. Isprs J. Photogramm. Remote Sens. 98, 106–118 (2014).<br><br>Available at https://modis.gsfc.nasa.gov/data/dataprod/mod11.php
**Difference between daytime and nighttime land surface temperature**	Annual	MODIS	Weiss, D. J. et al. An effective approach for gapfilling continental scale remotely sensed timeseries. Isprs J. Photogramm. Remote Sens. 98, 106–118 (2014).<br><br>Available at https://modis.gsfc.nasa.gov/data/dataprod/mod11.php
**Nighttime land surface temperature**	Annual	MODIS	Weiss, D. J. et al. An effective approach for gapfilling continental scale remotely sensed timeseries. Isprs J. Photogramm. Remote Sens. 98, 106–118 (2014).<br><br>Available at https://modis.gsfc.nasa.gov/data/dataprod/mod11.php
**Mean years of education among 15–49 year-old females (maternal education)**	Annual	IHME	Graetz, N., Woyczynski, L., Wilson, K.F. et al. Mapping disparities in education across low- and middle-income countries. Nature 577, 235–238 (2020).<br><br>Available at: http://internal-ghdx.healthdata.org/record/ihme-data/lmic-education-geospatial-estimates-2000-2017\

Supplemental Figures

Supplemental Figures S1–S23: Country-specific AUC step plots.

For each country (Angola, DRC, Ethiopia, Indonesia and Nigeria) and health indicator (children with stunting, U5M, missed ORT, missed ITNs and LF), these scatterplots visualize area under the curve (AUC) can be used to quantify the proportion of the target population for the given indicator (Y axis) that could be reached through cumulative proportion targeting of districts for children with no-DTP (X axis). Individual points represent districts, ordered to begin with the district with the highest number of no-DTP children, then the second highest, until the cumulative proportion of no-DTP children reaches 100%. The red line represents AUC=0.5, indicating equal proportions for the given health indicator reached through cumulative no-DTP targeting. Curves below the red line are associated with AUC<0.5 or smaller proportions for the given indicator reached, and curves above the red line are associated with AUC>0.5, or greater proportions for the given indicator reached. Point size represents district population size of children under 1.

Supplemental Figure S1: Nigeria, U5M

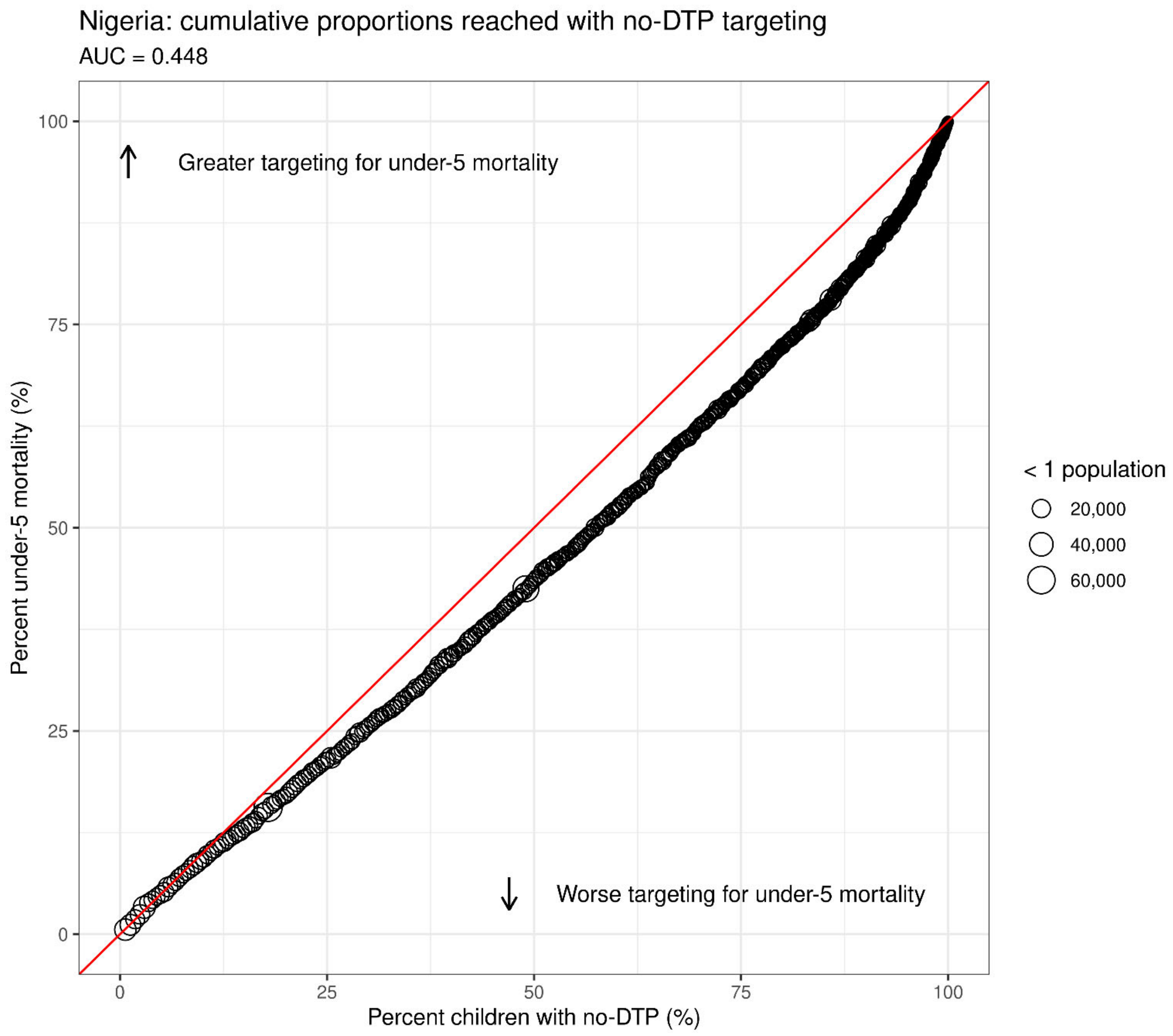

Supplemental Figure S2: Nigeria, missed ITNs

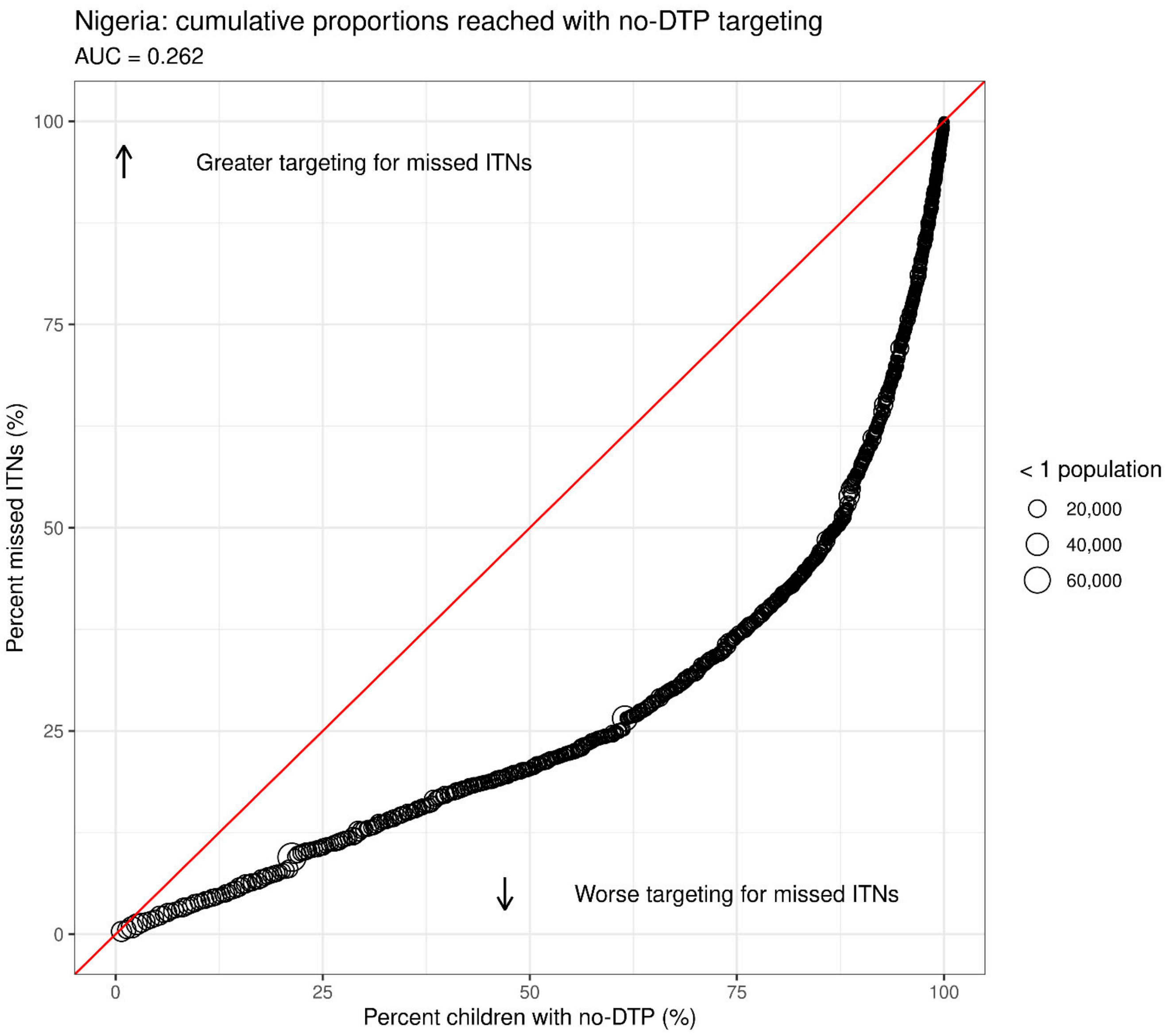

Supplemental Figure S3: Nigeria, missed ORT

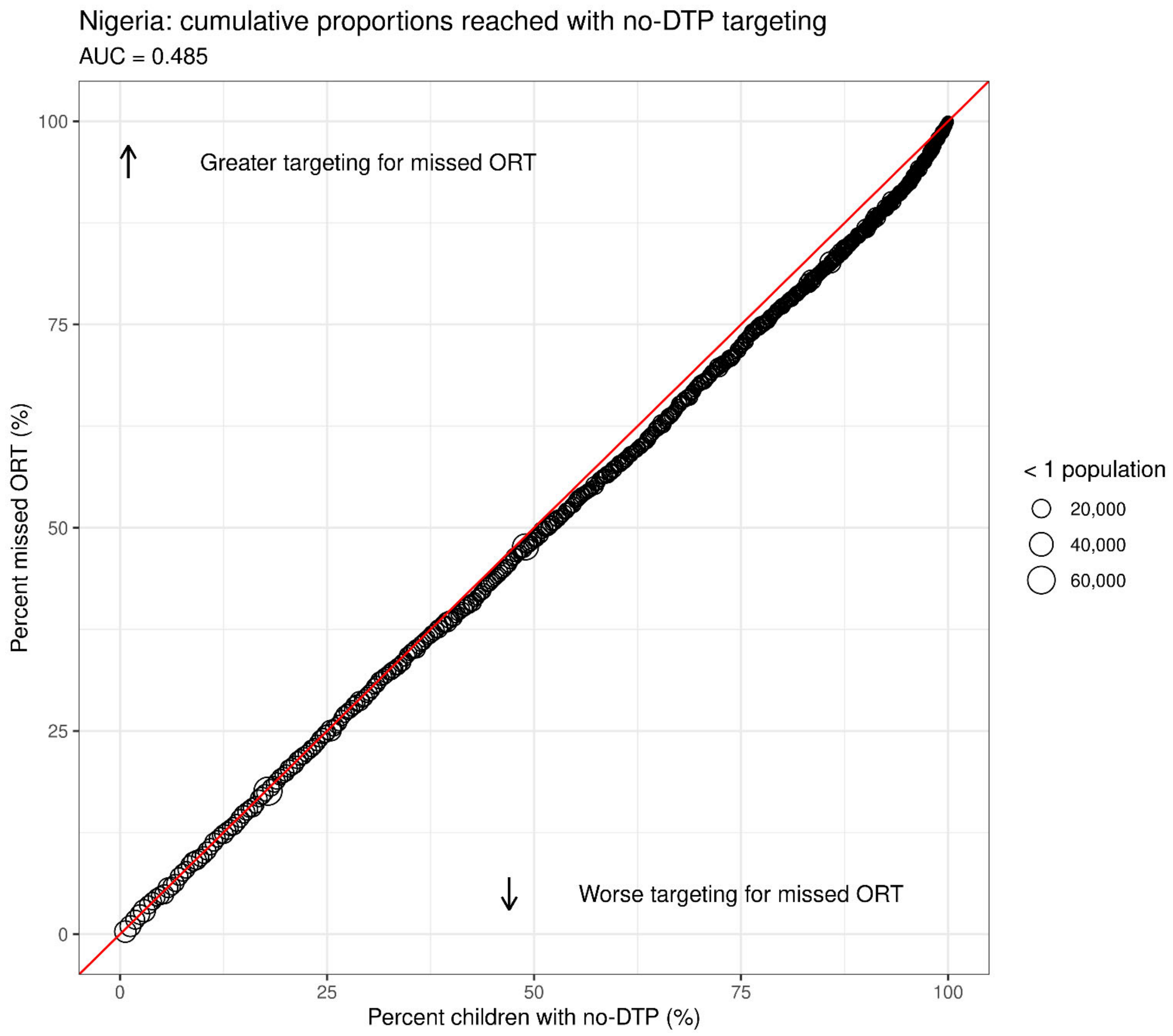

Supplemental Figure S4: Nigeria, LF

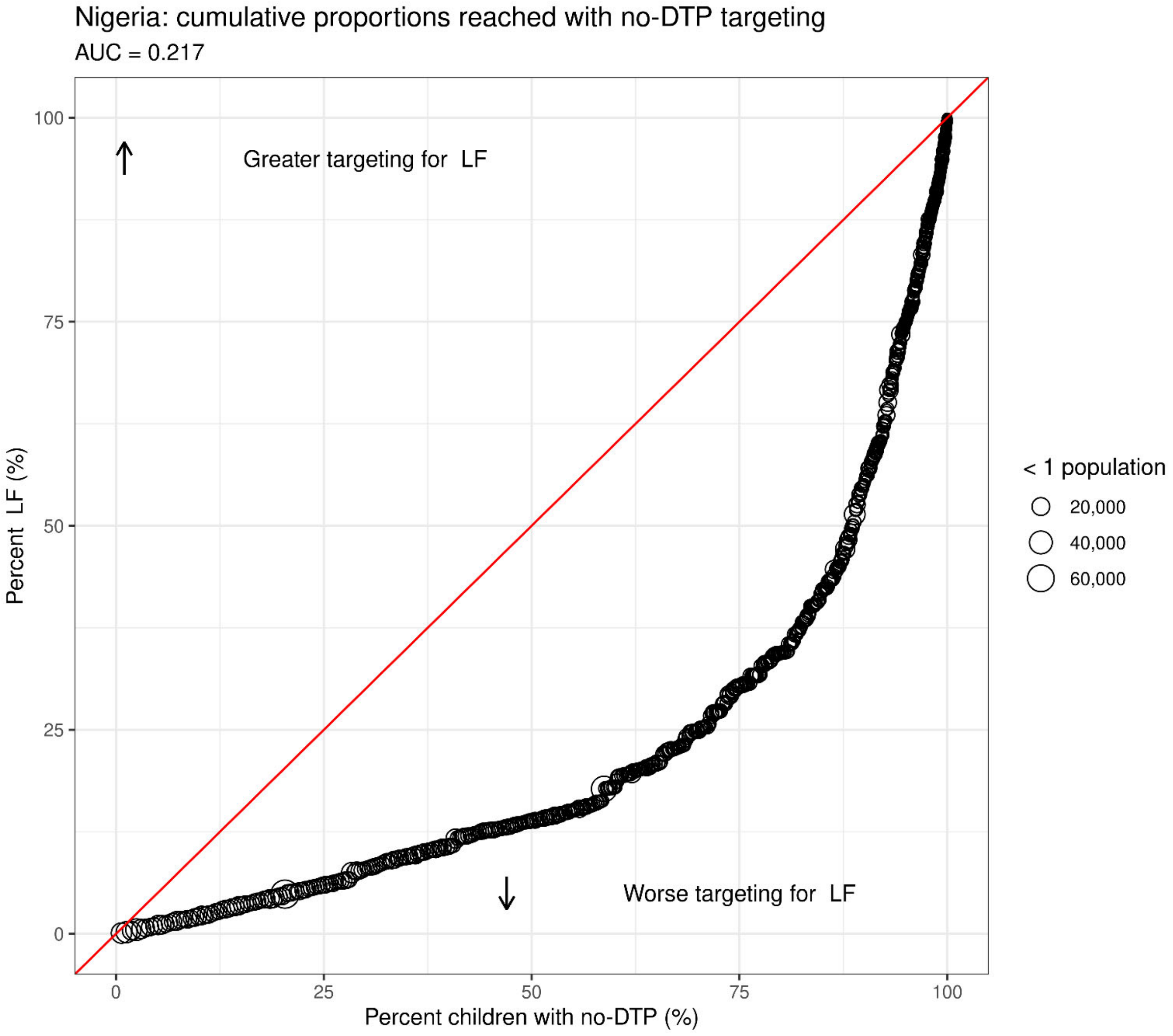

Supplemental Figure S5: Angola, stunting

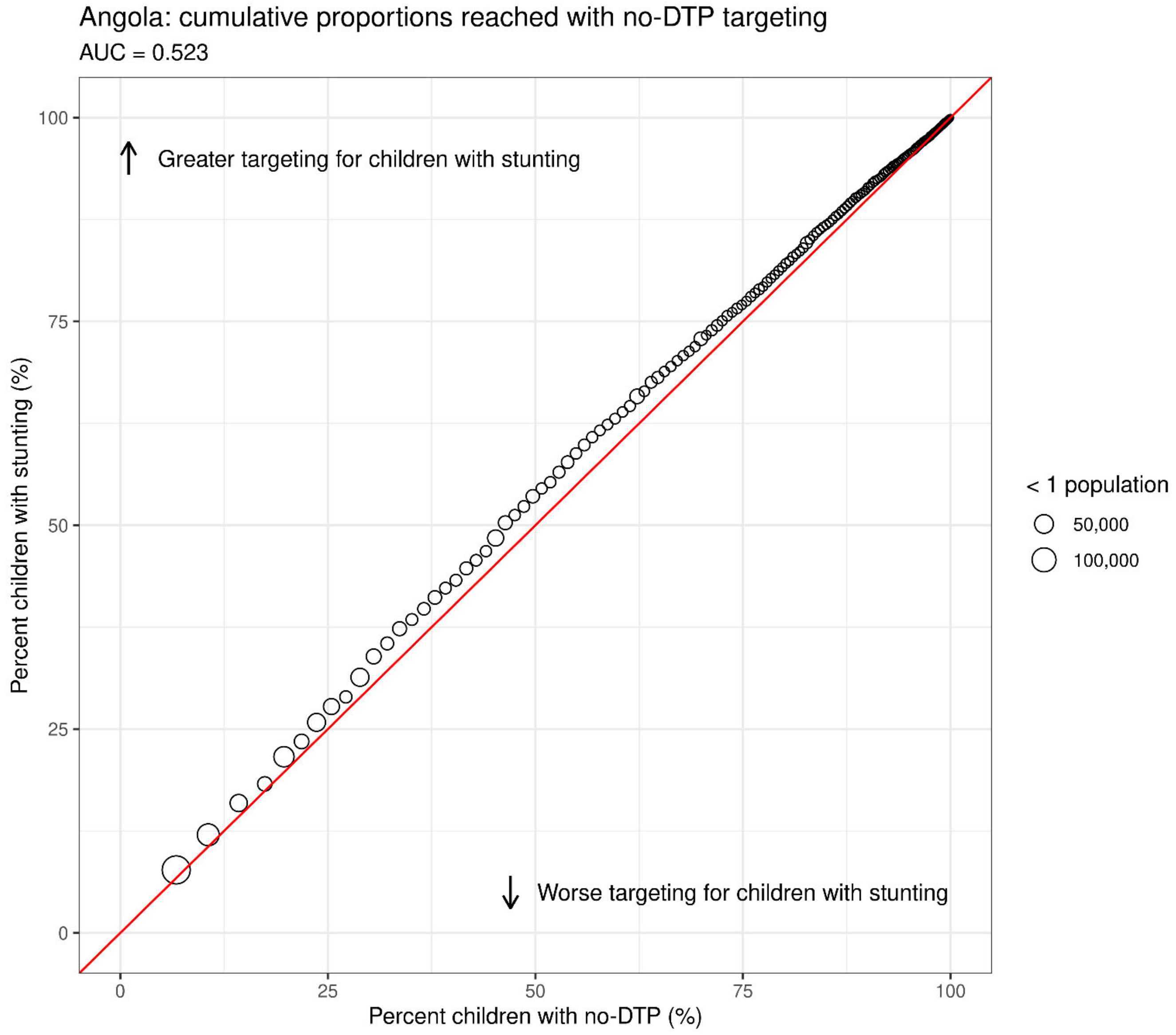

Supplemental Figure S6: Angola, U5M

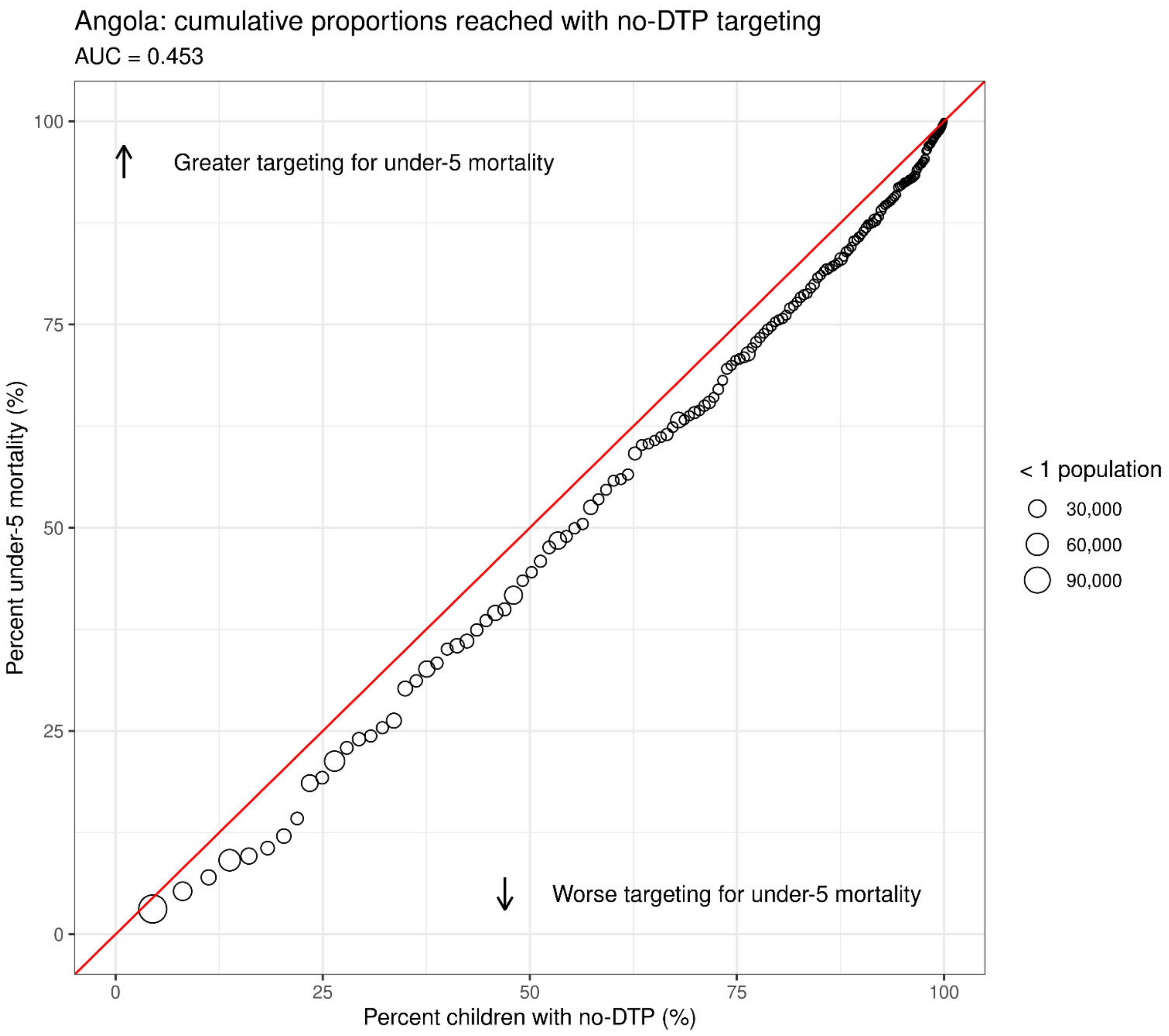

Supplemental Figure S7: Angola, missed ITNs

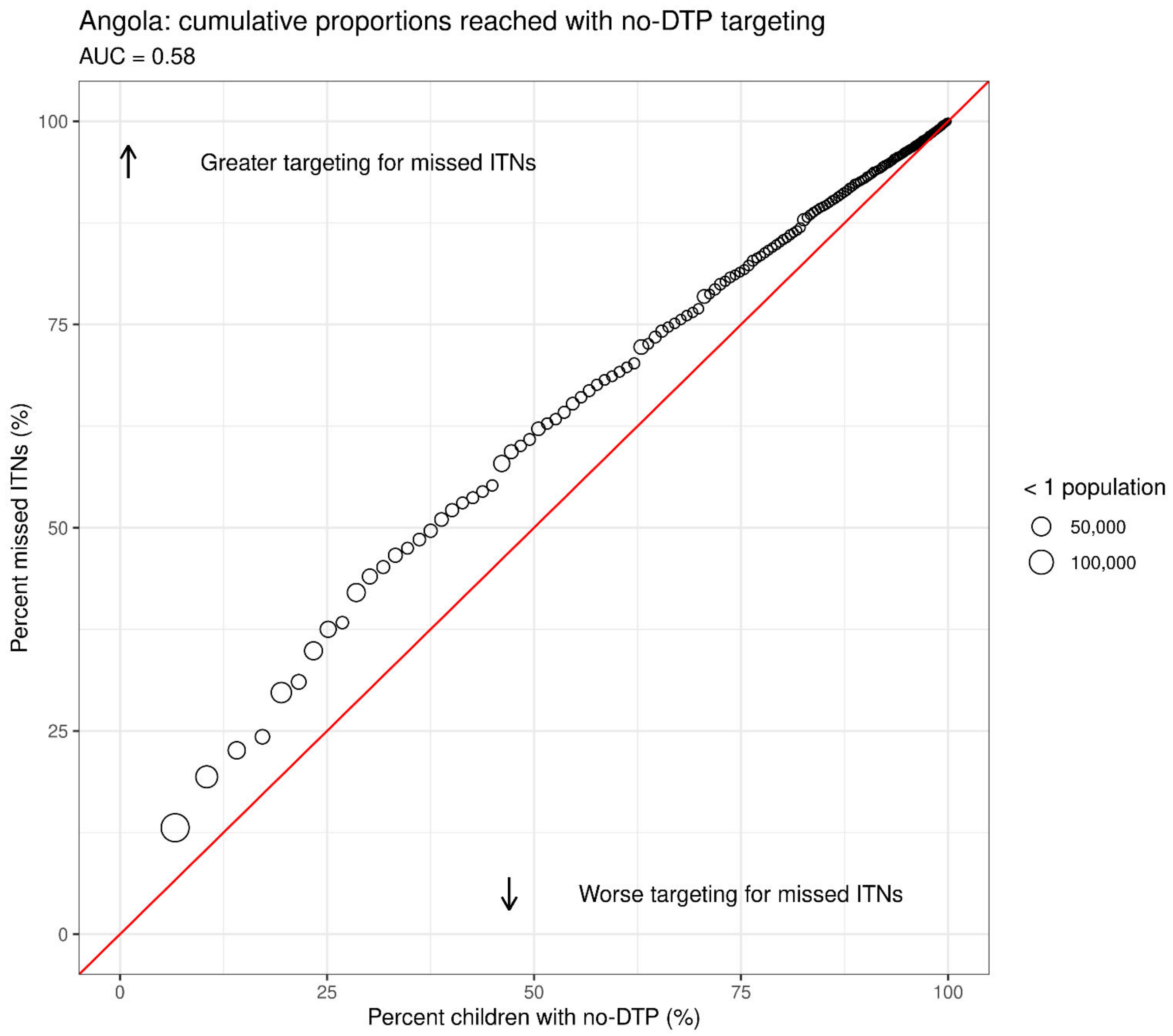

Supplemental Figure S8: Angola, missed ORT

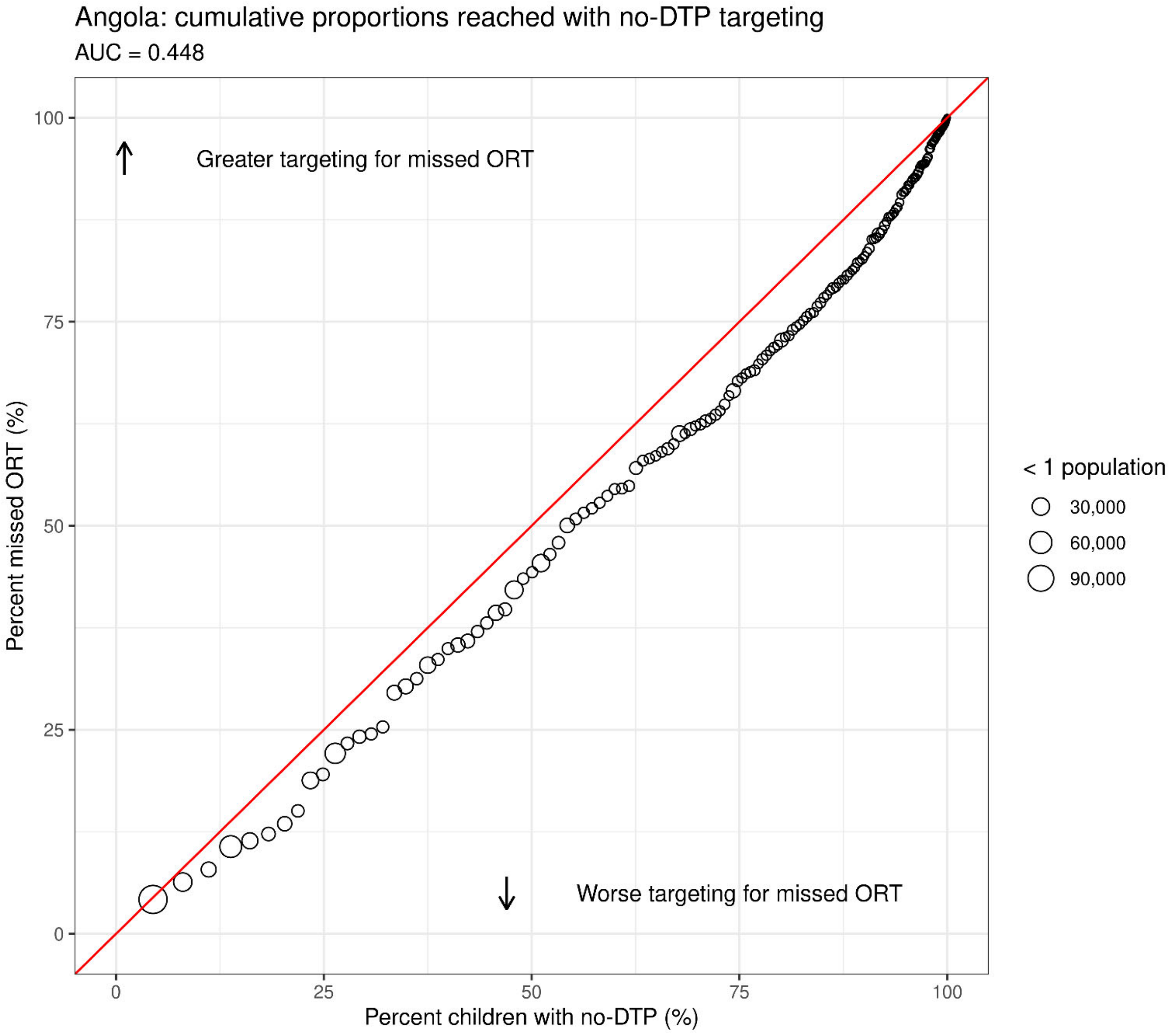

Supplemental Figure S9: Angola, LF

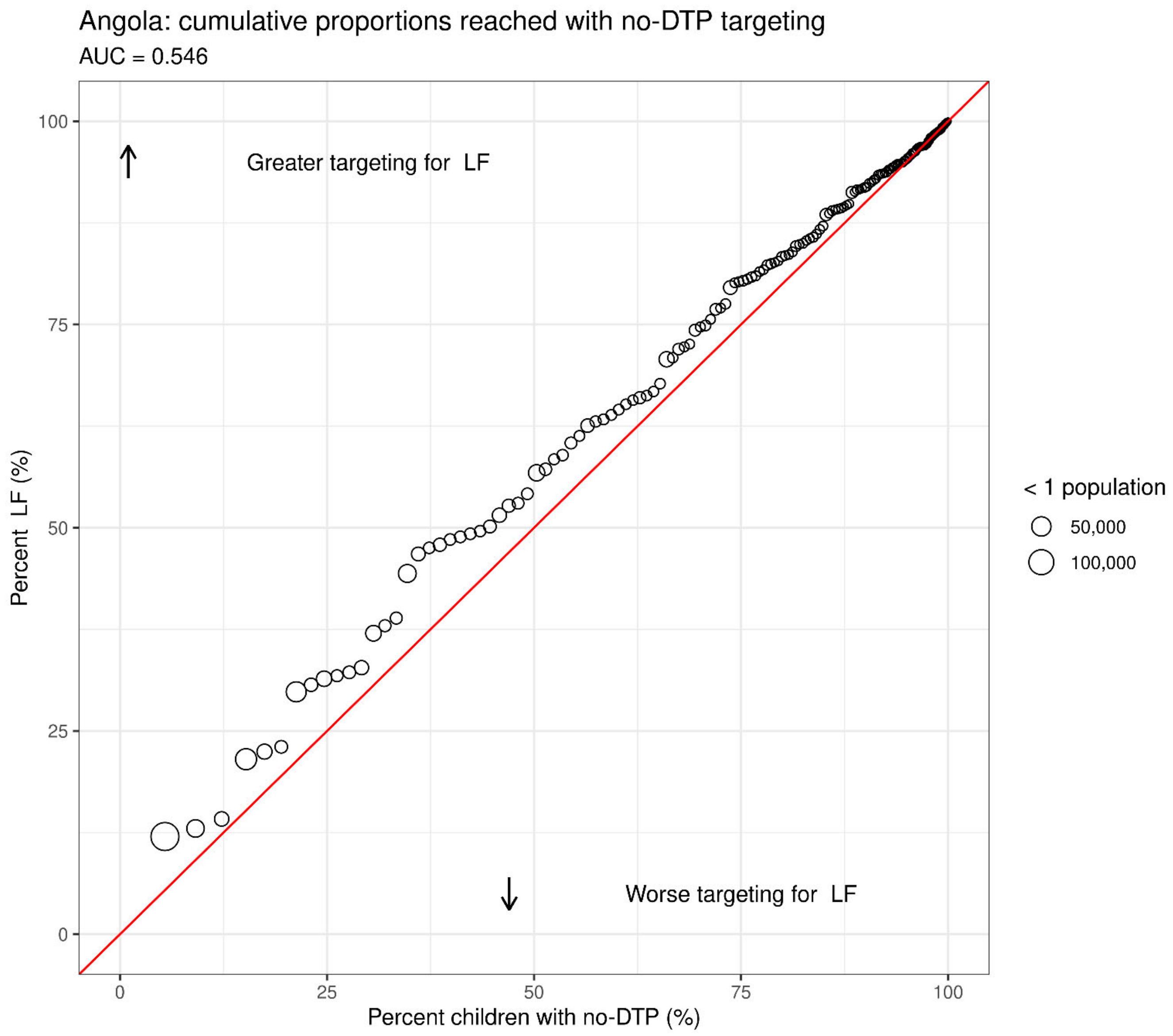

Supplemental Figure S10: DRC, stunting

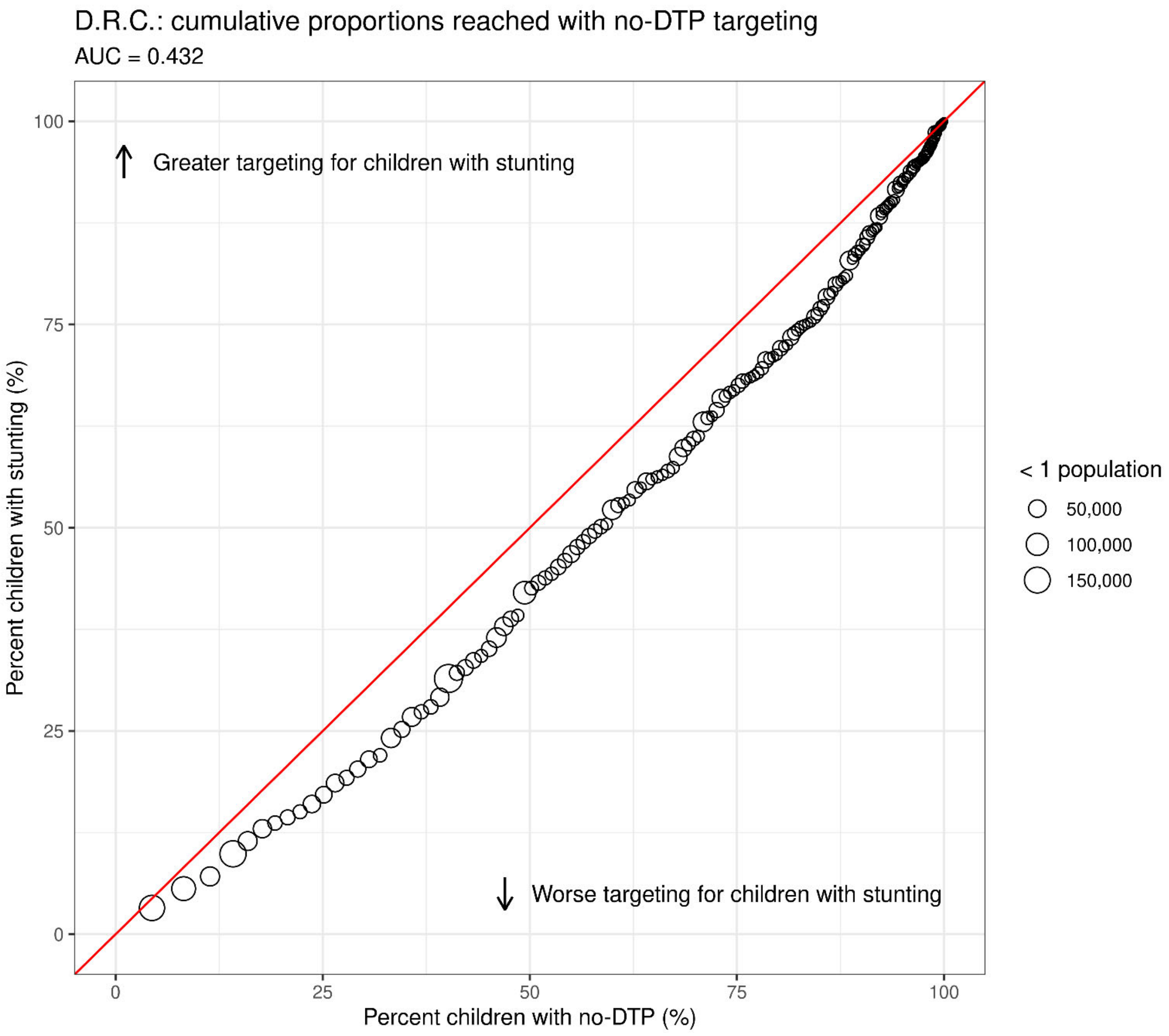

Supplemental Figure S11: DRC, U5M

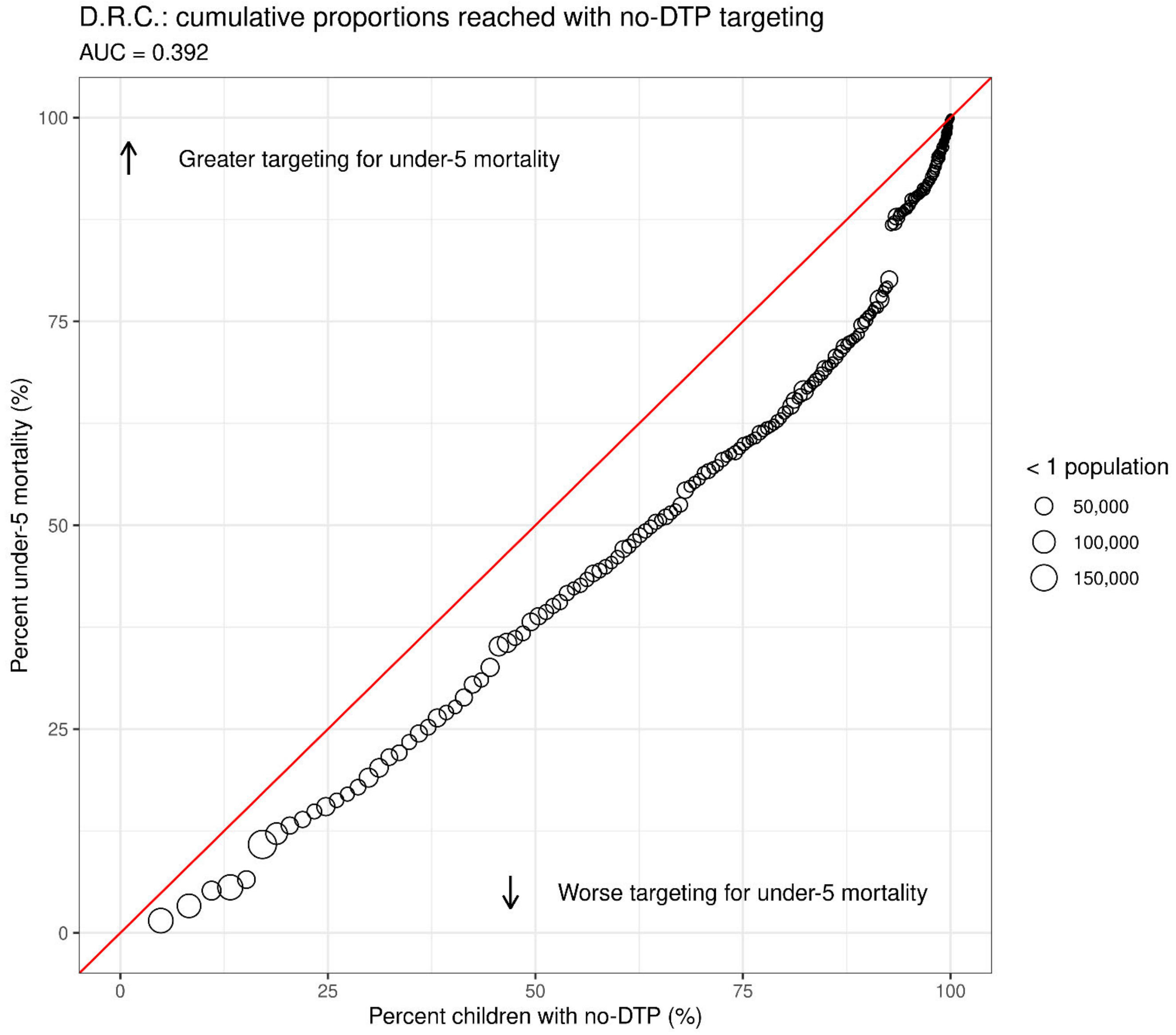

Supplemental Figure S12: DRC, missed ITNs

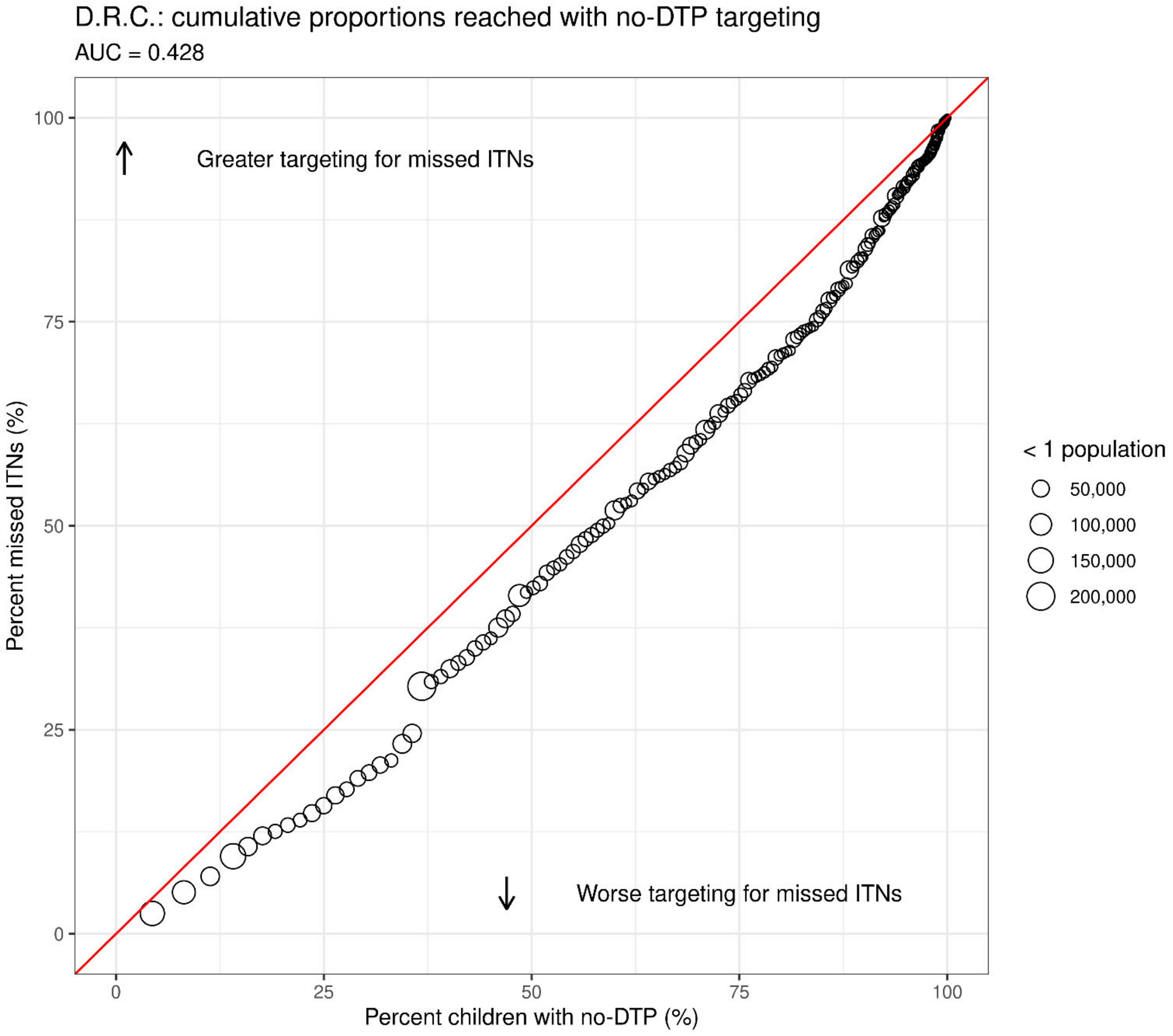

Supplemental Figure S13: DRC, missed ORT

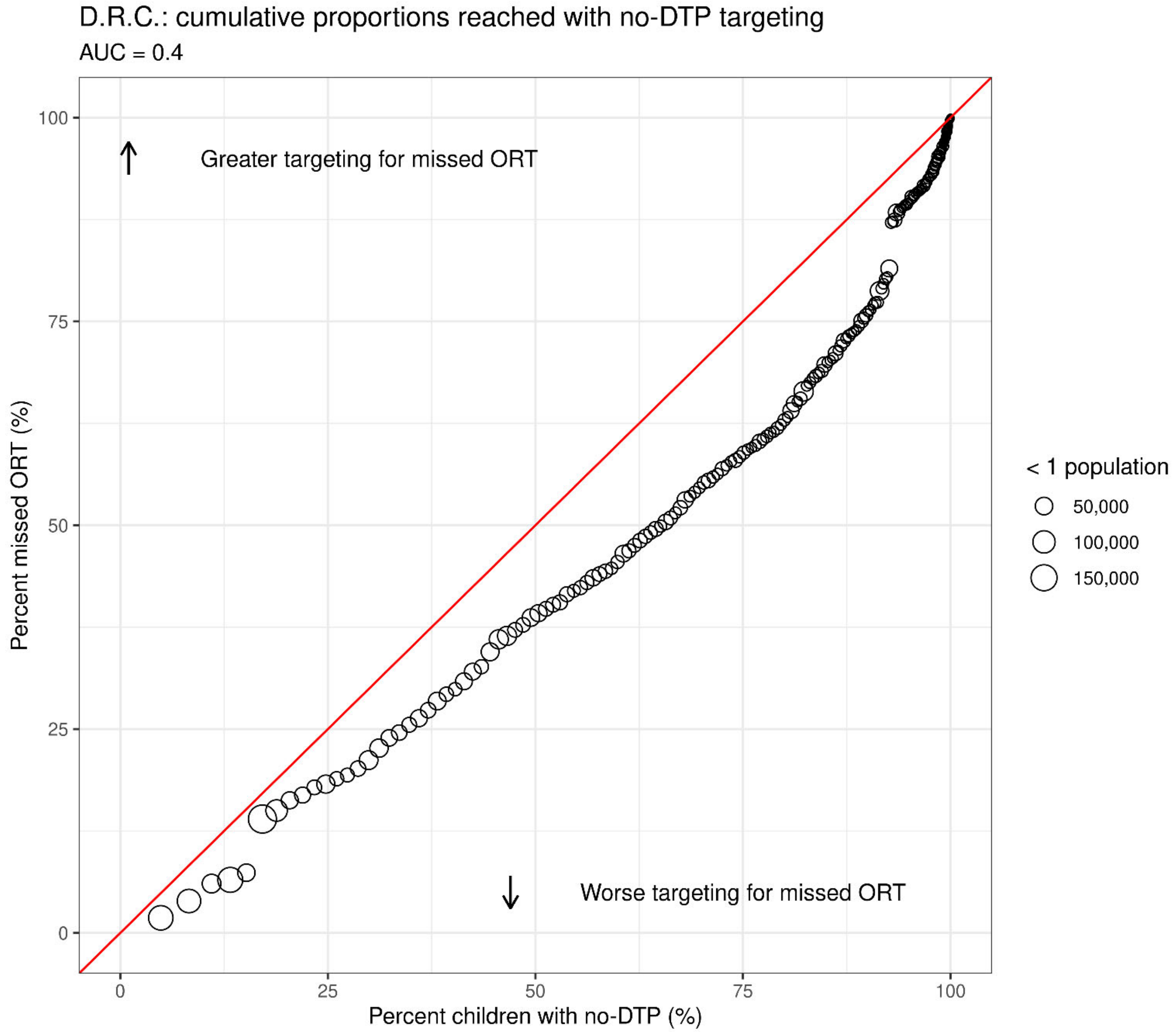

Supplemental Figure S14: DRC, LF

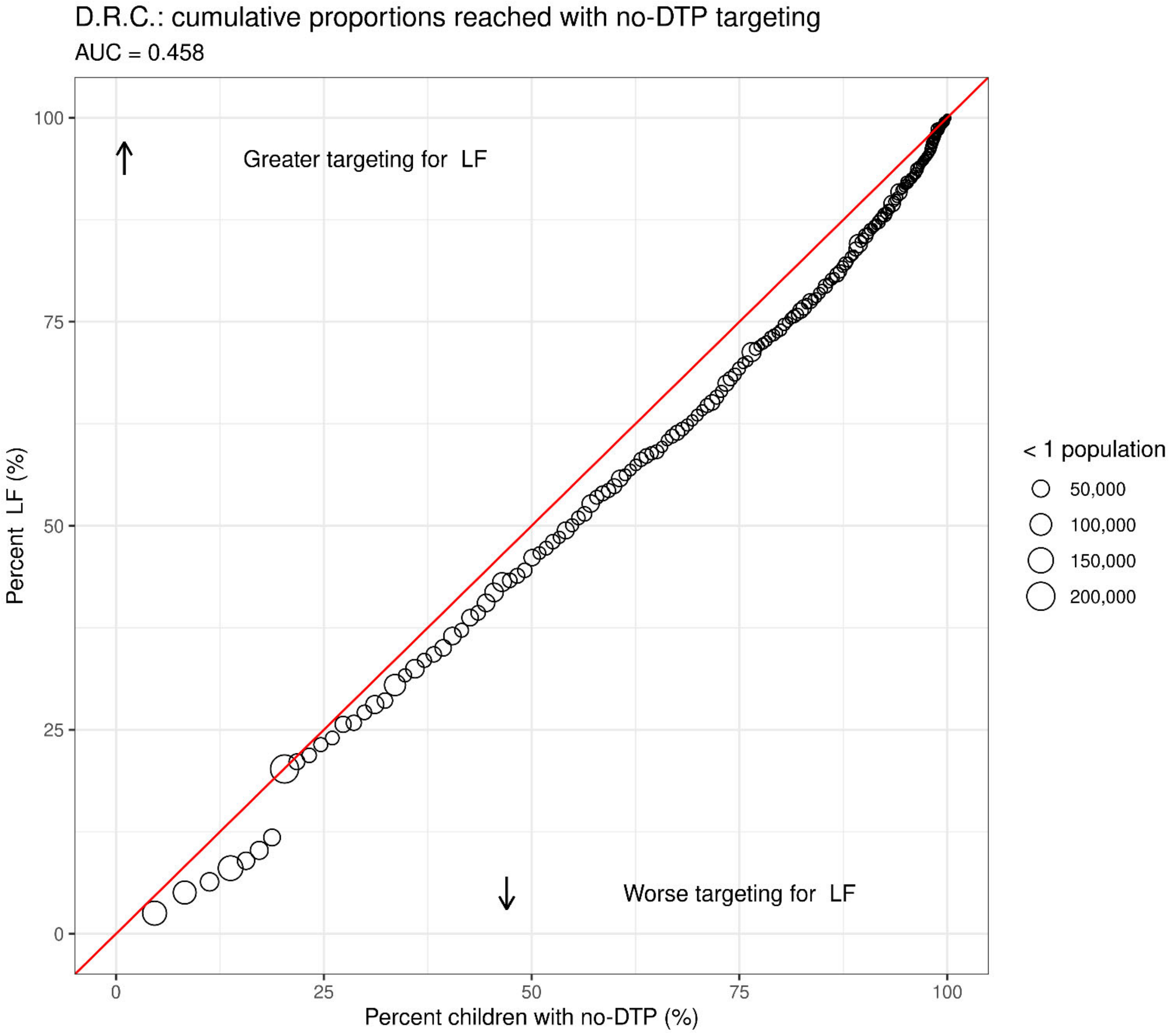

Supplemental Figure S15: Ethiopia, stunting

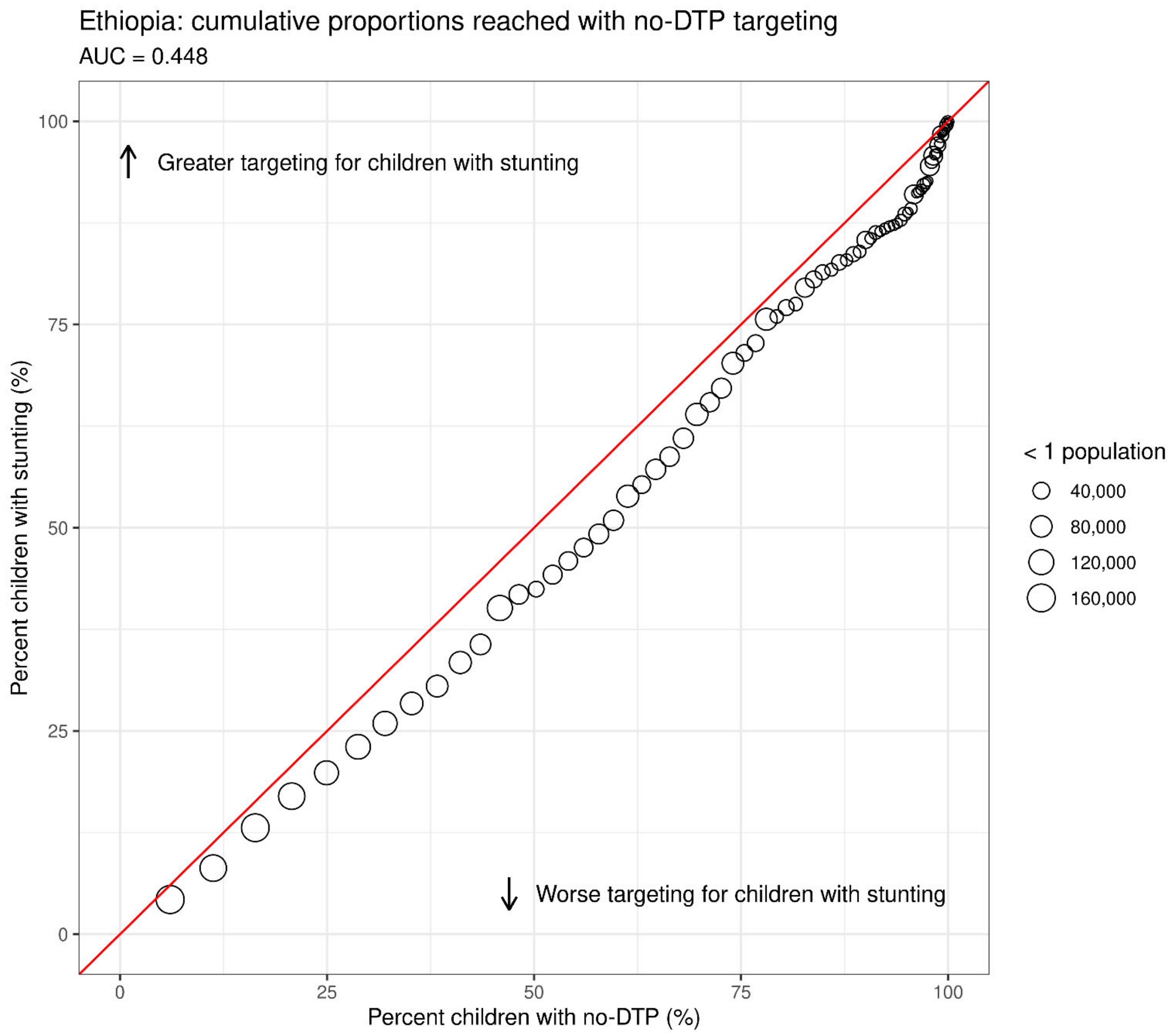

Supplemental Figure S16: Ethiopia, U5M

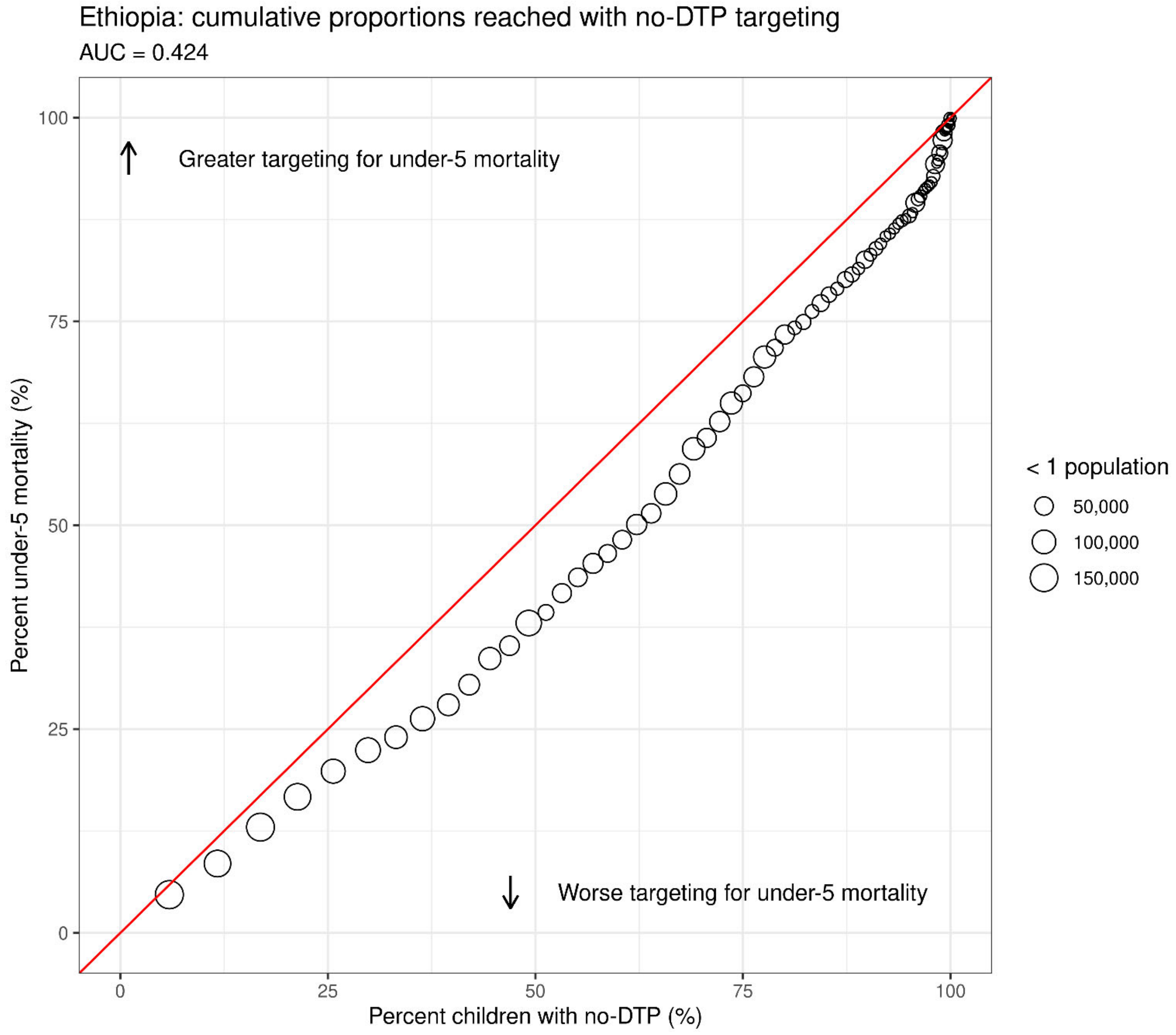

Supplemental Figure S17: Ethiopia, missed ITNs

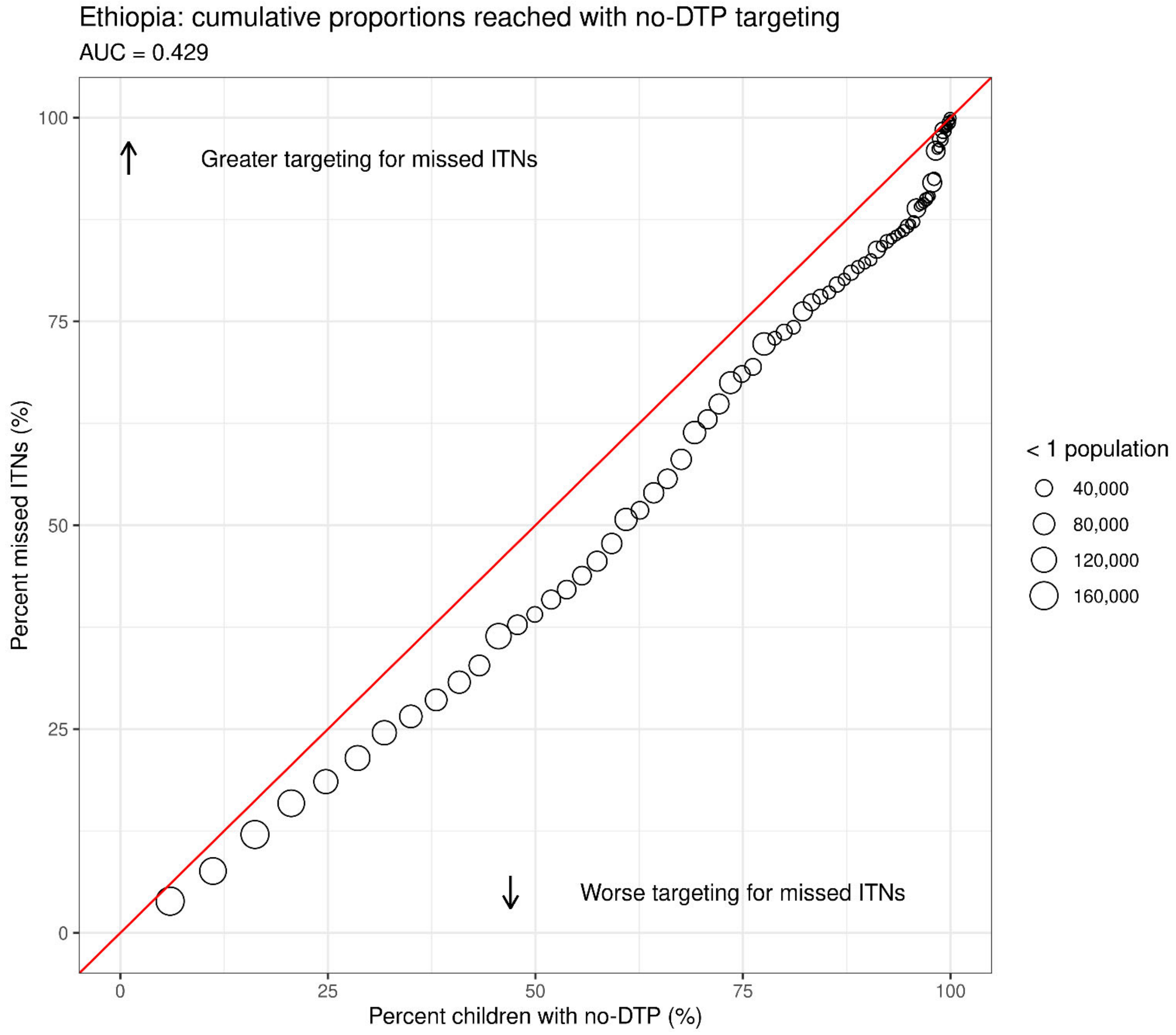

Supplemental Figure S18: Ethiopia, missed ORT

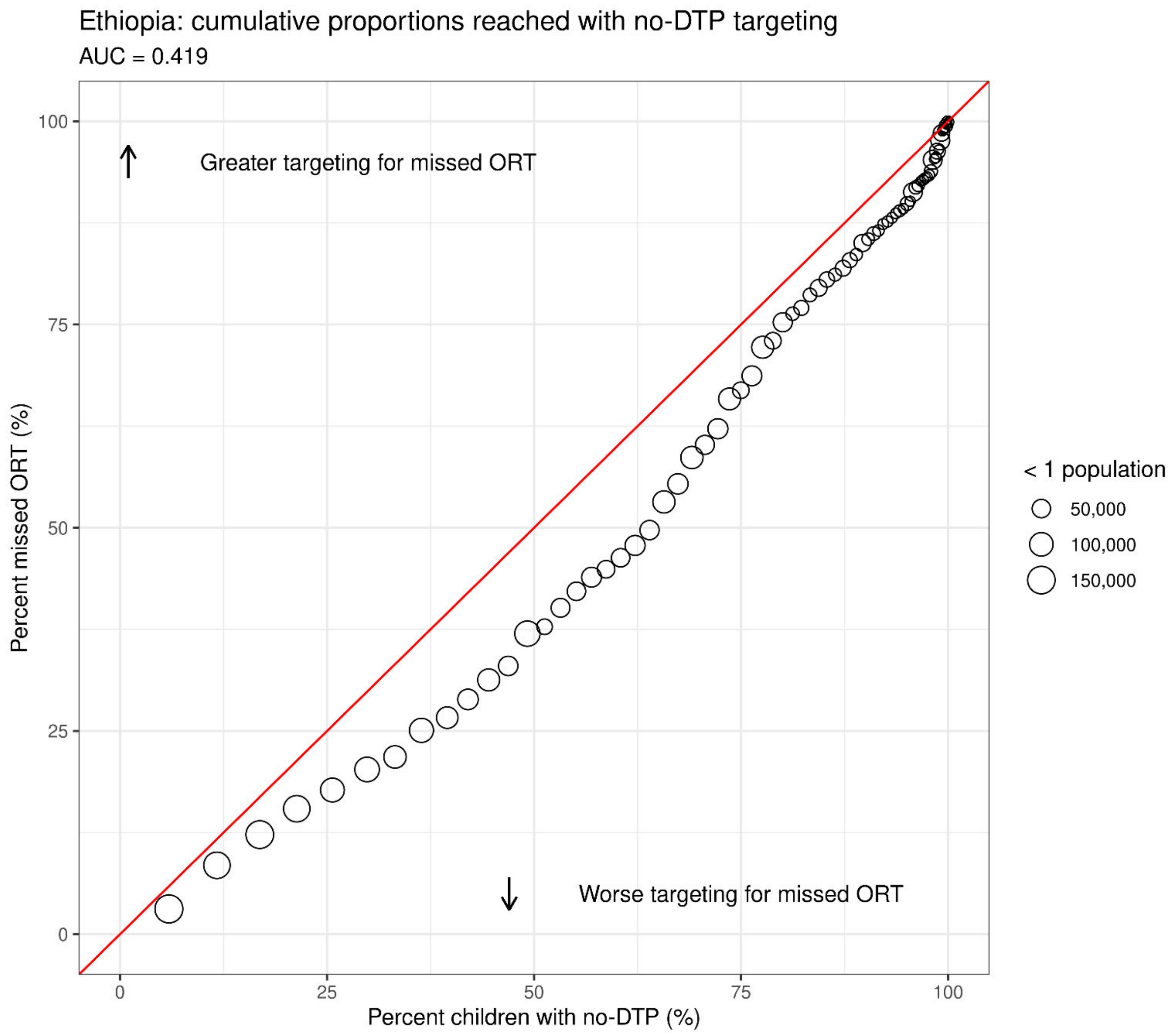

Supplemental Figure S19: Ethiopia, LF

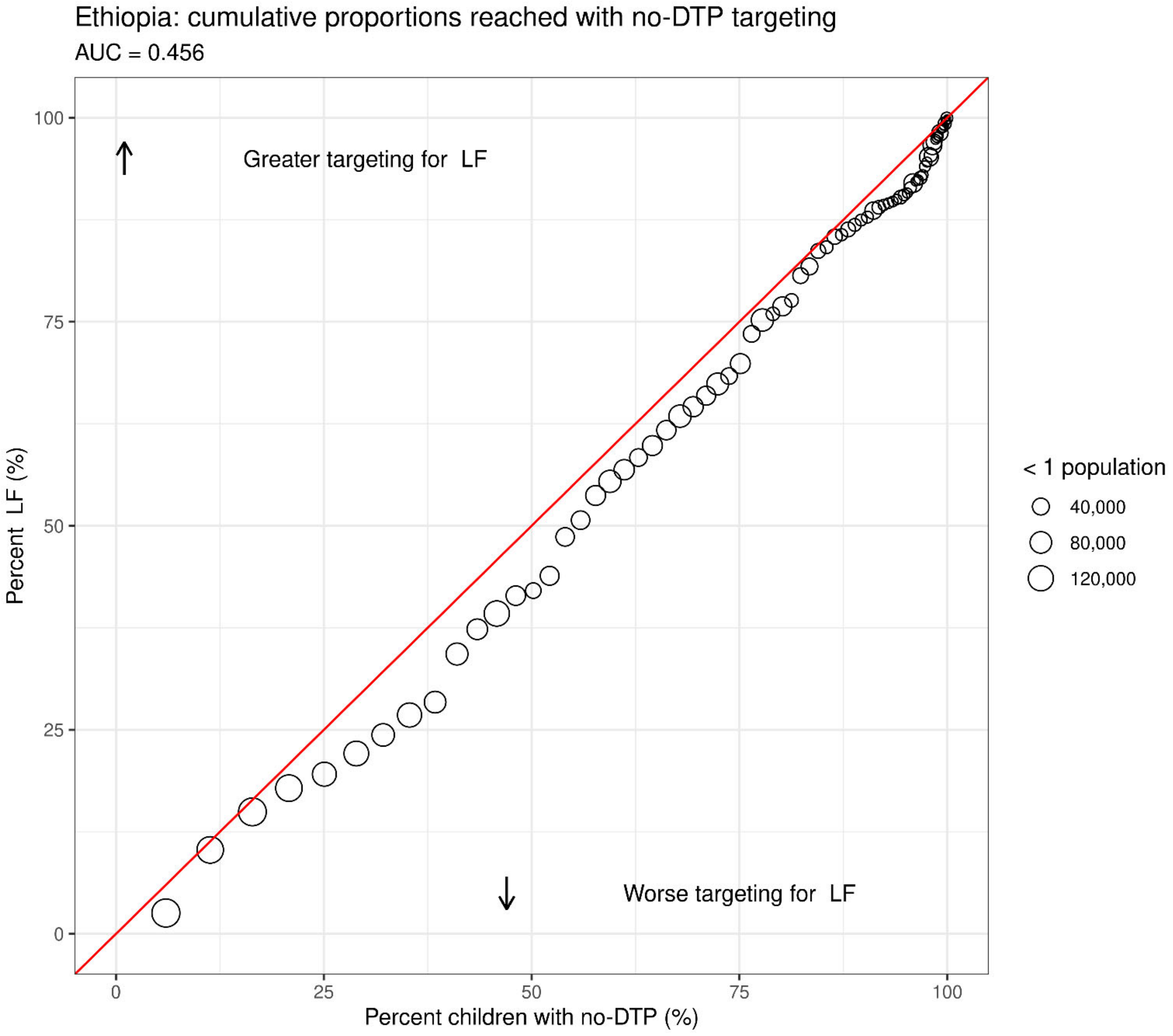

Supplemental Figure S20: Indonesia, stunting

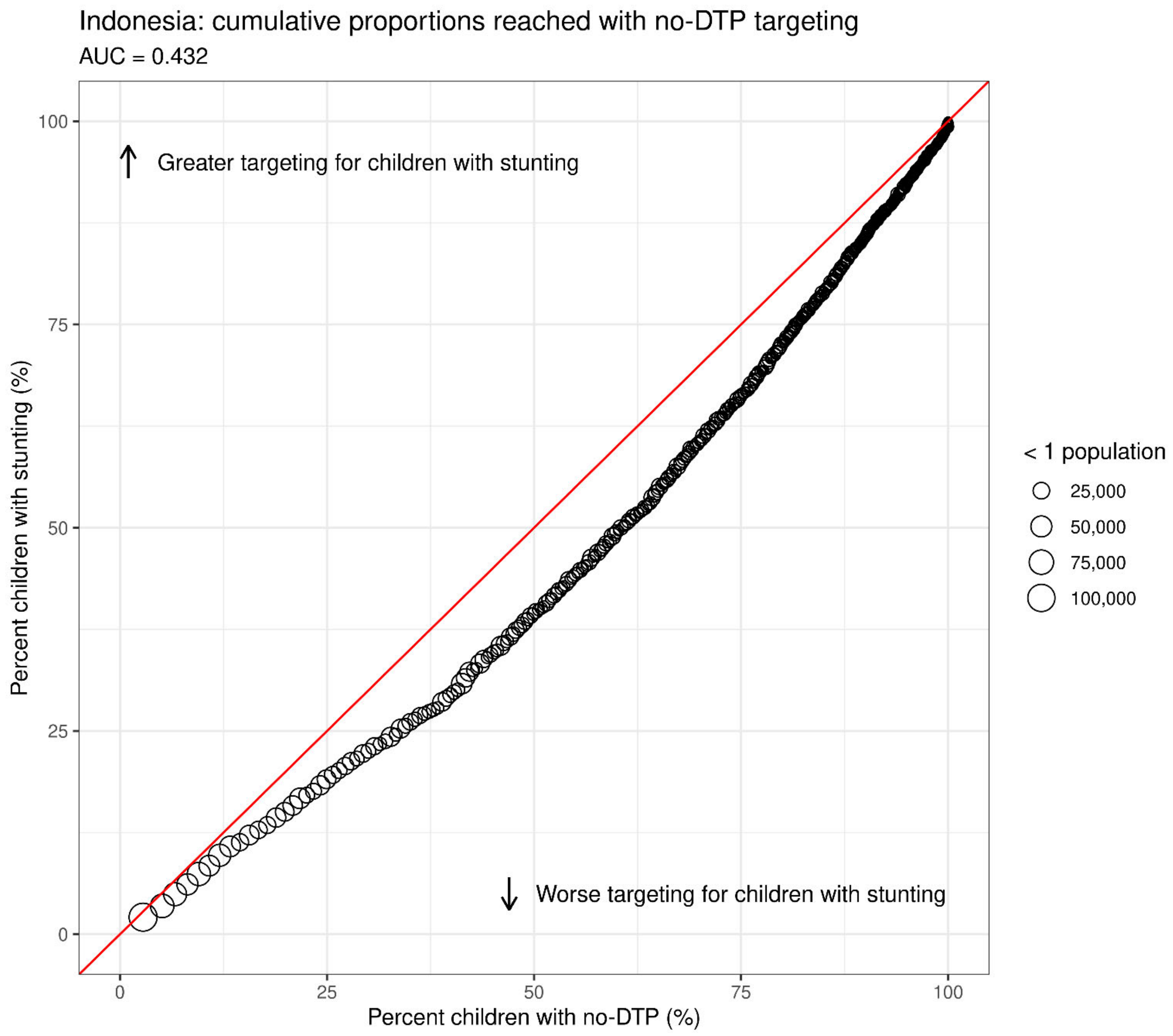

Supplemental Figure S21: Indonesia, U5M

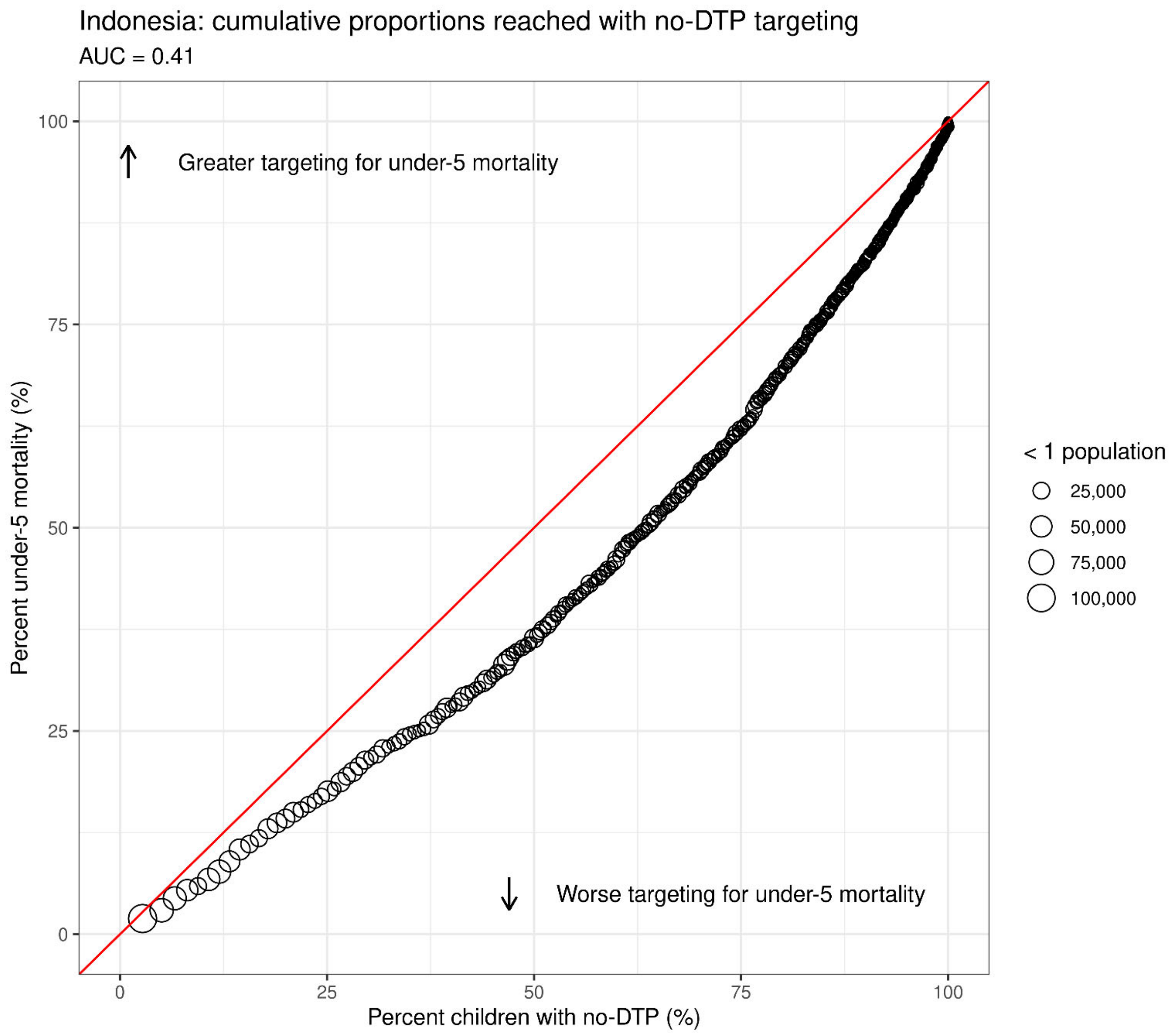

Supplemental Figure S22: Indonesia, missed ORT

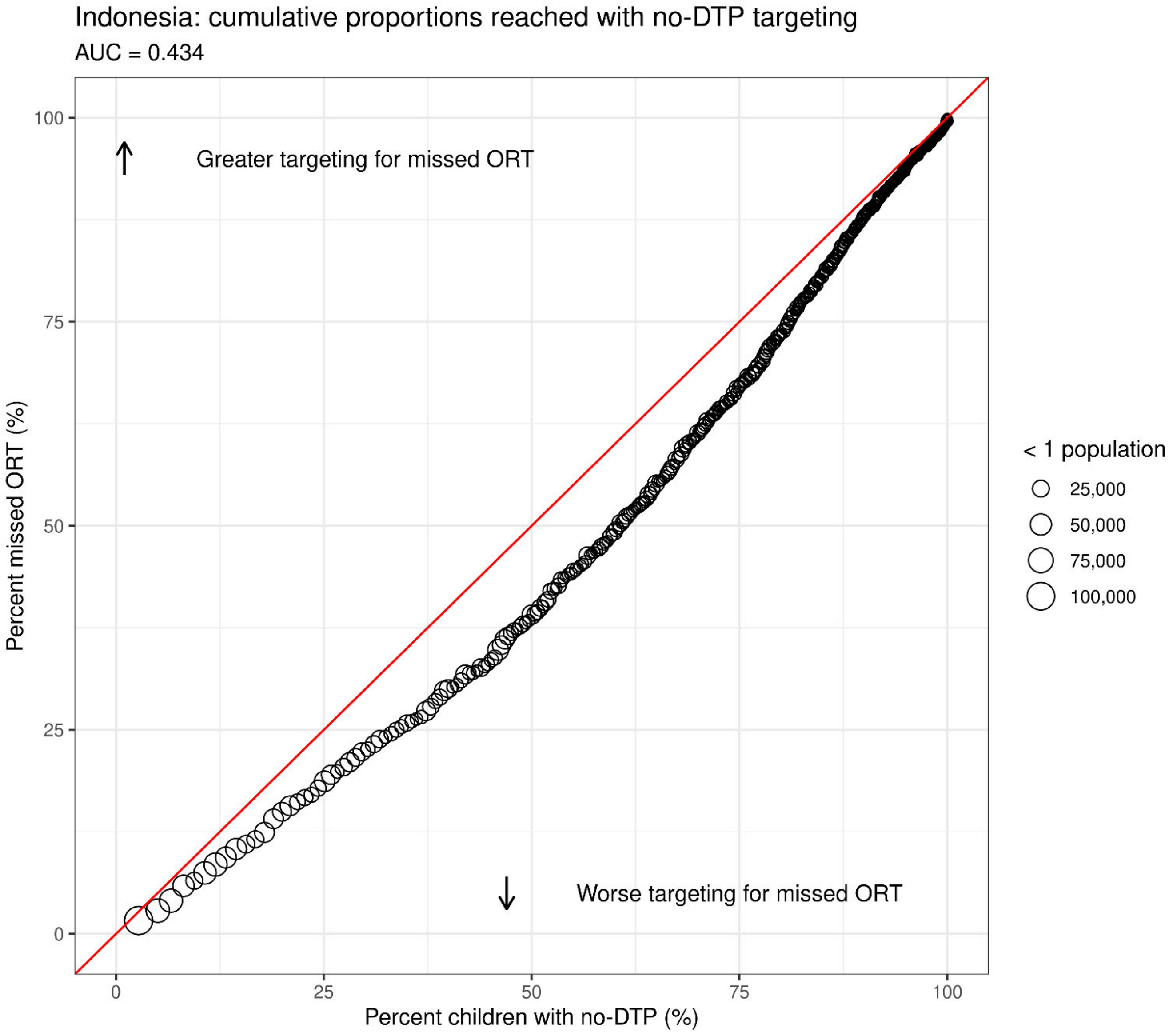

Supplemental Figure S23: Indonesia, LF

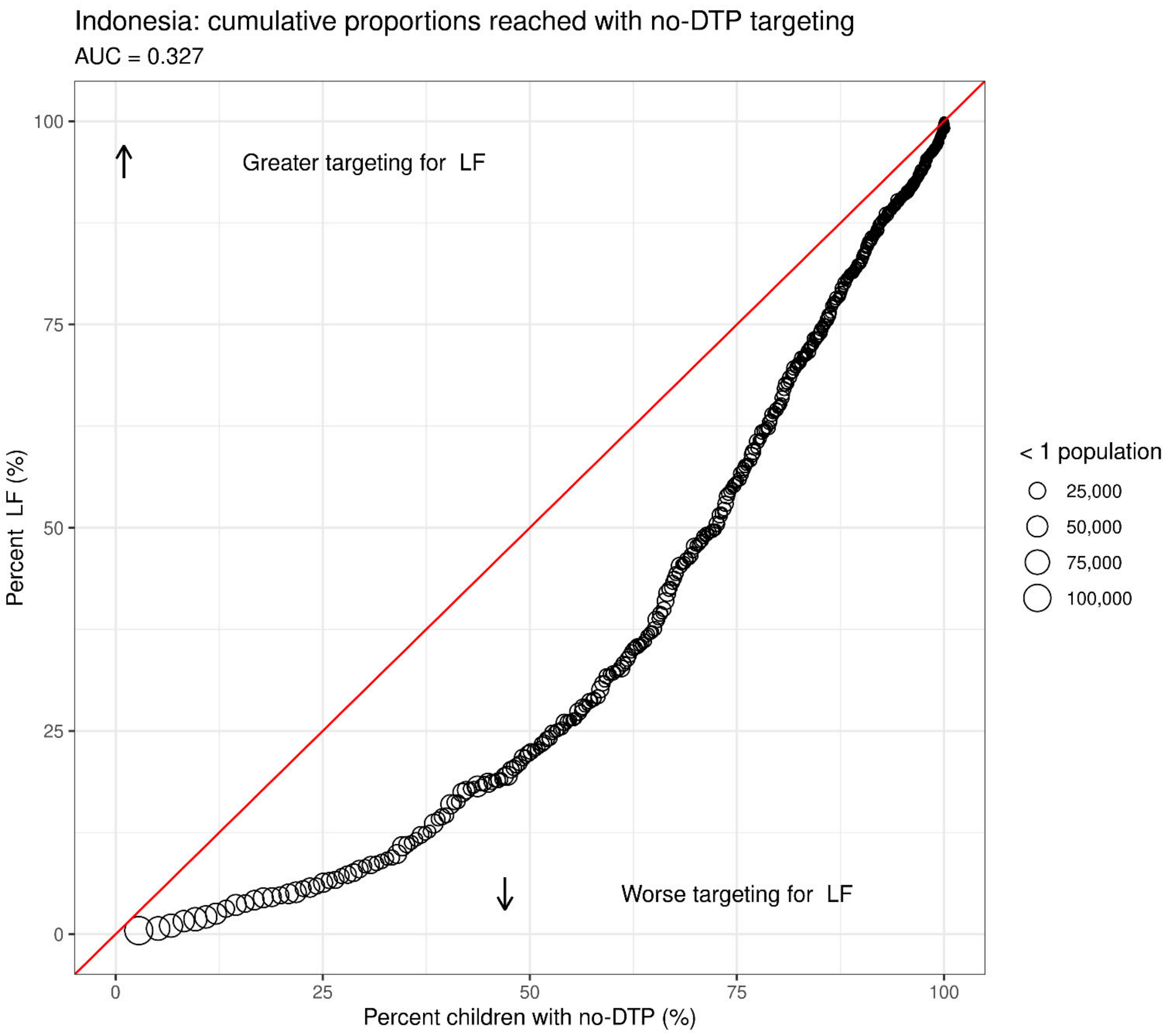

Supplemental Figures S24–S46: Country-specific, all quartile overlap.

Country-specific overlap between no-DTP and each indicator (children with stunting, U5M, missed ITNs, missed ORT, LF), for each country (Nigeria, Angola, DRC, Ethiopia and Indonesia), for all categorization quartiles. The top row (a) shows categorization based on prevalence, while the bottom row (b) shows categorization based on counts. Population-weighted quartile ranges for no-DTP and the comparator indicator are delineated in the bivariate color legends (center). District-level values are shown both as maps (left) and with scatterplots (right), with colors corresponding to quartile legend values. Point size in scatterplots reflects relative size of under 1 population in each district.

Supplemental Figure S24 Nigeria, U5M

(A) No-DTP and under-5 mortality quartiles (%): Nigeria

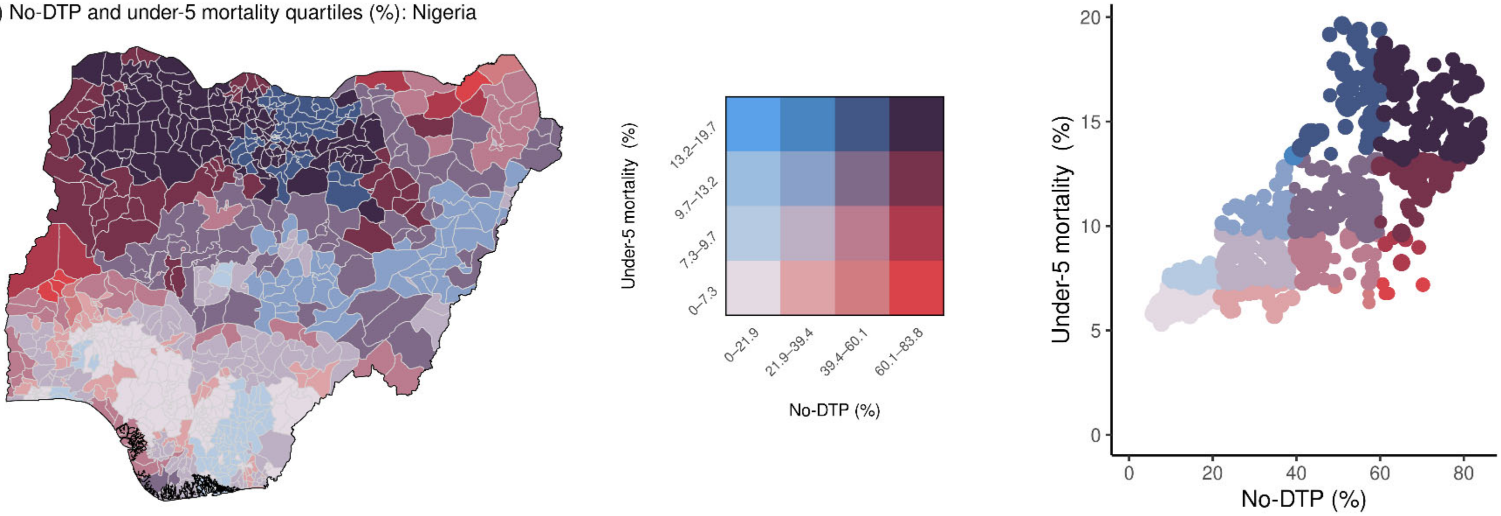

(B) No-DTP and under-5 mortality quartiles (counts): Nigeria

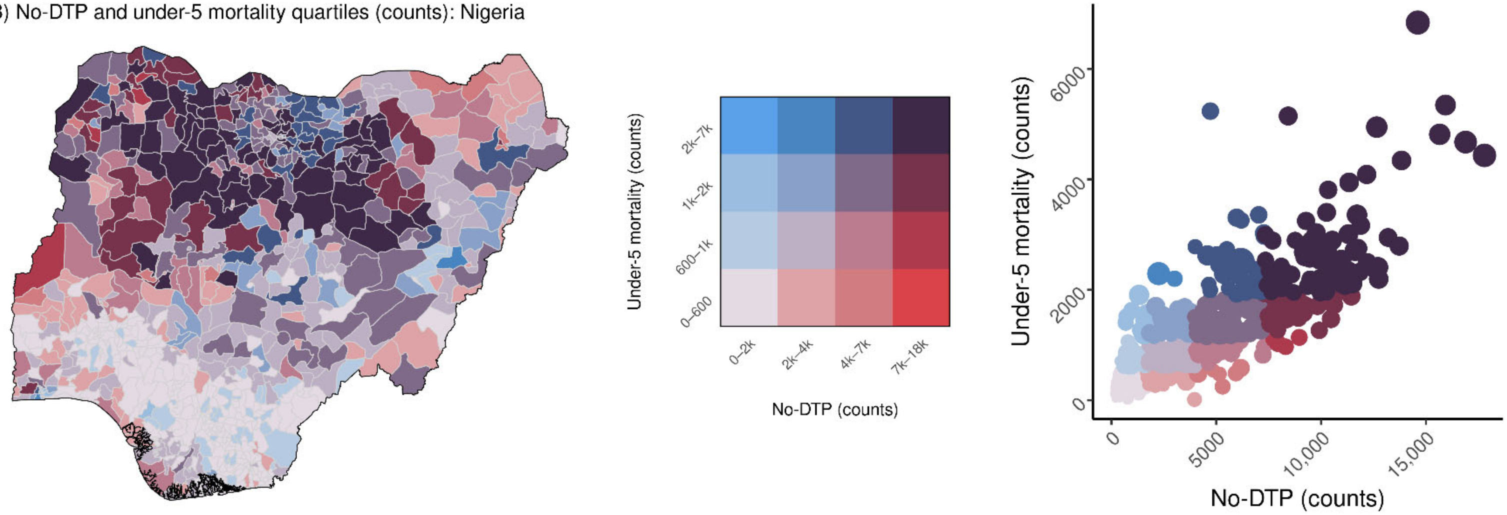

Supplemental Figure S25: Nigeria, missed ITNs

(A) No-DTP and missed ITNs quartiles (%): Nigeria

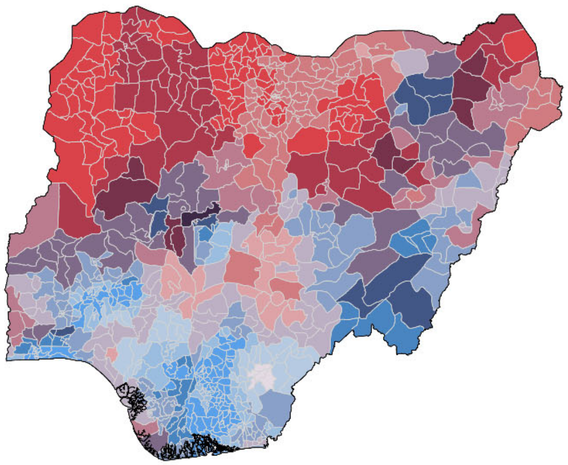

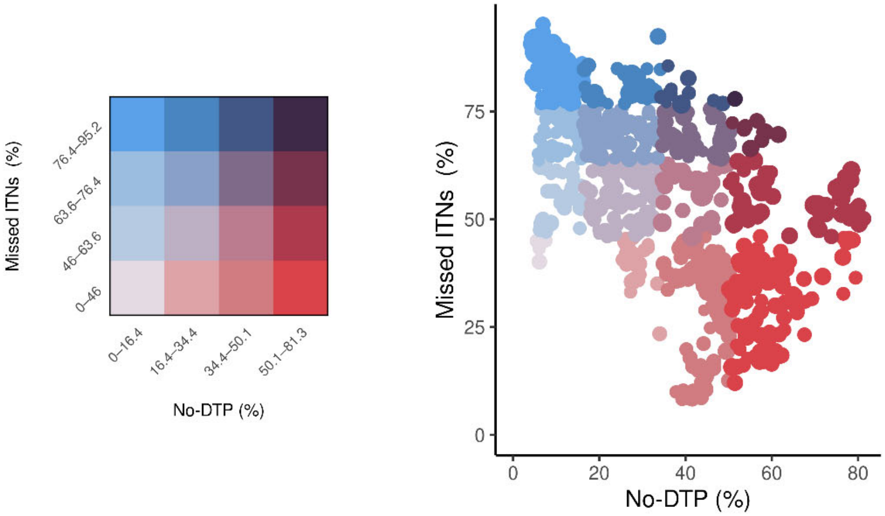

(B) No-DTP and missed ITNs quartiles (counts): Nigeria

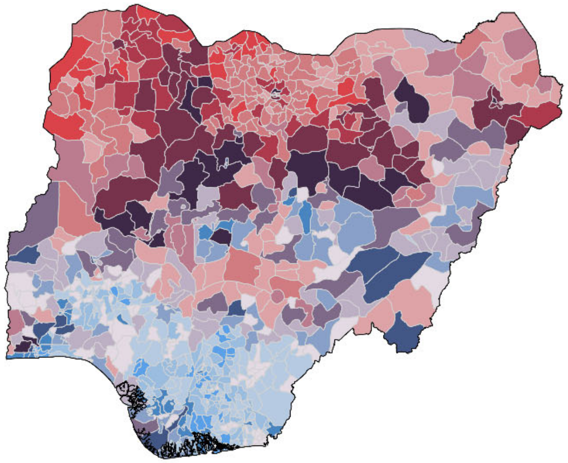

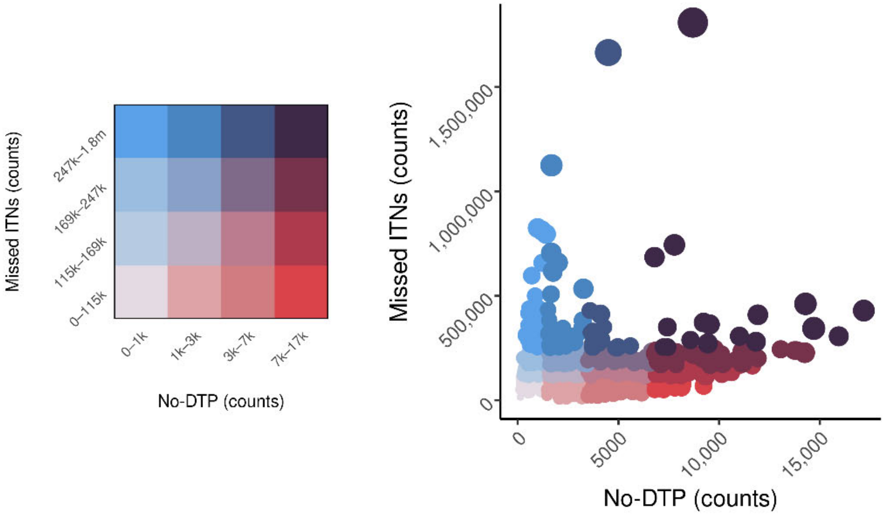

Supplemental Figure S26: Nigeria, missed ORT

(A) No-DTP and missed ORT quartiles (%): Nigeria

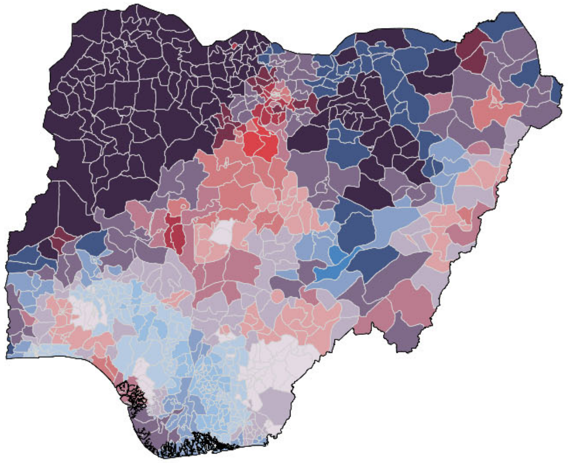

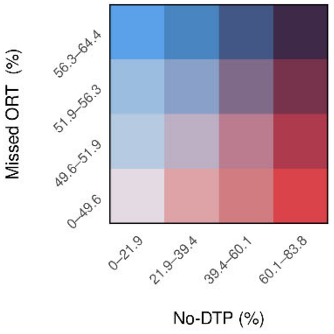

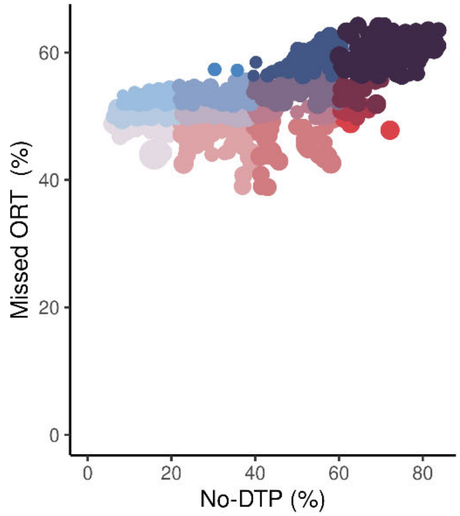

(B) No-DTP and missed ORT quartiles (counts): Nigeria

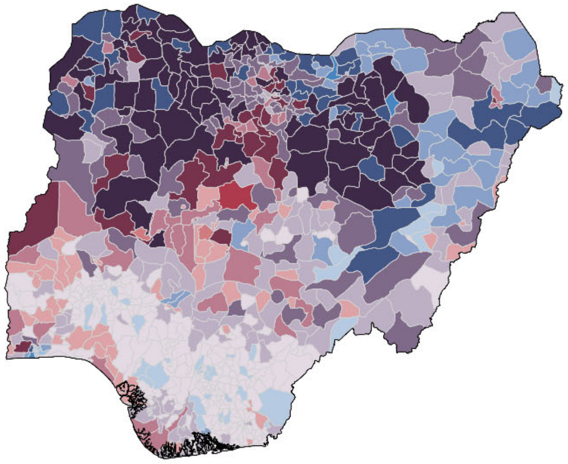

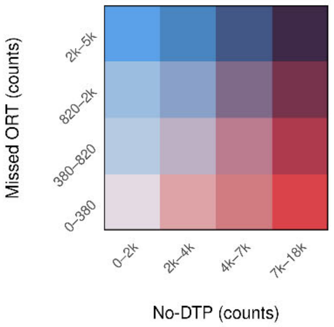

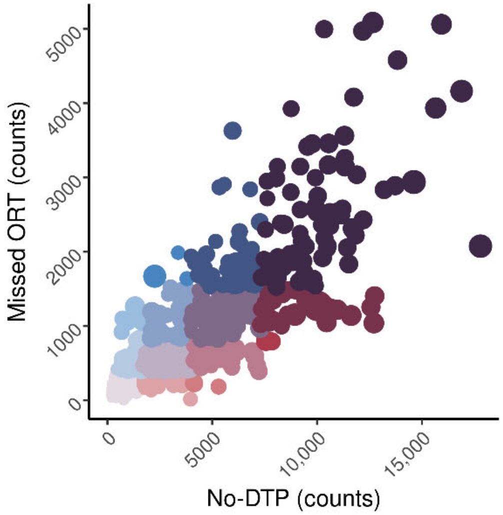

Supplemental Figure S27: Nigeria, LF

(A) No-DTP and LF quartiles (%): Nigeria

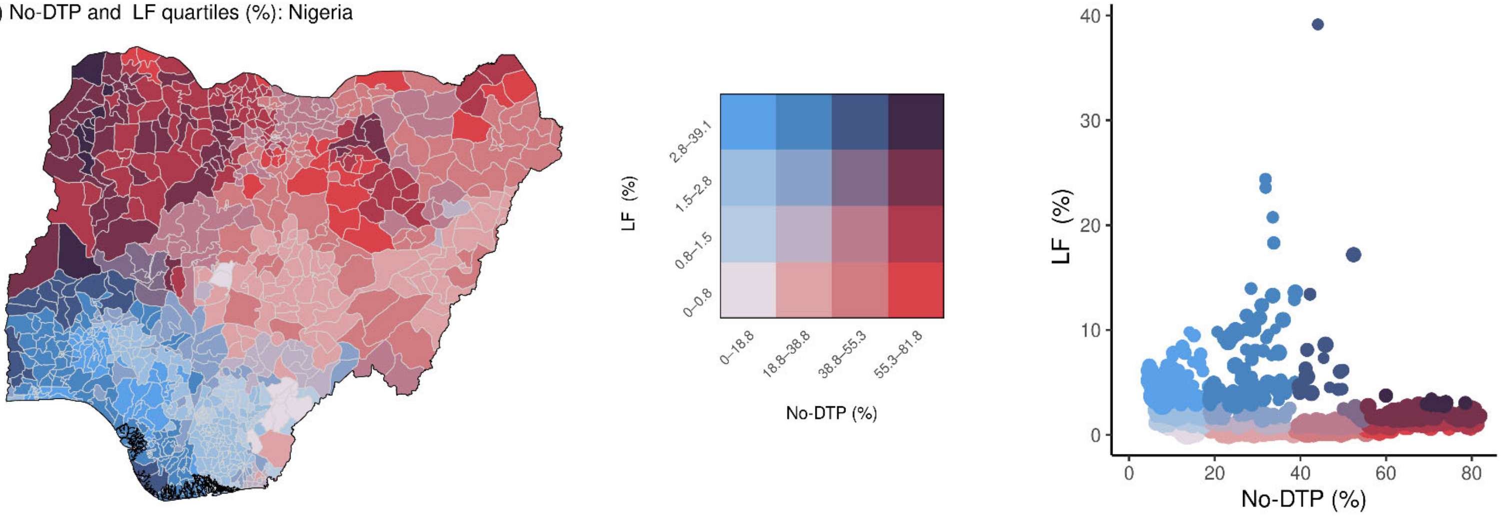

(B) No-DTP and LF quartiles (counts): Nigeria

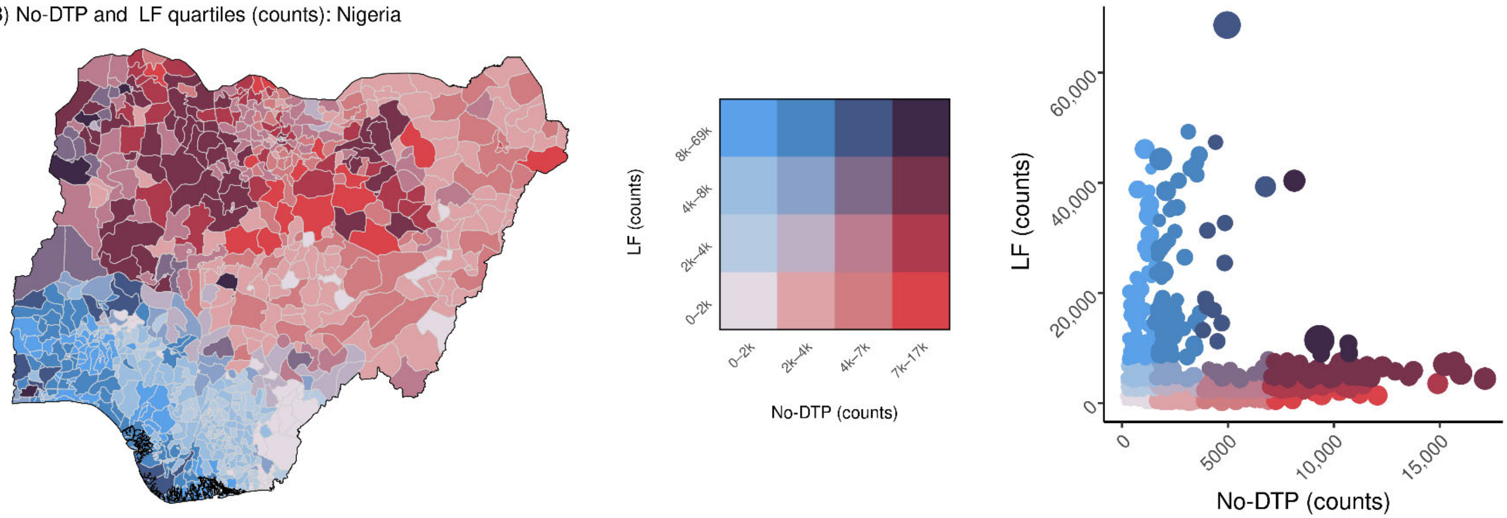

Supplemental Figure S28: Angola, stunting

(A) No-DTP and children with stunting quartiles (%): Angola

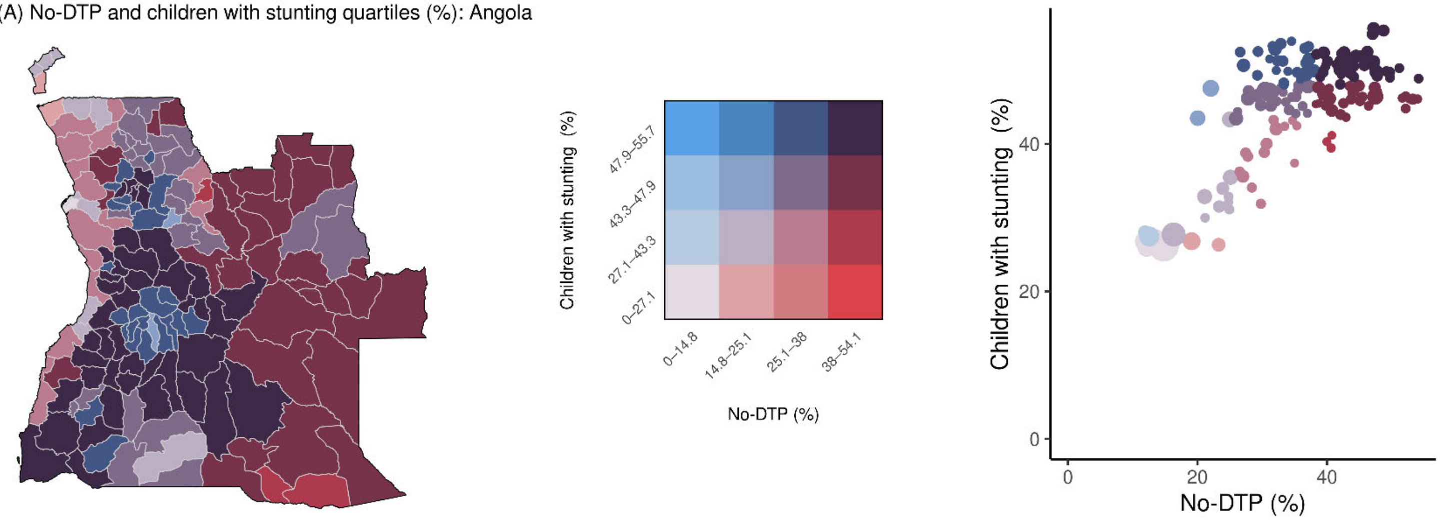

(B) No-DTP and children with stunting quartiles (counts): Angola

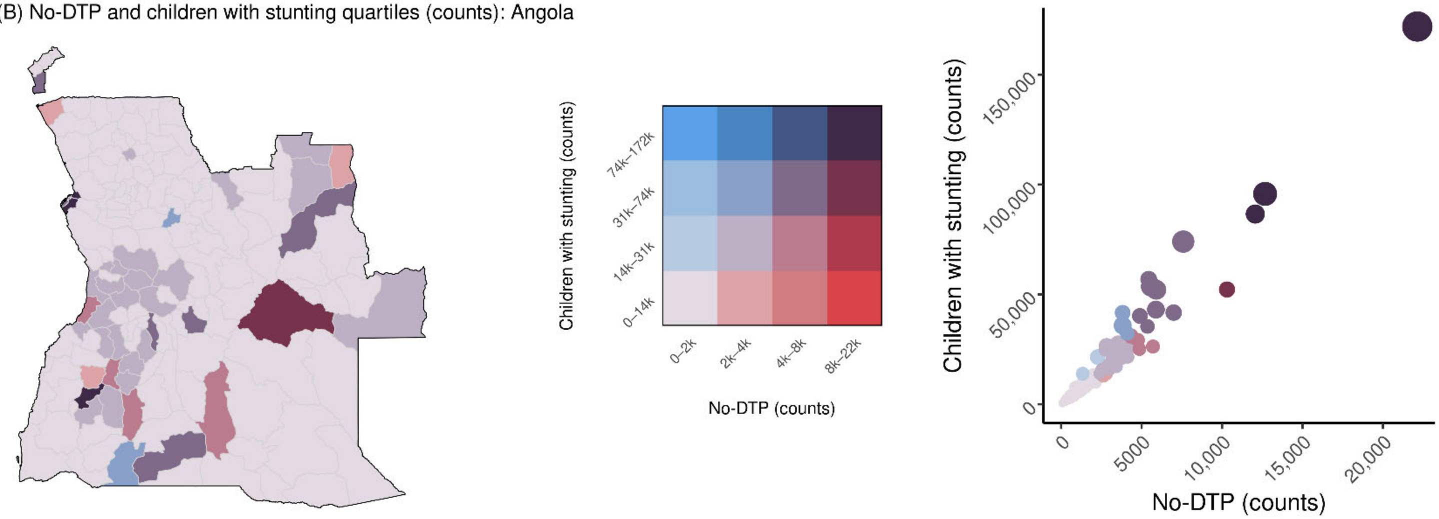

Supplemental Figure S29: Angola, U5M

(A) No-DTP and under-5 mortality quartiles (%): Angola

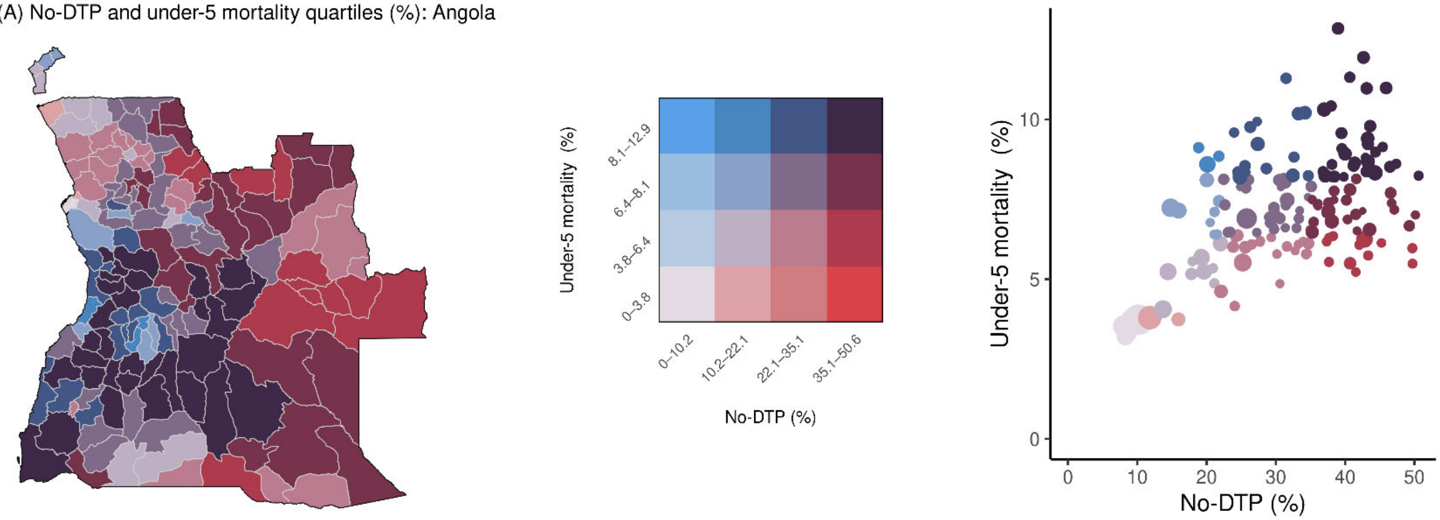

(B) No-DTP and under-5 mortality quartiles (counts): Angola

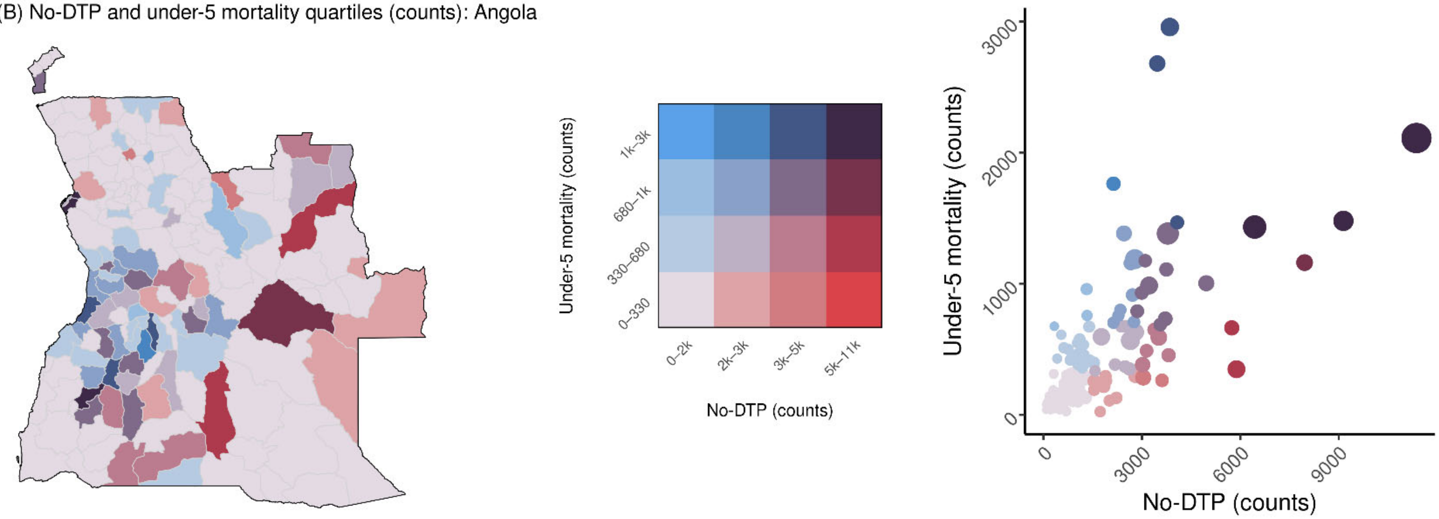

Supplemental Figure S30: Angola, missed ORT

(A) No-DTP and missed ORT quartiles (%): Angola

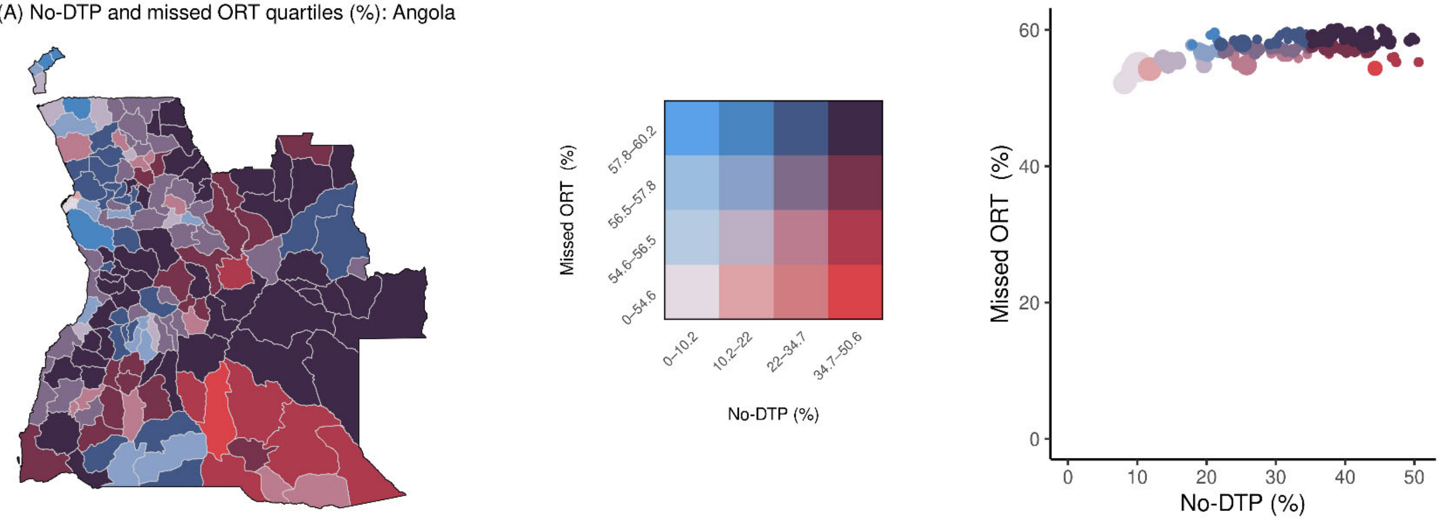

(B) No-DTP and missed ORT quartiles (counts): Angola

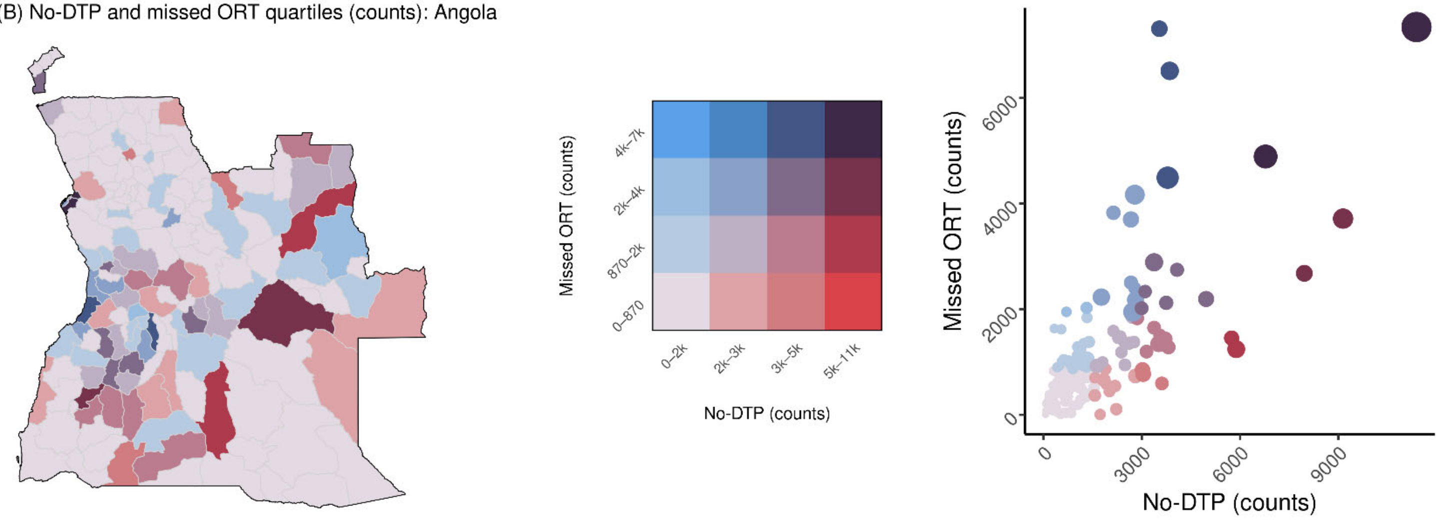

Supplemental Figure S31: Angola, missed ITNs

(A) No-DTP and missed ITNs quartiles (%): Angola

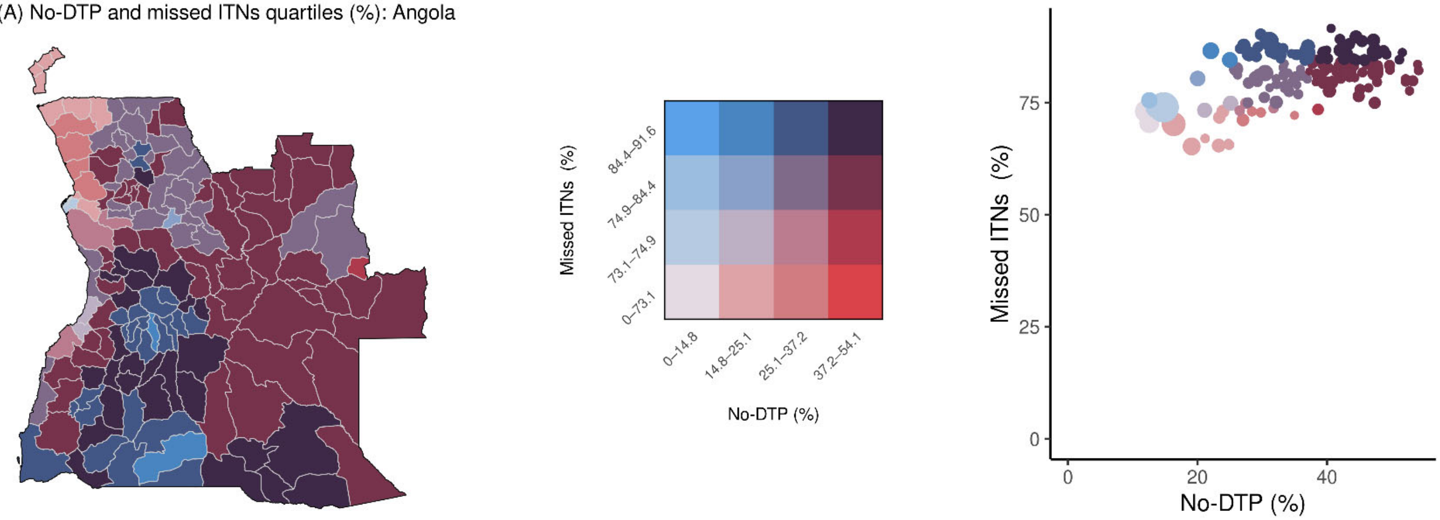

(B) No-DTP and missed ITNs quartiles (counts): Angola

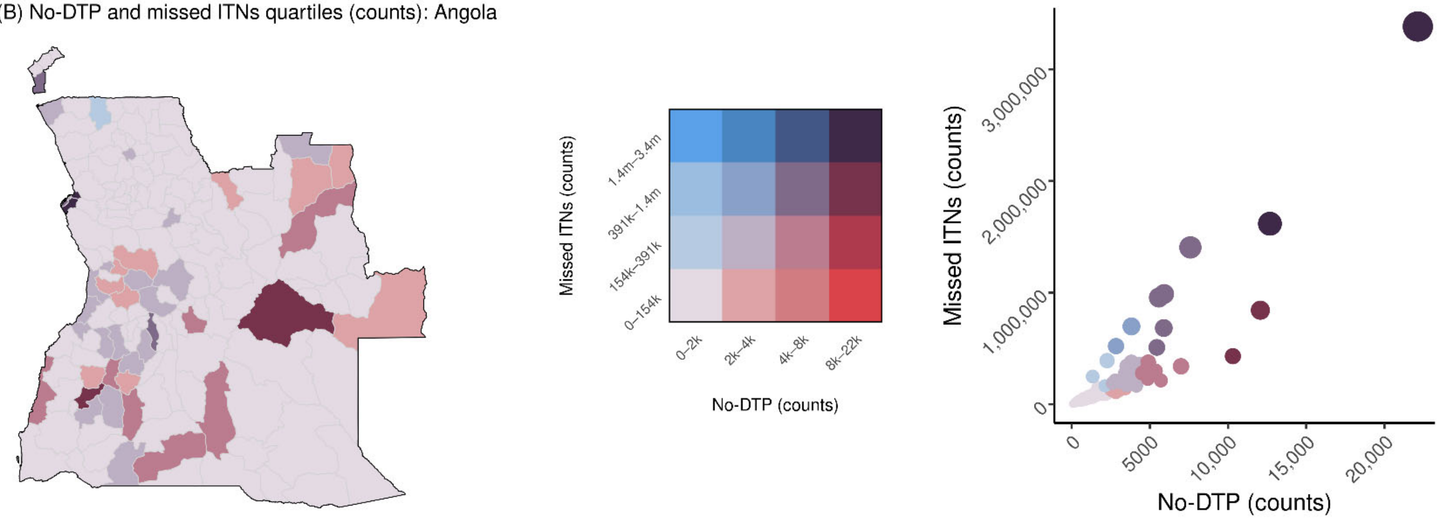

Supplemental Figure S32: Angola, LF

(A) No-DTP and LF quartiles (%): Angola

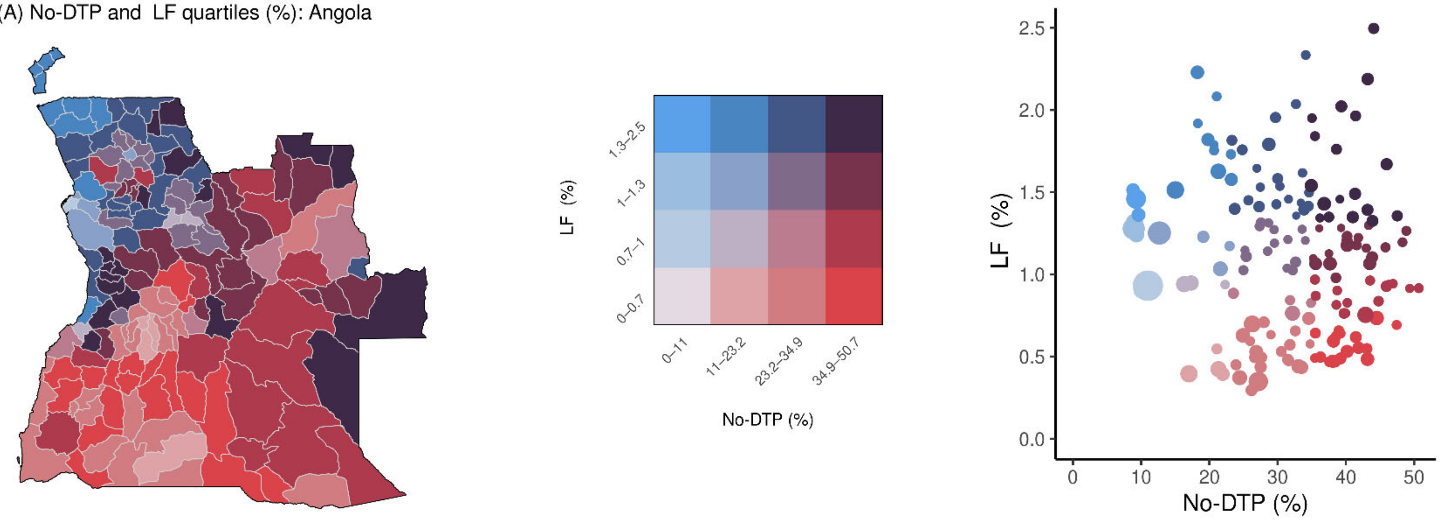

(B) No-DTP and LF quartiles (counts): Angola

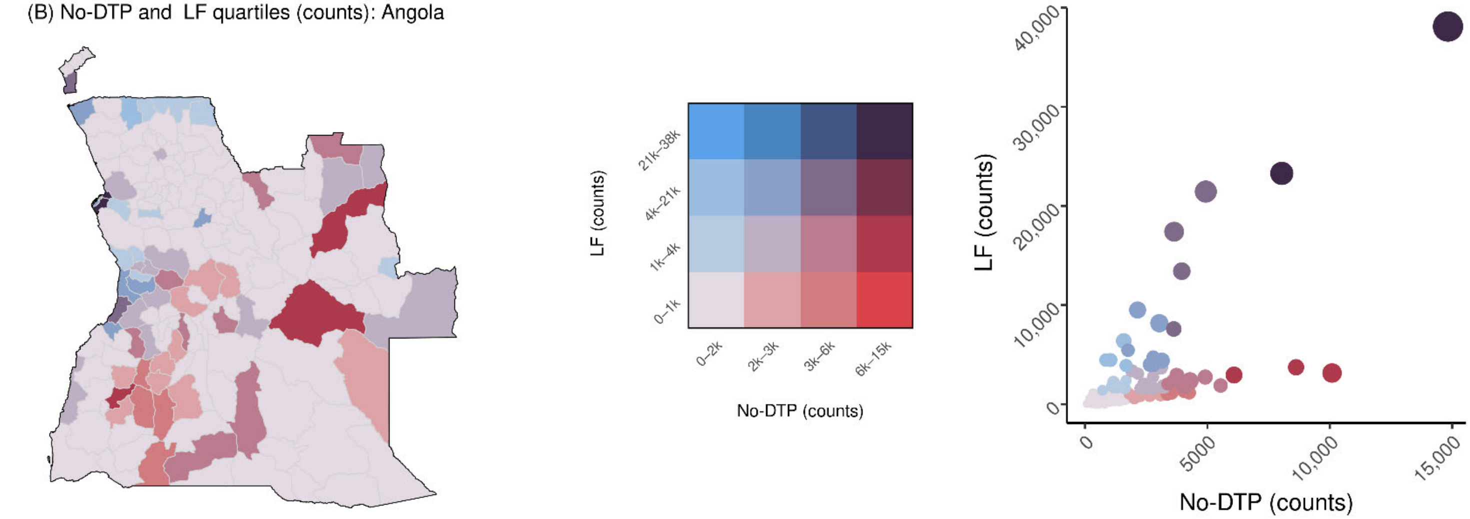

Supplemental Figure S33: DRC, stunting

(A) No-DTP and children with stunting quartiles (%): D.R.C.

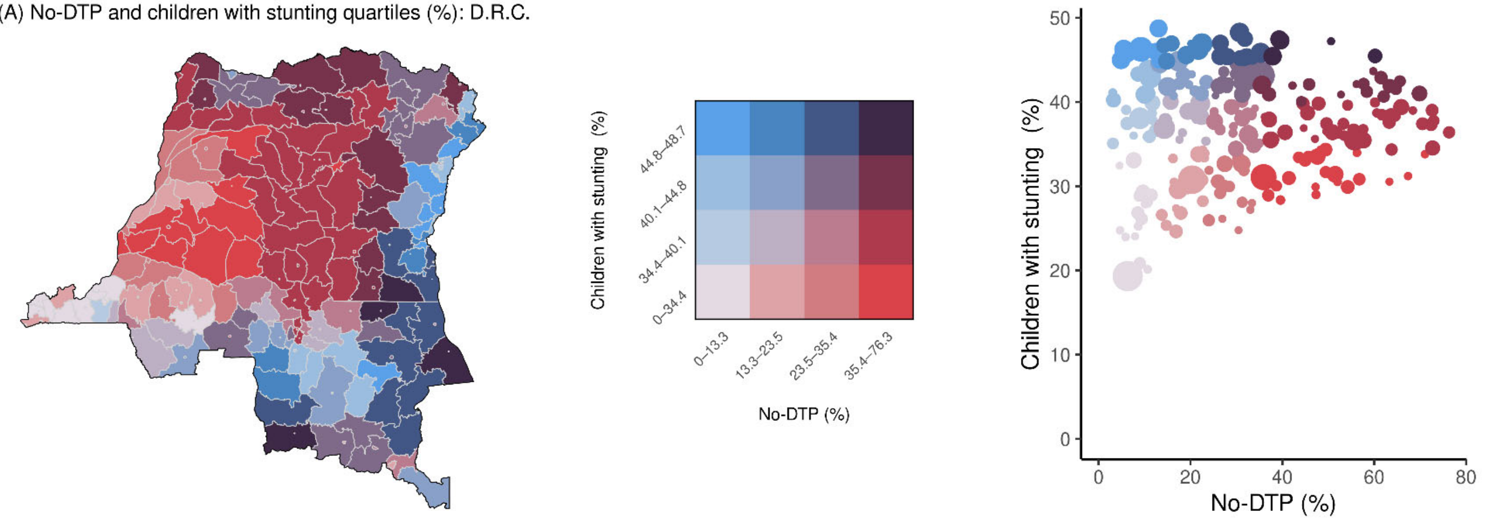

(B) No-DTP and children with stunting quartiles (counts): D.R.C.

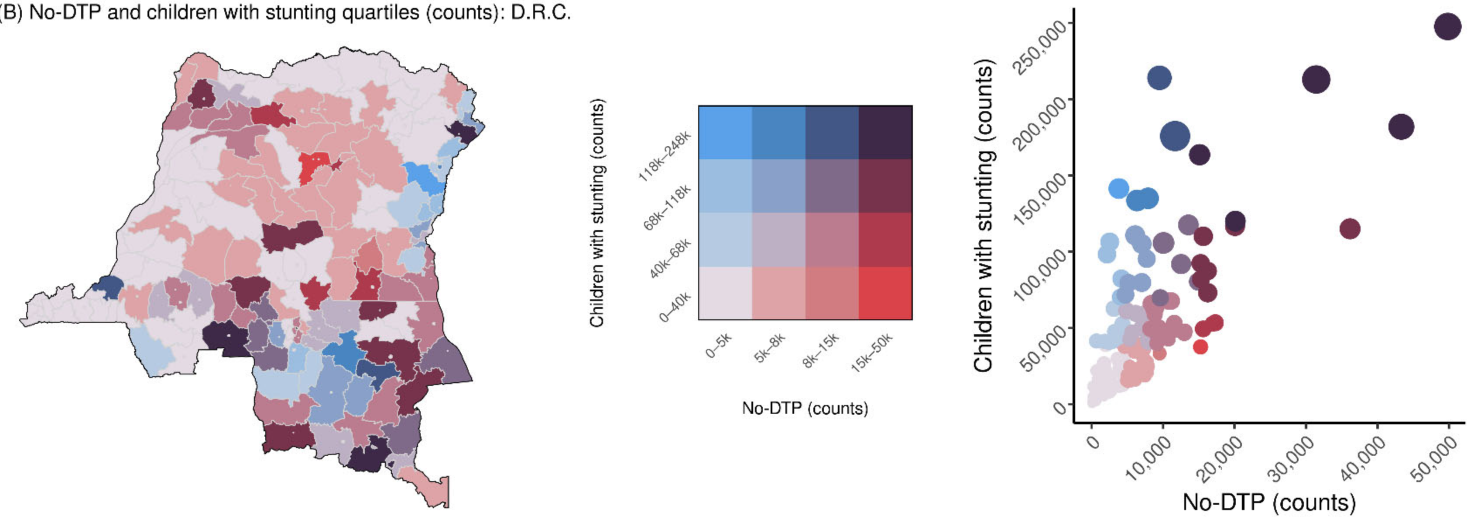

Supplemental Figure S34: DRC, U5M

(A) No-DTP and under-5 mortality quartiles (%): D.R.C.

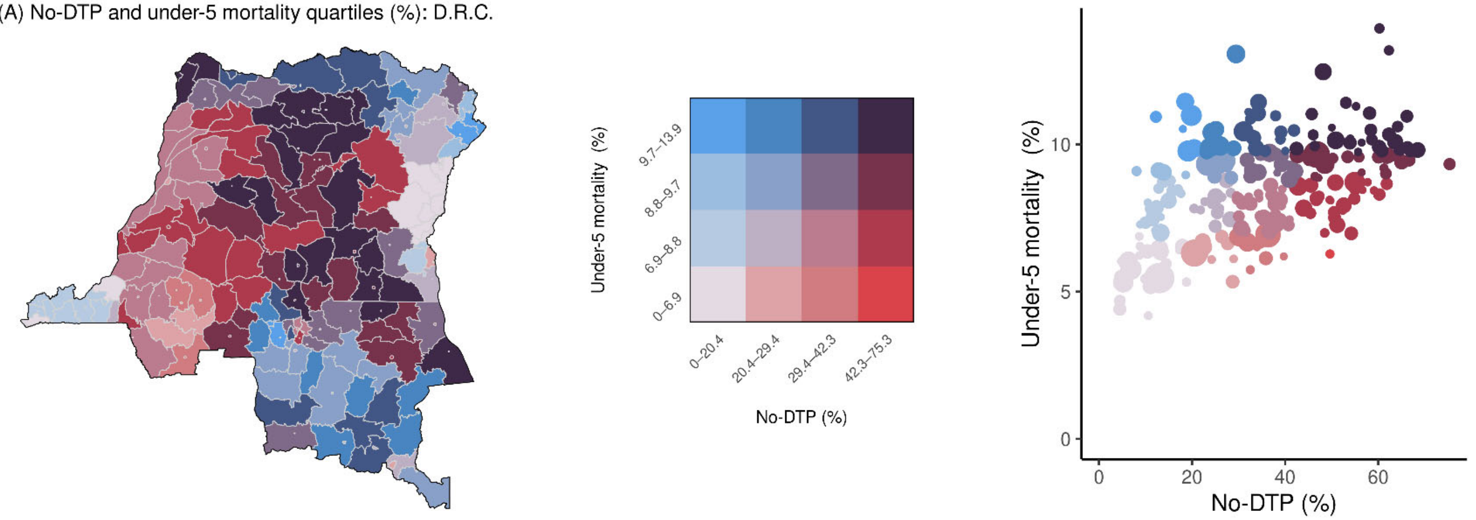

(B) No-DTP and under-5 mortality quartiles (counts): D.R.C.

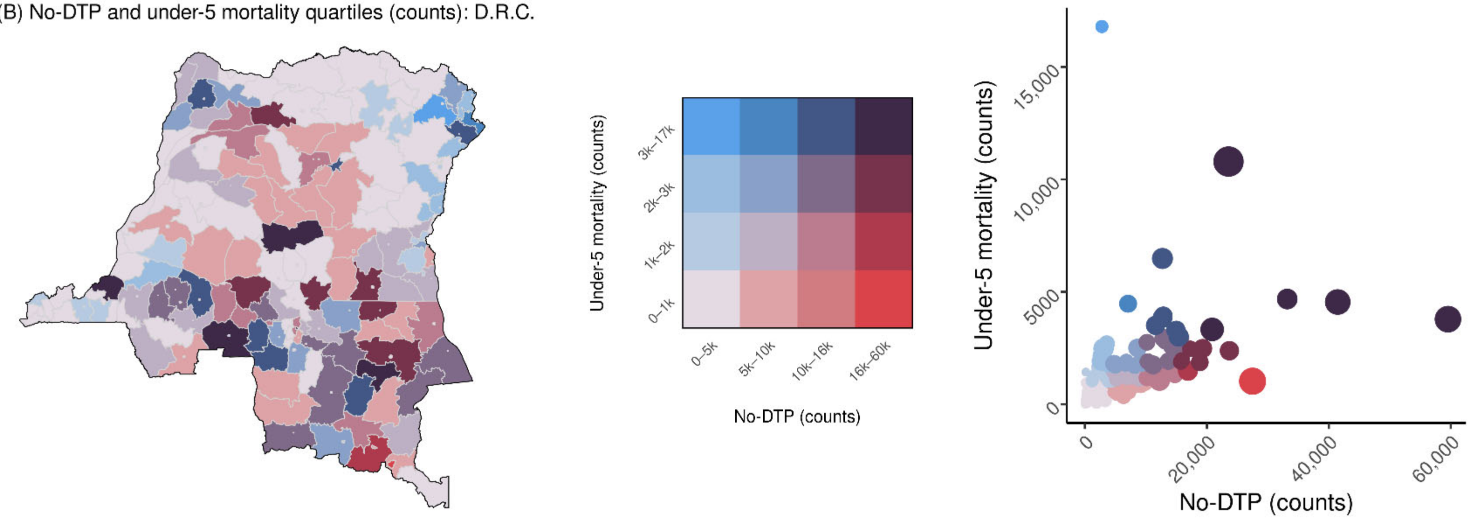

Supplemental Figure S35: DRC, missed ITNs

(A) No-DTP and missed ITNs quartiles (%): D.R.C.

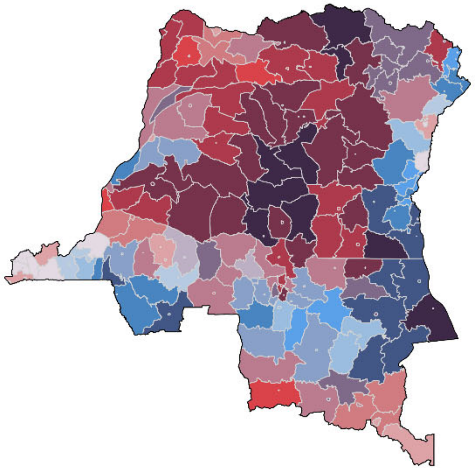

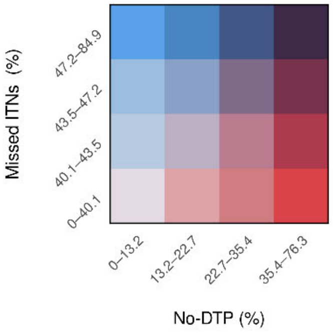

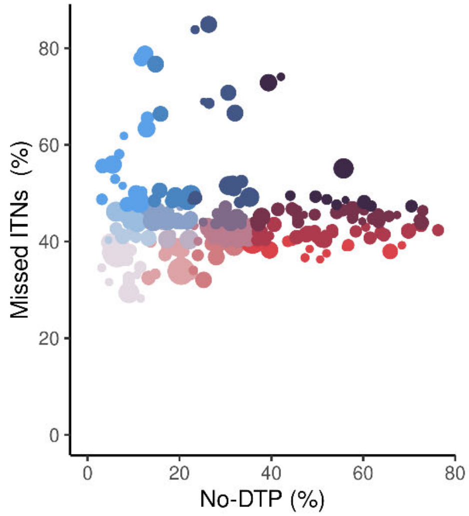

(B) No-DTP and missed ITNs quartiles (counts): D.R.C.

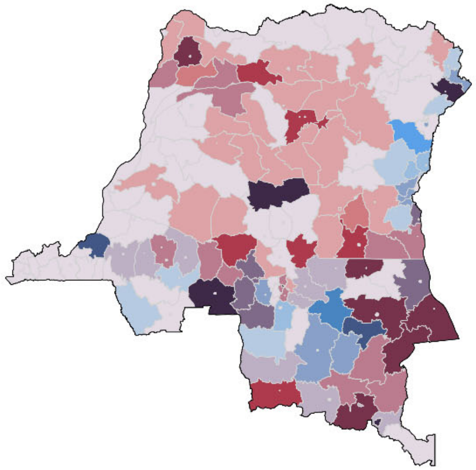

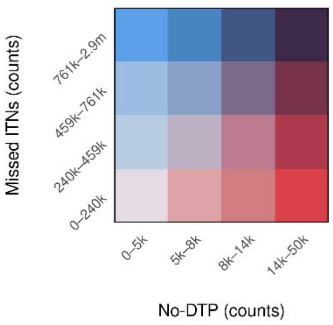

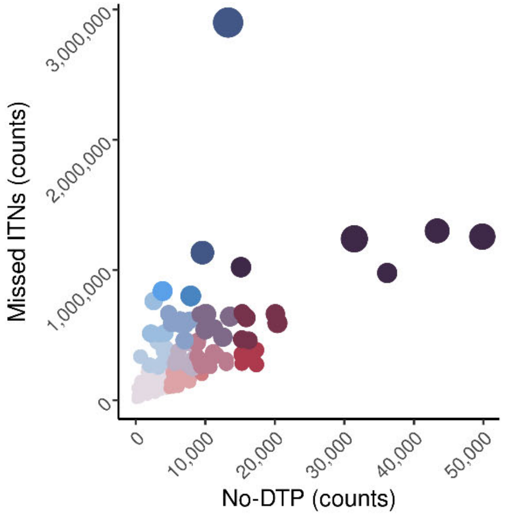

Supplemental Figure S36: DRC, missed ORT

(A) No-DTP and missed ORT quartiles (%): D.R.C.

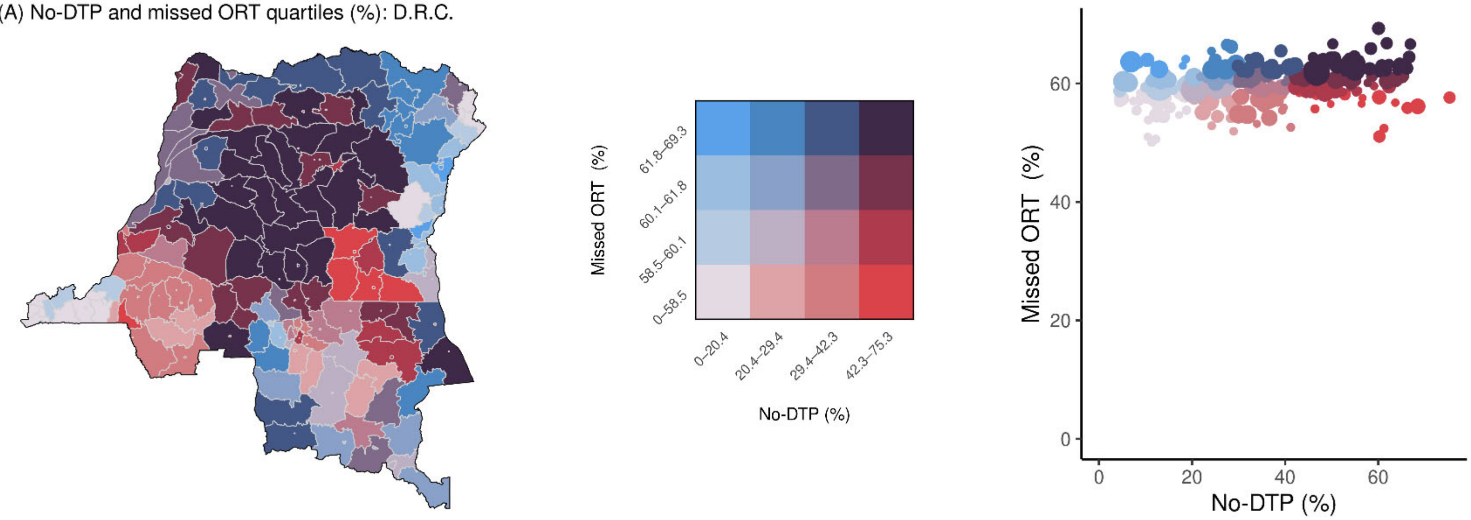

(B) No-DTP and missed ORT quartiles (counts): D.R.C.

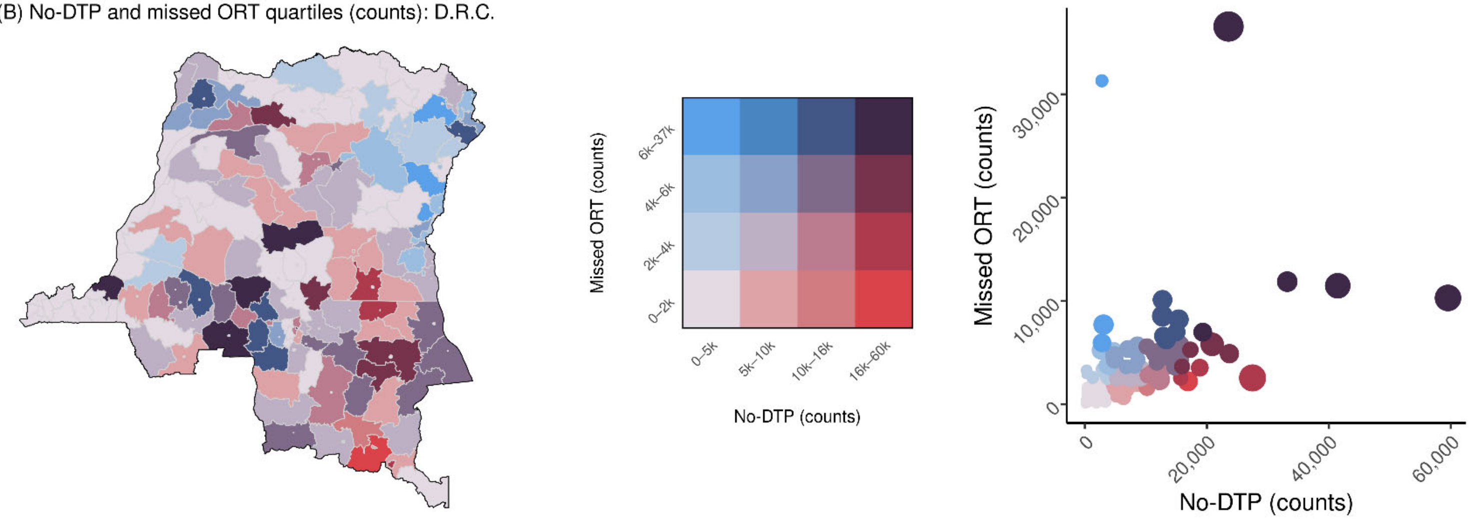

Supplemental Figure S37: DRC, LF

(A) No-DTP and LF quartiles (%): D.R.C.

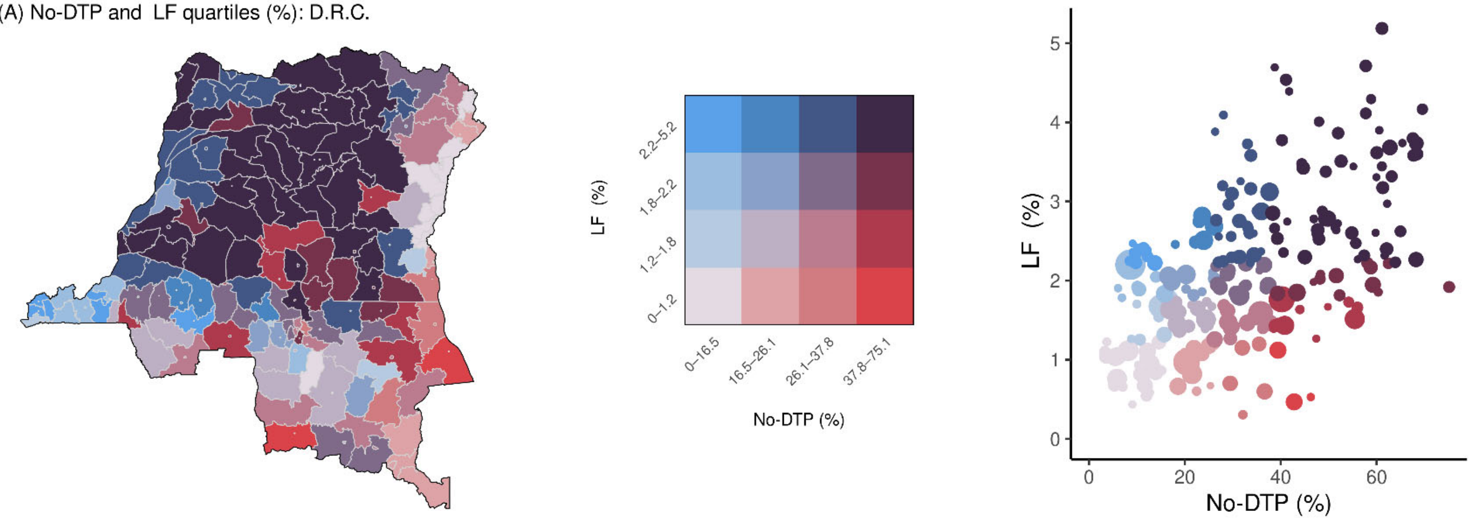

(B) No-DTP and LF quartiles (counts): D.R.C.

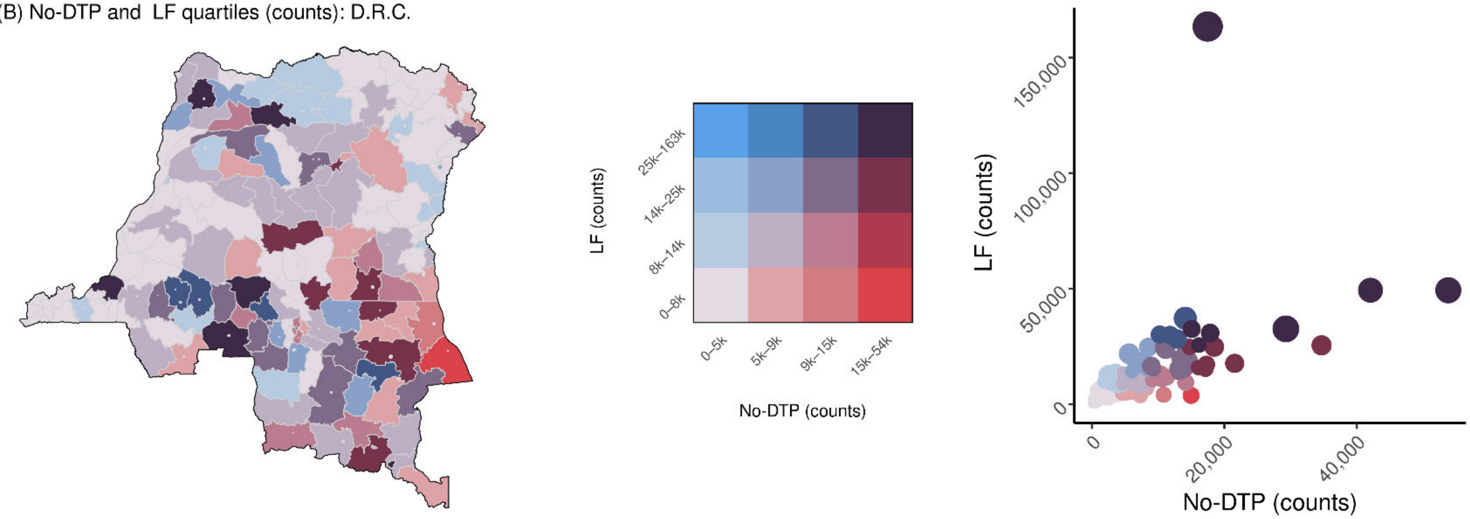

Supplemental Figure S38: Ethiopia, stunting

(A) No-DTP and children with stunting quartiles (%): Ethiopia

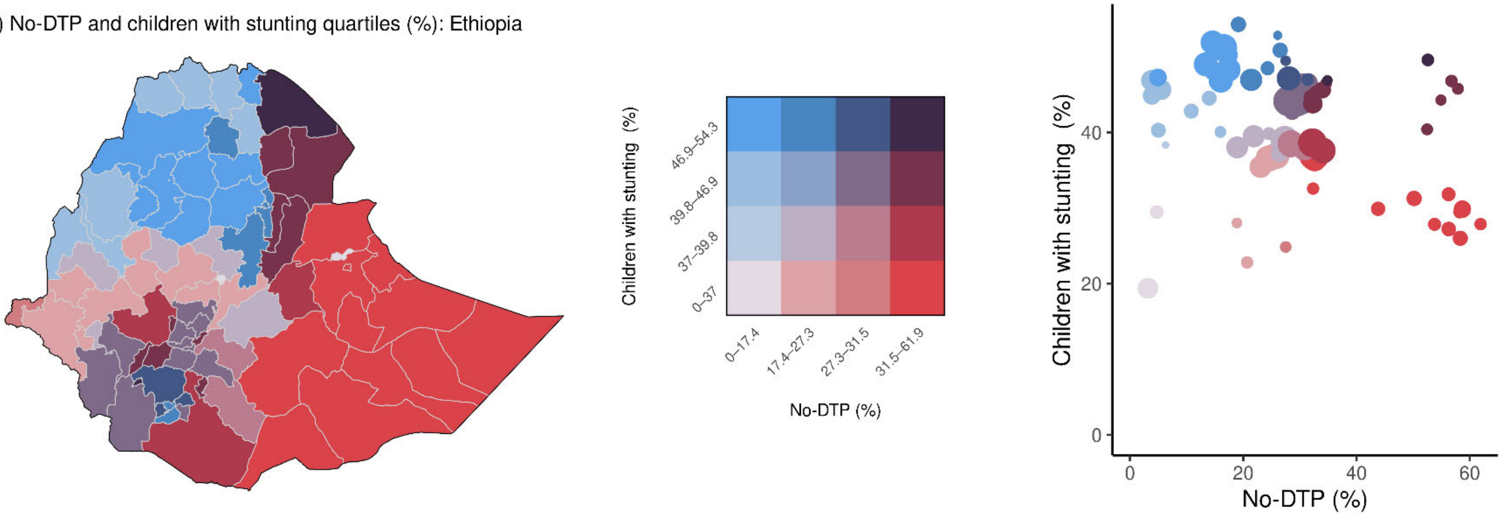

(B) No-DTP and children with stunting quartiles (counts): Ethiopia

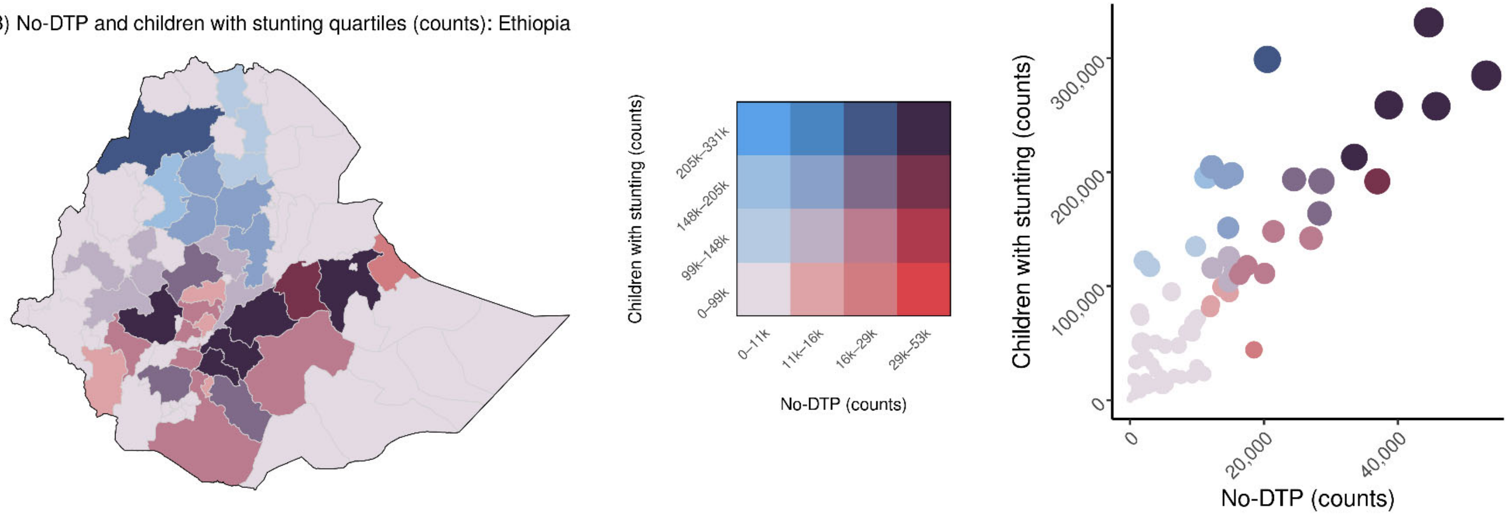

Supplemental Figure S39: Ethiopia, U5M

(A) No-DTP and under-5 mortality quartiles (%): Ethiopia

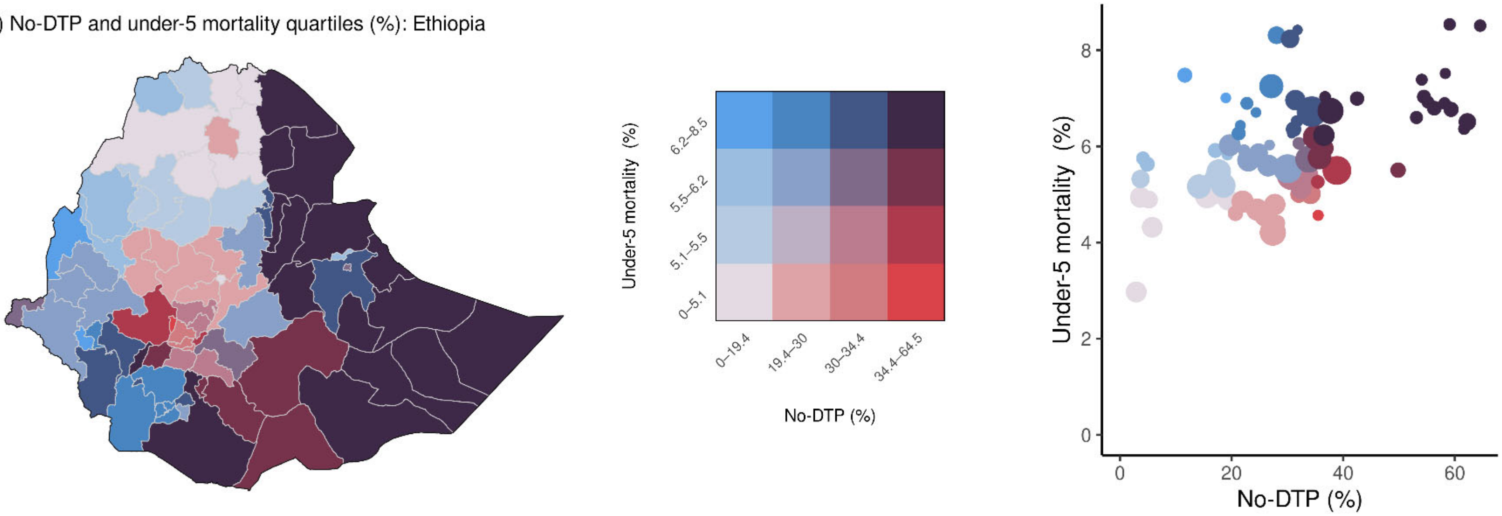

(B) No-DTP and under-5 mortality quartiles (counts): Ethiopia

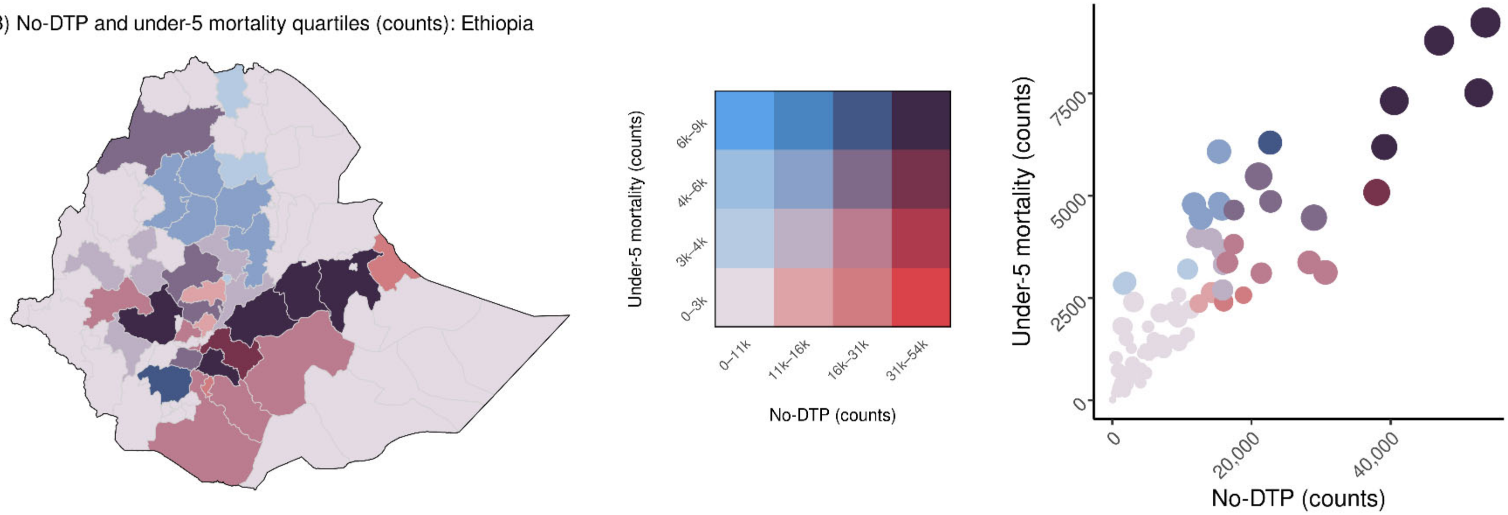

Supplemental Figure S40: Ethiopia, missed ITNs

(A) No-DTP and missed ITNs quartiles (%): Ethiopia

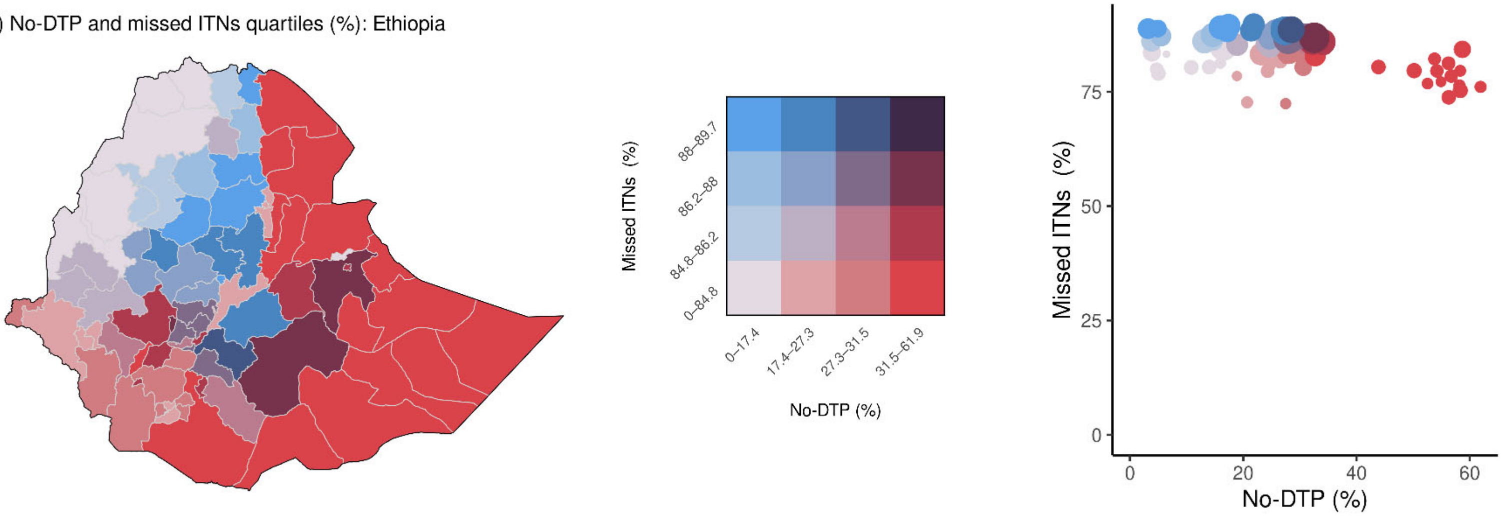

(B) No-DTP and missed ITNs quartiles (counts): Ethiopia

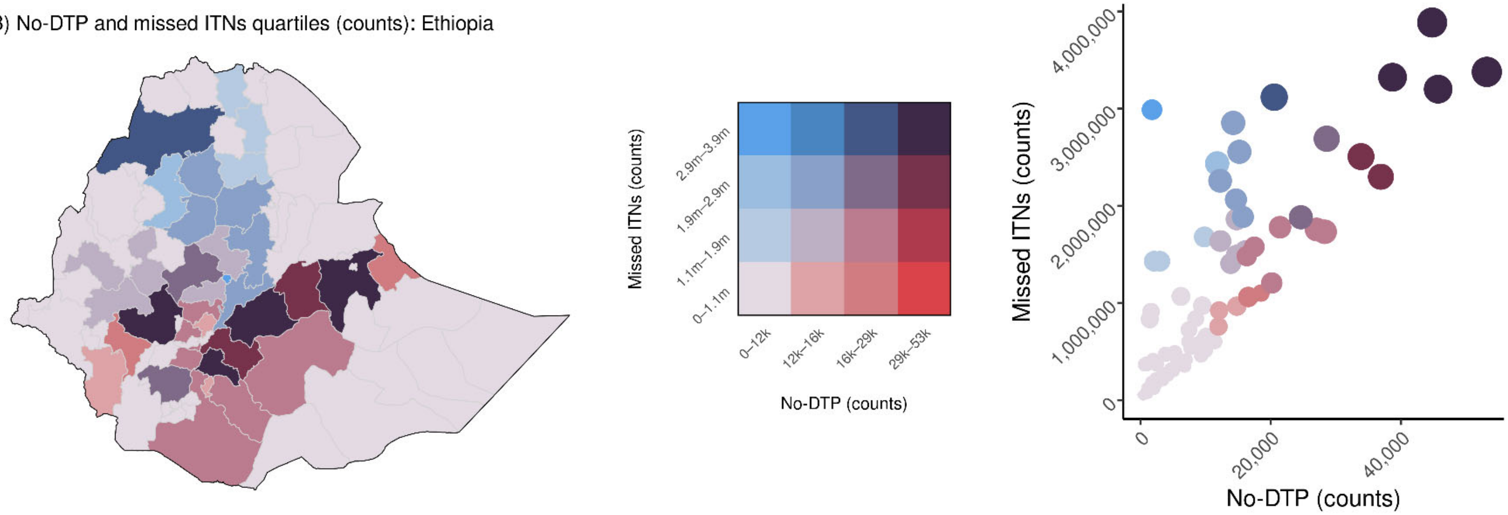

Supplemental Figure S41: Ethiopia, missed ORT

(A) No-DTP and missed ORT quartiles (%): Ethiopia

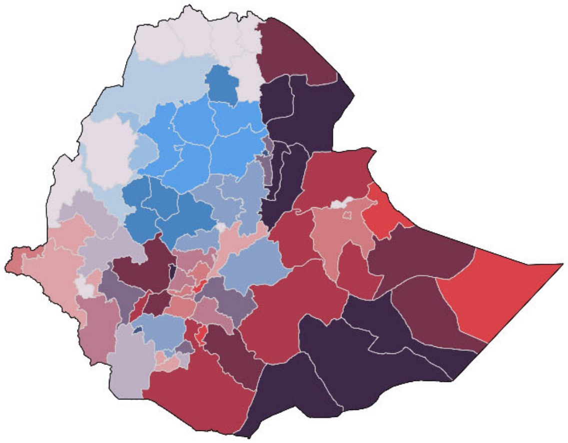

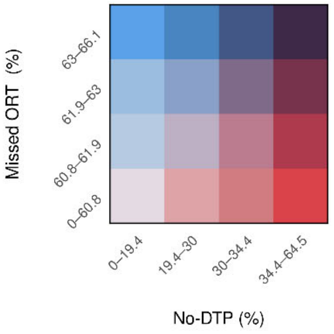

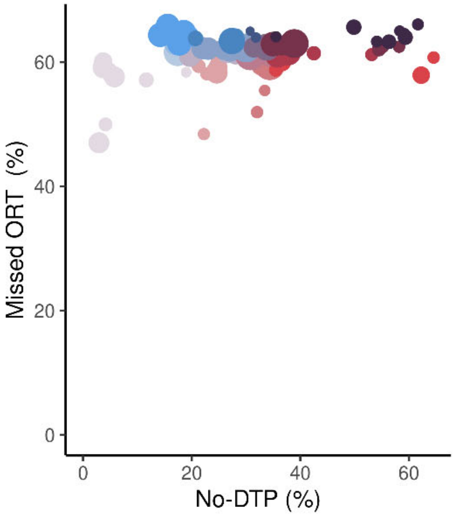

(B) No-DTP and missed ORT quartiles (counts): Ethiopia

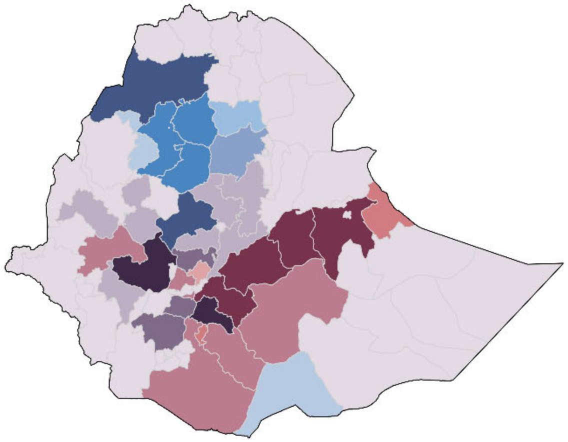

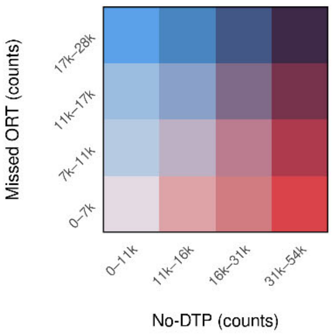

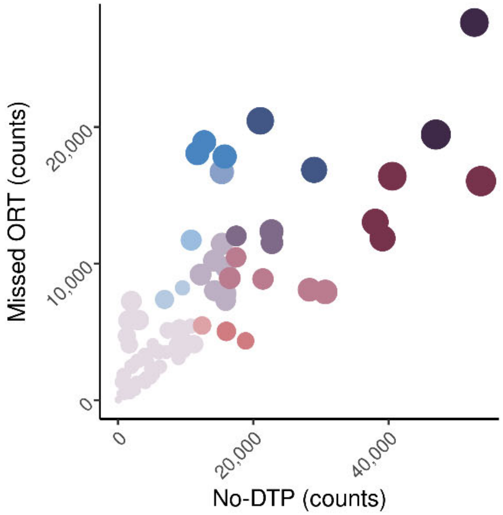

Supplemental Figure S42: Ethiopia, LF

(A) No-DTP and LF quartiles (%): Ethiopia

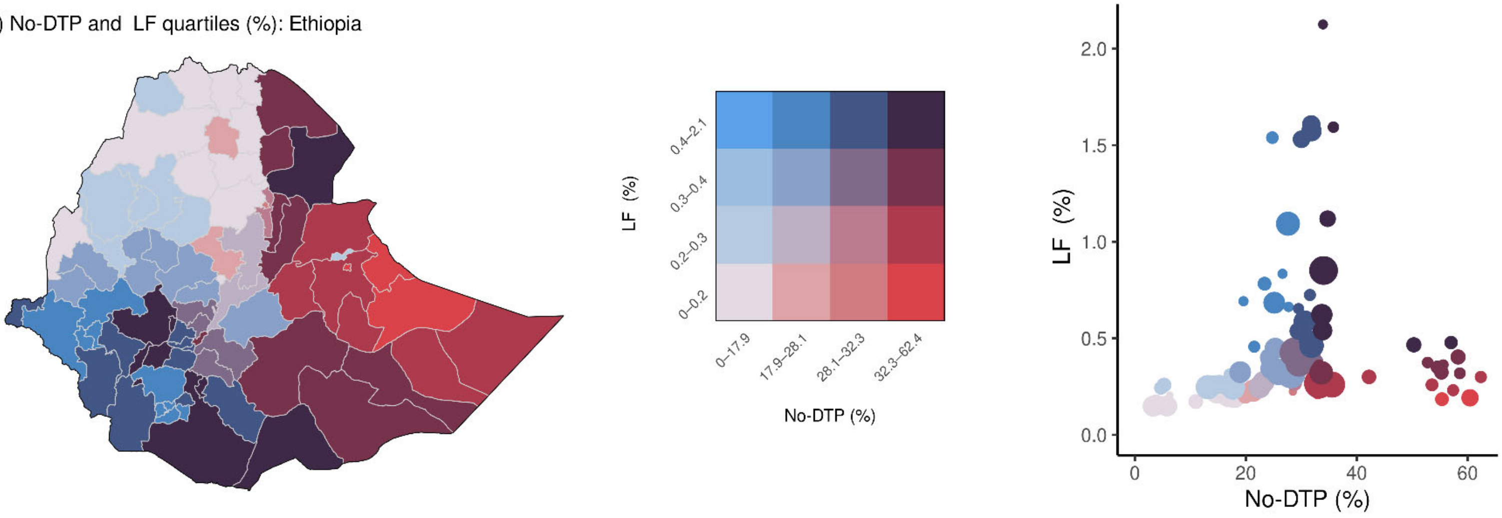

(B) No-DTP and LF quartiles (counts): Ethiopia

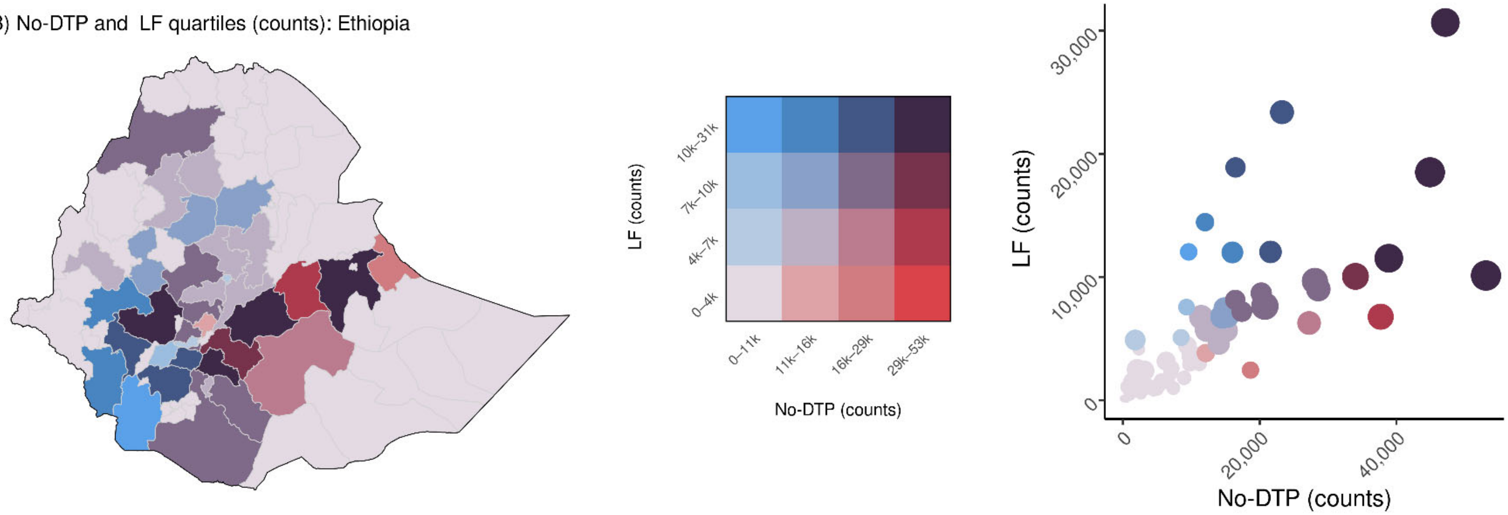

Supplemental Figure S43: Indonesia, stunting

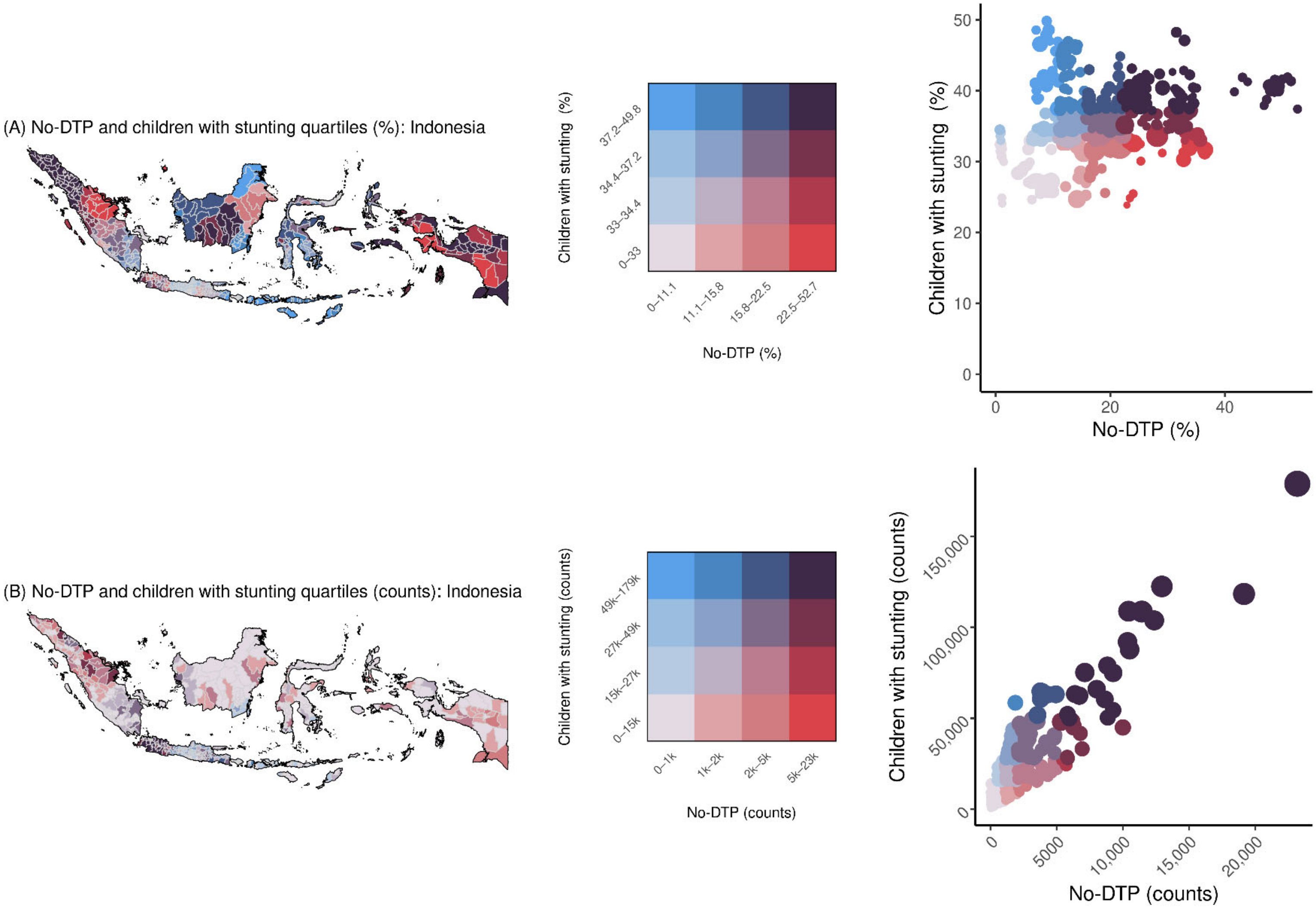

Supplemental Figure S44: Indonesia, U5M

(A) No-DTP and under-5 mortality quartiles (%): Indonesia

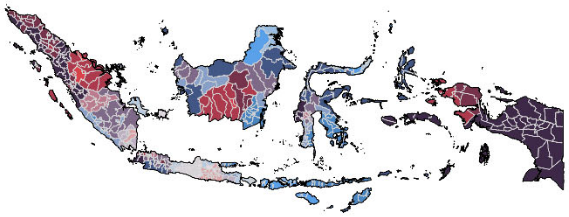

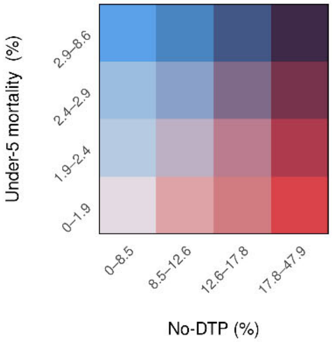

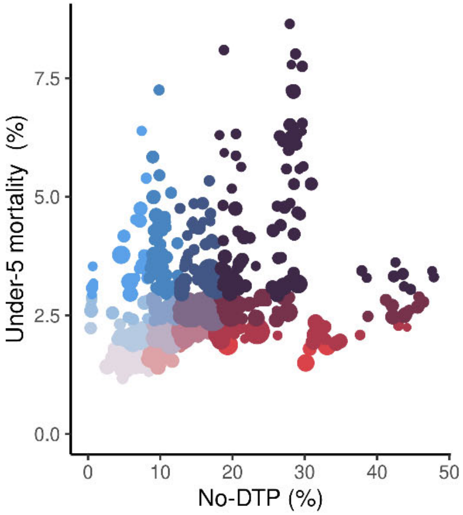

(B) No-DTP and under-5 mortality quartiles (counts): Indonesia

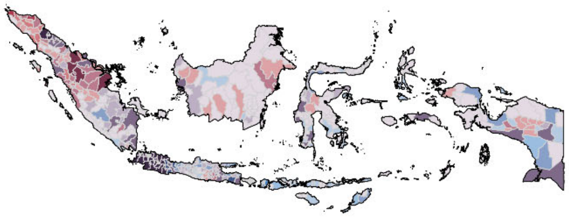

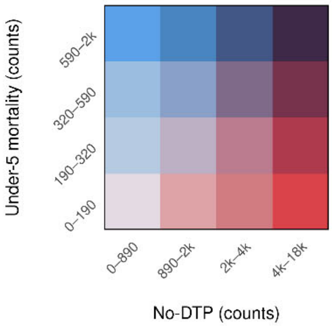

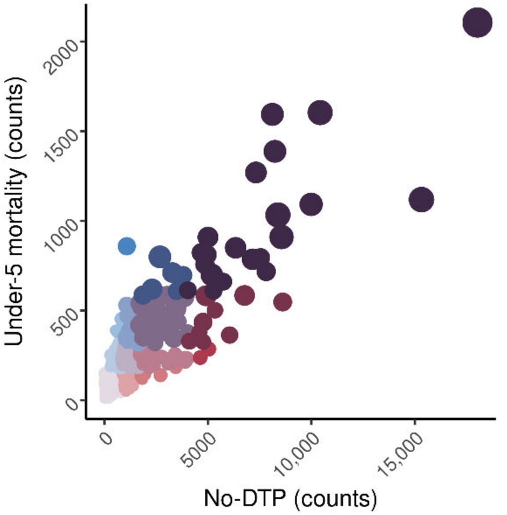

Supplemental Figure S45: Indonesia, missed ORT

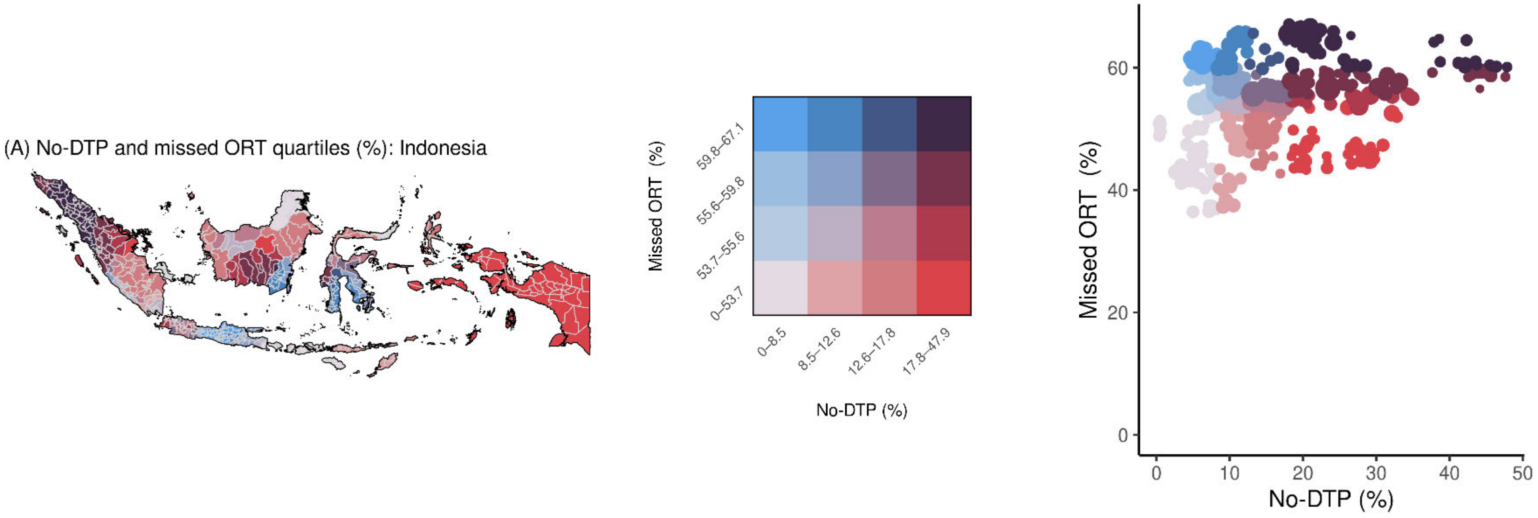

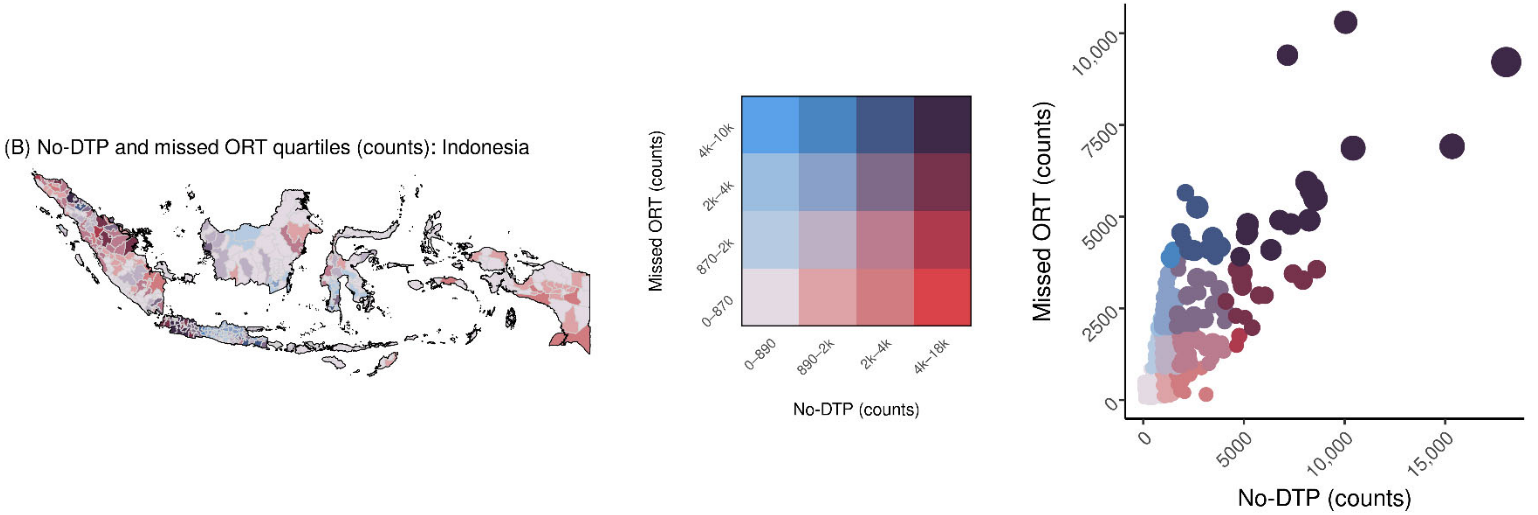

Supplemental Figure S46: Indonesia, LF

(A) No-DTP and LF quartiles (%): Indonesia

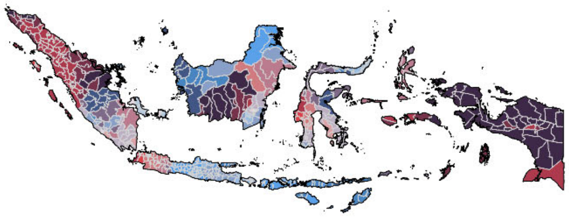

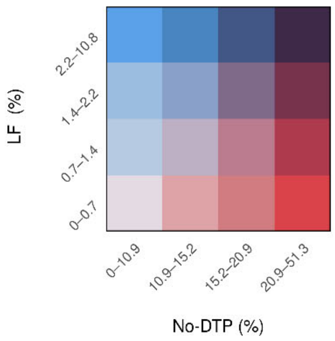

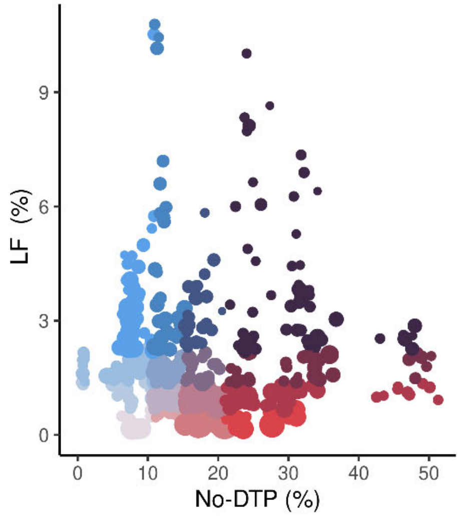

(B) No-DTP and LF quartiles (counts): Indonesia

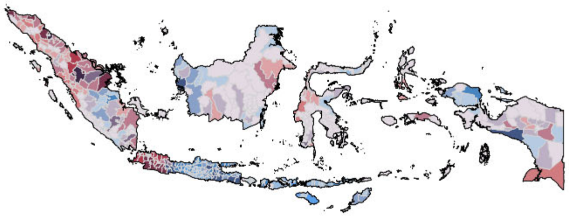

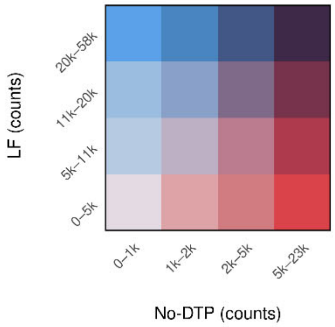

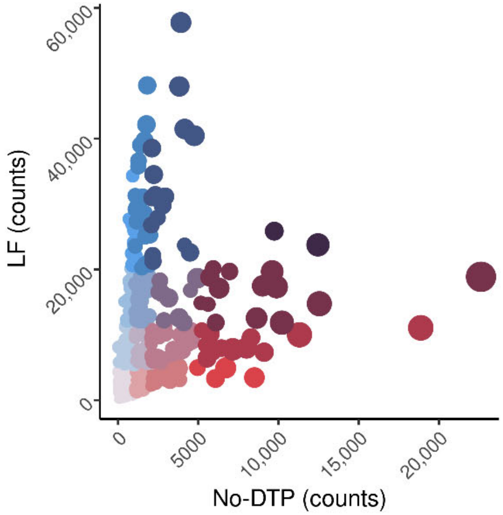

Supplemental Figures S47–S69: Country-specific, highest quartile overlap.

Country-specific overlap between no-DTP and each indicator (children with stunting, U5M, missed ITNs, missed ORT, LF), for each country (Nigeria, Angola, DRC, Ethiopia and Indonesia), for the highest quartiles only. Districts in red are in the highest quartile for no-DTP only, blue are in the highest quartile for the comparator indicator only, and purple are in the highest quartile for both indicators. The map on the left (a) shows categorization based on prevalence, and the map on the right (b) shows categorization based on counts.

Supplemental Figure S47: Nigeria, U5M

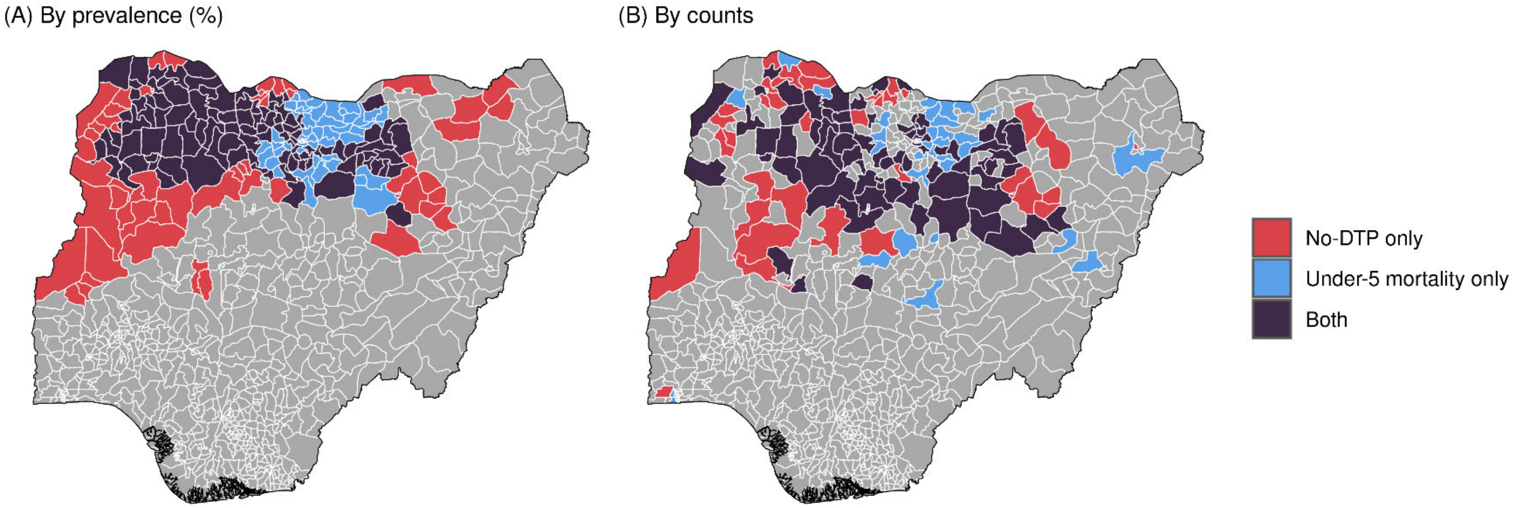

Supplemental Figure S48: Nigeria, missed ITNs

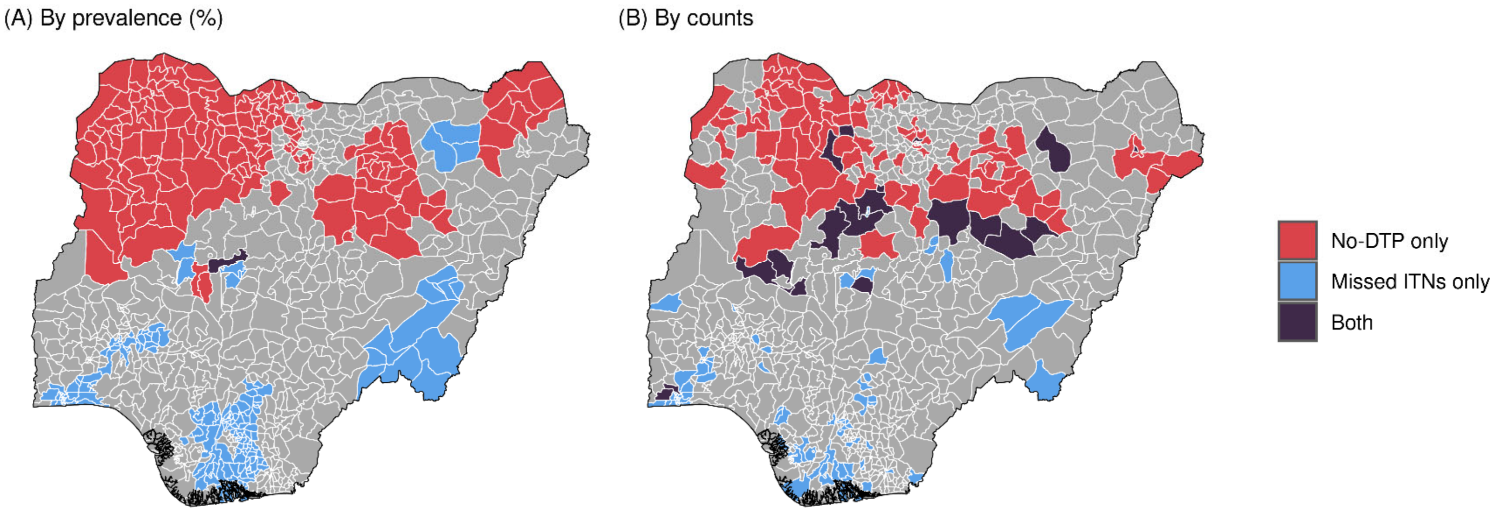

Supplemental Figure S49: Nigeria, missed ORT

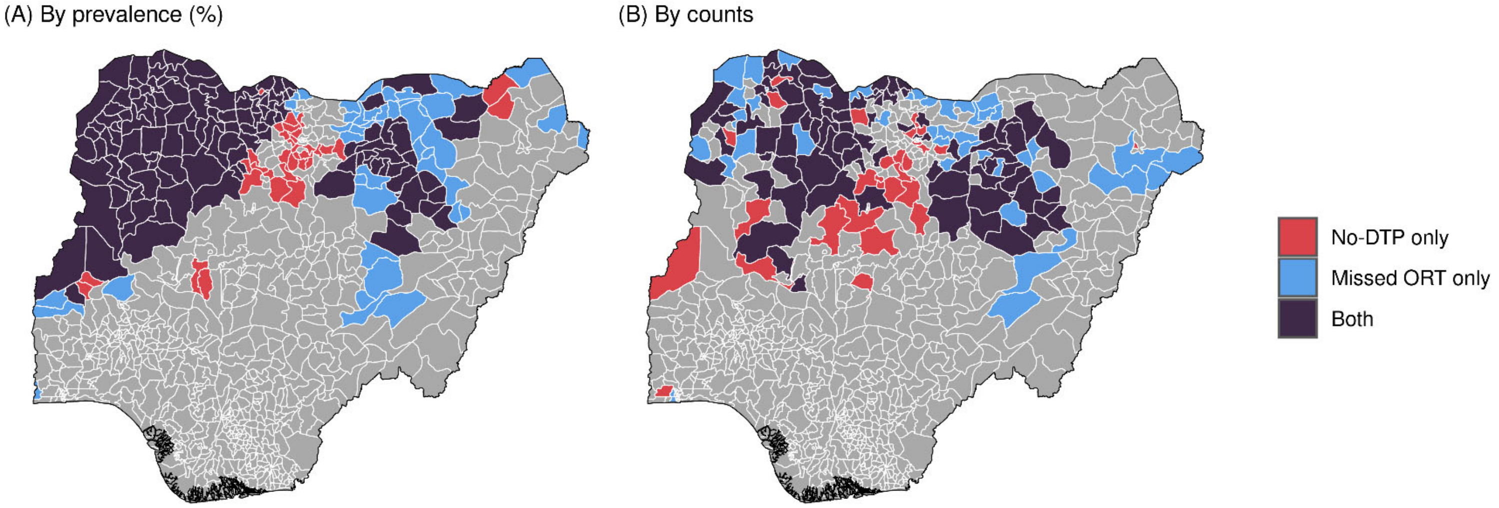

Supplemental Figure S50: Nigeria, LF

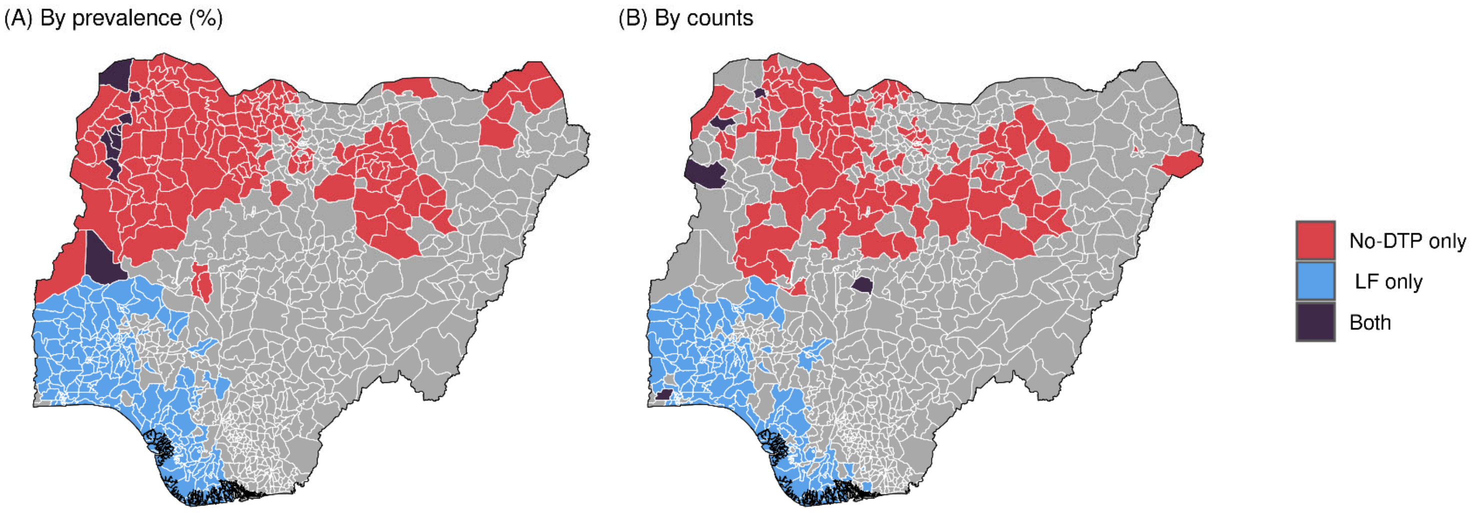

Supplemental Figure S51: Angola, stunting

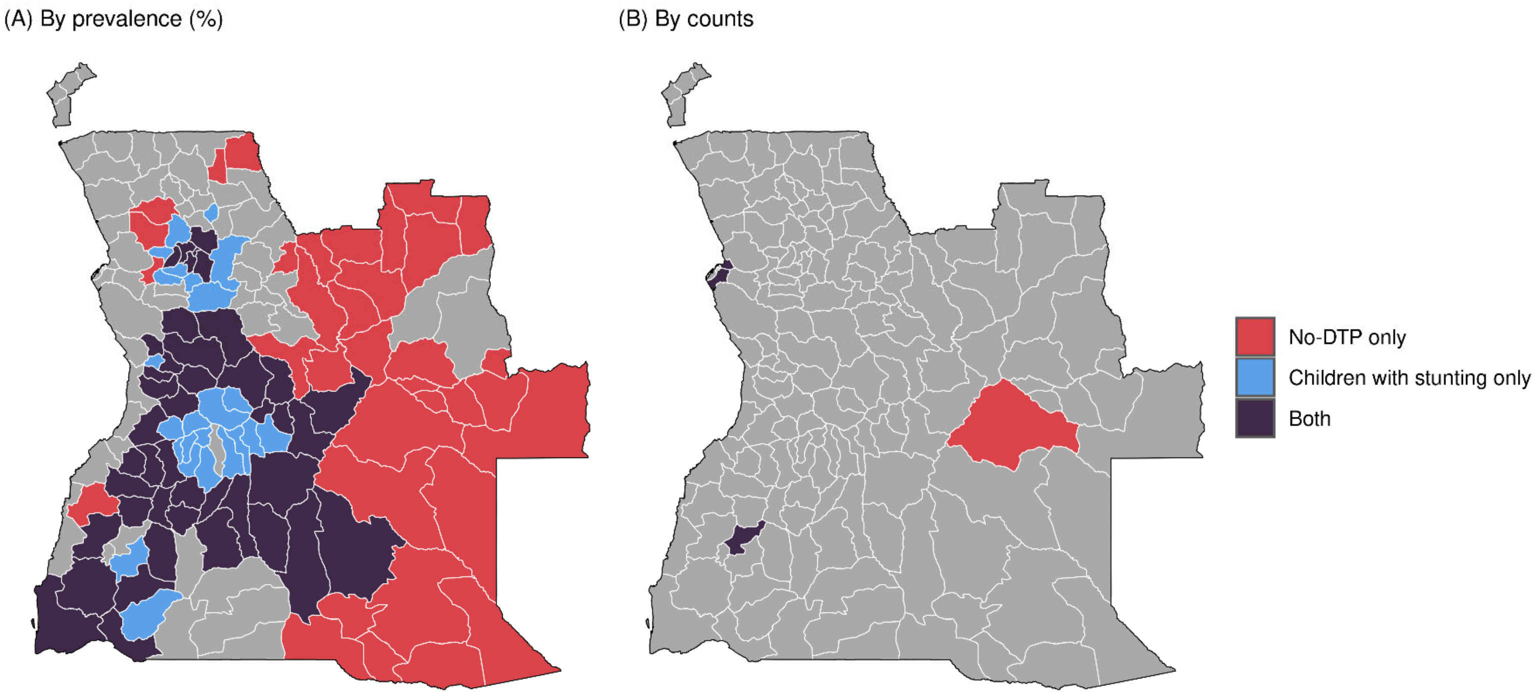

Supplemental Figure S52: Angola, U5M

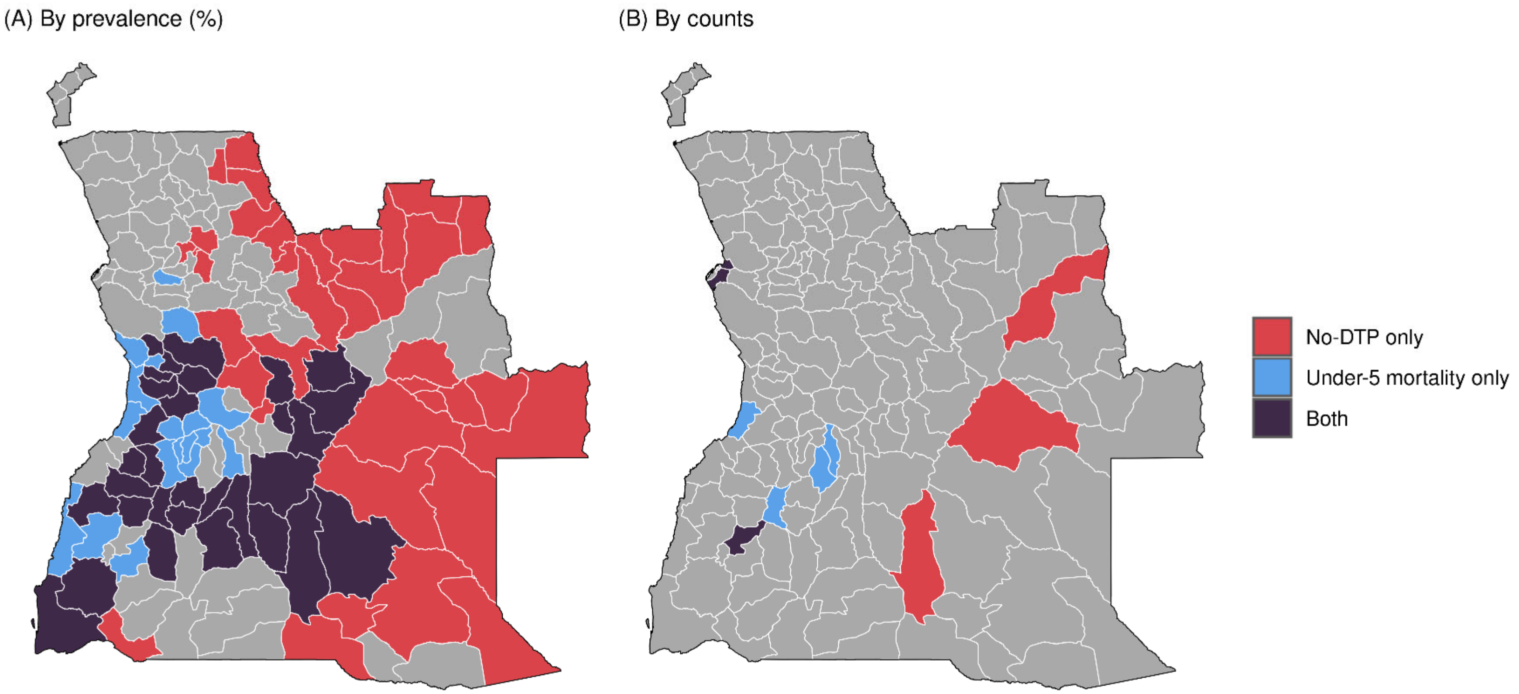

Supplemental Figure S53: Angola, missed ITNs

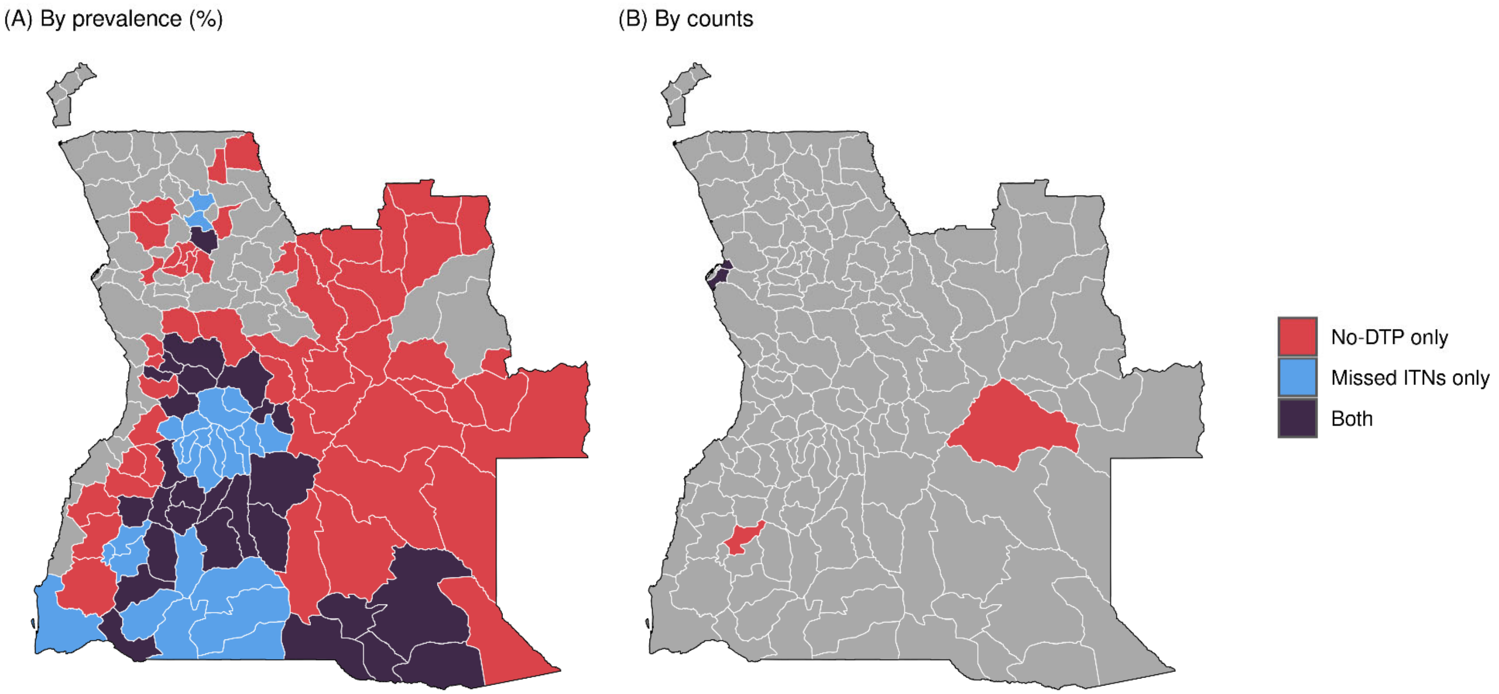

Supplemental Figure S54: Angola, missed ORT

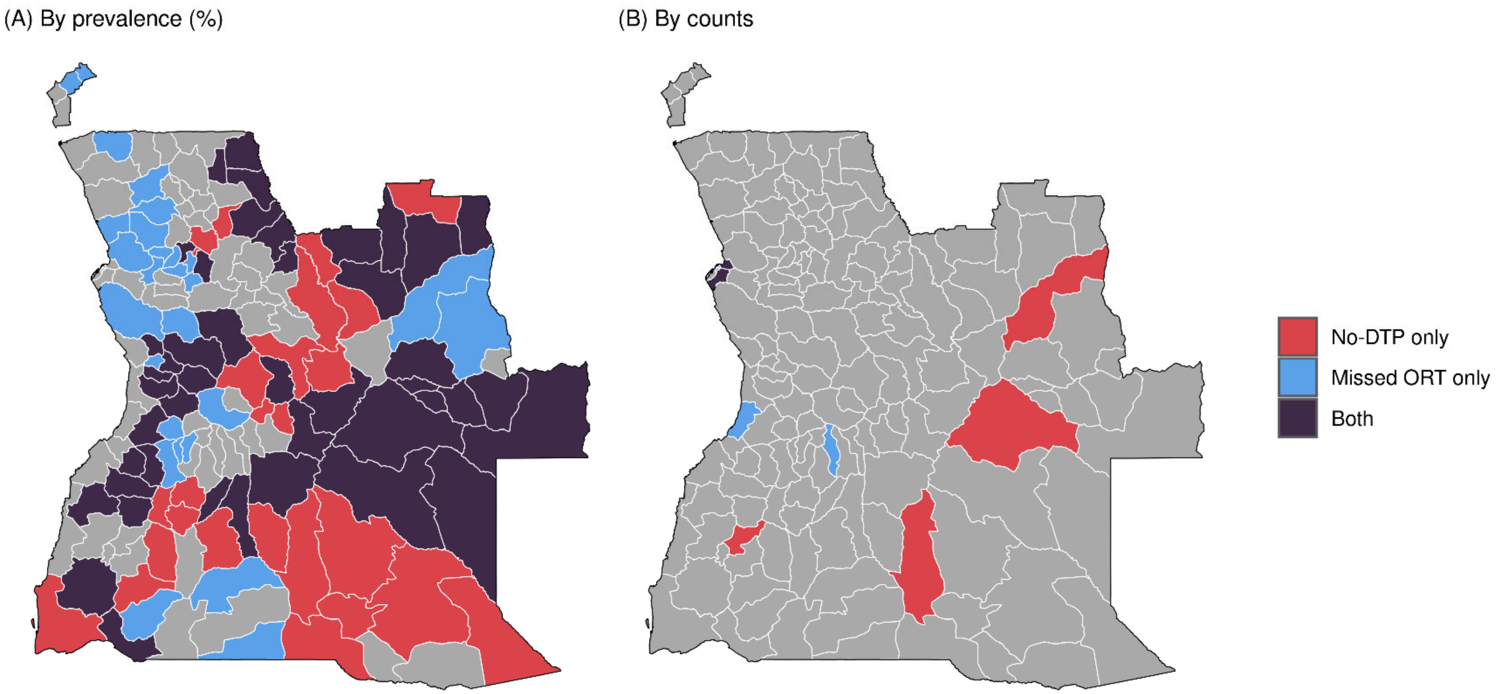

Supplemental Figure S55: Angola, LF

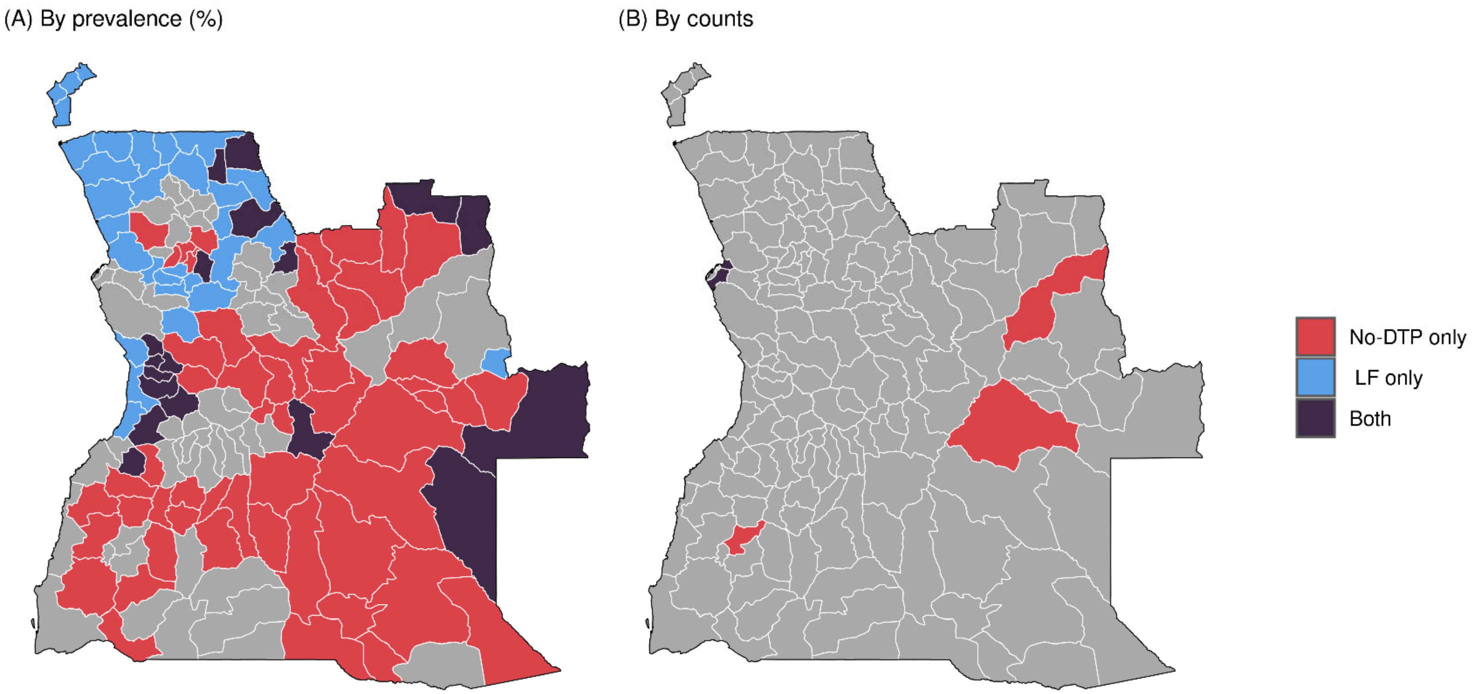

Supplemental Figure S56: DRC, stunting

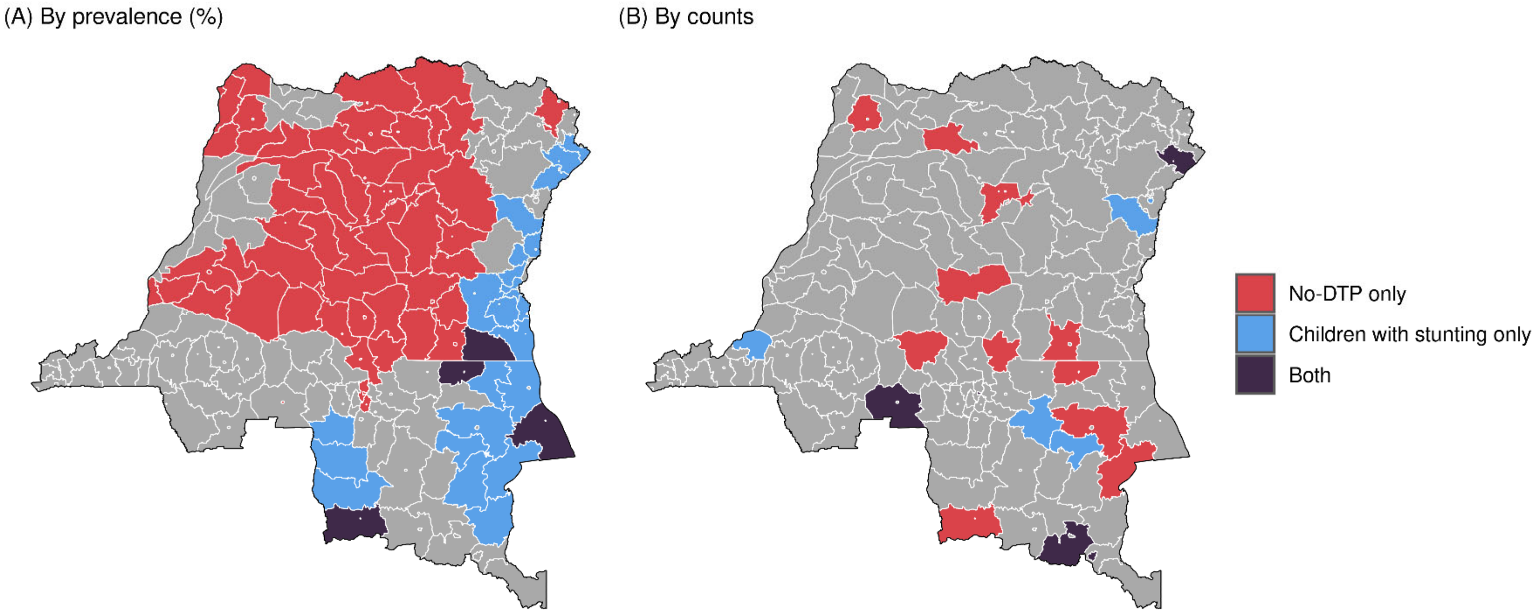

Supplemental Figure S57: DRC, U5M

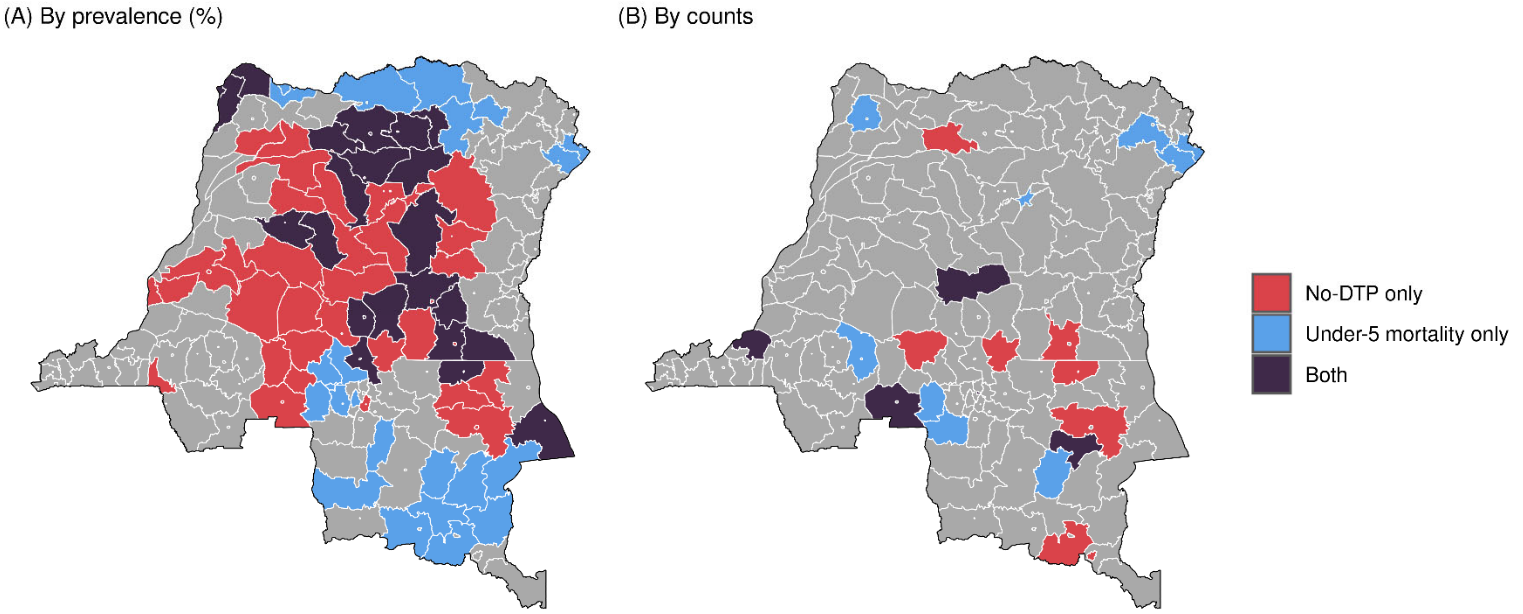

Supplemental Figure S58: DRC, missed ITNs

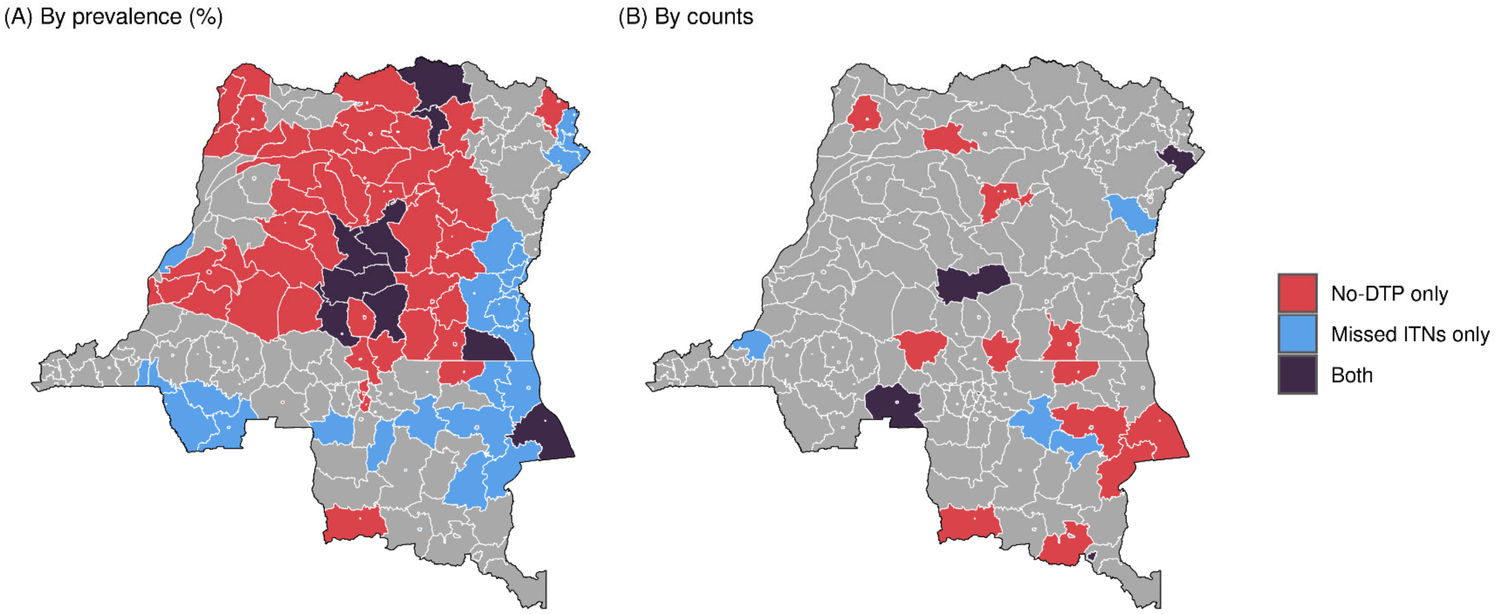

Supplemental Figure S59: DRC, missed ORT

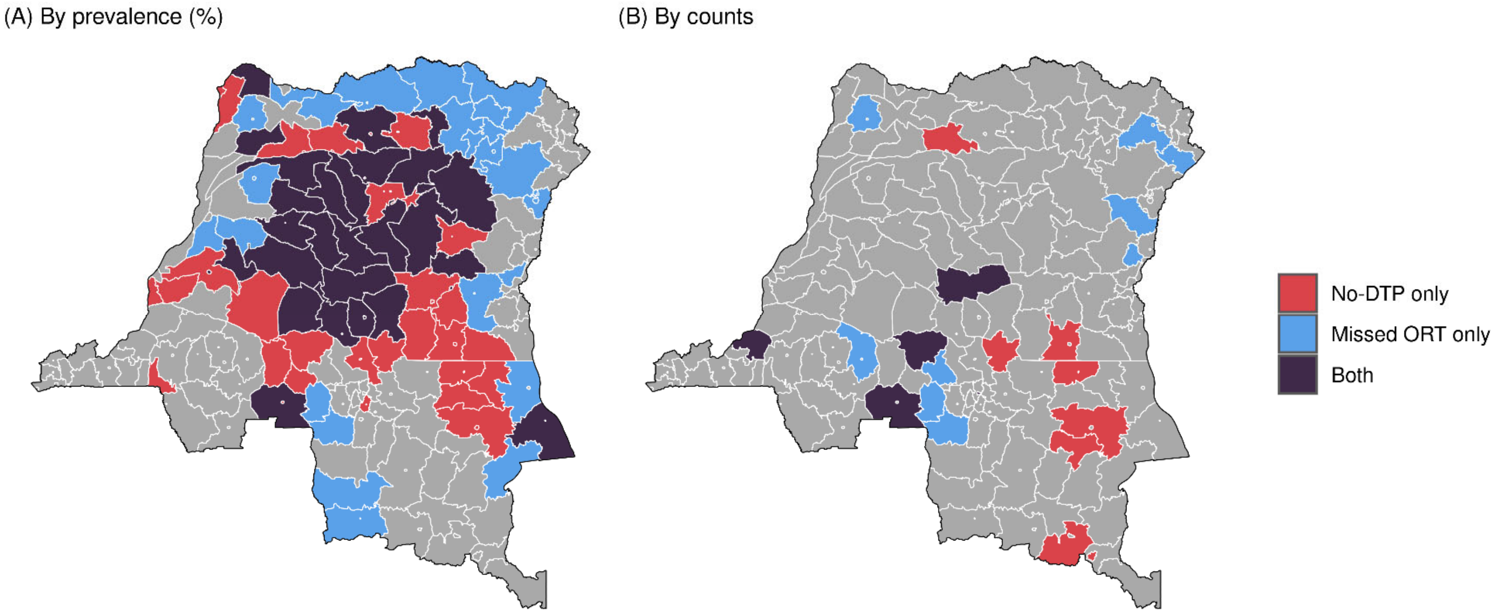

Supplemental Figure S60: DRC, LF

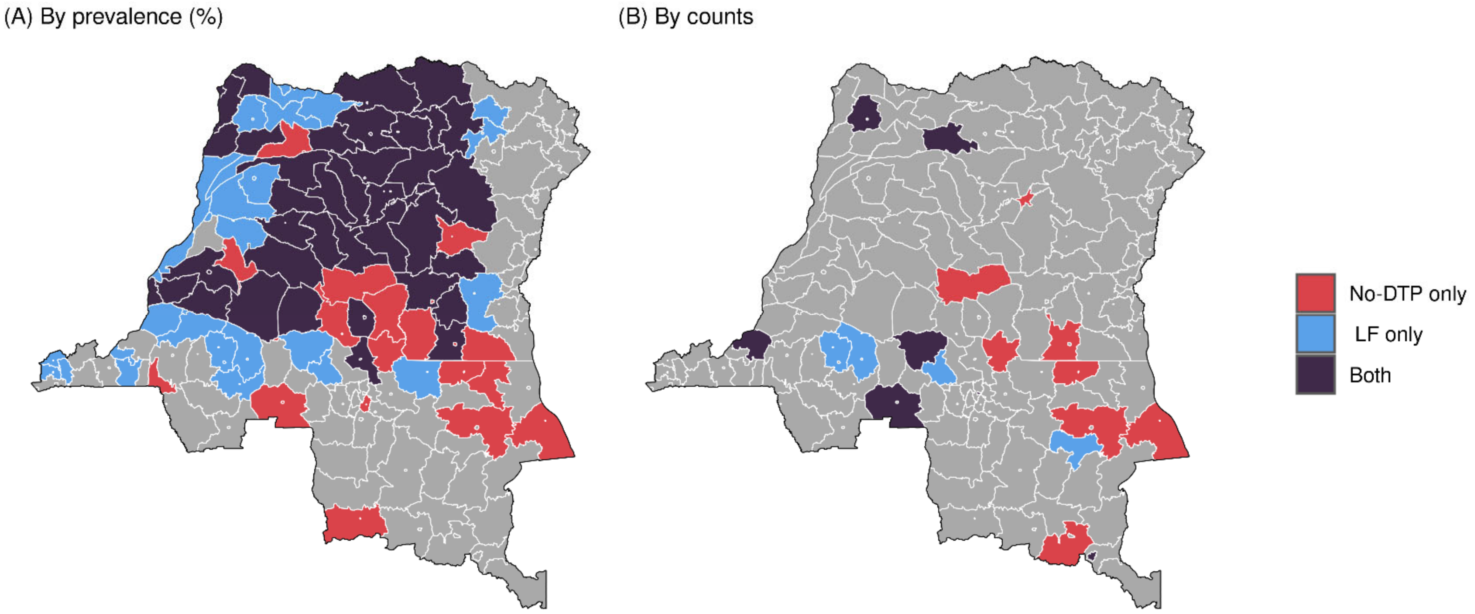

Supplemental Figure S61: Ethiopia, stunting

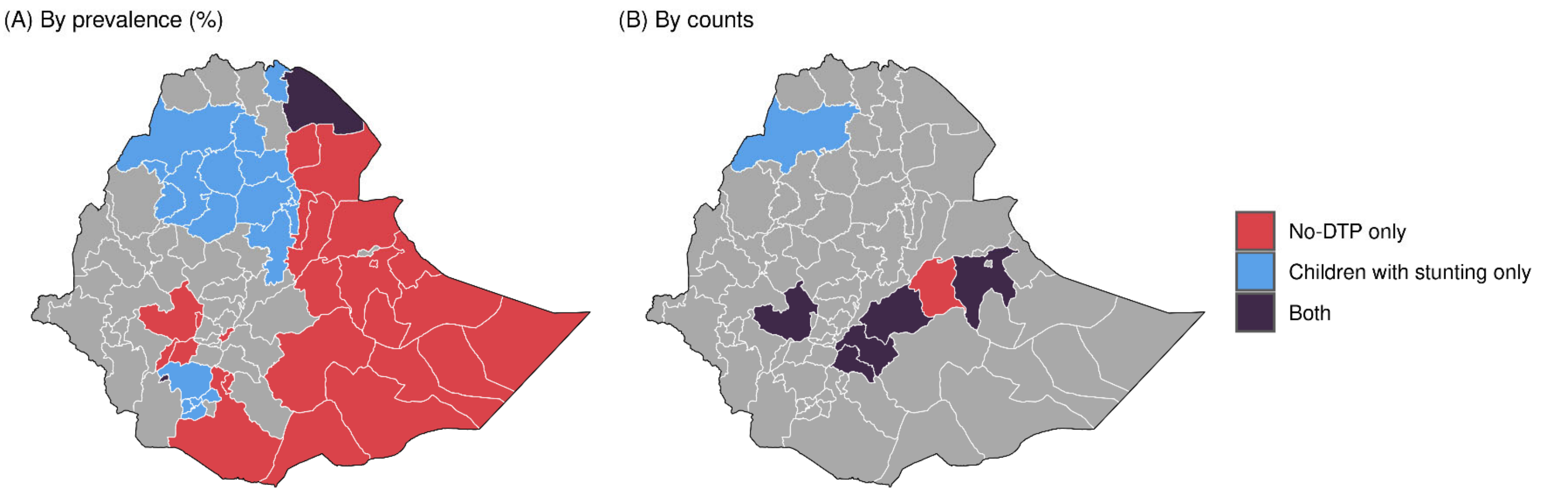

Supplemental Figure S62: Ethiopia, U5M

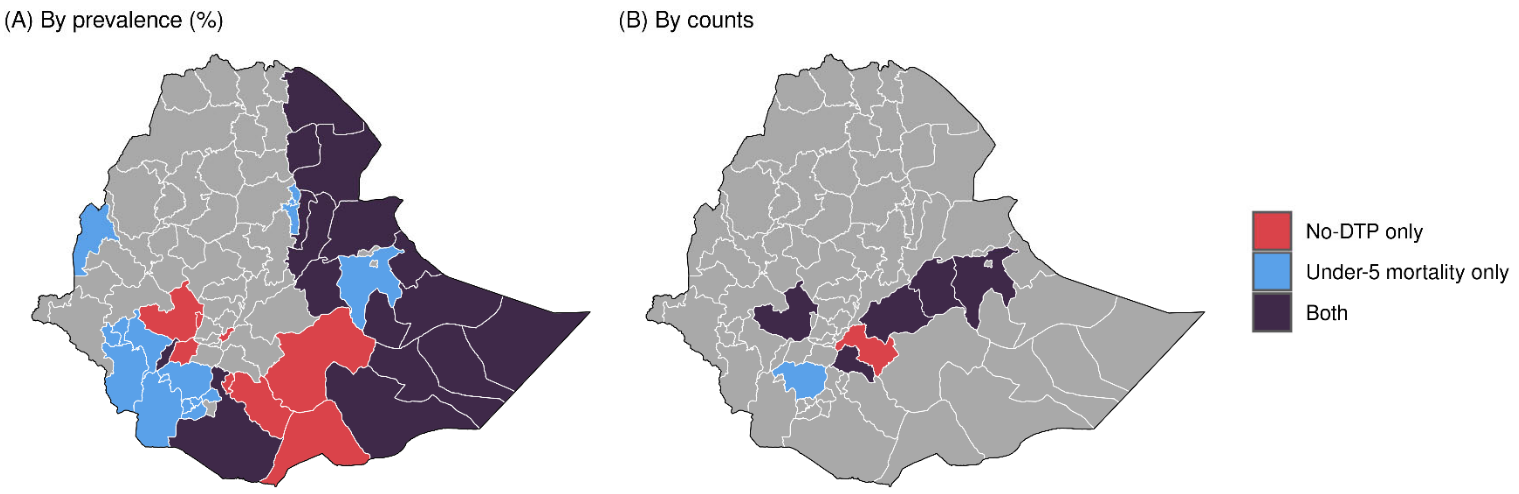

Supplemental Figure S63: Ethiopia, missed ITNs

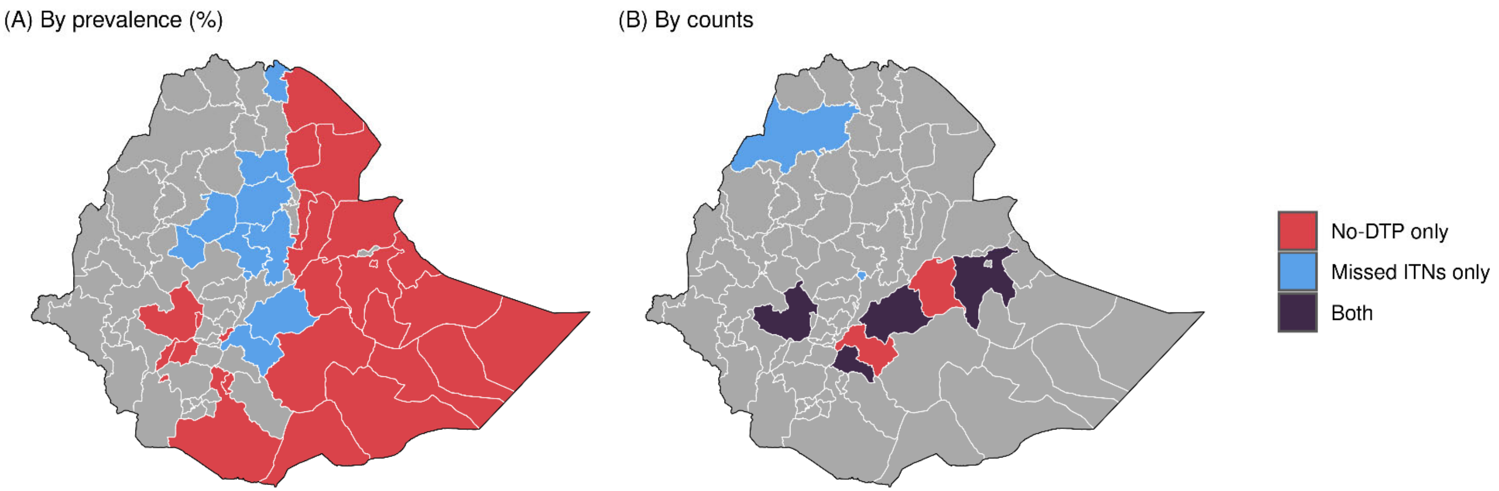

Supplemental Figure S64: Ethiopia, missed ORT

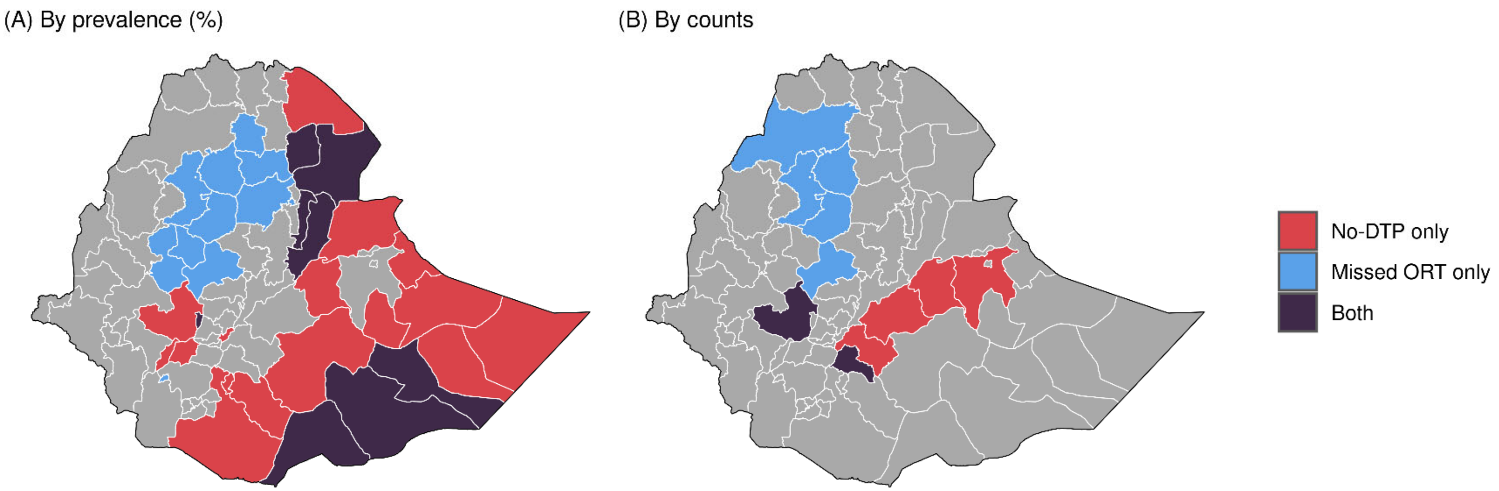

Supplemental Figure S65: Ethiopia, LF

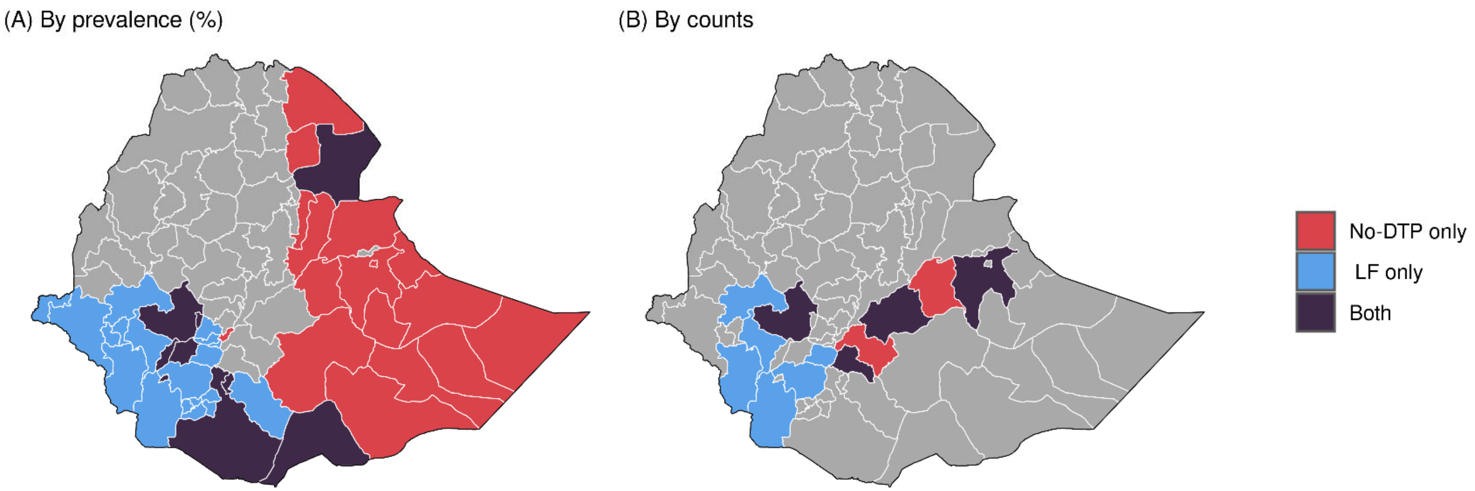

Supplemental Figure S66: Indonesia, stunting

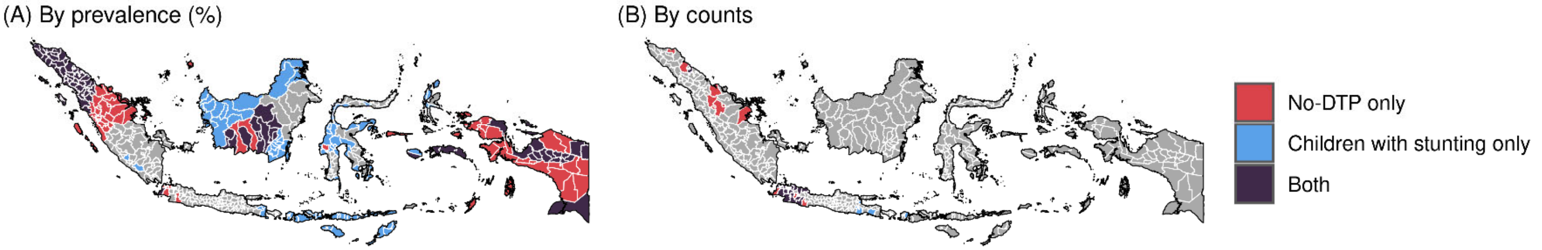

Supplemental Figure S67: Indonesia, U5M

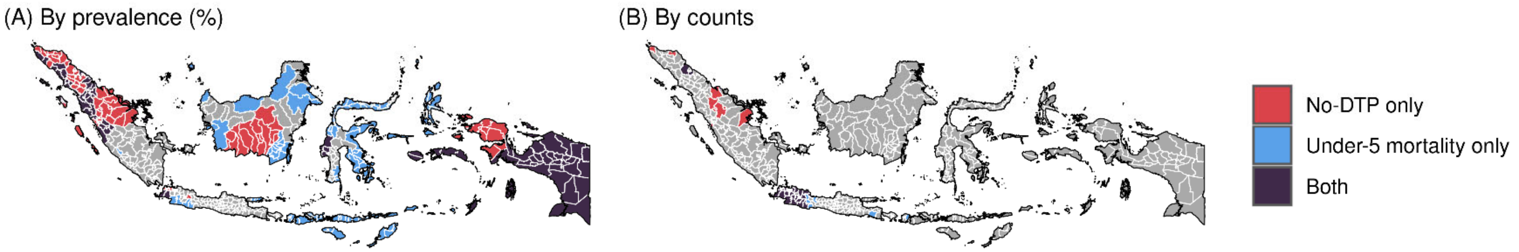

Supplemental Figure S68: Indonesia, missed ORT

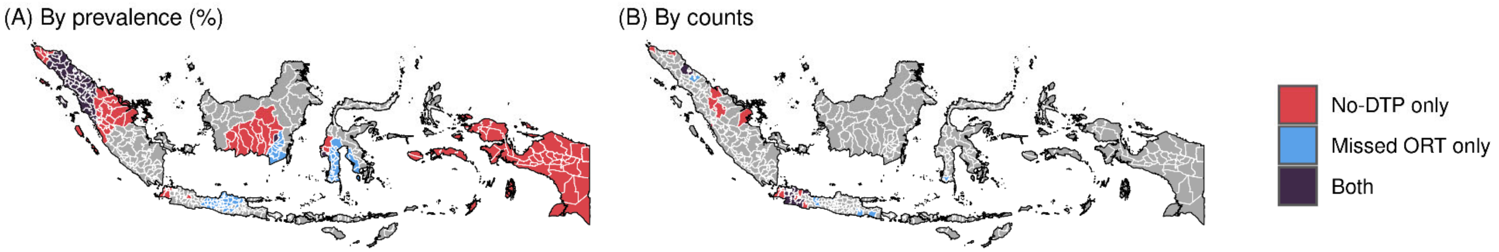

Supplemental Figure S69: Indonesia, LF

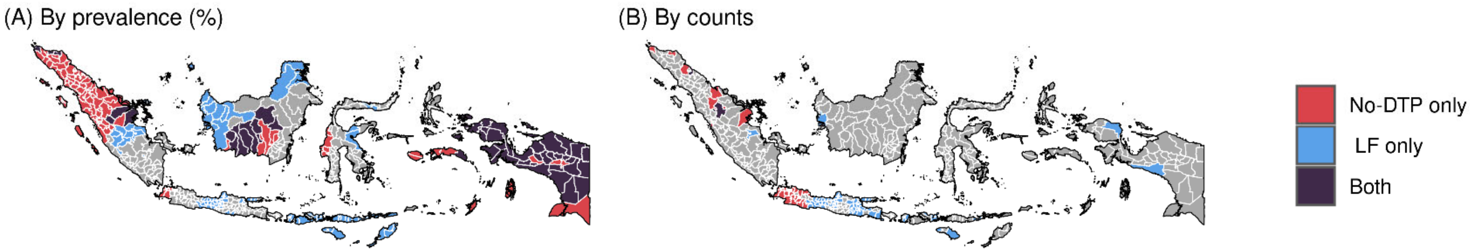

Supplemental Figures S70–S73: Multi-indicator overlap of country-specific highest quartiles.
For each country (Angola, DRC, Ethiopia and Indonesia), color given in each district indicates the number of indicators designated in the highest quartile in that district. Districts outlined in white indicate those where no-DTP is among the indicators in the highest quartile. The map on the left (a) shows categorization based on prevalence, and the map on the right (b) shows categorization based on counts.

Supplemental Figure S70: Angola

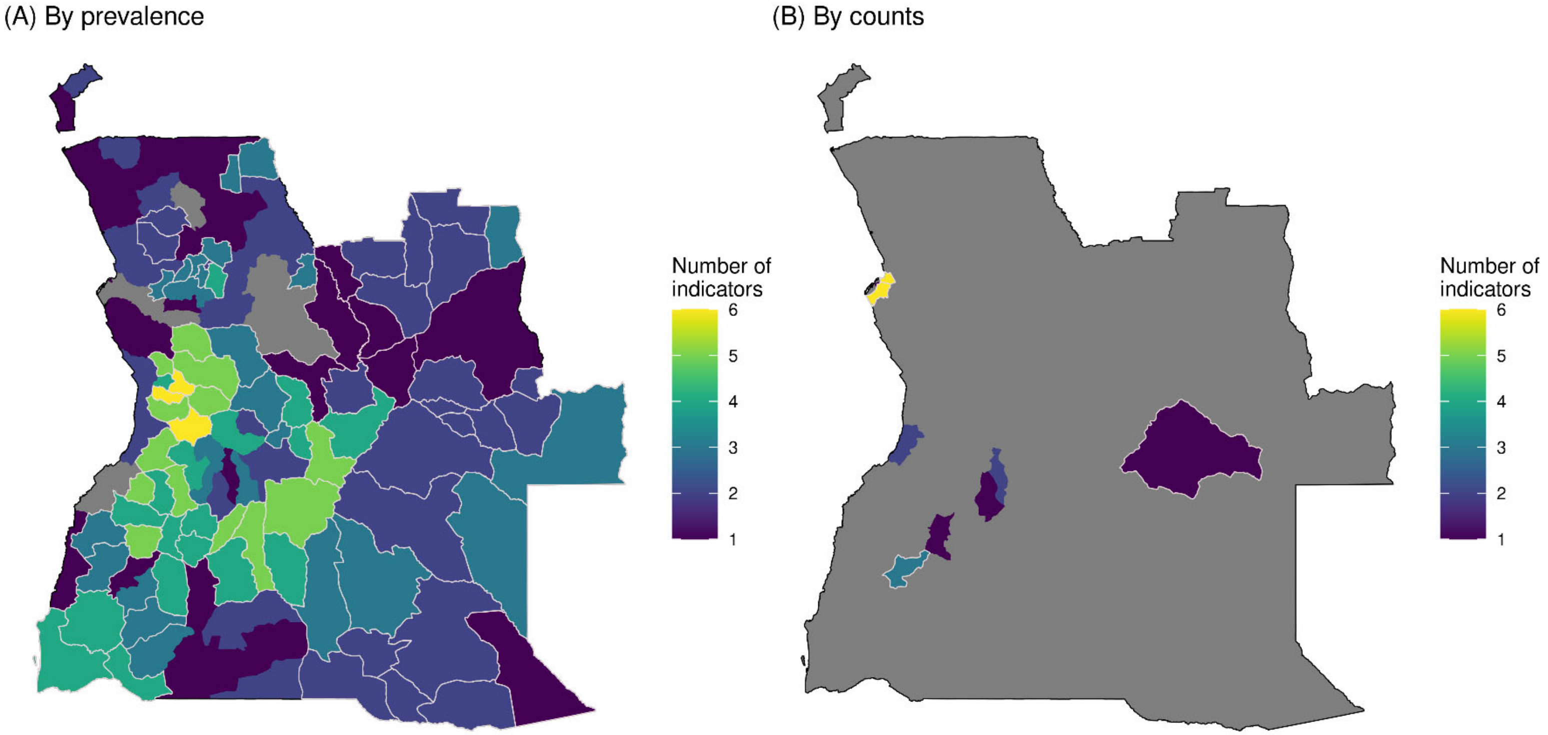

Supplemental Figure S71: DRC

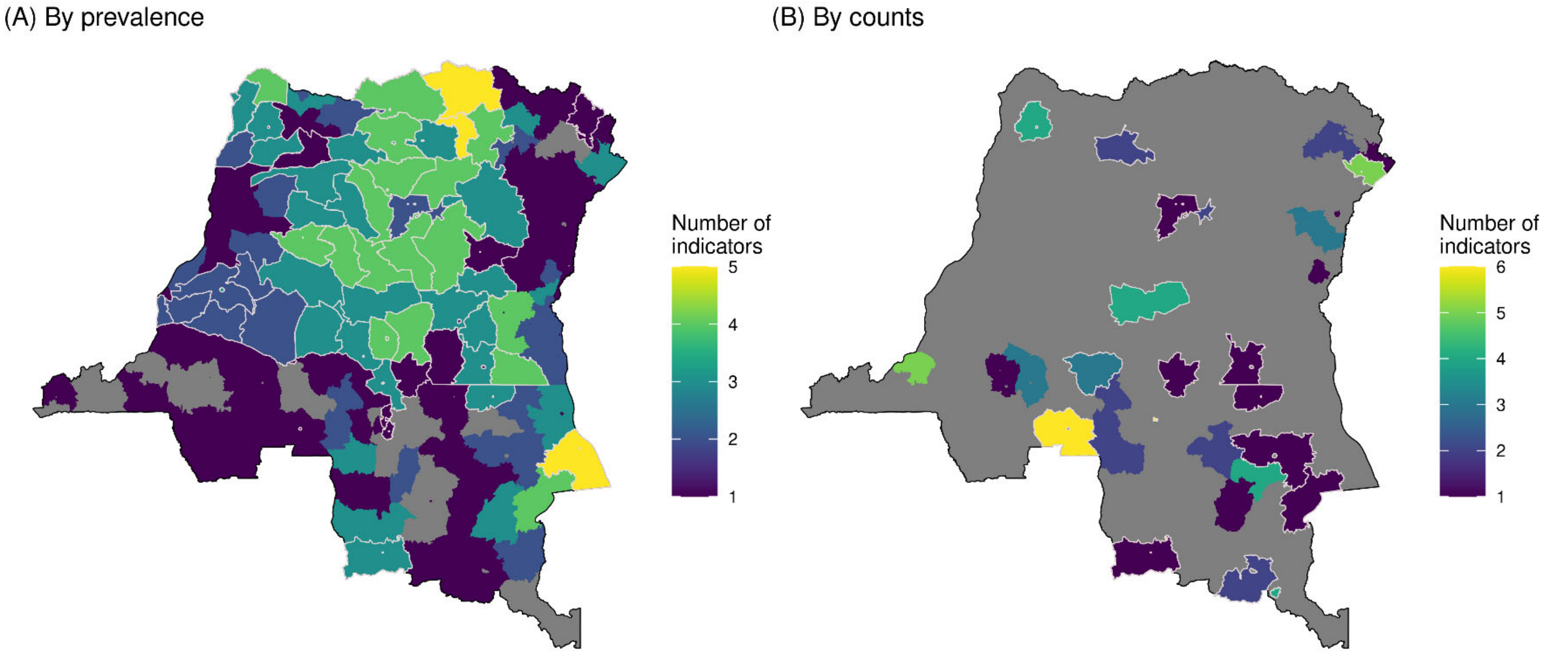

Supplemental Figure S72: Ethiopia

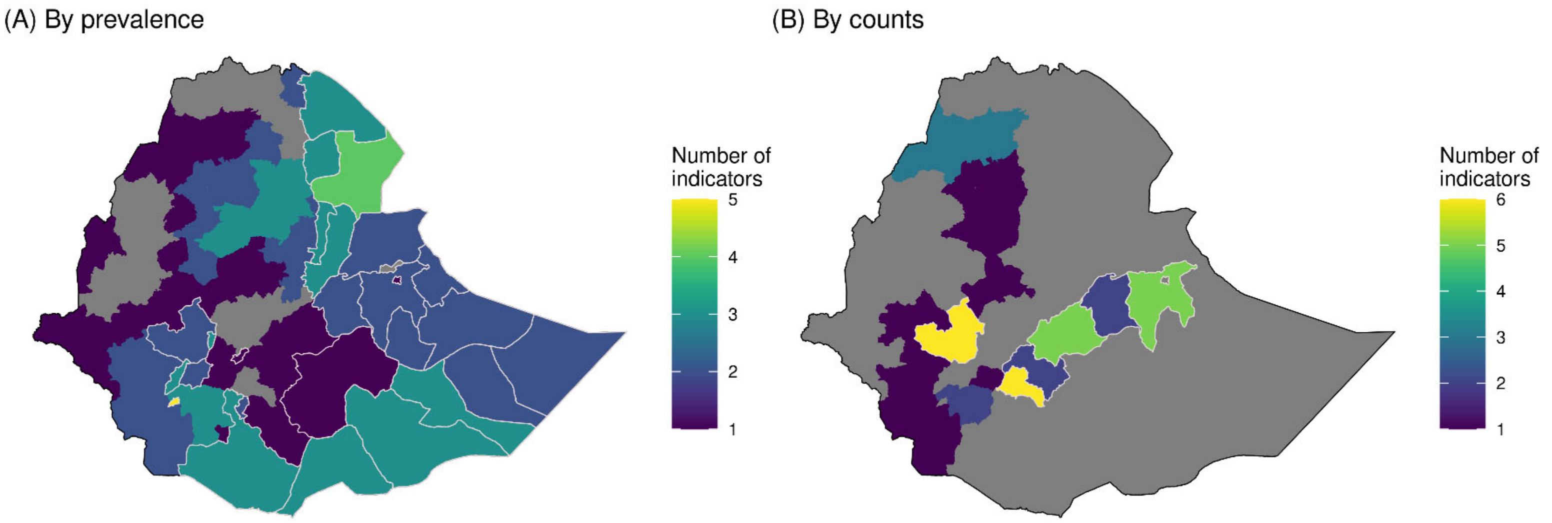

Supplemental Figure S73: Indonesia

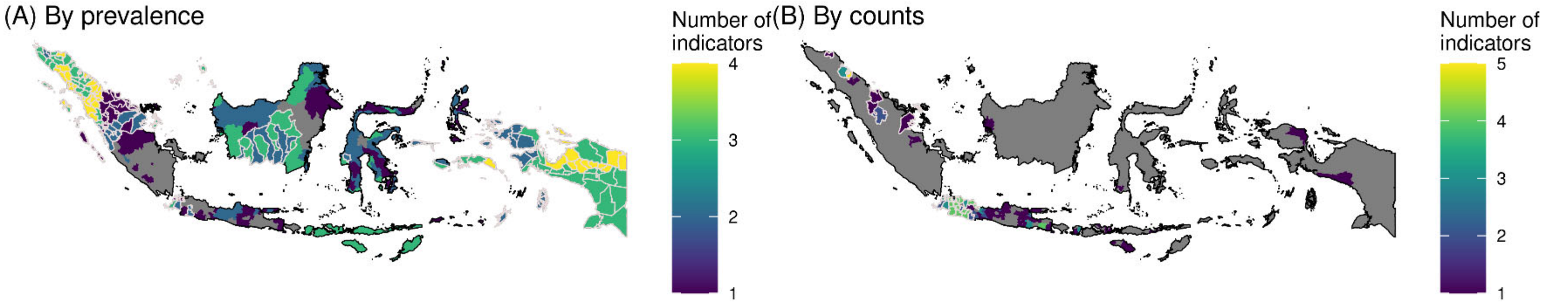

Supplemental Figures S74–S77: Multinational all quartile overlap by prevalence.
Multinational overlap no-DTP and each given indicator (U5M, missed ORT, missed ITNs and LF), for all categorization quartiles, based on prevalence. Ranges for population-weighted quartiles across the five example countries combined for no-DTP and children with stunting are delineated in the bivariate color legend (bottom right).

Supplemental Figure S74: U5M

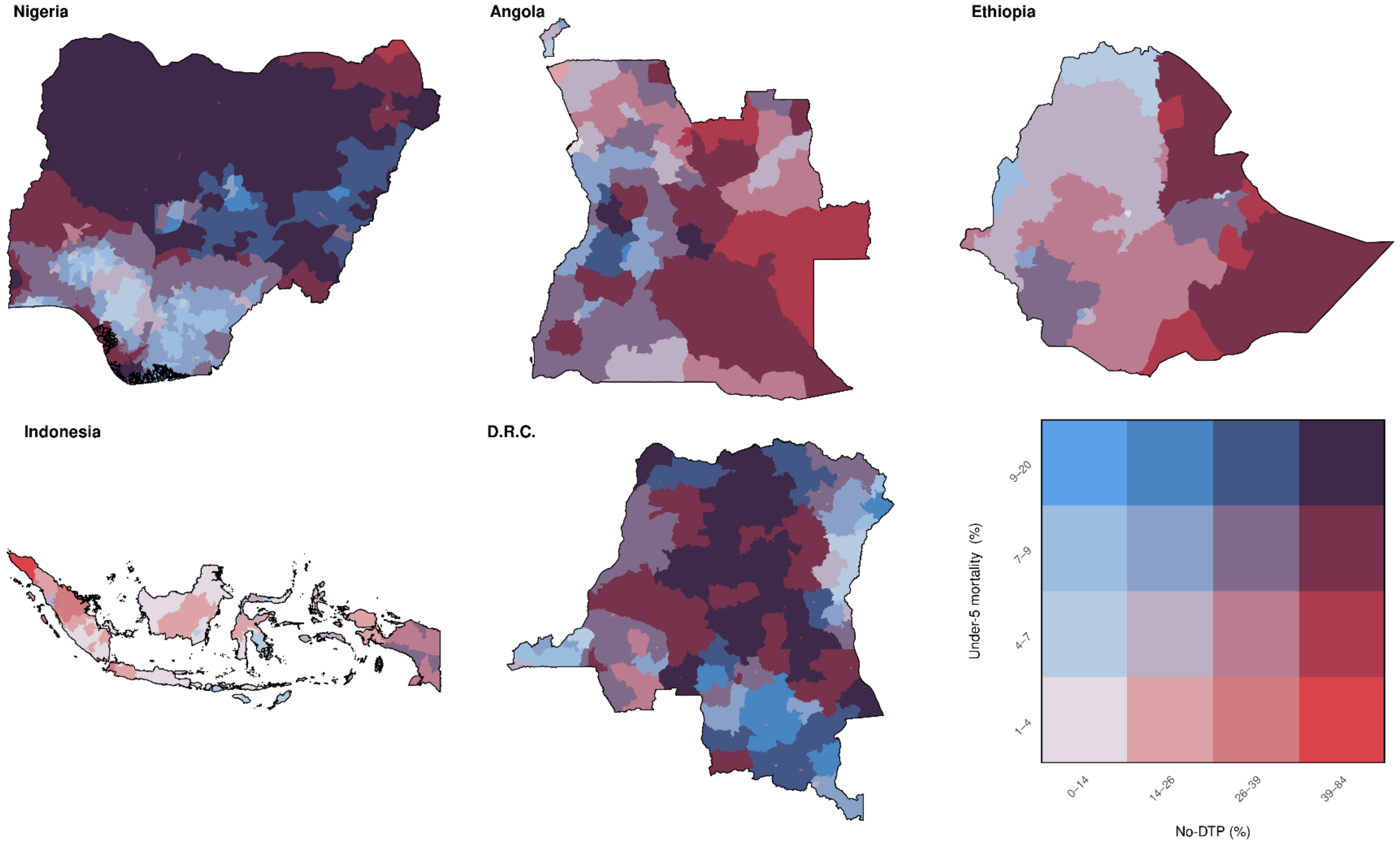

Supplemental Figure S75: Missed ITNs

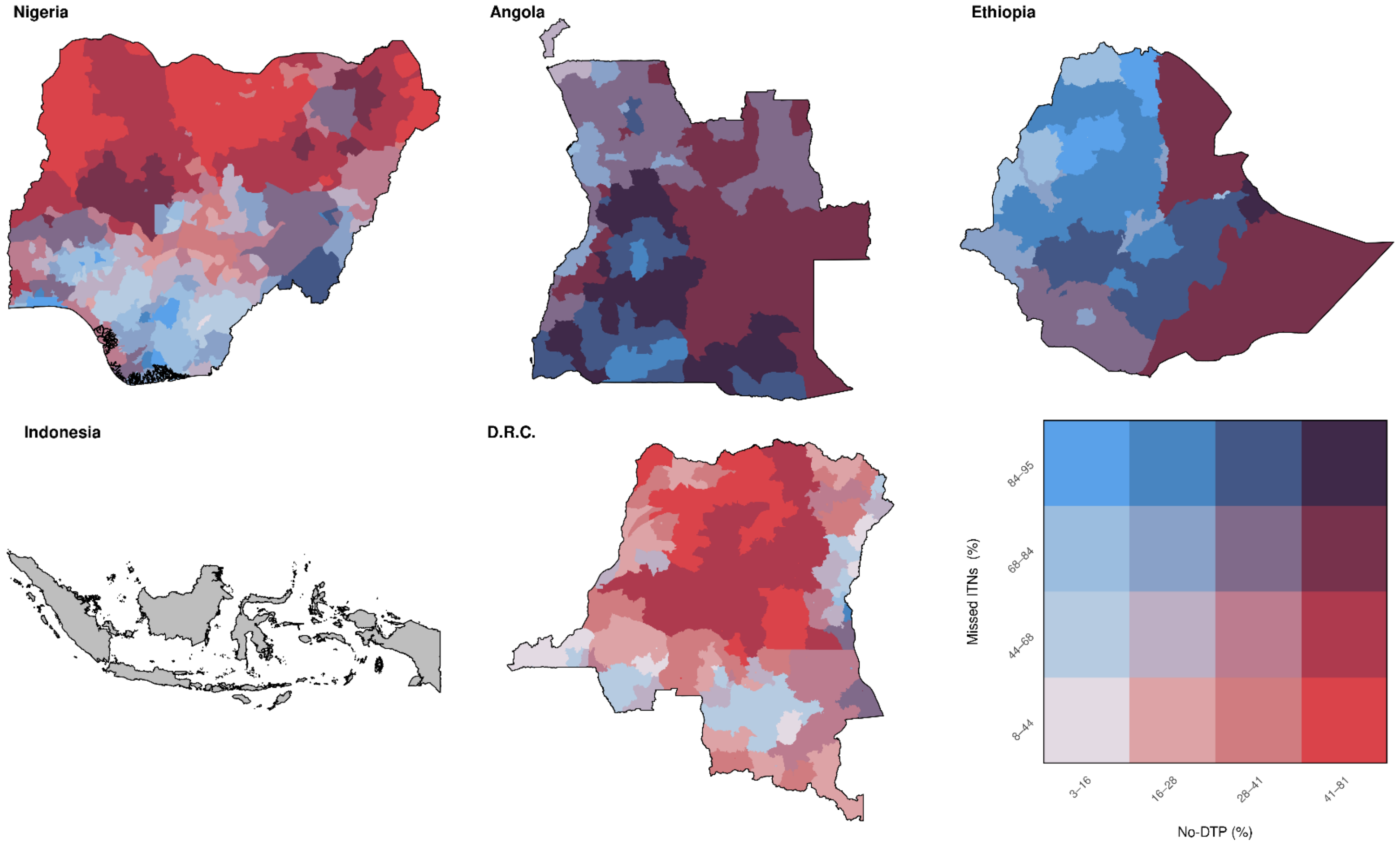

Supplemental Figure S76: Missed ORT

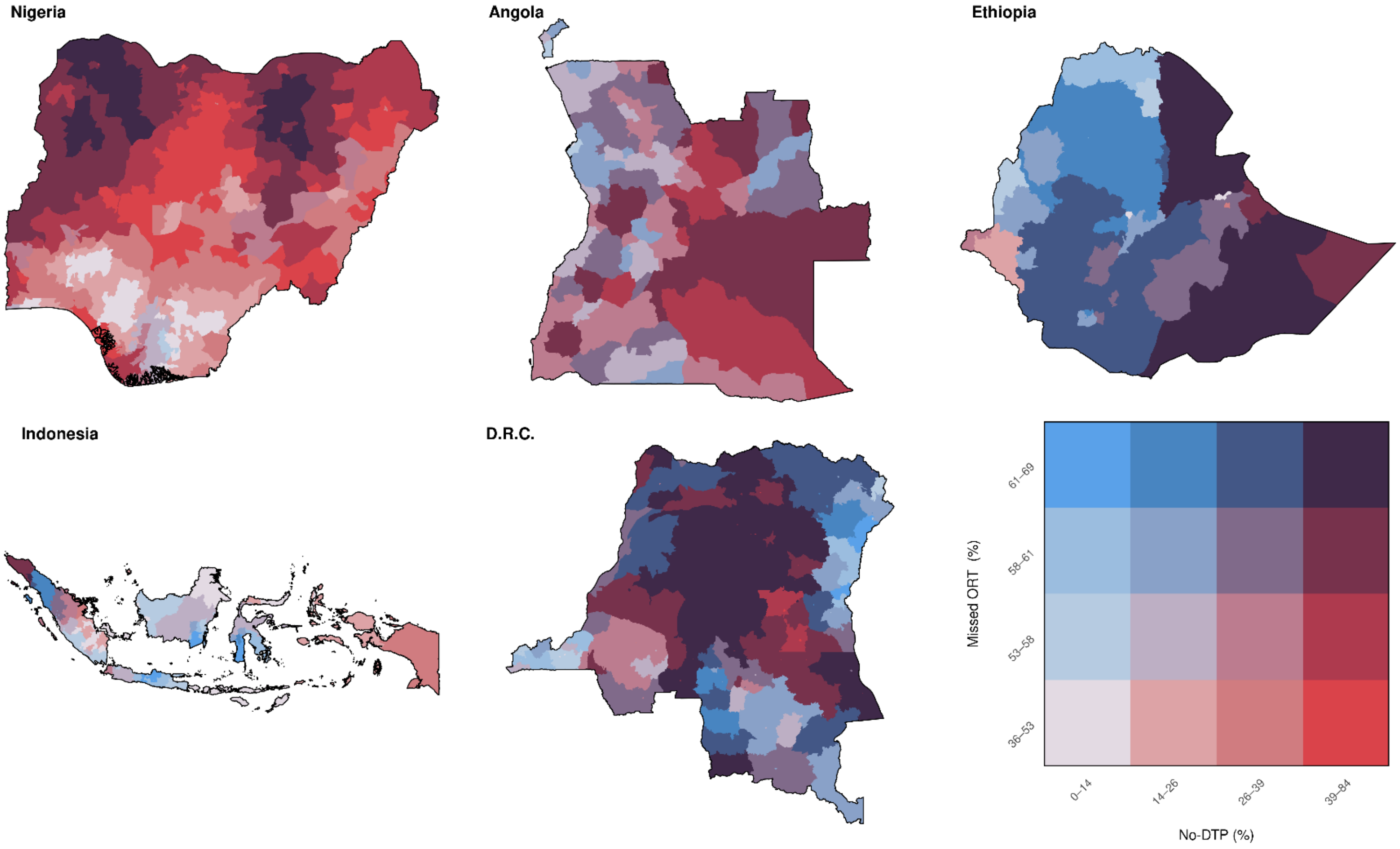

Supplemental Figure S77: LF

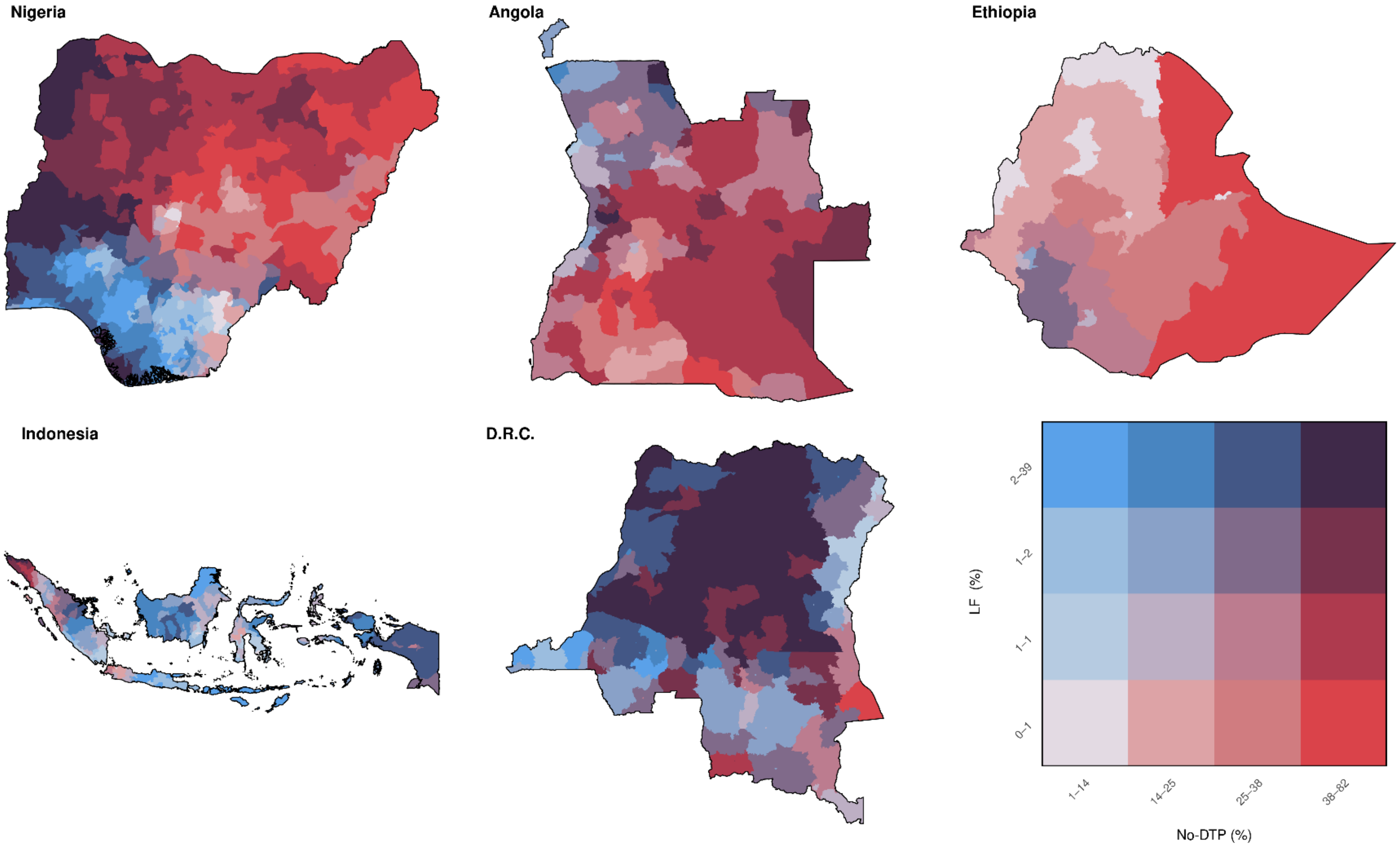

Supplemental Figures S78–S81: Multinational highest quartile overlap by prevalence.

Multinational overlap no-DTP and each given indicator (U5M, missed ORT, missed ITNs and LF), for highest quartiles only, based on prevalence. Districts in red are in the highest quartile for no-DTP only, blue are in the highest quartile for the comparator indicator only, and purple are in the highest quartile for both indicators.

Supplemental Figure S78: U5M

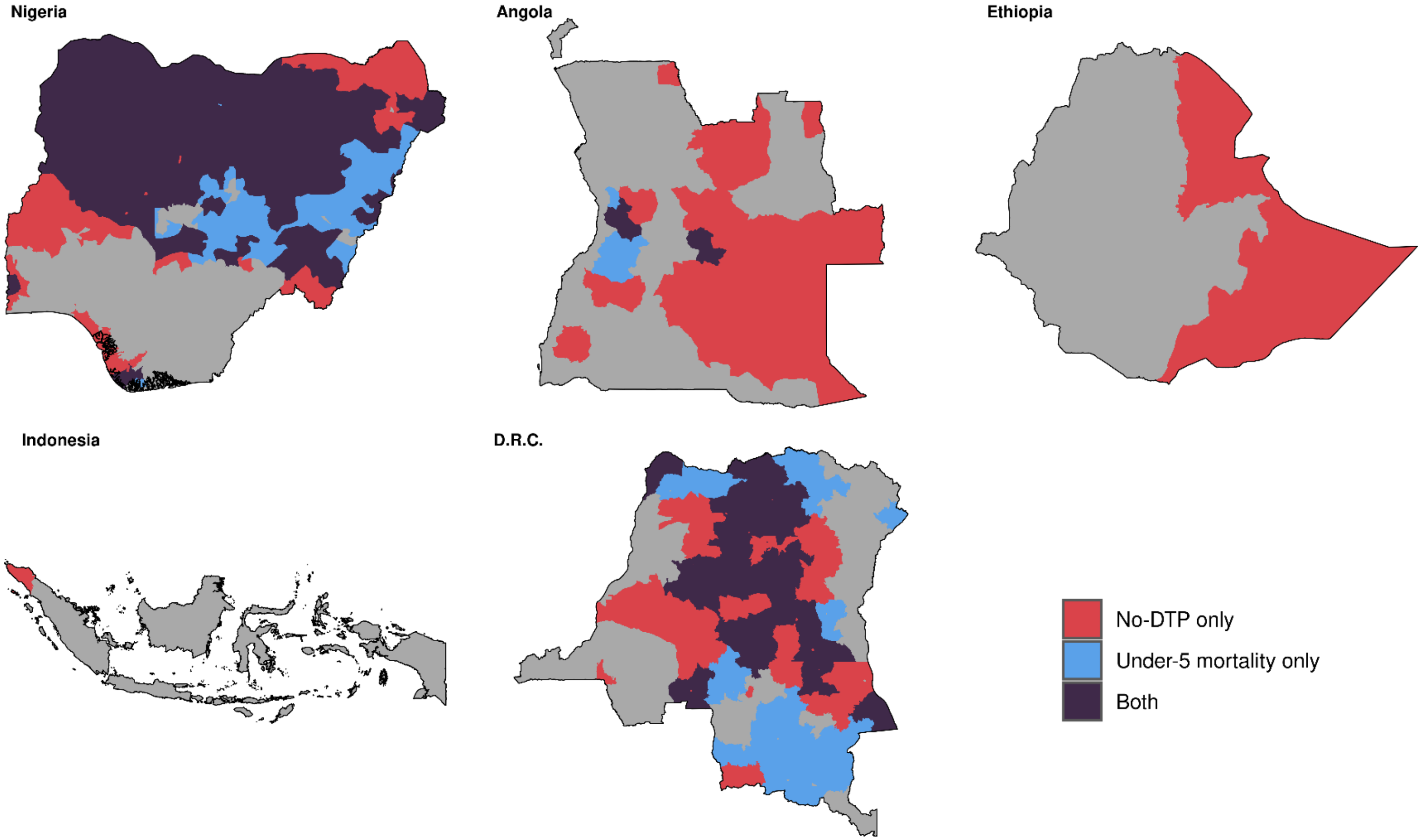

Supplemental Figure S79: Missed ITNs

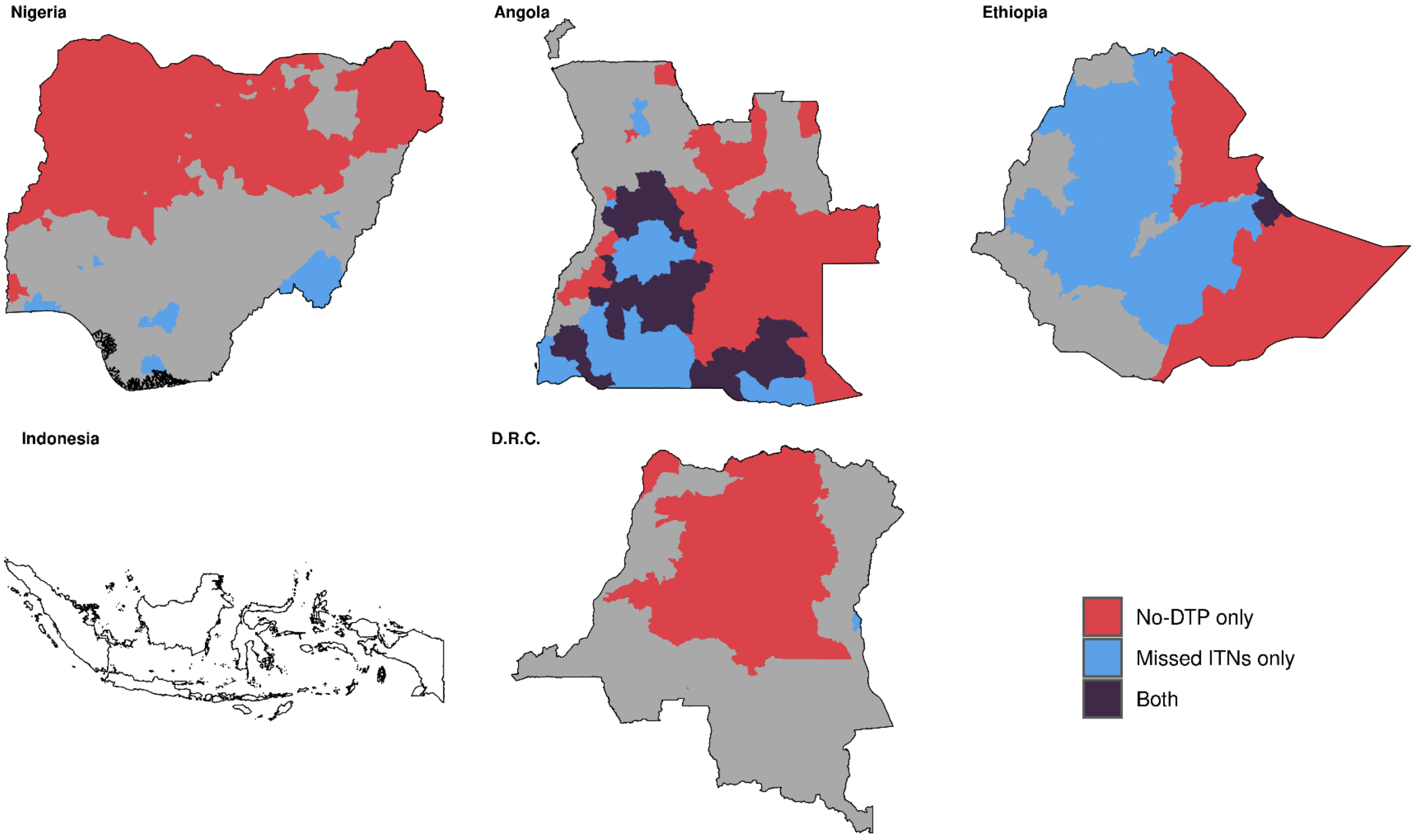

Supplemental Figure S80: Missed ORT

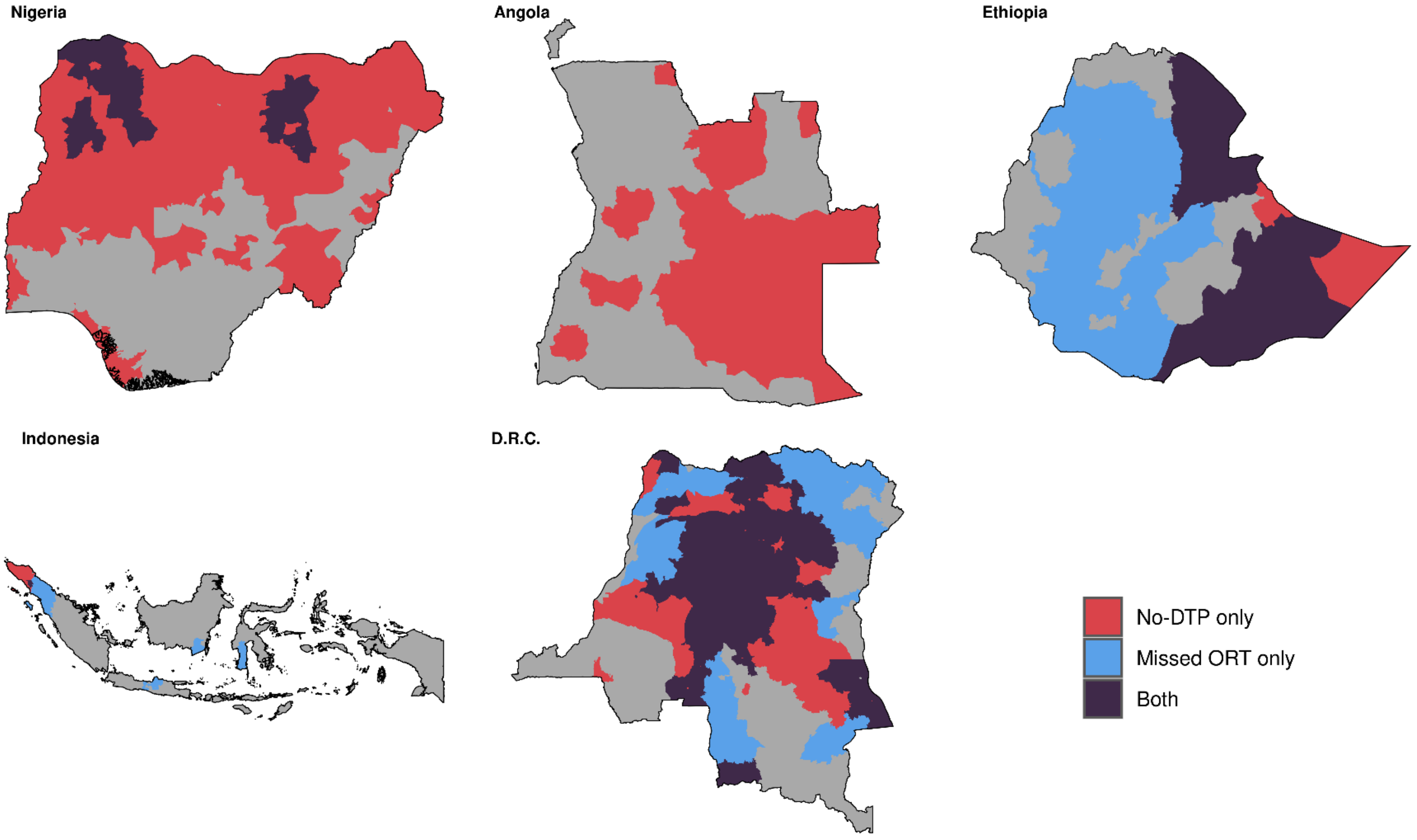

Supplemental Figure S81: LF

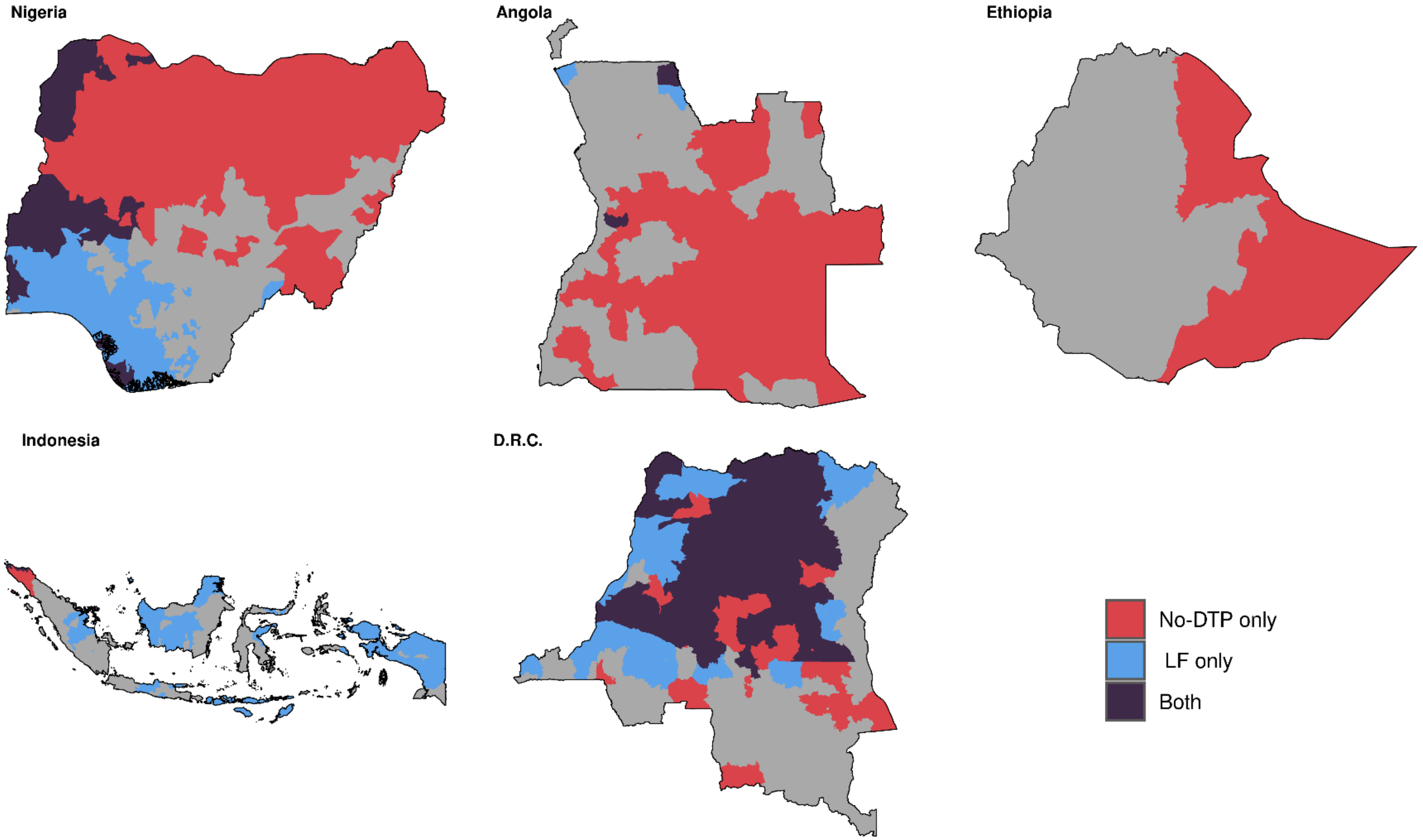

Supplemental Figures S82–S85: Multinational all quartile overlap by counts.
Multinational overlap no-DTP and each given indicator (U5M, missed ORT, missed ITNs and LF), for all categorization quartiles, based on counts. Ranges for population-weighted quartiles across the five example countries combined for no-DTP and children with stunting are delineated in the bivariate color legend (bottom right).

Supplemental Figure S82: U5M

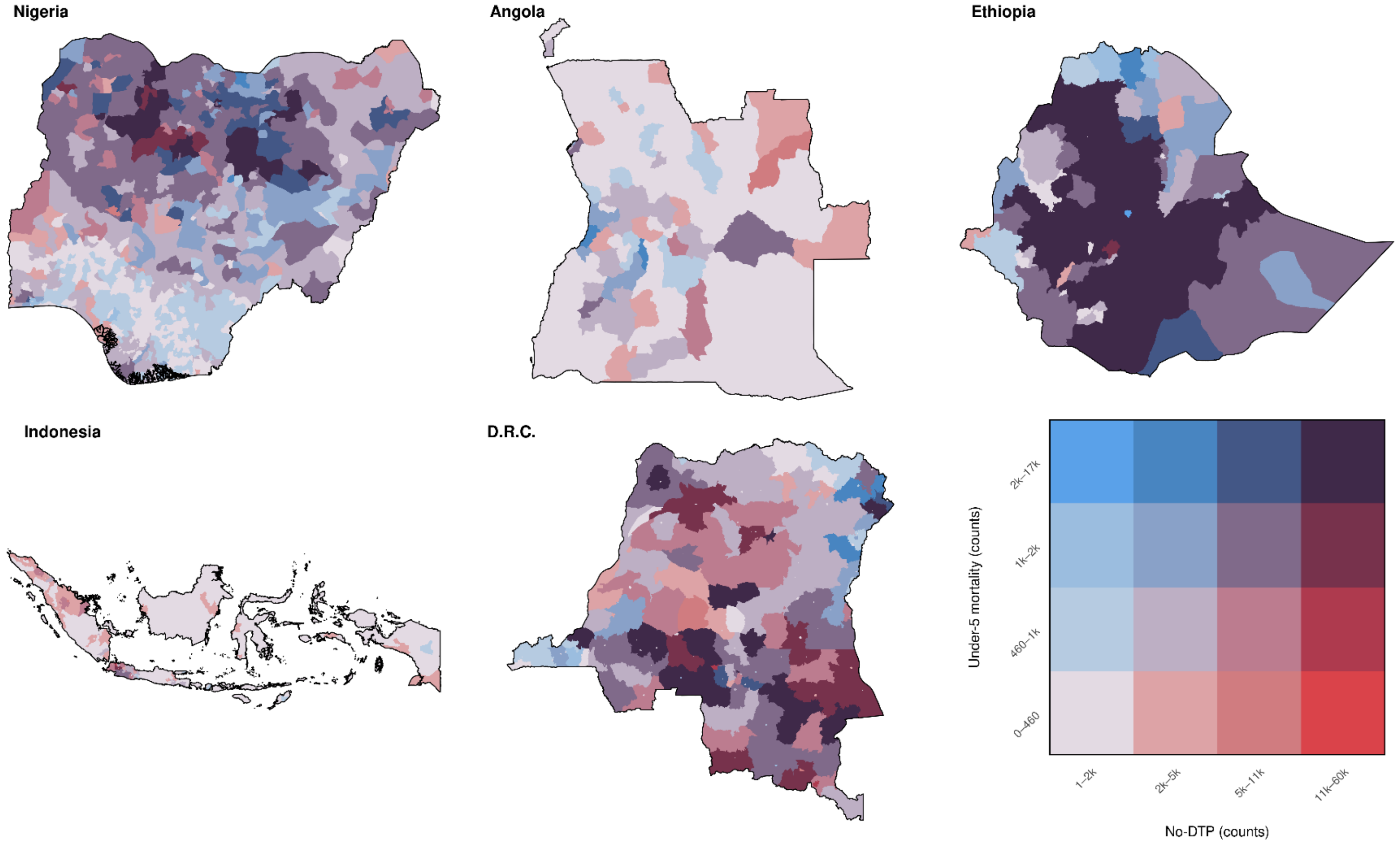

Supplemental Figure S83: Missed ITNs

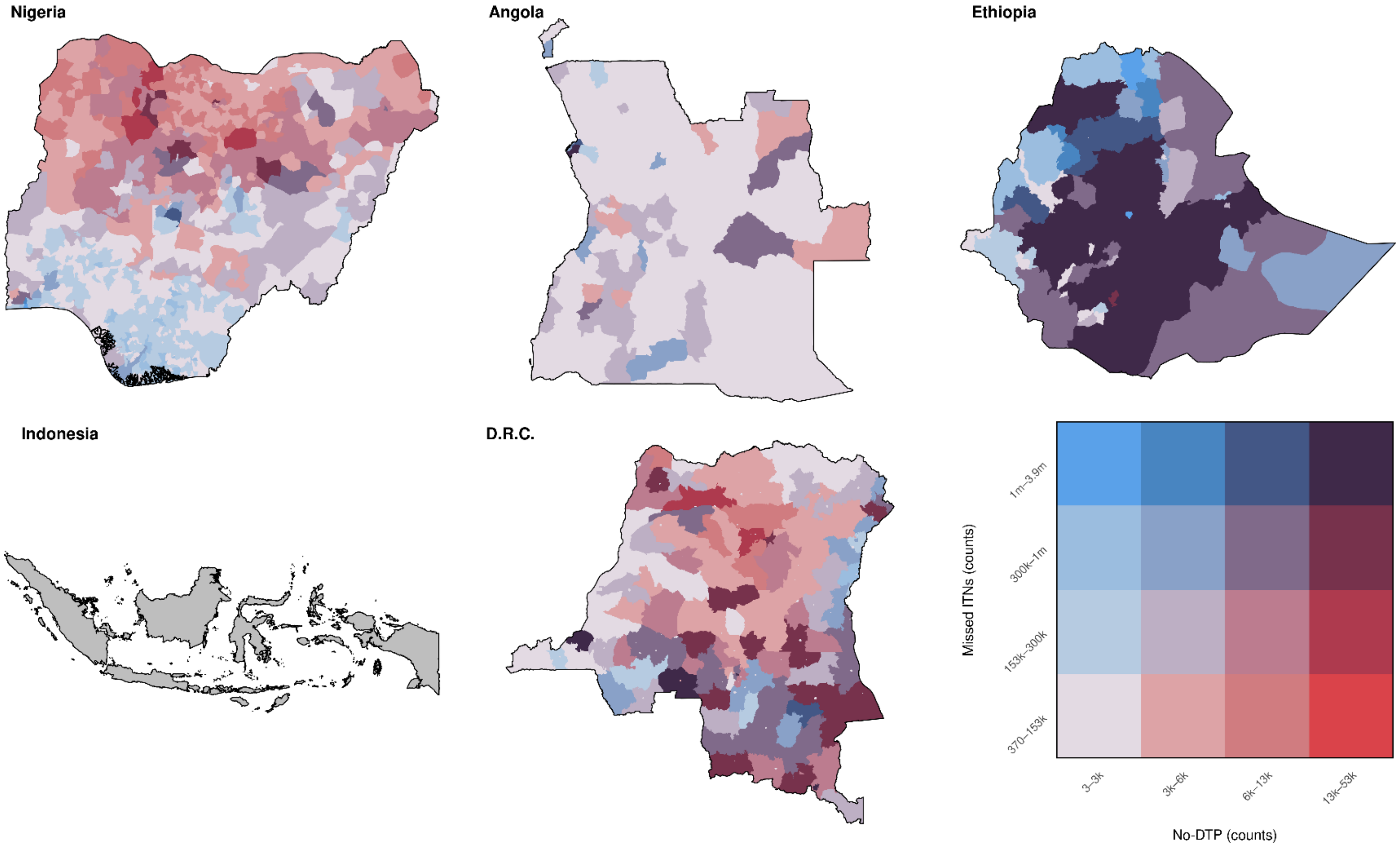

Supplemental Figure S84: Missed ORT

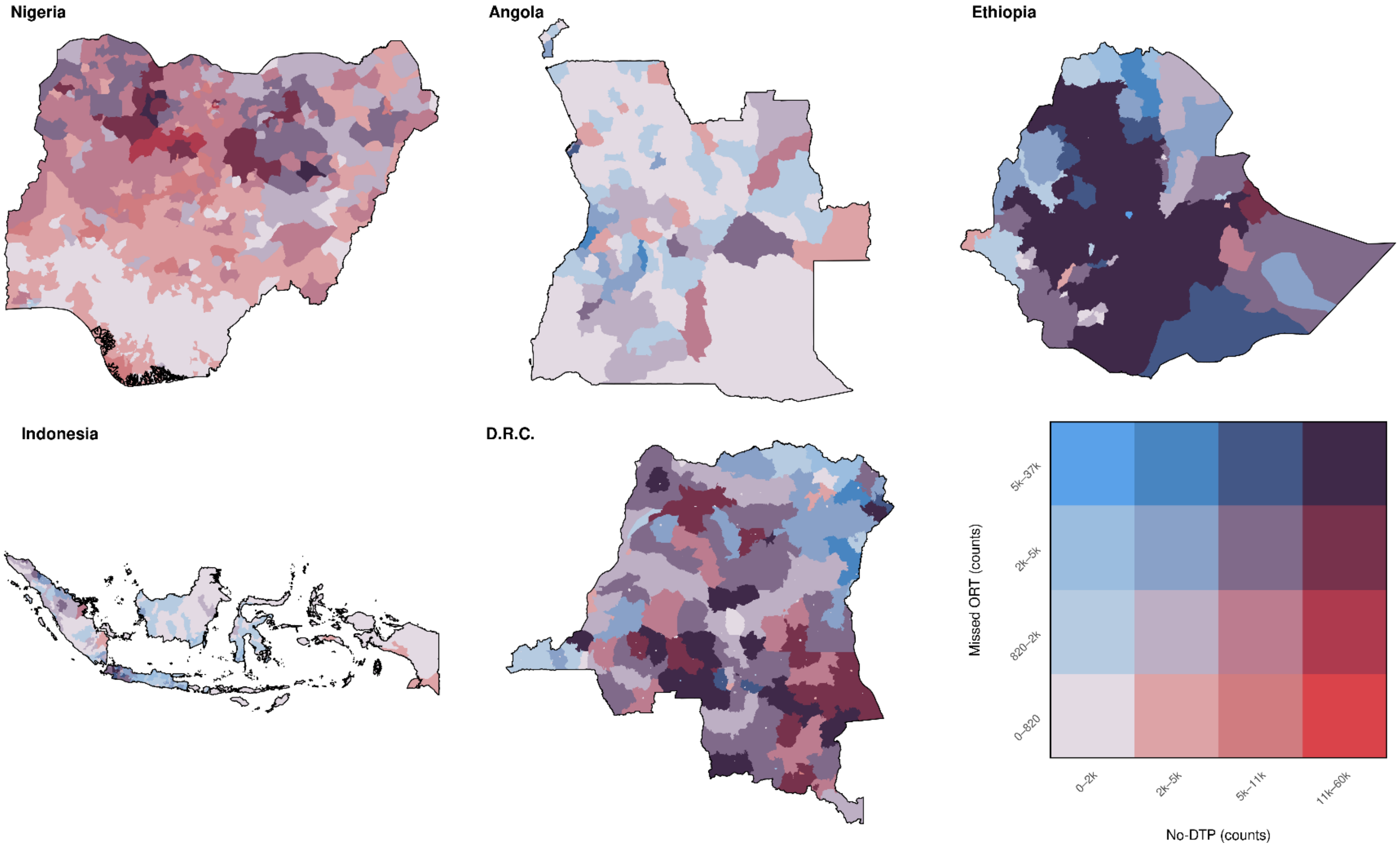

Supplemental Figure S85: LF

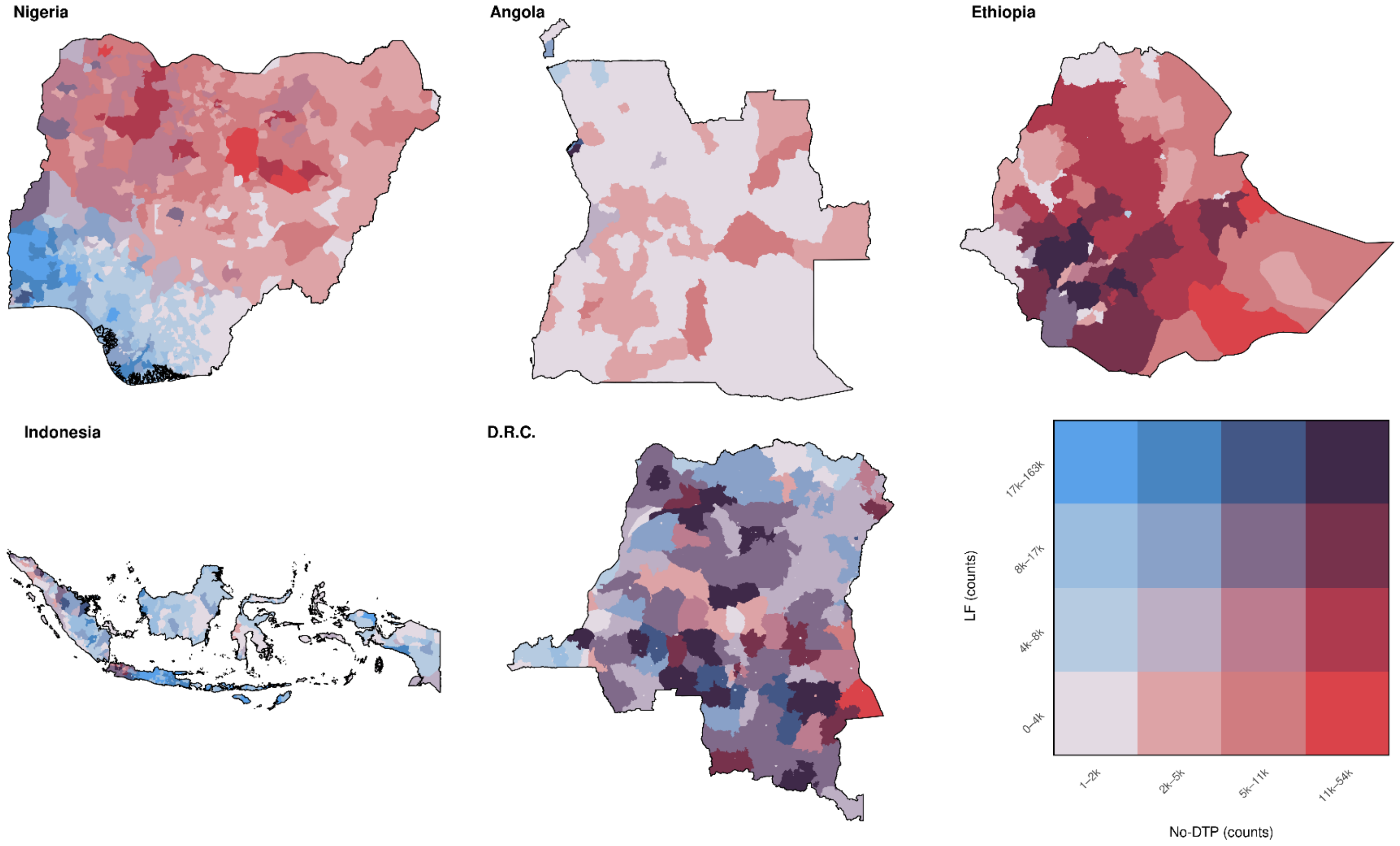

Supplemental Figures S86–S89: Multinational highest quartile overlap by counts.
Multinational overlap no-DTP and each given indicator (U5M, missed ORT, missed ITNs and LF), for highest quartiles only, based on counts. Districts in red are in the highest quartile for no-DTP only, blue are in the highest quartile for the comparator indicator only, and purple are in the highest quartile for both indicators.

Supplemental Figure S86: U5M

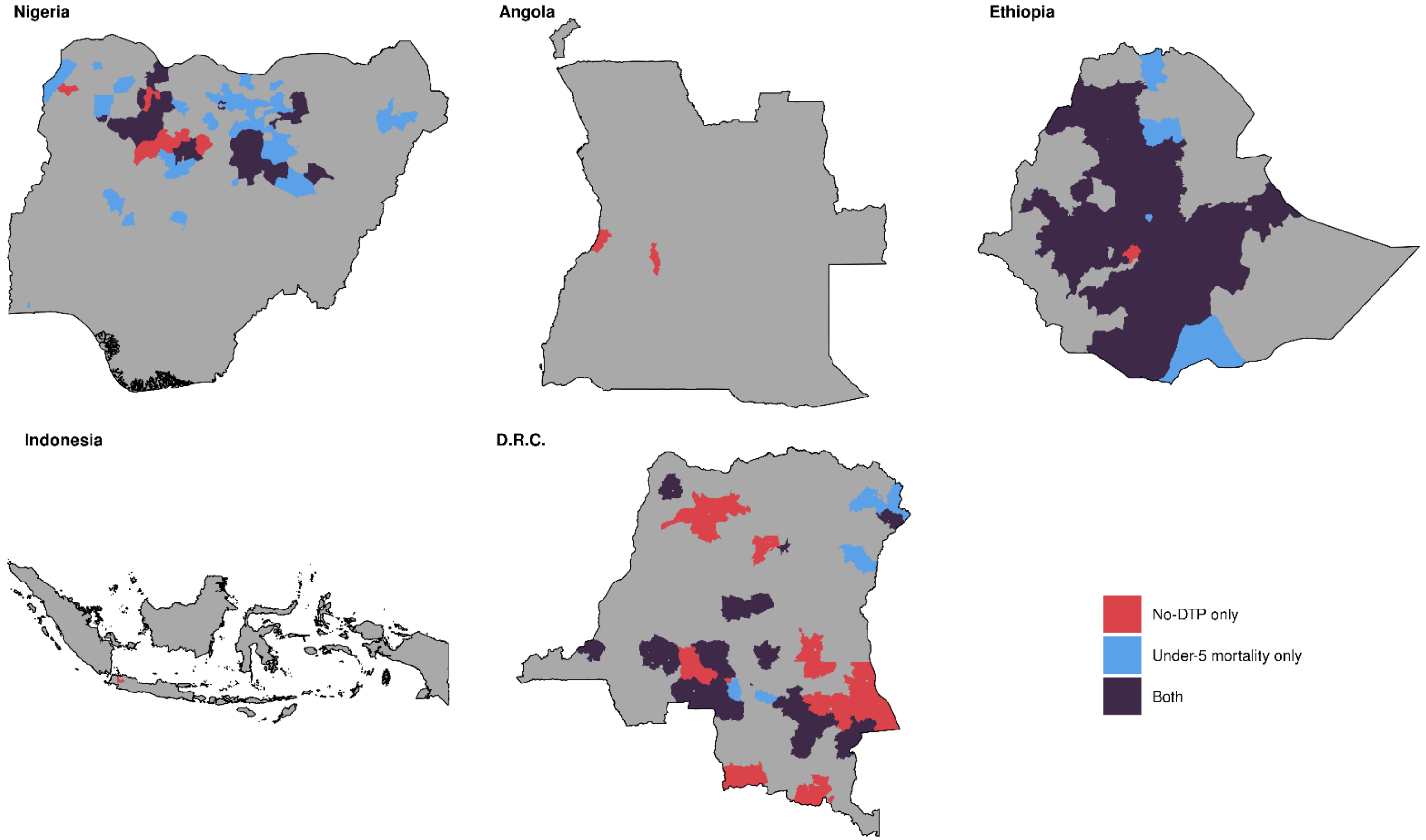

Supplemental Figure S87: Missed ITNs

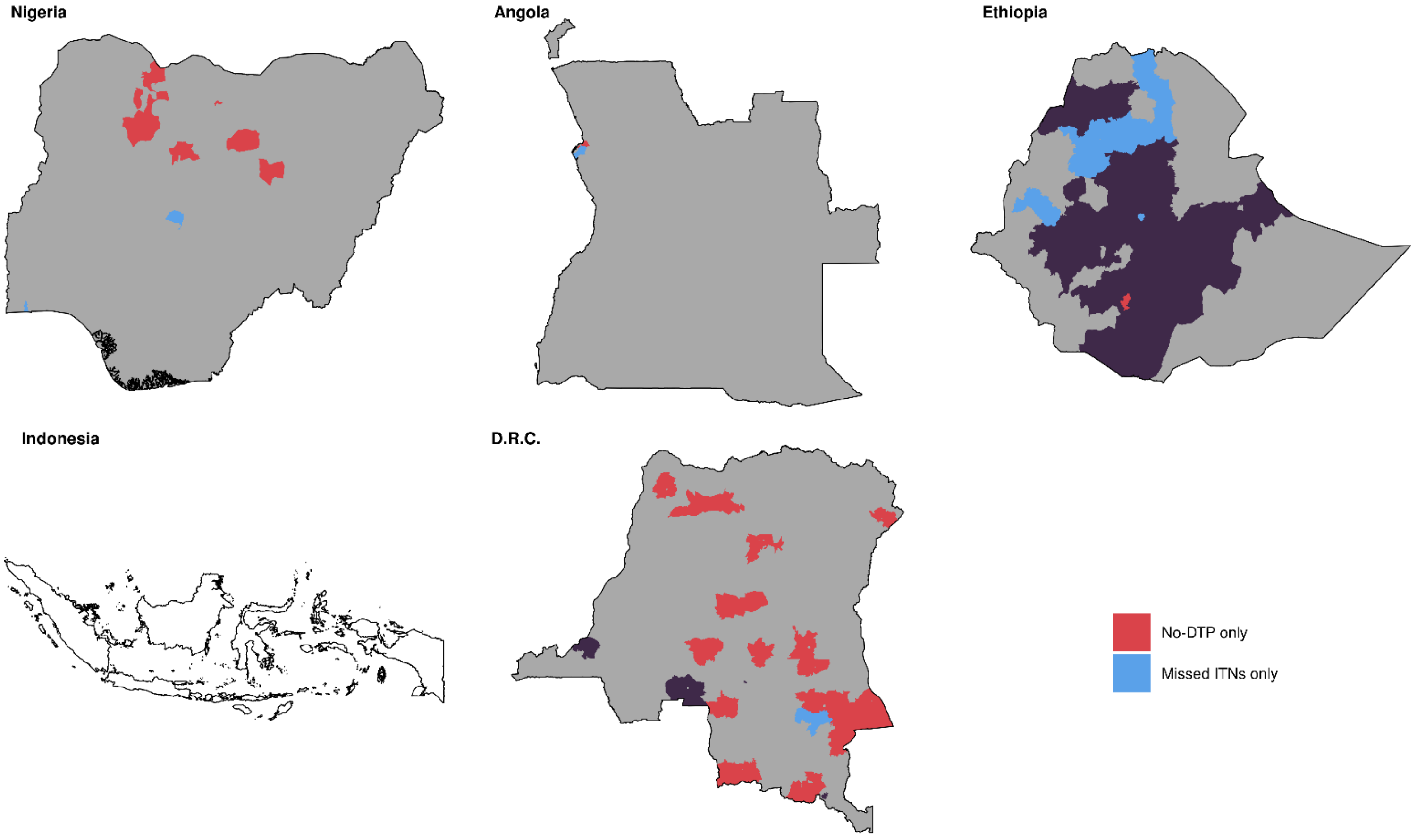

Supplemental Figure S88: Missed ORT

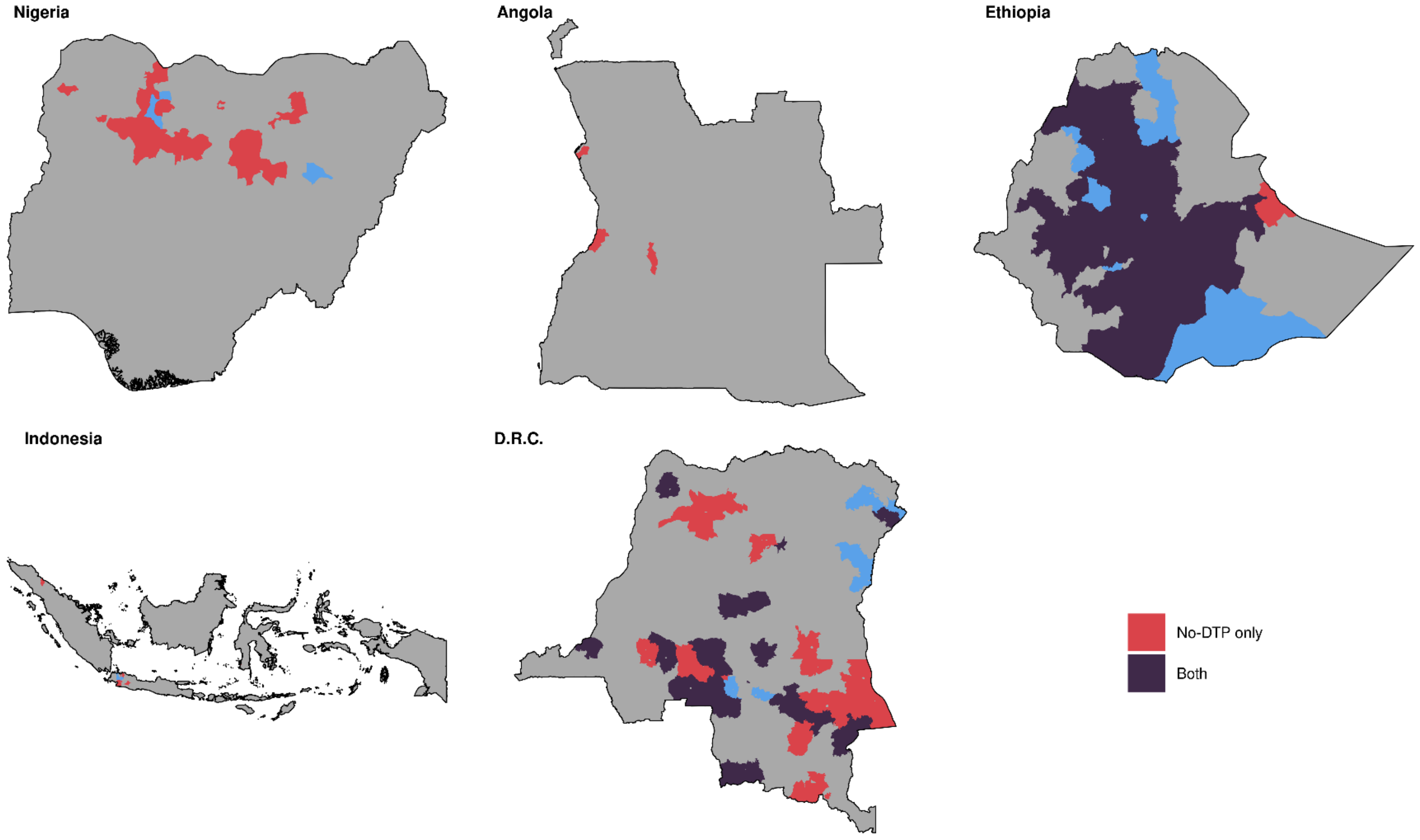

Supplemental Figure S89: LF

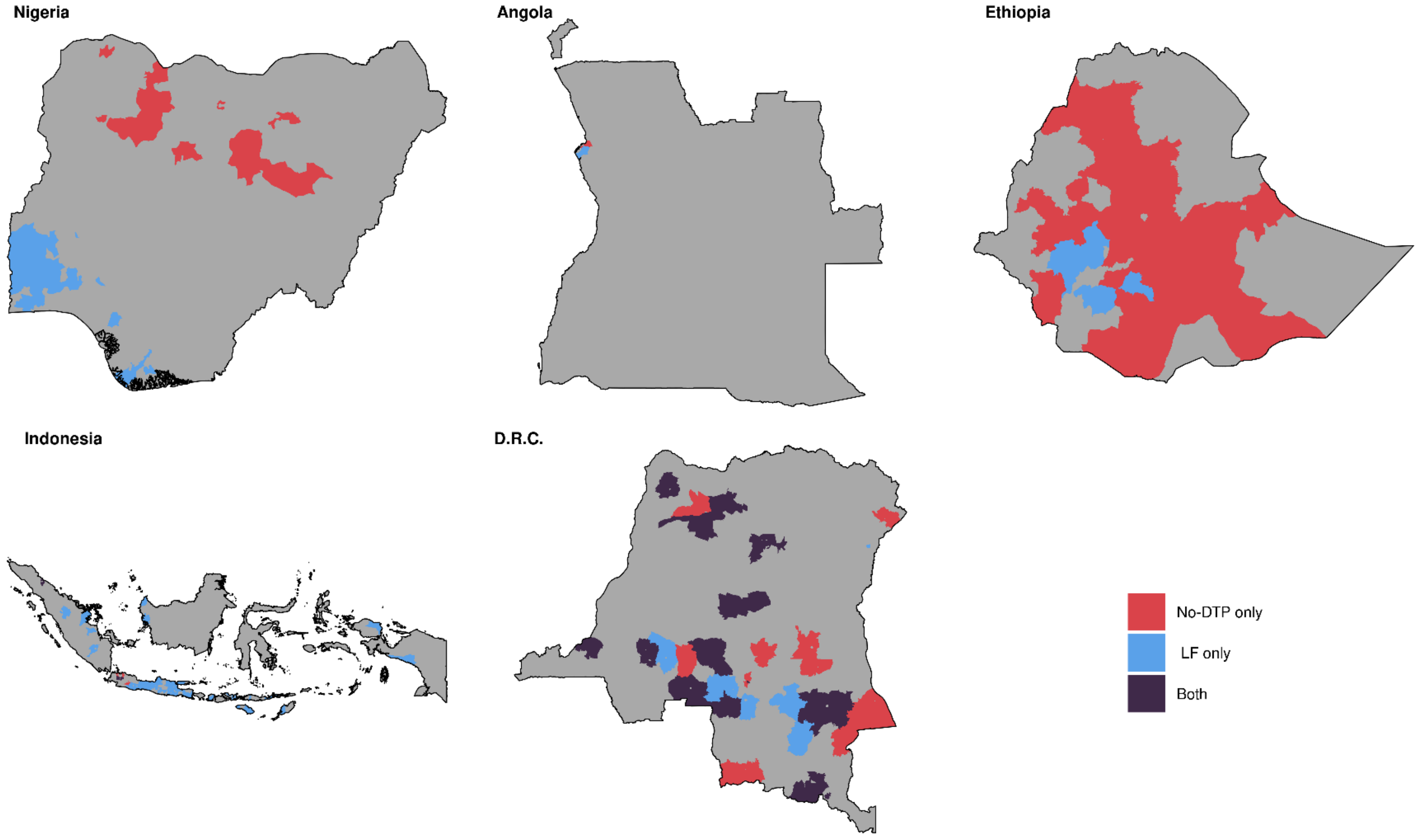

Supplement: Supplementary file 1 [file vaccines-11-00802-s001.zip › vaccines-2255180-supplementary.pdf]
